# Metabolomics-Guided Discovery of Bipolarolides H–O, New Ophiobolin-Type Sesterterpenes with Antibacterial Activity from the Marine-Derived Fungus *Uzbekistanica storfjordensis* sp. nov.

**DOI:** 10.1021/acs.jnatprod.4c01105

**Published:** 2025-01-30

**Authors:** Sailesh Maharjan, Johan Mattias Isaksson, Monika Krupova, Teppo Rämä, Kine Østnes Hansen, Jeanette Hammer Andersen, Espen Holst Hansen

**Affiliations:** † Marbio, Norwegian College of Fishery Science (NFH), Faculty of Biosciences, Fisheries, and Economics, UiT-The Arctic University of Norway, Tromsø 9037, Norway; ‡ Department of Pharmacy (IFA), Faculty of Health Sciences, UiT-The Arctic University of Norway, Tromsø 9037, Norway; § Department of Chemistry (IK), Faculty of Science and Technology, UiT-The Arctic University of Norway, Tromsø 9037, Norway; ∥ Hylleraas Centre for Quantum Molecular Sciences, Department of Chemistry (IK), Faculty of Science and Technology, UiT-The Arctic University of Norway, Tromsø 9037, Norway

## Abstract

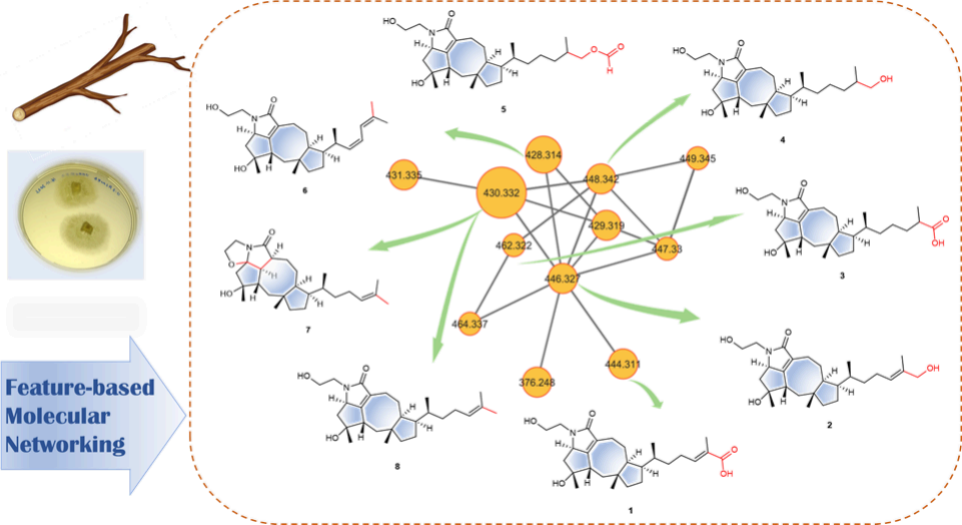

A marine-derived Pleosporales fungus, *Uzbekistanica storfjordensis*, was isolated from driftwood and described as a new species. The fungus was cultivated in liquid media and a molecular networking-driven approach was used to identify potential new secondary metabolites. The targeted compounds were isolated using preparative HPLC-MS, and through extensive spectroscopic analysis, eight new ophiobolin-type sesterterpenes, bipolarolides H–O (**1**–**8**), were identified. The absolute configurations of the compounds were determined by ECD assessment. Bipolarolide L (**5**), M (**6**), and O (**8**) exhibited inhibitory activity against *Streptococcus agalactiae* with MIC values of 86, 66, and 64 μM, respectively.

Marine fungi are an ecological group of fungi currently consisting of 2041 described species that inhabit marine habitats across tropical, temperate, and Arctic regions.^1^ While marine species show adaptation to the marine environment,^2^ the so-called marine-derived fungi are species isolated from the marine milieu without definitive evidence of adaptations specifically to this environment. Marine-derived fungi can be isolated from seawater, sediments, dead organic materials including driftwood, and less explored ecological niches such as marine plants (seaweeds, seagrasses, mangrove plants), vertebrates, and invertebrates.^3^ They are recognized as a notable source of new biologically active compounds with potential pharmacological applications. Many marine-derived fungi are saprotrophs classified within the Pleosporales (Dothideomycetes, Ascomycota) order, which also hosts fungi with other ecological strategies, such as parasites, pathogens, and endophytes.^4^ The order comprises 91 families that contain thousands of species in 614 genera.^5^*Uzbekistanica* is one of the newly introduced genera in *Pleosporales*.^6^ The genus and its type species, *U. rosae-hissaricae*, was first described as a saprobe from *Rosa hissarica* in Uzbekistan. Additional species have been described as saprotrophs of different woody plants in Europe and Asia. Currently, the genus consists of four species with names reflecting the host plant species or geographical origin: *U. rosae*-*hissaricae,*^6^*U. yakuthanika*,^6^*U. vitis-viniferae*,^7^ and *U. pruni*.^8^

Several bioactive secondary metabolites have been reported from fungi belonging to the *Pleosporales* order, including anti-inflammatory dimeric benzophenones polyketides,^9^ antimicrobial phaeosphaeridiols,^10^ chlamydosporols,^11^ ascosalipyrrolidinones,^12^ dihydrobenzofurans and xanthenes,^13^ antibacterial phenalenone and ergosterols,^14,15^ antimalarial and antiplasmodial palmarumycins,^16^ nodulisporacid and vermelhotin,^17^ cytotoxic polyketides and abscisic acid-type sesquiterpenes,^18−20^ antiproliferative tetramic acids and azaphilones,^21^ diketopiperazines and phthalides,^22^ nematicidal thailanones,^23^ and anti-HIV polyketide-nonribosomal peptide synthase (PKS-NRPS) hybrid metabolites.^24^ These compounds demonstrate that fungal genera within the *Pleosporales* order are attractive sources for exploring new bioactive metabolites in the drug discovery pipeline. However, the vast majority of the over 600 genera within *Pleosporales* have yet to be investigated for natural product discovery, including the genus *Uzbekistanica*. The isolate described here as a new species showed distinct differences in DNA sequence similarity, morphology, ecology, and geographical origin compared to the previously described *Uzbekistanica* species.

In our search for new bioactive natural products, the identification of previously undescribed marine-derived and driftwood-associated *Uzbekistanica* species has emerged as a promising avenue for discovering new chemistry. To comprehensively study the structural diversity of bioactive secondary metabolites from this fungus, mass spectrometry-based metabolomics was used to investigate cultures of the fungus *Uzbekistanica* to accelerate the structure-based discovery of new metabolites. The application of feature-based molecular networking on the Global Natural Product Social Molecular Networking (GNPS) platform as a tool for secondary metabolite profiling guided the isolation of eight ophiobolin-derived sesterterpenoid derivatives. These eight new variants of ophiobolin-type sesterterpenoids described herein represent the first compounds from the *Uzbekistanica* genus and Melanommataceae family. This paper describes the identification, isolation, and structure elucidation of these eight new metabolites from a new *Uzbekistanica* species, along with an evaluation of their antibacterial activity.
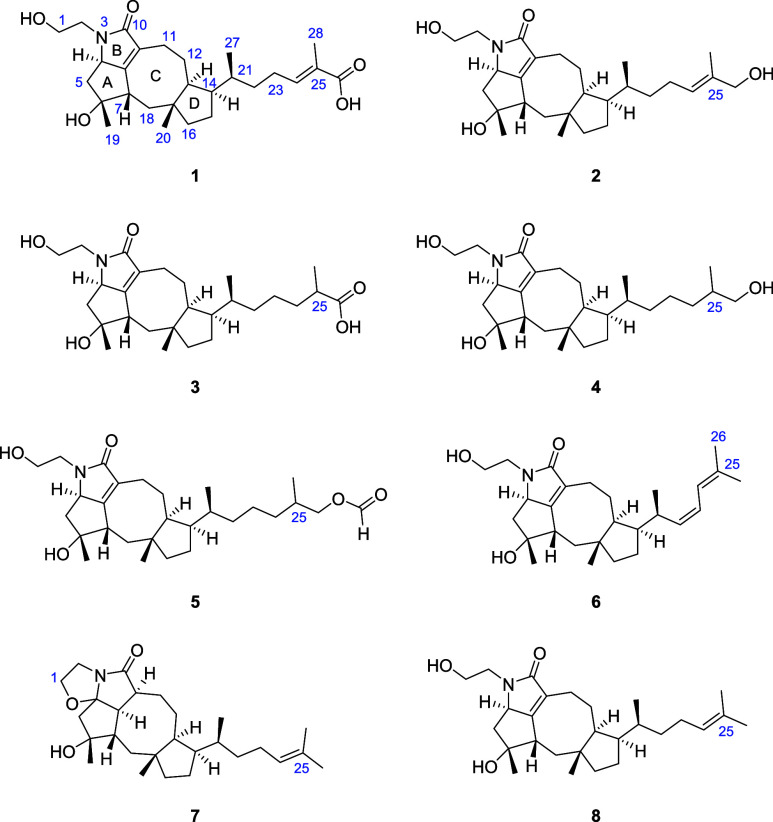


## Results and Discussion

### Morphological and Phylogenetic Analysis

**Figure 1:**
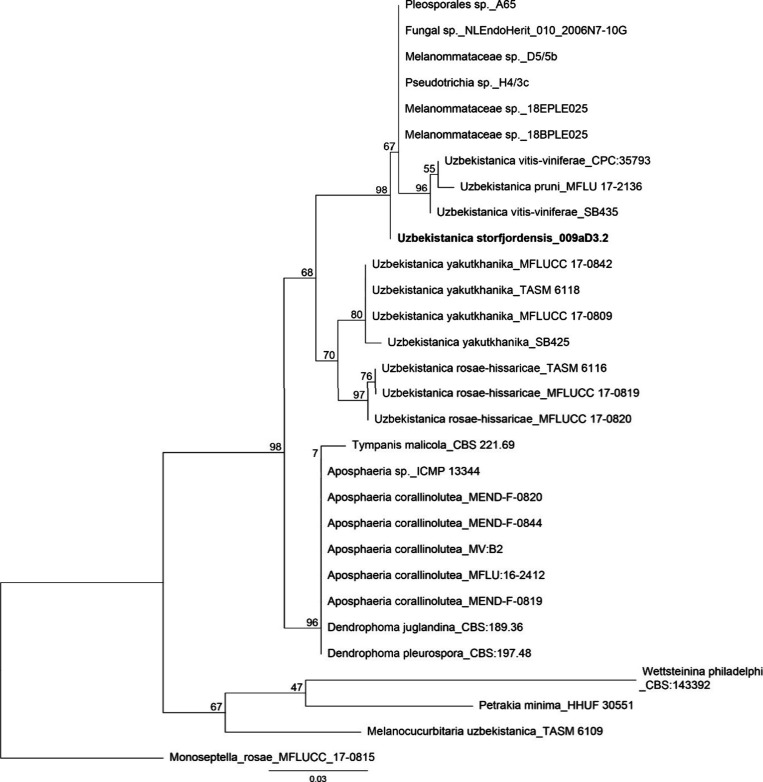
A best-scoring ML tree showing the placement of *U. storfjordensis* (in bold) within the Melanommataceae based on ITS sequences. Taxon names are followed by isolate names and GenBank accession numbers are listed in Table S1. The numbers at the nodes represent bootstrap support values.

Based on phylogenetic analysis of the internal transcribed spacer (ITS) (Figure S2) and 28S sequences (Figure S3) of nuclear rDNA, the isolate 009aD3.2 grouped within the recently described genus *Uzbekistanica*.^6^ The ITS tree shown in Figure 1 indicates that the isolate represents an undescribed *Uzbekistanica* species, which was confirmed by morphological investigations (Supporting Information). Consequently, the fungus was described as a new species *Uzbekistanica storfjordensis* (Figure S1). The fungus is only known from its type locality and was originally isolated by Rämä et al. (2014).^25^ All other described *Uzbekistanica* species are sourced from terrestrial environments as saprotrophs from different hosts predominantly at high altitudes, whereas *U. storfjordensis* is the first species isolated from the marine environment and at high latitudes.

### Molecular Networking-Based Prioritization of the Isolation Workflow

**Figure 2:**
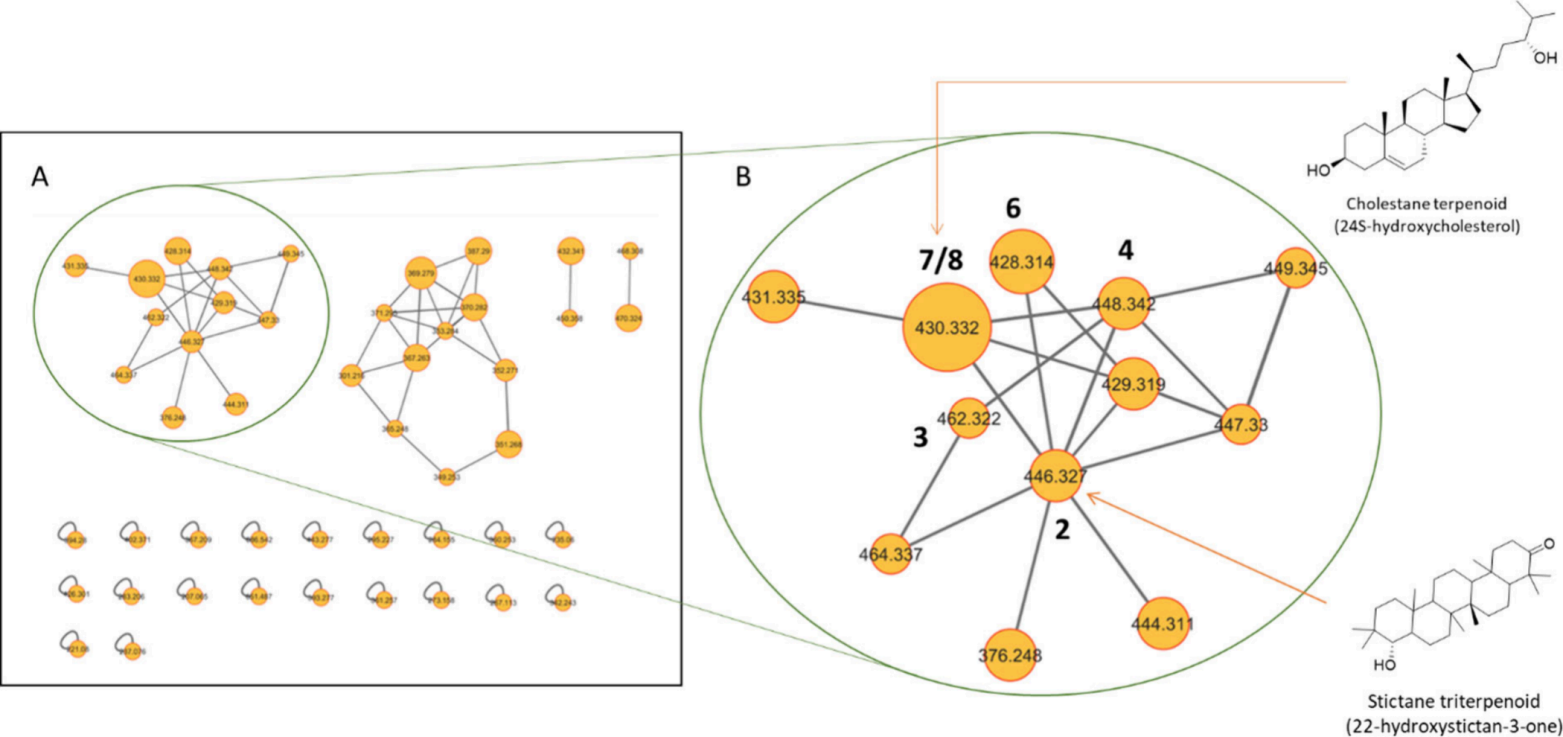
(A) Feature-based molecular network generated from the extract of *U. storfjordensis* with the clusters correlating to terpenoid, (B) Cluster A with proposed terpenoid hits, including compounds **2**–**8**, except **5**.

The fungal extract was analyzed using UPLC-MS/MS in the positive electrospray mode (Orbitrap). The resulting MS^2^ data were processed using MZmine and visualized through feature-based molecular networking to prioritize the compounds for isolation. A total of 123 MS^2^ spectra generated 47 nodes, of which 27 nodes were grouped to form four clusters (Figure 2). Cluster A was identified as a terpenoid cluster because dereplication against the GNPS libraries proposed two hits: namely, *Stictane* triterpenoid (22-hydroxystictan-3-one) and cholestane terpenoid (24S-hydroxycholesterol), corresponding to the nodes with *m*/*z* 446.327 and 430.332, respectively. However, the fragment ions in the experimental spectra for both nodes did not fully match the library hits. Therefore, all compounds within cluster A were targeted for isolation.

### Structure Elucidation

Bipolarolide H (**1**) was isolated as a white amorphous solid with the molecular formula C_27_H_41_NO_5_, as deduced from HRESIMS measurements at *m*/*z* 460.3059 [M + H]^+^, suggesting eight degrees of unsaturation. The ^1^H NMR data (Table 1) showed distinct signals for four methyl groups (H_3_-27 (δ_H_ 0.83), H_3_-20 (δ_H_ 0.91), H_3_-19 (δ_H_ 1.21), and H_3_-28 (δ_H_ 1.72)), one oxygenated methylene H_2_-1(δ_H_ 3.45), and an olefinic methine H-24 (δ_H_ 6.61). The ^13^C NMR data (Table 2) confirmed the presence of 27 carbon atoms, including two carbonyl (C-10 (δ_C_ 173.8) and C-26 (δ_C_ 169.2)), two quaternary carbon (C-6 (δ_C_ 81.0), C-17 (δ_C_ 43.5)), three tetra substituted olefinic (C-8 (δ_C_ 163.3), C-9 (δ_C_ 126.9), and C-25 (δ_C_ 128.3)), six methine (C-4 (δ_C_ 65.2), C-7 (δ_C_ 43.1), C-13 (δ_C_ 45.3), C-14 (δ_C_ 46.6), C-21 (δ_C_ 32.7), olefinic C-24 (δ_C_ 104.7)), ten methylene (C-1 (δ_C_ 59.5), C-2 (δ_C_ 44.4), C-5 (δ_C_ 44.3), C-11 (δ_C_ 19.9), C-12 (δ_C_ 25.5), C-15 (δ_C_ 25.8), C-16 (δ_C_ 43.4), C-18 (δ_C_ 42.1), C-22 (δ_C_ 35.7), C-23 (δ_C_ 26.1)), and four methyl carbon atoms (C-19 (δ_C_ 27.2), C-20 (δ_C_ 21.0), C-27 (δ_C_ 17.1), C-28 (δ_C_ 12.3)), as supported by HSQC signals. Four olefinic and two carbonyl carbons atoms accounted for four of the eight indices of hydrogen deficiency, the remaining indices of hydrogen deficiency suggested that **1** has a tetracyclic ring system. The ^1^H–^1^H COSY spectrum of **1** revealed the presence of six independent spin systems (H_2_-1/H_2_-2, H-4/H_2_-5, H-7/H_2_-18, H_2_-11/H_2_-12, H-13/H-14/H_2_-15/H_2_-16, H_3_-27/H-21/H_2_-22/H_2_-23/H-24). The HMBC correlations from H-24 to C-22, C-23, C-26, C-28; and H_3_-28 to C-24, C-25, and C-26, suggested the presence of a C-24/C-25 double bond on the alkyl side chain (H_3_-27/H-21/H_2_-22/H_2_-23/H-24) and the attachment of a methyl (CH_3_-28) and a carboxyl group (C-26) at C-25. This side-chain fragment was attached to the partial structure of ring D (H-13/H-14/H_2_-15/H_2_-16) at C-14 based on HMBC correlations from H_3_-27 to C-14; and H-14 to C-21, C-22, and C-27. Furthermore, HMBC correlations from H-13 to C-12, C-14, C-21; and H_3_-20 to C-16, C-17, C-18, and C-13 established the fusion of a pentane ring (ring D) with ring C extending from C-13 to C-17. The COSY cross peaks H-7/H_2_-18 and H-4/H_2_-5, as well as HMBC correlations from H-7 to C-4, C-8, and C-9; H_3_-19 to C-5, C-6, and C-7 supported a fused A/C ring system. Further analysis of the HMBC spectrum suggested the presence of a five-membered lactam ring (ring B) between rings A and C, which was established by the key HMBC correlations from H_2_-2 to C-4, and C-10; H-4 to C-5, C-8, and C-9; and H_2_-11 to C-9, and C-13. This also confirmed the presence of a C-8/C-9 double bond between rings B and C. The planar structure of **1** is structurally similar to ophiobolin derivatives, mainly bipolarolides^26^ and undobolins,^27^ isolated from *Bipolaris* and *Aspergillus* sp., respectively.

**Table 1:** ^1^H NMR Data (600 MHz, DMSO-*d*_6_) of Compounds **1**–**4**^a^

Position	**1**	**2**	**3**	**4**
1	3.45, t (5.7)	3.45, t (5.7)	3.45, t^b^ (5.7)	3.46, t (6.4)
2′	3.37, dt (13.7, 6.1)	3.37, dt (13.7, 6.1)	3.37, dt^b^ (13.7, 6.1)	3.37, dt (12.7, 6.1)
2″	3.28, dt (13.8, 5.9)	3.28, dt (13.8, 5.9)	3.28, dt^b^ (13.8, 5.9)	3.28, dt (13.8, 5.9)
4	4.51, dd (11.4, 7.3)	4.51 dd (11.4, 7.3)	4.51, dd (12.3, 6.1)	4.51, dd (11.6, 7.0)
5′	2.12, m^b^	2.13, m^b^	2.13, m^b^	2.13, m^b^
5″	1.04, t (11.6)	1.04, t^b^ (11.7)	1.04, t^b^ (11.7)	1.04, t^b^ (11.6)
7	2.55, d (11.6)	2.55, d (11.6)	2.55, m	2.55, d (12.02)
11	1.51, m^b^	1.50, m	1.51, m^b^	1.51, m^b^
12′	2.22, m	2.22, m	2.24, m	2.23, ddt (17.6, 5.5, 3.2)
12″	2.12, m^b^	2.12, m^b^	2.12, m^b^	2.12, m^b^
13	1.78, dt (10.8, 4.9)	1.77, m	1.76, dt (10.5, 4.8)	1.77, dt (10.2, 4.6)
14	2.00, tt (11.1, 7.1)	1.97, m	1.98, m	1.97, tt (11.1, 6.9)
15′	1.50, m^b^	1.49, m^b^	1.47, m^b^	1.48, m^b^
15″	1.37, m	1.33, m^b^	1.34, m	1.34, m
16′	1.50, m^b^	1.49, m^b^	1.50, m^b^	1.49, m^b^
16″	1.24, m^b^	1.23, m^b^	1.22, m^b^	1.23, m^b^
18′	1.64, dd (13.9, 2.1)	1.64, dd	1.64, dd (13.9, 2.1)	1.64, dd (13.8, 2.1)
18″	1.24, m^b^	1.23, m^b^	1.23, m^b^	1.24, m^b^
19	1.21, s	1.21, s	1.21, s	1.21, s
20	0.91, s	0.91, s	0.90, s	0.90, s
21	1.45, m	1.44, m	1.42, m	1.43, m^b^
22′	1.37, m	1.33, m^b^	1.24, m^b^	1.23, m^b^
22″	1.12, m	1.03, m^b^	0.98, m	0.99, m^b^
23′	2.18, m^b^	2.03, m	1.31, m^b^	1.28, m^b^
23″	2.11, m^b^	1.94, m	1.22, m^b^	
24′	6.61, dt (7.7, 3.9)	5.31, td (7.3, 1.4)	1.49, m^b^	1.29, m^b^
24″			1.29, m^b^	0.98, m^b^
25			2.25, m^b^	1.43, m^b^
26′		3.76, s		3.24, dd (10.4, 5.8)
26″		3.76, s		3.16, dd (10.3, 6.5)
27	0.83, d (6.6)	0.82, d (6.5)	0.78, d (6.6)	0.80, d (6.7)
28	1.72, s	1.54, s	1.01, d^b^ (6.7)	0.81, d (6.8)
29				

a δ_H_ in ppm, *J* in Hz.

b Signal overlapped.

**Table 2:** ^13^C NMR Data (150 MHz, DMSO-*d*_6_) of Compounds **1**–**8**^a^

Position	**1**	**2**	**3**	**4**	**5**	**6**	**7**	**8**
1	59.5, CH_2_	59.5, CH_2_	59.4, CH_2_	59.4, CH_2_	59.4, CH_2_	59.4, CH_2_	59.0, CH_2_	59.5, CH_2_
2	44.4, CH_2_	44.4, CH_2_	44.4, CH_2_	44.4, CH_2_	44.4, CH_2_	44.4, CH_2_	42.6, CH_2_	44.4, CH_2_
4	65.2, CH	65.2, CH	65.2, CH	65.3, CH	65.2, CH	65.2, CH	97.2, C	65.2, CH
5	44.3, CH_2_	44.3, CH_2_	44.2, CH_2_	44.3, CH_2_	44.2, CH_2_	44.3, CH_2_	52.8, CH_2_	44.3, CH_2_
6	81.0, C	81.0, C	81.0, C	81.0, C	81.0, C	81.0, C	78.9, C	81.0, C
7	43.1, CH	43.1, CH	43.0, CH	43.1, CH	43.0, CH	43.2, CH	46.7, CH	43.1, CH
8	163.3, C	163.4, C	163.2, C	163.3, C	163.3, C	163.3, C	56.6, CH	163.4, C
9	126.9, C	126.9, C	126.8, C	126.9, C	126.9, C	127.4, C	42.1, CH	126.9, C
10	173.8, C	173.8, C	173.9, C	173.9, C	173.8, C	173.8, C	175.2, C	173.8, C
11	19.9, CH_2_	20.0, CH_2_	19.9, CH_2_	19.9, CH_2_	19.9, CH_2_	20.1, CH_2_	26.0, CH_2_	20.0, CH_2_
12	25.5, CH_2_	25.4, CH_2_	25.6, CH_2_	25.5, CH_2_	25.5, CH_2_	25.1, CH_2_	25.5, CH_2_	25.4, CH_2_
13	45.3, CH	45.2, CH	45.4, CH	45.3, CH	45.3, CH	45.1, CH	42.5, CH	45.2, CH
14	46.6, CH	46.8, CH	46.6, CH	46.9, CH	46.8, CH	48.6, CH	49.9, CH	46.8, CH
15	25.8, CH_2_	25.9, CH_2_	25.6, CH_2_	25.8, CH_2_	25.7, CH_2_	28.6, CH_2_	26.8, CH_2_	25.9, CH_2_
16	43.4, CH_2_	43.5, CH_2_	43.5, CH_2_	43.4, CH_2_	43.4, CH_2_	43.3, CH_2_	45.4, CH_2_	43.5, CH_2_
17	43.5, C	43.5, C	40.1, C	43.5, C	43.5, C	43.6, C	42.9, C	43.5, C
18	42.1, CH_2_	42.1, CH_2_	42.1, CH_2_	42.1, CH_2_	42.1, CH_2_	41.8, CH_2_	44.9, CH_2_	42.1, CH_2_
19	27.2, CH_3_	27.2, CH_3_	27.2, CH_3_	27.2, CH_3_	27.2, CH_3_	27.2, CH_3_	25.5, CH_3_	27.2, CH_3_
20	21.0, CH_3_	21.0, CH_3_	20.9, CH_3_	21.0, CH_3_	20.9, CH_3_	21.3, CH_3_	22.4, CH_3_	21.0, CH_3_
21	32.7, CH	32.6, CH	32.8, CH	32.9, CH	32.9, CH	34.1, CH	31.3, CH	32.5, CH
22	35.7, CH_2_	37.0, CH_2_	36.9, CH_2_	37.3, CH_2_	37.1, CH_2_	137.0, CH	37.7, CH_2_	37.1, CH_2_
23	26.1, CH_2_	25.0, CH_2_	24.8, CH_2_	24.5, CH_2_	24.2, CH_2_	122.2, CH	25.52, CH_2_	25.5, CH_2_
24	140.7, CH	123.4, CH	33.8, CH_2_	33.2, CH_2_	32.9, CH_2_	120.4, CH	124.6, CH	124.5, CH
25	128.3, C	135.4, C	39.3, CH	35.4, CH	32.0, CH	134.8, C	130.6, C	130.7, C
26	169.2, C	66.4, CH_2_	165.5, C	66.3, CH_2_	67.9, CH_2_	26.2, CH_3_	25.5, CH_3_	25.5, CH_3_
27	17.1, CH_3_	17.3, CH_3_	17.2, CH_3_	17.3, CH_3_	17.3, CH_3_	20.6, CH_3_	18.2, CH_3_	17.3, CH_3_
28	12.3, CH_3_	13.5, CH_3_	17.2, CH_3_	16.8, CH_3_	16.5, CH_3_	18.0, CH_3_	17.5, CH_3_	17.5, CH_3_
29					162.2, CH			

a δ_C_ in ppm.

The relative configuration of **1** was determined using ROESY. In the ROESY spectrum, the correlations of H-5″/H_3_-19, H-5″/H-7, H_3_-19/H-7, H-7/H-18′, H-18′/H_3_-20, H-7/H_3_-20, and H_3_-20/H-16′ indicated that these protons were in a cofacial arrangement and were assigned as β-oriented, whereas H-5′ was established to be α-oriented. The ROESY correlation of H-4/H-5′(α), along with the absence of correlations H-4/H-5″(β), H-4/H_3_-19, H-4/H-7, and H-4/H-13, revealed that H-4 should be α-oriented. Similarly, the ROESY correlations of H-18″(α)/H-13, H-16″(α)/H-13, H-15′(α)/H-13, 15′(α)/H-14, and H-13/H-14, but the absence of cross-peaks H_3_-20/H-13, H-18′(β)/H-13, H-16′(β)/H-13, and 15″(β)/H-13 suggested that H-13 and H-14 were α-oriented (Figure 4). Furthermore, the relative configuration of C-21 was established by examining the Newman projection of C-14–C-21 (Figure 5B).^27−29^ The observed ROESY correlations of H-21/H-12″, H_3_-27/H-15′/″, H_3_-27/H-14, H-22″/H-12″, and H-22′/″-H-14 indicated that the configuration of C-21 was 21S* as shown in Figure 5B. Previous biosynthetic studies have also confirmed that during the cyclization of all ophiobolins, the process tends to favor the 21*S* configuration of the side chain.^26,30^ Therefore, the relative configurations of the stereocenters in the ring system were determined as 4R*, 6R*, 7S*, 13S*, 14R*, 17R*, 21S*. In addition, the observed ROESY correlation of H_2_-23/H_3_-28 and the absence of H-24/H_3_-28 support the *E*-configuration of the Δ^24^-double bond.

To determine its absolute configuration, the ECD spectra of 1a and *ent*-1a were calculated. The experimental ECD curve was in close agreement with those calculated for 1a, suggesting a configuration of 4*R*, 6*R*, 7*S*, 13*S*, 14*R*, 17*R*, 21*S* (Figure 5C). To further confirm the assignment of C-4 as 4*R*, the 4*S*-epimer of 1a (1b) and its enantiomer (*ent*-1b) were subjected to ECD calculations (Figure S105). The geometry-optimized structures of four stereoisomers (1a, *ent*-1a, 1b, and *ent*-1b) were examined by focusing on the measured distances between key protons: H-4/H-7, H-4/H_3_-19, and H-4/H-13. The distances H-4/H-7 and H-4/H_3_-19 varied less among the stereoisomers, making them less useful for differentiation. However, the distance H-4/H-13 was notably shorter in 1a (<4 Å) compared to 1b and *ent*-1b (>5 Å) (Figure 5A). The stereoisomer 1a had a distance between H-4 and H-13 that agreed with the observed ROESY correlation, establishing the absolute configuration of **1** as 4*R*, 6*R*, 7*S*, 13*S*, 14*R*, 17*R, 21S*. To further confirm the assignment of C-21 as 21*S*, the ECD spectra of 1c (the 21*R* epimer of 1a) and its enantiomer (*ent*-1c) were also analyzed (Supporting Information). A comparison of the calculated and experimental ECD curves further corroborated the assignment of C-21 as 21*S* (Figure S106).

**Figure 3:**
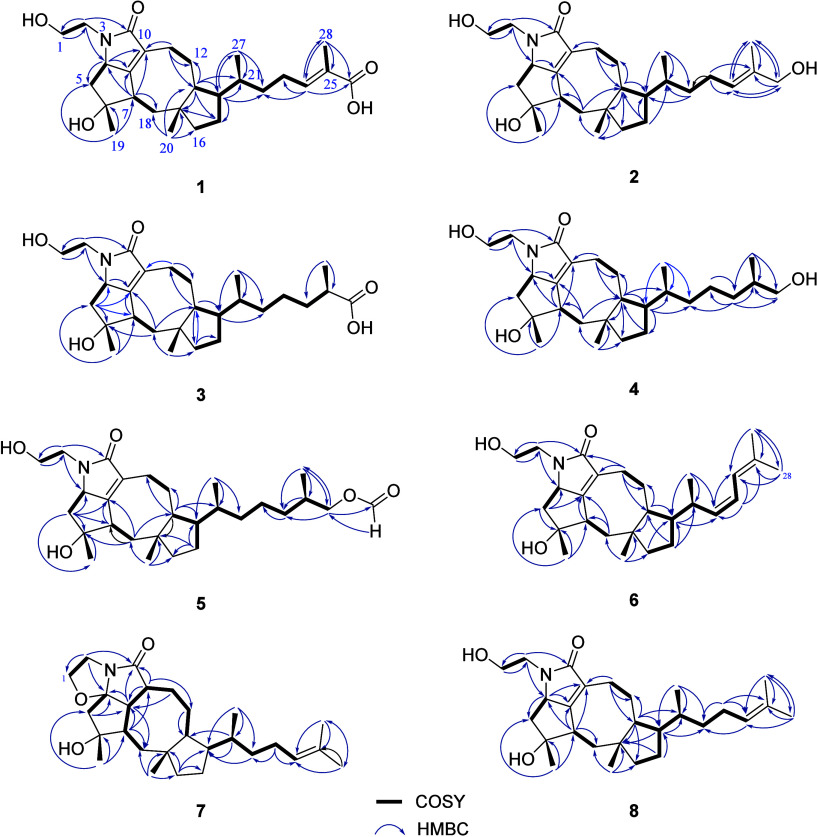
Key HMBC and COSY correlations of compounds **1**–**8**.

Bipolarolide I (**2**) had a molecular formula of C_27_H_43_NO_4_, based on HRESIMS analysis at *m*/*z* 446.3265 [M + H]^+^. ^1^H and ^13^C NMR showed close similarities to **1**, except that the carboxylic group (δ_C_ 128.3) in **1** was replaced by a hydroxylated methylene (δ_C_ 66.4) in **2**. This was inferred from the HMBC correlations (Figure 3) from H-24 (δ_C_ 5.31) to C-26 (δ_C_ 66.4), and C-28 (δ_C_ 13.5); H_3_-28 to C-24 (δ_C_ 123.4), C-25 (δ_C_ 135.4), and C-26; and H_2_-26 (δ_H_ 3.76) to C-24, C-25, and C-28. The ROESY correlation observed for this structure was also aligned with the data recorded for **1**, suggesting the same relative configurations. Comparison of the experimental ECD curve with that of **1** (Figures 5C, S107), which was similar to the calculated ECD curve of 1a, confirmed its absolute configuration to be 4*R*, 6*R*, 7*S*, 13*S*, 14*R*, 17*R*, 21*S.*

**Figure 4:**
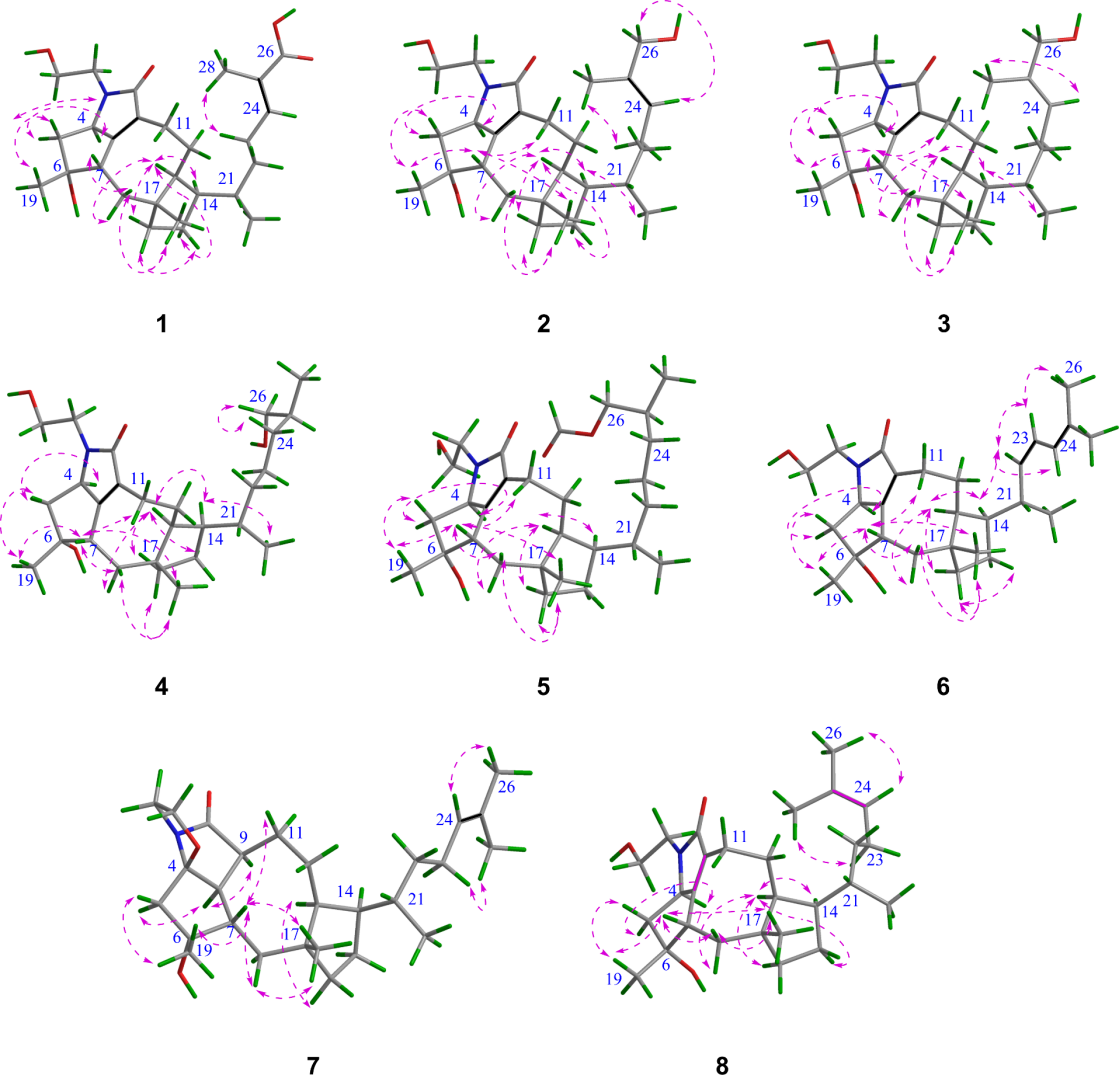
ROESY correlations of compounds **1**–**8**.

**Figure 5:**
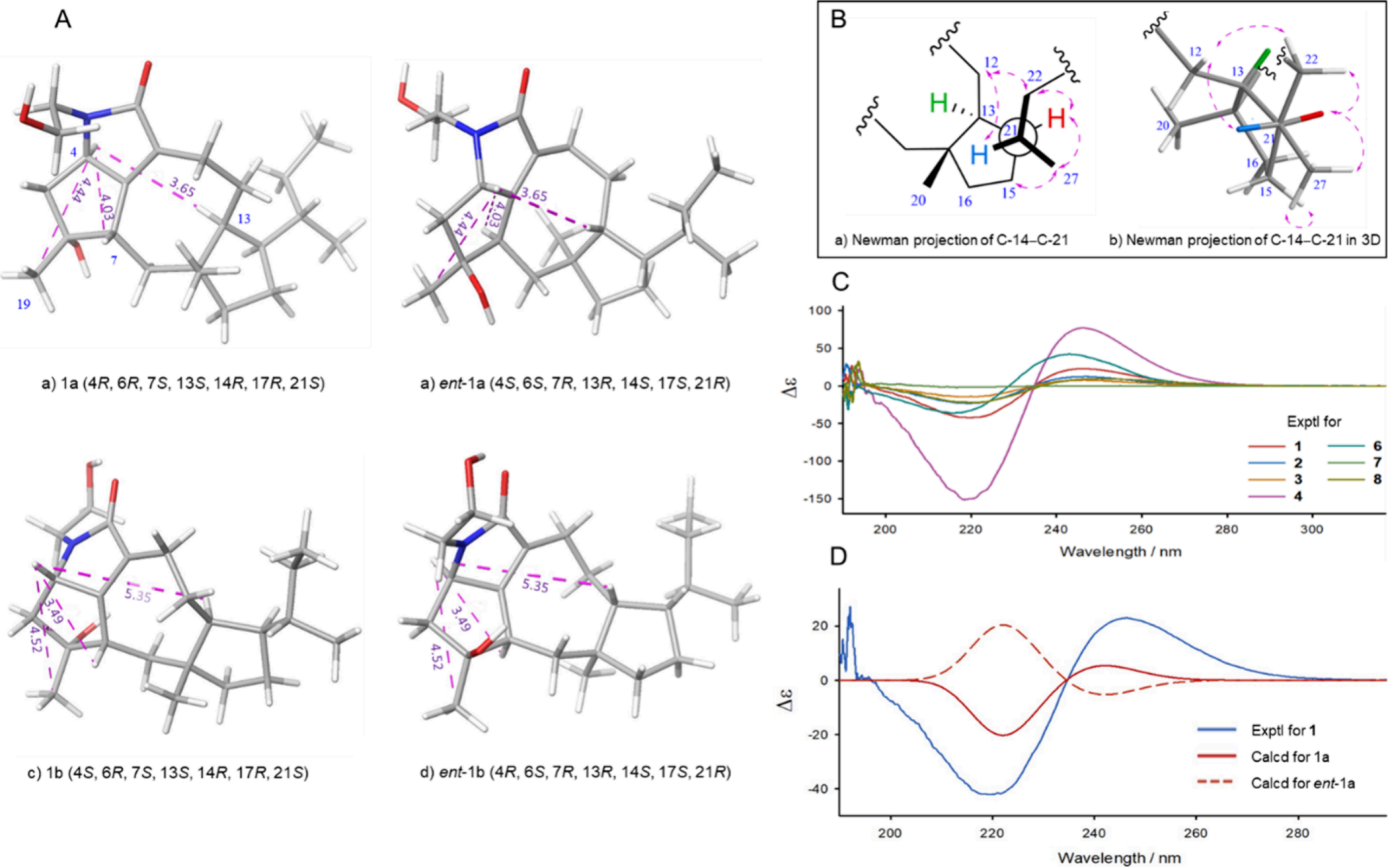
(A) Geometry-optimized structures of four stereoisomers of **1** showing the distances H-4/H-7, H-4/H_3_-19, and H-4/H-13. (B) Newman projection of C-14–C-21 showing key ROESY correlations in 2D and 3D (geometry-optimized) structures of **1a** with 21S configuration. (C) Experimental ECD spectra of compounds **1**–**8**. (D) Calculated (for **1a** and *ent*-**1a**) and experimental ECD spectra of **1**. The energies of the calculated spectra were scaled by a scaling factor of 0.82.

Bipolarolide J (**3**) was purified as a white amorphous solid, and its HRESIMS (*m*/*z* 462.3215 [M + H]^+^) analysis indicated a molecular formula of C_27_H_43_NO_5_, implying seven degrees of unsaturation. A comparison with the elemental composition of **1** revealed the addition of two proton atoms to **3**. In addition, the difference of one degree of unsaturation between **1** and **3** suggested a reduction of one of the double bonds in **1**. The 1D NMR spectra of **3** matched well with those of **1** and revealed the same ophiobolin-based moiety, except for the Δ^24^-double bond in **1**. The degree of unsaturation of **3** and the shielded carbons C-24 (δ_C_ 33.7) and C-25 (δ_C_ 39.2) indicated a reduction in the Δ^24^-double bond compared to **1**. This was further confirmed by HMBC correlations from H_3_-28 (δ_H_ 1.01) to C-24 and C-25, as well as ^1^H–^1^H COSY correlations of H_2_-22/H_2_-23, H_2_-23/H_2_-24, and H_2_-24/H-25. The relative configuration of **3** compared to that of **1** and **2** was the same with the addition of a chiral center at C-25 (Figure 4). The configuration of C-25 carbon is not described in this paper, as its determination necessitates more extensive experiments and simulations. The configuration of other stereocenters was assigned as 4*R*, 6*R*, 7*S*, 13*S*, 14*R*, 17*R*, 21*S* as the experimental ECD curve showed good agreement with the curves of **1** and 1a (Figures 5C, S107).

Bipolarolide K (**4**) was isolated as a white amorphous solid. Its molecular formula was deduced to be C_27_H_45_NO_4_ with only six degrees of unsaturation by HRESIMS at *m*/*z* 448.3422 [M + H]^+^, differing from **2** by two additional hydrogen atoms. This was confirmed through analysis of NMR data (Tables 1–3), which indicated that the Δ^24^-double bond in **2** was reduced in **4**. The reduction was evidenced by the lack of olefinic proton and carbon signals at δ_H_ 5.13/ δ_C_ 123.4 (CH-24) and δ_C_ 135.4 (C-25) in the spectra of **4**, which were present in **2**. Instead, the 1D NMR spectra of **4** displayed signals at δ_H_ 0.9, 1.29/ δ_C_ 33.2 (CH_2_-24), and δ_H_1.44/ δ_C_ 35.4 (CH-25), indicating the reduction of the double bond. The observed COSY correlations of H_2_-23/H_2_-24/H-25/(H_2_-26 and H_3_-28), as well as the HMBC correlations from H_2_-24 to C-22, C-23, C-25, and C-26; H_3_-28 to C-24, C-25, and C-26; and H_2_-26 to C-25, confirmed this deduction. The relative configuration of **4** was similar to that of **3** based on their similar ROESY. Additionally, the ECD spectrum of **4** (Figures 5C, S107) was also similar to that of **1**, and **3**, suggesting that they shared the same absolute configuration.

**Table 3:** ^1^H NMR Data (600 MHz, DMSO-*d*_6_) of Compounds **1**–**8**^a^

Position	**5**	**6**	**7**	**8**
1	3.46, t (6.1)	3.45, t (6.3)	3.48, m	3.45, t (5.8)
2′	3.36, m^b^	3.35, m^b^	3.34, m^b^	3.37, dt (12.4, 6.2)
2″	3.28, dd (13.4, 6.4)^b^	3.28, m^b^	3.02, dt (13.5, 6.8)	3.28, dt (13.8, 6.0)
4	4.51, m	4.49, dd (12.7, 6.5)		4.51, dd (12.0, 6.5)
5′	2.13, m^b^	2.12, dd (11.2, 6.5)	2.01, m^b^	2.13, dd^b^
5″	1.04, t (11.6)	1.01, t (11.6)	1.73, m^b^	1.04, m^b^
7	2.55, m	2.53, m^b^	1.52, t (11.1)	2.54, d (17.5)
8			2.52, m^b^	
9			2.36, m	
11′	1.51, m^b^	1.61, m^b^	1.36, m^b^	1.51, td (11.0, 5.4)^b^
11″		1.42, m		
12′	2.23, m	2.04, m	2.00, m^b^	2.21, m
12″	2.12, m^b^			2.13, dd (11.2, 6.5)^b^
13	1.77, dt (10.5, 4.8)	1.75, dt (10.7, 5.1)	1.73, m^b^	1.77, m
14	1.98, m	1.93, qd^b^	1.74, m^b^	1.96, m^b^
15′	1.48, m^b^	1.62, m^b^	1.42, m^b^	1.48, m^b^
15″	1.35, m^b^	1.37, m	1.27, m^b^	1.34, m
16′	1.50, m^b^	1.51, m^b^	1.38, m^b^	1.48, m^b^
16″	1.23, m^b^	1.24, m^b^	1.30, m^b^	1.23, m^b^
18′	1.64, d (13.5)	1.60, m^b^	1.63, d (12.4)^b^	1.64, d (9.1)
18″	1.23, m^b^	1.24, m^b^	1.24, m^b^	1.24, m^b^
19	1.21, s	1.21, s	1.11, s	1.21, s
20	0.90, s	0.93, s	0.87, s	0.90, s
21	1.43, m	2.53, m^b^	1.40, m	1.43, m
22′	1.22, m^b^	5.15, t (8.5)	1.40, m^b^	1.27, m
22″	0.99, m		0.97, m	1.00, m^b^
23′	1.29, m^b^	6.04, d^b^ (8.5)	1.89, m^b^	1.99, m^b^
23″				1.91, m^b^
24′	1.30, m^b^	6.05, s^b^	5.11, t (7.3)	5.09, td (6.5, 1.5)
24″	1.12, m^b^			
25	1.74, m^b^			
26′	3.98, dd (10.7, 5.8)	1.78, s	1.64, s	1.64, s
26″	3.90, dd (10.7, 6.5			
27	0.80, d (6.6)	0.89, d (6.7)	0.83, d (6.6)	0.81, d (6.6)
28	0.88, d (6.7)	1.71, s	1.57, s	1.56, s
29	8.24, s			

a δ_H_ in ppm, *J* in Hz.

b Signal overlapped.

Bipolarolide L (**5**) had a molecular formula of C_28_H_45_NO_5_ based on HRESIMS data at *m*/*z* 476.3369 [M + H]^+^. This suggested the addition of one carbon and one oxygen unit compared to the elemental composition of **4** (C_27_H_45_NO_4_). The structure of **5** was similar to **4** except for the presence of (−OCHO) instead of the hydroxyl group (−OH) at C-26. The presence of a formic acid ester group (−OCHO) in **5** was confirmed by the HMBC correlations from H_2_-26 (δ_H_ 3.98, 3.90) to C-29 (δ_C_ 162.2); and H-29 (δ_H_ 8.24) to C-26 (δ_C_ 67.9). Additionally, the attachment of the formic acid ester group at C-26 was indicated by deshielded shifts of H_2_-26, C-25 (δ_H_ 1.74), and H_3_-28 (δ_H_ 0.88) protons in the ^1^H spectrum, compared to **4**. The ROESY spectrum analysis illustrated that the relative configuration of **5** was 4R*, 6R*, 7S*, 13S*, 14R*, 17R*, 21S*. This configuration was identical to that of **1** with one more chiral carbon (C-25) as in **3** (Figure 4). The experimental ECD spectrum was not measured. Nevertheless, it is expected to have the same absolute configuration as other compounds, since similar ROESY correlations were observed in compound **1**–**8**, except for **7**, and all these compounds had similar ECD spectra.

Bipolarolide M (**6**) was purified as a light brown amorphous solid. HRESIMS displayed a signal for [M + H]^+^ at *m*/*z* 428.3156 with a molecular formula of C_27_H_41_NO_3_, suggesting eight degrees of unsaturation. Comparison of the 1D NMR spectra of **6** with those recorded for **1** revealed that the chemical shifts of CH_2_–22 (δ_C_ 35.7/δ_H_ 1.37, 1.12) and CH_2_–23 (δ_C_ 26.1/δ_H_ 2.18, 2.11) in **1** were deshielded to δ_C_ 137.0/δ_H_ 5.15 (CH-22) and δ_C_ 122.2/δ_H_ 6.04 (CH-23) in **6**. This indicated a presence of Δ^22^-double bond in **6**, as supported by key HMBC correlations from H-14 (δ_H_ 1.93) and H_3_-27 (δ_H_ 0.89) to C-22; and H-23 to C-22, C-24 (δ_C_ 120.4), and C-25 (δ_H_ 134.8), as confirmed by the ^1^H–^1^H COSY correlation of H-21/H-22/H-23/H-24. Moreover, the substitution of the carboxylic group at C-25 in **1** with a methyl group (CH_3_-26) in **6** was further corroborated by the key HMBC correlations observed from H_3_-26 to C-24, C-25, and C-28 (δ_C_ 18.0). The ROESY correlation of **6** was similar to that of **1**, suggesting that they have the same relative configuration at C-4, C-6, C-7, C-13, C-17, and C-21. However, the cross-peaks H-14/H-22, H-22/H-23|24, H-21/H-23|24, and H-23/H-28 could not unambiguously determine the configuration of the Δ^22^-double bond at the resolution of the acquired ROESY because H-23 and H-24 almost perfectly overlapped (∼1–2 Hz separation). Instead, the ^1^H coupling patterns of H-24, H-23, and H-22 were examined. H-24 appeared as a singlet, presumably the potential splitting to the overlapping H-23 was auto-decoupled as the separation between the resonances was much smaller than the coupling constant between them, while H-23 appeared as 8.5 Hz doublet (split by H-22), and H-22 appeared as 8.5 Hz triplet (split by H-23 and H-21). Thus, the coupling constant (^3^*J*_H-22/H-23_) was assigned to 8.5 Hz, and it was concluded that **6** has a 22*Z*-configuration, based on the expected trans coupling (11–18 Hz) vis-à-vis cis coupling (6–15 Hz). The key ROESY correlations are shown in Figure 4. The absolute configuration of **6** was suggested to be the same as that of **1** as shown by comparable ECD curves (Figures 5C, S107).

Bipolarolide N (**7**) was obtained as a white amorphous solid, and its molecular formula was determined to be C_27_H_43_NO_3_ at *m*/*z* 430.3315 [M + H]^+^ using HRESIMS, with an index of hydrogen deficiency of seven. This indicated the reduction of one of the double bonds in **6** (C_27_H_41_NO_3_) to form **7**. In addition, there were significant differences in both ^1^H and ^13^C chemical shifts. The chemical shifts of C-8 (δ_C_ 163.3), C-9 (δ_C_ 127.4), C-22 (δ_C_ 137.0/δ_H_ 5.15), and C-23 (δ_C_ 122.2/δ_H_ 6.04) in **6** were shielded to δ_C_ 56.6/δ_H_ 2.51, δ_C_ 42.1/δ_H_ 2.36, δ_C_ 37.7/δ_H_ 1.40, 0.97, and δ_C_ 25.5/ δ_H_ 1.89 respectively in **7**. These spectroscopic features suggested that the Δ^8^- and Δ^22^-double bonds in **6** were reduced in **7**. This was supported by H-7/H-8/H-9/H_2_-11/H_2_-12 and H_3_-20/H-21/H_2_-22/H_2_-23/H-24 COSY correlations. Furthermore, 1D NMR revealed that the chemical shifts of C-4 (δ_C_ 65.2/δ_H_ 4.49) and C-5 (δ_C_ 44.3) in **6** were deshielded to δ_C_ 97.2 with the loss of the methine proton and δ_C_ 52.8, respectively in **7**, suggesting oxygen connectivity at C-4. HRMS analysis suggested the formation of an additional ring in the structure, which was consistent with the calculated elemental composition and accounted for seven degrees of unsaturation. The hydroxy group (−OH) at C-1 was connected to C-4 to form a five-membered ring (oxazolidine) because it was the most realistic connection, considering the structural similarity to compounds **1**–**6** and **8**. This was supported by the **∼**32 ppm deshielding of C-4 and **∼**8.5 ppm deshielding of C-5, reflecting the new oxygen bond. One of the H_2_-2 protons was shielded by 0.3 ppm compared to **1**–**6**, and **8**, suggesting that the chain was more rotationally restricted relative to the shielding effect of the nearby carbonyl. The coupling pattern of H_2_-1 changed from a broadened triplet to a complex pattern, indicating that it was rotationally restricted (possibly in multiple conformations). The assignment of the C-4 chemical shift and the formation of the oxazolidine ring were also supported by HMBC correlations from H_2_-2, H_2_-5, and H-8 to C-4, although correlation from H_2_-1 to C-4 was not well detected. The ROESY analysis illustrated that the relative configuration of **7** was similar to that of **1**, except at C-4 (a quaternary carbon), C-8, and C-9. The lack of a ROESY correlation of H-7/H-8, along with the observed correlation of H-8/H-9, suggested that H-8 and H-9 were α-oriented. This indicated a relative configuration of **7** as 6R*, 7S*, 8R*, 9S*, 13S*, 14R*, 17R*, 21S*. ECD analysis did not provide enough evidence for the absolute configuration of **7** due to a lack of chromophores in the molecule. The absence of the Δ^8^-double bond, in contrast to other compounds, resulted in an almost flat ECD curve (Figures 5C, S107).

Bipolarolide O (**8**) was isolated as a white amorphous solid and assigned a molecular formula of C_27_H_43_NO_4_ by analysis of HRESIMS data at *m*/*z* 430.3315 [M + H]^+^, with seven degrees of unsaturation. The ^1^H and ^13^C NMR spectra of **8** showed resonances similar to those of **6**, and the only significant difference was the reduction of the Δ^22^-double bond. This was ascertained by the deshielded chemical shift of signals of C-22 (δ_C_ 137.0/ δ_H_ 5.15) and C-23 (δ_C_ 122.2/ δ_H_ 6.04) in **6** to C-22 (δ_C_ 37.1/ δ_H_ 1.27, 1.00) and C-23 (δ_C_ 25.5/ δ_H_ 1.99, 1.91) in **8**, as well as the HMBC correlations from H-14, H-24, and H_3_-27 to C-22; and H_2_-23 to C-22, C-24, C-25. The structure was further corroborated by the ^1^H–^1^H COSY correlations of H-21/H_2_-22/H_3_-23/H-24. The relative configuration of **8** was the same as that of **1** and other compounds, based on similar ROESY correlations (Figure 4). Furthermore, the absolute configuration of **8** was assigned as 4*R*, 6*R*, 7*S*, 13*S*, 14*R*, 17*R*, 21*S* due to its similar ECD spectrum to that of **1** (Figures 5C, S107).

### Antibacterial Activity

**Figure 6:**
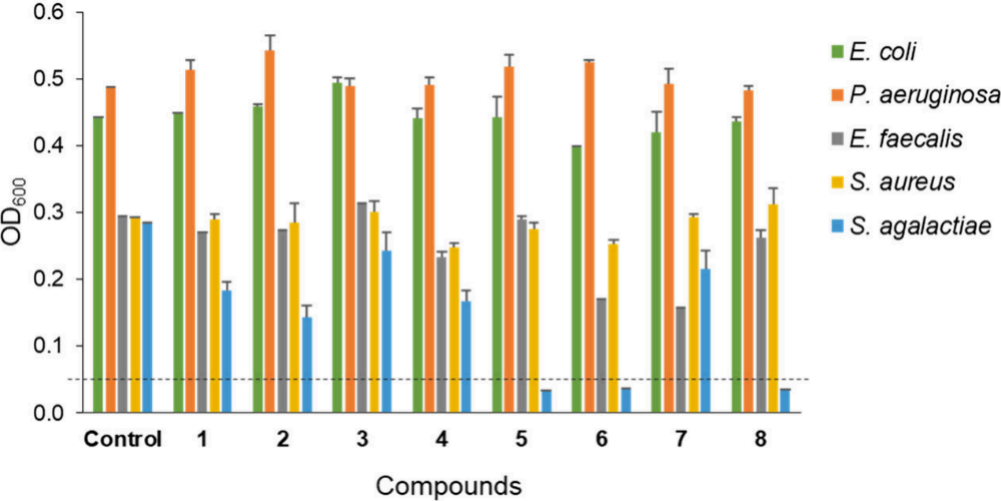
Growth inhibition assay of **1**–**8** tested at 100 μM concentration toward five pathogenic bacterial strains. The data are mean ± SD of duplicate assays (for two independent experiments).

The antibacterial activity of the chromatography fractions and isolated pure compounds **1**–**8** was evaluated against three Gram-positive (*Enterococcus faecalis*, *Staphylococcus aureus*, and *Streptococcus agalactiae*) and two Gram-negative bacteria (*Escherichia coli*, and *Pseudomonas aeruginosa*). The fractions were tested at a concentration of 100 μg/mL. In the initial screening, fractions 5 and 6 showed significant inhibition of the growth of *S. agalactiae* and *E. faecalis* (Figure S108). Compounds **1**–**8** isolated from fraction 5 were tested against the same five pathogenic bacterial strains at a concentration of 100 μM. Compounds **5**, **6**, and **8** showed antibacterial activity against *S. agalactiae*, while none of them were active against *E. faecalis*, *S. aureus*, *E. coli*, and *P. aeruginosa* as shown in Figure 6. The antimicrobial efficacies of **5**, **6**, and **8** were further assessed to determine their minimum inhibitory concentrations (MIC) against *S. agalactiae* with a dilution series of concentrations ranging from 125 to 12.5 μM. The MIC values of the three active compounds against *S. agalactiae* were calculated to be 86, 66, and 64 μM for **5**, **6**, and **8**, respectively, using the concentration-effect curve (Figure S109, Table 4). The presence of two methyl substituents at C-25 and a hydroxylated ethylene substituent at N-3 seems important to the antibacterial effect since compounds **1**–**4** and **7** were inactive against *S. agalactiae*. The data also suggests that the substitution of one of the methyl groups at C-25 carbon of **6** or **8** by carboxylic acid or hydroxylated methylene groups affects the compound’s ability to inhibit the growth of *S. agalactiae*. The exception is **5**, where the formyl group is present at C-26. In addition, the presence of additional oxazolidine rings in **7** formed by intramolecular cyclization revealed that the N-substituted ethylene hydroxide moiety is also important for antibacterial activity against *S. agalactiae*.

**Table 4:** MICs of **5**, **6**, and **8** against *S. agalactiae*

Compound	MIC (μM)
**5**	86
**6**	66
**8**	64
Gentamycin	3

## Experimental Section

### General Experimental Sections

UV and ECD spectra were measured using a JASCO J–815 spectropolarimeter. NMR spectra were recorded using a Bruker Avance III HD spectrometer (operating at 600 MHz for ^1^H and 150 MHz for ^13^C) equipped with a cryogenically enhanced TCI probe at 25 °C. The NMR spectra were recorded in DMSO-*d*_6_, and the chemical shifts were referenced relative to the residual solvent signal. Chemical shifts (δ) were expressed in ppm, and coupling constants were given in Hz.

High-resolution electrospray ionization mass spectrometry (HRESIMS) was performed on an Acquity I-class UPLC (Waters Corporation) coupled with a Vion IMS QTof mass spectrometer (Waters Corporation) running in positive ion mode. A high-resolution LC-MS/MS experiment for metabolomic profiling using the GNPS platform was conducted on a Vanquish Horizon UPLC coupled to an Orbitrap ID-X Mass Spectrometer with an ESI source (Thermo Fisher Scientific Inc.). Diaion HP-20 resin (Supelco, 13607) and Diaion HP-20SS resin (Supelco, 13615) were used for the extraction and flash column chromatography, respectively. MPLC was conducted on a flash chromatography system (Biotage SP4 system) with a Biotage SNAP 10 g cartridge column (self-packed with Diaion HP-20ss resin). Mass-guided isolation of compounds was performed on a preparative HPLC-MS (Waters Auto Purification LCMS system with 2996 PDA and 3100 Mass Detector) with a Sunfire C18 OBD prep column (250 × 10 mm, 5 μm) in the first round of purification and XSelect CSH Phenyl-Hexyl Prep column (250 × 10 mm, 5 μm) or XSelect CSH Fluoro-Phenyl Prep column (250 × 10 mm, 5 μm) in the second round of purification. Solvents A (0.1% formic acid in water) and B (0.1% formic acid in acetonitrile) were used as mobile phases during both the first and second rounds of purification. Milli-Q water used was produced by an in-house Milli-Q system.

### Collection and Phylogenetic Analysis of the Fungus

The *Pleosporales* isolate 009aD3.2 was isolated from a piece of driftwood log of deciduous wood collected from the intertidal zone at Taterneset (69°16′25.8″ N 19°56′25.8″ E), Storfjord municipality, Troms, Norway, in May 2010.^25^ The fungus was grown in a D2MA medium until the plate was almost fully grown and pieces of agar with fungal mycelium were used for DNA extraction and sequencing as described in Rämä et al. (2014).^25^ The fungus was preserved in a 20% sterile filtered glycerol solution at −80 °C until further use. The fungus is described here as a new species *U. storfjordensis* (Mycobank number MB854047) based on the results of phylogenetic analyses and morphological investigations (Figures S1–3). The holotype of the fungus TROM-F-910502 is a freeze-dried culture on malt extract agar medium (isotype TROM-F-26886 air-dried culture on malt extract agar) and stored at Tromsø university museum fungarium. The ex-holotype culture CBS 152423 is preserved at the Westerdijk Fungal Biodiversity Institute in The Netherlands and the Norwegian marine biobank Marbank (M10F0001). For phylogenetic analysis, ITS and 28S sequences with GenBank accession numbers PP820634, and PP820633 respectively were subjected to BLAST searches against the sequences available in GenBank.^31^ Sequences with high similarity BLAST matches and out group sequences were selected and aligned using MAFFT^32,33^ implemented in Geneious Prime (available at www.geneious.com), followed by manual adjustments. This resulted in a 441 bp long ITS and an 817 bp long 28S alignment that was used to build the phylogenetic trees with RAxML version 8.2.11 in Geneious Prime.^34^*Monoseptella rosea* was used as an out group in both analyses. A general time-reversible model was used as a substitution matrix, and the support values were obtained from a 1000 generation bootstrap analysis.

### Fermentation and Extraction

The fermentation of the isolate 009aD3.2 *Pleosporales* sp. was performed on D2MA (each flask contained 1 g of malt extract (Sigma-Aldrich), 10 g of sea salt (Instant Ocean Sea Salt), and 250 mL of Milli-Q water) in 40 × 500 mL Erlenmeyer flasks incubated for 67 days at 16 °C. The fermented material was extracted twice using Diaion HP-20 resin and methanol to yield 6.074 g of dry extract.

### Mass Spectrometry Data Processing

The data generated by the software AcquireX in the. RAW format was converted to. mzXML format using MSConvert.^35^ Subsequently, the data was processed in MZmine v3.7.2.^36,37^ For mass detection, a background noise filter of 2.0E4 and 1.0E3 was applied for MS^1^ and MS^2^ levels, respectively. The ADAP chromatogram builder algorithm was employed with a minimum group intensity threshold of 3.0E4, a minimum group size of scans of 4, and a *m*/*z* tolerance of 15.0 ppm. The chromatograms were deconvoluted using the ADAP algorithm with a signal-to-noise (S/N) window as an estimator, and a threshold of 80. Isotopes were detected using the isotope peak grouper with an *m*/*z* tolerance of 3.0 ppm and a retention time (RT) tolerance of 0.01 min (absolute), selecting the most intense isotope with a maximum charge of 1. Peak alignment was performed with the join aligner algorithm using an *m*/*z* tolerance of 8.0 ppm and an absolute RT tolerance of 0.05 min, with a weight for *m*/*z* of 70 and a weight for RT of 30. The resulting peak list was filtered to select features associated with MS^2^ scans eluted between 0.6 and 8 min and de-replicated using an in-house database of MAAs with an *m*/*z* tolerance of 8.0 ppm. The final output was a peak list comprising 2973 individual features exported to a. mgf file and. csv quantitation table for submission to the GNPS platform, without applying gap-filling in this assay.

### Feature-Based Molecular Networking

The .mgf file obtained with Mzmine was used to generate a network using the feature-based molecular network workflow on the GNPS web platform (https://gnps.ucsd.edu). Molecular networks were generated using the following parameters: precursor ion and fragment ion tolerance set to 0.02, minimum pairs cosine score 0.6, minimum matched fragment ions 4, and minimum cluster size 2. The data was filtered by excluding all MS^2^ fragment ions within a range of ±17 Da from the precursor *m*/*z*. MS^2^ spectra were window-filtered by choosing only the top six fragment ions in the ±50 Da window throughout the spectrum. MS data in the network were searched for in the GNPS spectral libraries with a score threshold of 0.6 and at least four matched peaks. Molecular networks (MN) were visualized using Cytoscape 3.10.0.^38^

### Isolation and Purification

The extract was subjected to flash column chromatography with a maximum loading of 2 g of extract in each round. Fractionation was performed in a self-packed column cartridge (Diaion HP-20SS) with a step gradient elution of water–methanol (95:5 to 0:100, v/v) followed by elution with methanol–acetone (1:1 to 0:1, v/v) to afford eight fractions (Fr.1–8).

Fr. Five was subjected to preparative HPLC-MS on a semipreparative reversed-phase column with a gradient of elution of solvents A (0.1% formic acid in water) and B (0.1% formic acid in acetonitrile). The following optimized gradient was used at a flow rate of 6 mL/min to obtain compounds **1**–**8**: 0–2 min (15% B); 2–20 min (15–100% B); 20–24 min (100% B); 24.1–26 min (15% B).

Compounds **1**, **2**, and **3** were further purified by semipreparative HPLC-MS using a semipreparative HPLC equipped with a phenyl hexyl column with gradient elution of solvent A and B: 0–5 min (40% B); 5–12.5 min (40–68% B); 12.5–15 min (100% B); 15.1–18 min (40% B). Further separation of **4** over the XSelect fluoro-phenyl column with elution was as below; 0–2 min (52% B); 2–7 min (52–71% B); 7.1–8 min (100% B); 8.1–9 min (52% B) provided pure **4** and **5**. In addition, **6** and **7** were subjected to a fluoro-phenyl column eluted with the same gradient [solvent A: B (48:52 to 29:71% v/v)] in the second round of purification. Compound **8** was purified on the same fluoro-phenyl column and eluted with solvents A–B (48:52 to 13:87, v/v).

#### Bipolarolide H (**1**)

white solid; UV (c 0.06, MeOH) λ_max_ (log ε) 218 (3.62) nm; ECD (c 0.06, MeOH) λ_max_ (Δε) 217 (−42.25), 246 (+22.94) nm; 1D and 2D NMR (600 MHz, DMSO-*d*_6_), see Tables 1 and 2; HRESI(+)- MS, *m*/*z* 460.3059 [M + H]^+^ (calcd for C_27_H_42_NO_5_^+^, 460.3063); Observed CCS, 207.77 Å^2^.

#### Bipolarolide I (**2**)

white solid; UV (c 0.06, MeOH) λ_max_ (log ε) 217 (3.15) nm; ECD (c 0.06, MeOH) λ_max_ (Δε) 219 (−23.17), 246 (+12.04) nm; 1D and 2D NMR (600 MHz, DMSO-*d*_6_), see Tables 1 and 2; HRESI(+)- MS, *m*/*z* 446.3265 [M + H]^+^ (calcd for C_27_H_44_NO_4_^+^, 446.3270); Observed CCS, 206.39 Å^2^.

#### Bipolarolide J (**3**)

white solid; UV (c 0.06, MeOH) λ_max_ (log ε) 218 (2.92) nm; ECD (c 0.06, MeOH) λ_max_ (Δε) 218 (−14.77), 246 (+7.27) nm; 1D and 2D NMR (600 MHz, DMSO-*d*_6_), see Tables 1 and 2; HRESI(+)- MS, *m*/*z* 462.3215 [M + H]^+^ (calcd for C_27_H_44_NO_5_^+^, 462.3219); Observed CCS, 209.25 Å^2^.

#### Bipolarolide K (**4**)

white solid; UV (c 0.06, MeOH) λ_max_ (log ε) 218 (3.88) nm; ECD (c 0.06, MeOH) λ_max_ (Δε) 219 (−150.7), 246 (+76.36) nm; 1D and 2D NMR (600 MHz, DMSO-*d*_6_)), see Tables 1 and 2; HRESI(+)- MS, *m*/*z* 448.3422 [M + H]^+^ (calcd for C_27_H_46_NO_4_^+^, 448.3427). Observed CCS, 208.09 Å^2^.

#### Bipolarolide L (**5**)

white solid; 1D and 2D NMR (600 MHz, DMSO-*d*_6_)), see Tables 2 and 3; HRESI(+)- MS, *m*/*z* 476.3369 [M + H]^+^ (calcd for C_28_H_46_NO_5_^+^, 476.3376). Observed CCS, 215.71 Å^2^.

#### Bipolarolide M (**6**)

light brown solid; UV (c 0.06, MeOH) λ_max_ (log ε) 238 (3.67) nm; ECD (c 0.06, MeOH) λ_max_ (Δε) 215 (−36.8), 242 (+41.89) nm; 1D and 2D NMR (600 MHz, DMSO-*d*_6_), see Tables 2 and 3; HRESI(+)- MS, *m*/*z* 428.3156 [M + H]^+^ (calcd for C_27_H_42_NO_3_^+^, 428.3165); Observed CCS, 216.59 Å^2^.

#### Bipolarolide N (**7**)

white solid; UV (c 0.06, MeOH) λ_max_ (log ε) 0 nm; ECD (c 0.06, MeOH) λ_max_ (Δε) 0 nm; 1D and 2D NMR (600 MHz, DMSO-*d*_6_)), see Tables 2 and 3; HRESI(+)- MS, *m*/*z* 430.3315 [M + H]^+^ (calcd for C_27_H_44_NO_3_^+^, 430.3321); Observed CCS, 213.52 Å^2^.

#### Bipolarolide O (**8**)

white solid; UV (c 0.06, MeOH) λ_max_ (log ε) 213 (3.58) nm; ECD (c 0.06, MeOH) λ_max_ (Δε) 220 (−21.91), 250 (+9.54) nm; 1D and 2D NMR (600 MHz, DMSO-*d*_6_)), see Tables 2 and 3; HRESI(+)- MS, *m*/*z* 430.3315 [M + H]^+^ (calcd for C_27_H_44_NO_3_^+^, 430.3321); Observed CCS, 215.23 Å^2^.

### ECD Calculations

To simplify computations, only the substructure (core part) without the side chain was considered for calculations. The conformational search for structures of two diastereomers (1a and 1b) was performed using Balloon v. 1.8.2.^39,40^ The resulting conformers were geometry optimized in Gaussian 16 (Rev. B.01)^41^ using density functional theory (DFT) at wB97XD^42^/6-311++G**^43,44^/PCM(MeOH)^45^ level. The ECD spectra for optimized structures of individual conformers were then calculated at the same level of theory using time-dependent DFT (TD-DFT).^46,47^ The final UV and ECD spectra were generated as Boltzmann averages based on free energies for the unique and stable conformers using a Gaussian band shape with a full width at half-maximum (fwhm) of 0.15 eV by GaussView 6.0.16 software.^48^ The calculated energies of the transitions were lower than the experimental ones, therefore the calculated spectra were scaled for a better comparison with the experiment (scaling factors are indicated in the figure captions).

### Antibacterial Assay

The chromatography fractions and purified compounds were investigated for antibacterial potential using a set of pathogenic bacteria including Gram-positive (*Enterococcus faecalis* (ATCC 29122), *Staphylococcus aureus* (ATCC25923), and *Streptococcus agalactiae* (ATCC 12386)) and Gram-negative (*Escherichia coli* (ATCC 25922), and *Pseudomonas aeruginosa* (ATCC 27853)). The assay was performed in 96-well plates in duplicates using the broth microdilution method following EUCAST guidelines and the standard test protocol.^49,50^ The fractions were prepared at 40 mg/mL in DMSO (VWR Chemicals, Radnor, PA, USA) as sample stocks, which were subsequently diluted with Milli-Q water and tested at 100 μg/mL as the final concentration. Stock solutions of the isolated pure compounds were prepared at 10 mM using 20% DMSO, further diluted with Milli-Q water, and screened for activity at a final concentration of 100 μM. Compounds that showed an inhibitory effect were then tested at different concentrations ranging from 12.5 to 125 μM. The results were plotted as a concentration-effect curve, and MIC values were recorded as the concentration (μM) corresponding to the optical density (OD600 nm) reading at 0.05. This was considered as a cutoff value to calculate the MIC values of the test compounds. In each experiment, gentamycin (Biochrom GmbH, part of Merck Millipore, Germany) at concentrations 32, 16, 8, 4, 2, 1, 0.5, 0.25, 0.13, 0.06, 0.03 μg/mL was included as a positive control, and 0.25% of DMSO and growth medium as a negative control.

## Data Availability

The raw NMR data of compounds **1**–**8** have been deposited in the Natural Products Magnetic Resonance Database (NP-MRD; www.np-mrd.org) and found at (NP0333791-NP0333798).

## Supporting Information Available

Morphological characteristics of the fungus; Isolation HPLC_MS chromatograms, HRESIMS spectra, 1D and 2D NMR spectra, calculated and experimental ECD spectra of compounds **1**–**8**; Antibacterial activity data (PDF)
