# Supplementary material for: Metabolomics-Guided Discovery of Bipolarolides H–O, New Ophiobolin-Type Sesterterpenes with Antibacterial Activity from the Marine-Derived Fungus Uzbekistanica storfjordensis sp. nov
Source: J Nat Prod. 2025 Jan 30;88(2):349–60. doi: 10.1021/acs.jnatprod.4c01105 (PMC11877529; doi:10.1021/acs.jnatprod.4c01105)
Supplement: Supplementary file 1 — np4c01105_si_001.pdf [file np4c01105_si_001.pdf]

Supporting Information

Metabolomics-Guided Discovery of Bipolarolides H-O, New Ophiobolin-Type Sesterterpenes with Antibacterial Activity from the Marine-Derived *Uzbekistanica storfjordensis* sp. nov.

Sailesh Maharjan[1],* Johan Mattias Isaksson[2, 3], Monika Krupova[3, 4], Teppo Rämä[1], Kine Østnes Hansen[1], Jeanette Hammer Andersen[1], Espen Holst Hansen[1]*

[1] Marbio, Faculty of Biosciences, Fisheries, and Economics, UiT-The Arctic University of Norway, Tromsø, Norway

[2] Department of Pharmacy (IFA), Faculty of Health Sciences, UiT-The Arctic University of Norway, Tromsø, Norway

[3]Department of Chemistry (IK), Faculty of Science and Technology, UiT-The Arctic University of Norway, Tromsø, Norway

[4] Hylleraas Centre for Quantum Molecular Sciences, Department of Chemistry (IK), Faculty of Science and Technology, UiT-The Arctic University of Norway, Tromsø, Norway

*** Corresponding authors:**

Sailesh Maharjan: Phone: +4796804941; Email: sailesh.maharjan@uit.no

Espen Holst Hansen: Phone: +4777649262; Email: espen.hansen@uit.no

List of Figures

Figure S 1. Morphological characteristics of *U. storfjordensis*. ... 8
Figure S 2. A best-scoring ML tree showing the placement of *U. storfjordensis* ... 9
Figure S 3. A best-scoring ML tree showing the placement of *U. storfjordensis* ... 10
Figure S 4. BPI Chromatogram of the first round of purification. ... 12
Figure S 5. BPI Chromatogram of the second round of purification of **1**. ... 12
Figure S 6. BPI Chromatogram of the second round of purification of **2** and **3**. ... 13
Figure S 7. BPI Chromatogram of the second round of purification of **4** and **5**. ... 13
Figure S 8. BPI Chromatogram of the second round of purification of **6**. ... 14
Figure S 9. BPI Chromatogram of the second round of purification of **7**. ... 14
Figure S 10. BPI Chromatogram of the second round of purification of **8**. ... 15
Figure S 11. Low-collision (top) and High-collision (bottom) energy mass spectra of **1** in ESI^+ mode. . 16
Figure S 12. ^{1}H NMR spectrum of **1**. ... 17
Figure S 13. ^{13}C NMR spectrum of **1**. ... 17
Figure S 14. HSQC spectrum of **1**. ... 18
Figure S 15. HMBC spectrum of **1**. ... 18
Figure S 16. H2BC spectrum of **1**. ... 19
Figure S 17. DQF-COSY spectrum of **1**. ... 19
Figure S 18. TOCSY ((DIPSI2) 60ms) spectrum of **1**. ... 20
Figure S 19. ROESY spectrum of **1**. ... 20
Figure S 20. Low-collision (top) and High-collision (bottom) energy mass spectra of **2** in ESI^+ mode. . 21
Figure S 21. ^{1}H NMR spectrum of **2**. ... 22
Figure S 22. ^{1}H NMR (Expanded) spectrum of **2**. ... 22
Figure S 23. ^{13}C NMR spectrum of **2**. ... 23
Figure S 24. HSQC spectrum of **2**. ... 23
Figure S 25. HMBC spectrum of **2**. ... 24
Figure S 26. COSY spectrum of **2**. ... 24
Figure S 27. DQF-COSY spectrum of **2**. ... 25
Figure S 28. TCOSY ((DIPSI2) 60ms) spectrum of **2**. ... 25
Figure S 29. ROESY spectrum of **2**. ... 26
Figure S 30. ROESY (Expanded) spectrum of **2**. ... 26
Figure S 31. Low-collision (top) and High-collision (bottom) energy mass spectra of **3** in ESI^+ mode. . 27
Figure S 32. ^{1}H NMR spectrum of **3**. ... 28
Figure S 33. ^{1}H NMR (Expanded) spectrum of **3**. ... 28
Figure S 34. ^{13}C NMR spectrum of **3**. ... 29
Figure S 35. ^{13}C NMR (Expanded) spectrum of **3**. ... 29
Figure S 36. HSQC spectrum of **3**. ... 30
Figure S 37. HMBC of spectrum **3**. ... 30
Figure S 38. HMBC (Expanded) spectrum of **3**. ... 31
Figure S 39. H2BC spectrum of **3**. ... 31
Figure S 40. DQF-COSY spectrum of **3**. ... 32
Figure S 41. ROESY spectrum of **3**. ... 32
Figure S 42. ROESY (Expanded) spectrum of **3**. ... 33
Figure S 43. Low-collision (top) and High-collision (bottom) energy mass spectra of **4** in ESI^+ mode. . 34
Figure S 44. ^{1}H NMR spectrum of **4**. ... 35
Figure S 45. ^{1}H NMR (Expanded) spectrum of **4**. ... 35
Figure S 46. ^{13}C NMR spectrum of **4**. ... 36

Figure S 47. ^{13}C NMR (Expanded) spectrum of **4**. 36
Figure S 48. HSQC+ HMBC spectrum of **4**. 37
Figure S 49. HSQC+ HMBC (Expanded) spectrum of **4**. 37
Figure S 50. HSQC+ H2BC spectrum of **4**. 38
Figure S 51. DQF-COSY spectrum of **4**. 38
Figure S 52. ROESY spectrum of **4**. 39
Figure S 53. Low-collision (top) and High-collision (bottom) energy mass spectra of **5** in ESI$^+$ mode. 40
Figure S 54. ^{1}H NMR spectrum of **5**. 41
Figure S 55. ^{1}H NMR spectrum of **5**. 41
Figure S 56. ^{13}C NMR spectrum of **5**. 42
Figure S 57. ^{13}C NMR (Expanded) spectrum of **5**. 42
Figure S 58. HSQC spectrum of **5**. 43
Figure S 59. HMBC spectrum of **5**. 43
Figure S 60. HMBC (Expanded) spectrum of **5**. 44
Figure S 61. DQF-COSY spectrum of **5**. 44
Figure S 62. DQF-COSY (Expanded) spectrum of **5**. 45
Figure S 63. ROESY spectrum of **5**. 45
Figure S 64. ROESY (Expanded) spectrum of **5**. 46
Figure S 65. Low-collision (top) and High-collision (bottom) energy mass spectra of **6** in ESI$^+$ mode. 47
Figure S 66. ^{1}H NMR spectrum of **6**. 48
Figure S 67. ^{1}H NMR (Expanded) spectrum of **6**. 48
Figure S 68. ^{13}C NMR spectrum **6**. 49
Figure S 69. ^{13}C NMR (Expanded) spectrum of **6**. 49
Figure S 70. HSQC spectrum of **6**. 50
Figure S 71. HMBC spectrum of **6**. 50
Figure S 72. DQF-COSY spectrum of **6**. 51
Figure S 73. ROESY spectrum of **6**. 51
Figure S 74. ROESY spectrum of **6**. 52
Figure S 75. ROESY (Expanded) spectrum of **6**. 52
Figure S 76. Low-collision (top) and High-collision (bottom) energy mass spectra of **7** in ESI$^+$ mode. 53
Figure S 77. ^{1}H NMR spectrum of **7**. 54
Figure S 78. ^{1}H NMR (Expanded) spectrum of **7**. 54
Figure S 79. ^{13}C NMR spectrum of **7**. 55
Figure S 80. ^{13}C NMR spectrum of **7**. 55
Figure S 81. HSQC spectrum of **7**. 56
Figure S 82. HSQC (Expanded) spectrum of **7**. 56
Figure S 83. HMBC spectrum of **7**. 57
Figure S 84. COSY spectrum of **7**. 57
Figure S 85. DQF-COSY spectrum of **7**. 58
Figure S 86. ROESY spectrum of **7**. 58
Figure S 87. ROESY (Expanded) spectrum of **7**. 59
Figure S 88. Low-collision (top) and High-collision (bottom) energy mass spectra of **8** in ESI$^+$ mode. 60
Figure S 89. ^{1}H NMR spectrum of **8**. 61
Figure S 90. ^{1}H NMR spectrum of **8**. 61
Figure S 91. ^{13}C NMR spectrum of **8**. 62
Figure S 92. ^{13}C NMR spectrum of **8**. 62
Figure S 93. HSQC spectrum of **8**. 63
Figure S 94. HSQC (Expanded) spectrum of **8**. 63
Figure S 95. HMBC spectrum of **8**. 64

Figure S 96. HMBC (Expanded) spectrum of **8**. 64
Figure S 97. HSQC+ HMBC spectrum of **8**. 65
Figure S 98. HSQC+ HMBC (Expanded1) spectrum of **8**. 65
Figure S 99. HSQC+ HMBC (Expanded2) spectrum **8**. 66
Figure S 100. DQF-COSY spectrum of **8**. 66
Figure S 101. ROESY spectrum of **8**. 67
Figure S 102. ROESY (Expanded) spectrum of **8**. 67
Figure S 103. (A) Experimental UV-Vis absorption spectra of compounds **1**–**8**. (B) Calculated and Experimental UV-Vis absorption spectra of compound **1**. The calculated transition energies were lower than the experimental ones, therefore the calculated energies were multiplied by a scaling factor of 0.82. 68
Figure S 104. Structures (1a and 1b) used for ECD calculations and their enantiomers (*ent*-1a and *ent*-1b) 69
Figure S 105. (A) Geometry-optimized structures of four stereoisomers of **1** showing the distances H-4/H-7, H-4/H_3-19, and H-4/H-13**.** (B) Comparison of calculated (for 1a and *ent*-1a) and experimental ECD spectra of **1**. (C) Comparison of calculated (for 1b and *ent*-1b) and experimental ECD spectra of **1**. The energies of the calculated spectra were scaled by a scaling factor of 0.82 in both B and C. 85
Figure S 106. Geometry-optimized structure of C-21 epimers. (A) Newman projection of C-14–C-21 showing key ROESY correlations in 2D and 3D (geometry-optimized) structures of 1a with 21*S* configuration. Comparison of calculated (for 1a and *ent*-1a) and experimental ECD spectra of **1**. The calculated energies were scaled by a scaling factor of 0.82. (B) Newman projection of C-14–C-21 in 2D and 3D (geometry-optimized structure showing measured distances H-22''/H-12''and H_3-27/H-15'/'') structures of 1c with 21*R* configuration. Comparison of calculated (for 1c and *ent*-1c) and experimental ECD spectra of **1**. The calculated energies were scaled by a scaling factor of 0.88. 85
Figure S 107. (B) Experimental or calculated ECD spectra of **2**–**8**, except **5**. The energies of the calculated spectra were scaled by a scaling factor of 0.82. 86
Figure S 108. Antibacterial effect of flash fractions (Fr. 1- 8). 87
Figure S 109. Concentration-effect curve for compounds **5**, **6**, and **8** against *Streptococcus agalactiae*. 87

List of Tables

Table S 1. Sequences used to build the phylogenetic trees. Sequences in bold were created for this study. 11
Table S 2. Geometry-optimized conformers of diastereomer 1a at wB97XD/6-311++G**/PCM(MeOH) level. 70
Table S 3. Gibbs free energy and Boltzmann population of eight geometry-optimized conformers of 1a. 70
Table S 4. Atomic coordinates for the geometry-optimized conformers of 1a (1a-01 to 04). 71
Table S 5. Atomic coordinates for the geometry-optimized conformers of 1a (1a-05 to 08). 72
Table S 6. Geometry-optimized conformers of diastereomer 1b at wB97XD/6-311++G**/PCM(MeOH) level. 74
Table S 7. Gibbs free energy and Boltzmann population of ten geometry-optimized conformers of 1b. 74
Table S 8. Atomic coordinates for the geometry-optimized conformers of 1b (1b-01 to 04). 75
Table S 9. Optimized atomic coordinates for the geometry-optimized conformers of 1b (1b-05 to 08). 76
Table S 10. Optimized atomic coordinates for the geometry-optimized conformers of 1b (1b-09 to 10). 77

Table S 11. Geometry-optimized conformers of diastereomer 1c at wB97XD/6-311++G**/PCM(MeOH) level. 79
Table S 12. Gibbs free energy and Boltzmann population of twelve geometry-optimized conformers of 1c. 79
Table S 13. Atomic coordinates for the geometry-optimized conformers of 1c (1c-01 to 04). 80
Table S 14. Atomic coordinates for the geometry-optimized conformers of 1c (1c-05 to 08). 81
Table S 15. Atomic coordinates for the geometry-optimized conformers of 1c (1c-09 to 12). 82

Taxonomic description of *Uzbekistanica storfjordensis* Rämä, sp. nov.

Figure S 11, Mycobank MB854047

Etymology: *storfjordensis*, referring to the municipality where the fungus was isolated from.

Sexual morph Undetermined. **Asexual morph** *Conidiomata* developing in culture, pycnidial, stromatic, in groups, globose, black, smooth, 330–520 µm (mean 406, n=7) in diameter, non-papillate, indistinct ostiolate; *Pycnidial walls* (Figure S 11b) 27–35 µm wide, 2-layered, outer layer 13–19 µm wide, *textura angularis – prismatica*, consisting of pigmented cells 3–6 µm in diameter, inner layer 12–20 µm wide, textura angularis, consisting of less pigmented-hyaline cells 5–8 µm in diam. ; *Conidiogenous cells* phialidic, hyaline, cylindrical–subcylindrical with rounded apex, (9.9-)14.7(-17.3) x (1.8-)1.9(-2.0) µm; *Conidia* (2.7-)3.2(-4.2) x (1.1-)1.5(-2.0) µm (n=50), unicellular to 1-septate, septum often subapical, oblong to ellipsoid, with rounded ends, hyaline, smooth.

Culture characteristics: Colonies on MEA were 24 cm in diameter after 13 days at 23°C, flat, margin dense with small lobes, felty with greyish white aerial hyphae that are frequent, colony surface greyish white, reverse same color, no pigments exudated to media, frequently, but sparsely with aerial hyphae, edge clearly delimited, densely filamentous, mycelium wraps around into round – ellipsoid aggregates (Figure S 11g).

Colonies on PDA were 31 cm in diameter after 14 days at 23°C, up to 5 mm thick, in inner parts glabrous colony with entire margin, towards the colony margins velvety with less aerial hyphae than in glabrous parts, white in color, with edge producing guttation droplets that become gray (white when young) and reverse surface gray in older colony parts. No clear pigments in the media, sparsely with round–ellipsoid mycelial aggregates.

Typus: Norway, Troms County, Storfjord Municipality, Taterneset, isolated from a deciduous driftwood log in the intertidal zone of a shore, N69°16'25.8" E19°56'25.8", 19th of May. 2010, T. Rämä, 009aD3.2; holotype TROM-F-910502 freeze-dried culture on malt extract agar medium (isotype TROM-F-26886 air-dried culture on malt extract agar), stored at Tromsø university museum fungarium; ex-holotype culture CBS 152423 (= M10F0001 preserved at the Norwegian marine biobank Marbank); ITS and LSU sequences in GenBank PP820634, PP820633, respectively; MycoBank MB854047.

Note: The fungus is only known from its type locality and was originally isolated to pure culture by Rämä et al. (2014)[4]. All other described *Uzbekistanica* species are sourced from terrestrial environments as saprotrophs from different hosts predominantly at high altitudes, whereas *U. storfjordensis* is the first species isolated from the marine environment and at high latitudes.

The species has clusters of pycnidia developing on rich media (PDA, MEA) prepared with fresh water. The morphology of the fungus could not be compared to the type species of the genus *U. rosa-hissariaceae*, which is described based on its sexual morph. Our isolate developed only an asexual morph with pycnidia embedded in a small stroma, similar in appearance to *U. yakuthanika*[1]. However, the conidiospores of *U. storfjordensis* are smaller than those of *U. yakuthanika*. The differences between *U. pruni* and *U. storfjordensis* include brown and multiseptate conidiospores[2]. Morphologically, the most closely related species is *U. vitis-viniferae*, from which *U. storfjordensis* differs with its larger pycnidia, longer and narrower conidiogenous cells, and shorter, straight, and 1-septated conidia[3]. *U. vitis-viniferae* is also phylogenetically the most closely related species (Figure S 22, Figure S 33) yet is distinct based on the more variable ITS region. Based on a BLAST search against the nr-database of the NCBI, the closest ITS sequence match was the *U. vitis-viniferae* culture isolate CPC:35793 derived from the type-material (GenBank MT223867) with 98.8% sequence similarity (number of identical bases 503/509). The 28S sequence had the closest sequence match with the same *U. vitis-viniferae* isolate and *U. pruni* type-isolate MFLU 17-2136 (GenBank NG_068927), both with 99.8% sequence identity (number of identical bases 816/817 and 810/811, respectively). It is also worth noting that several undescribed *Pleosporales* and *Melanommataceae* isolates from different woody substrates in Eurasia and USA are phylogenetically closely related with high ITS similarity to *U. storfjordensis* (Figure S 22, Figure S 33).

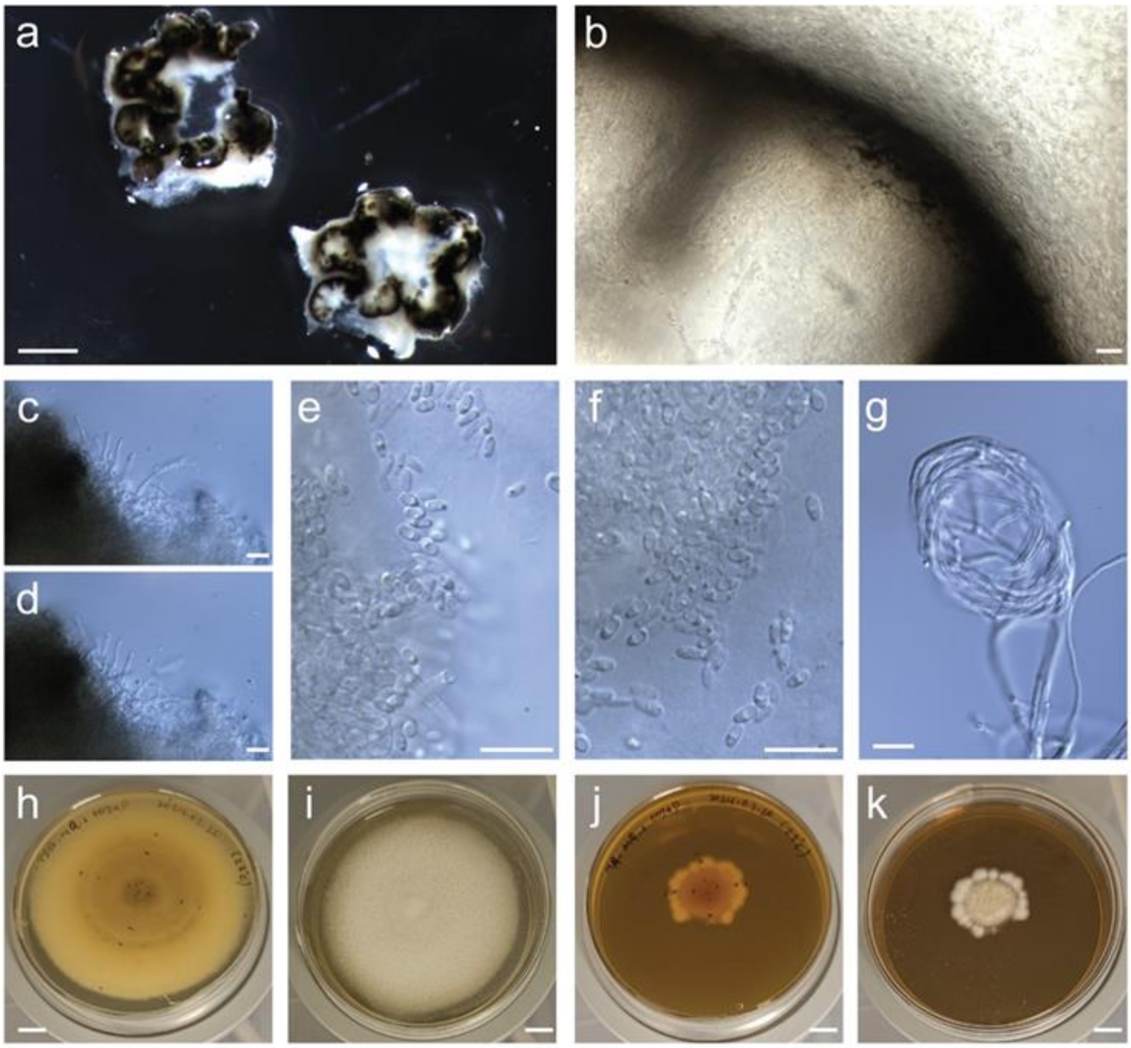

Figure S 11. Morphological characteristics of *U. storfjordensis*.
a. Vertical sections of clusters of pycnidia that have developed in culture. **b.** 2-layered pycnidial wall consisting of a layer of pigmented and a layer of less pigmented-hyaline cells. **c-d.** Phialides. **e-f.** Conidia. **g.** Hyphal aggregate. **h-k.** 28-days old cultures on PDA (h. reverse, i. upperside) and MEA (j. reverse, k upperside). Scale bar: a=500 μm, b-k=10 μm.

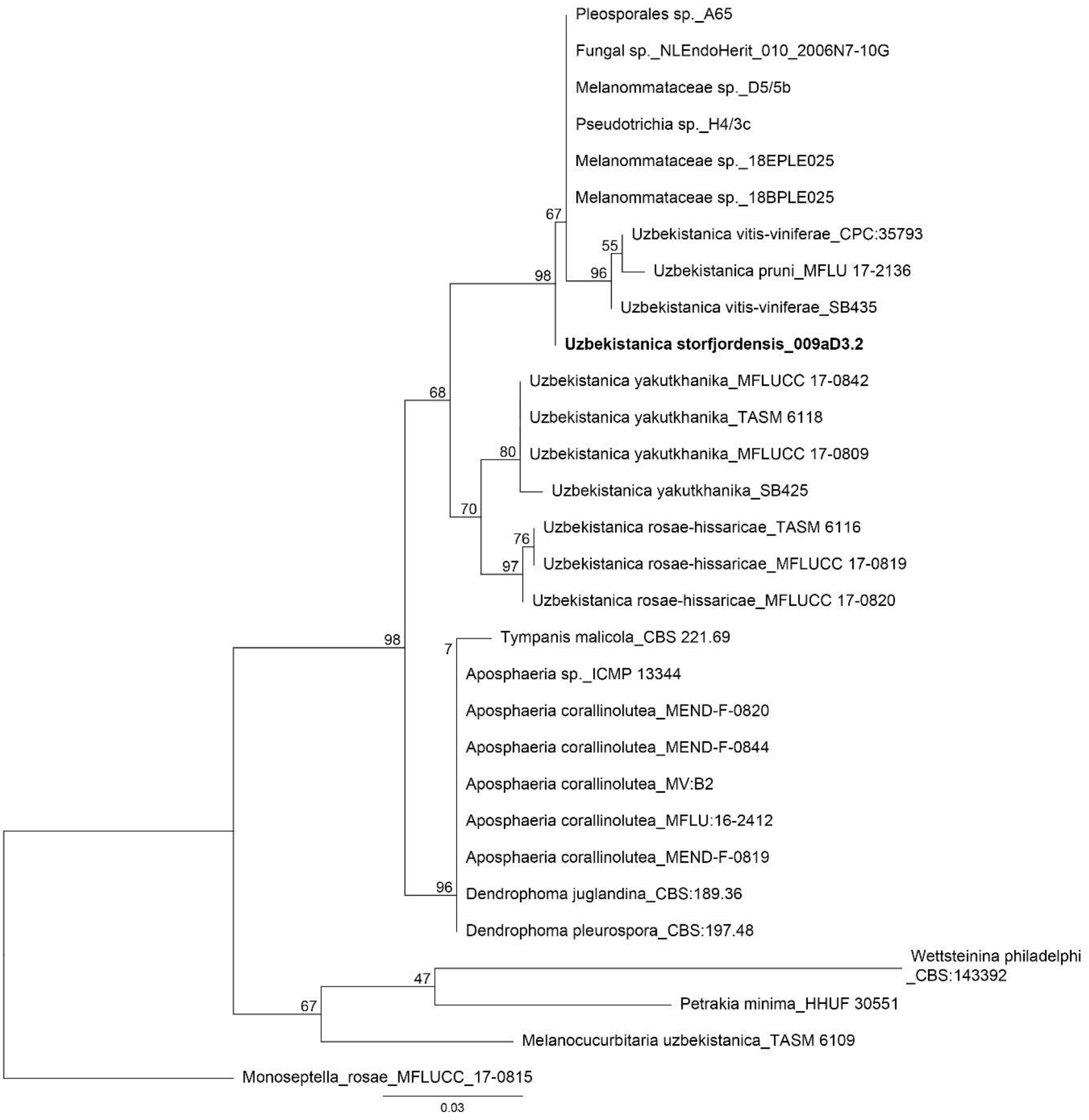

Figure S 22. A best-scoring ML tree showing the placement of *U. storfjordensis* (in bold) within the *Melanommataceae*. The tree was built using publicly available ITS sequences in GenBank (Table S 11) with isolate names following the taxon names[5]. *Monoseptella rosea* was used as an outgroup. The numbers at the nodes represent bootstrap support values from a 1000 generations analysis using RAxML version 8.2.11 (Stamatakis 2014) in Geneious Prime® (available at www.geneious.com). General time reversible model was used as a substitution matrix.

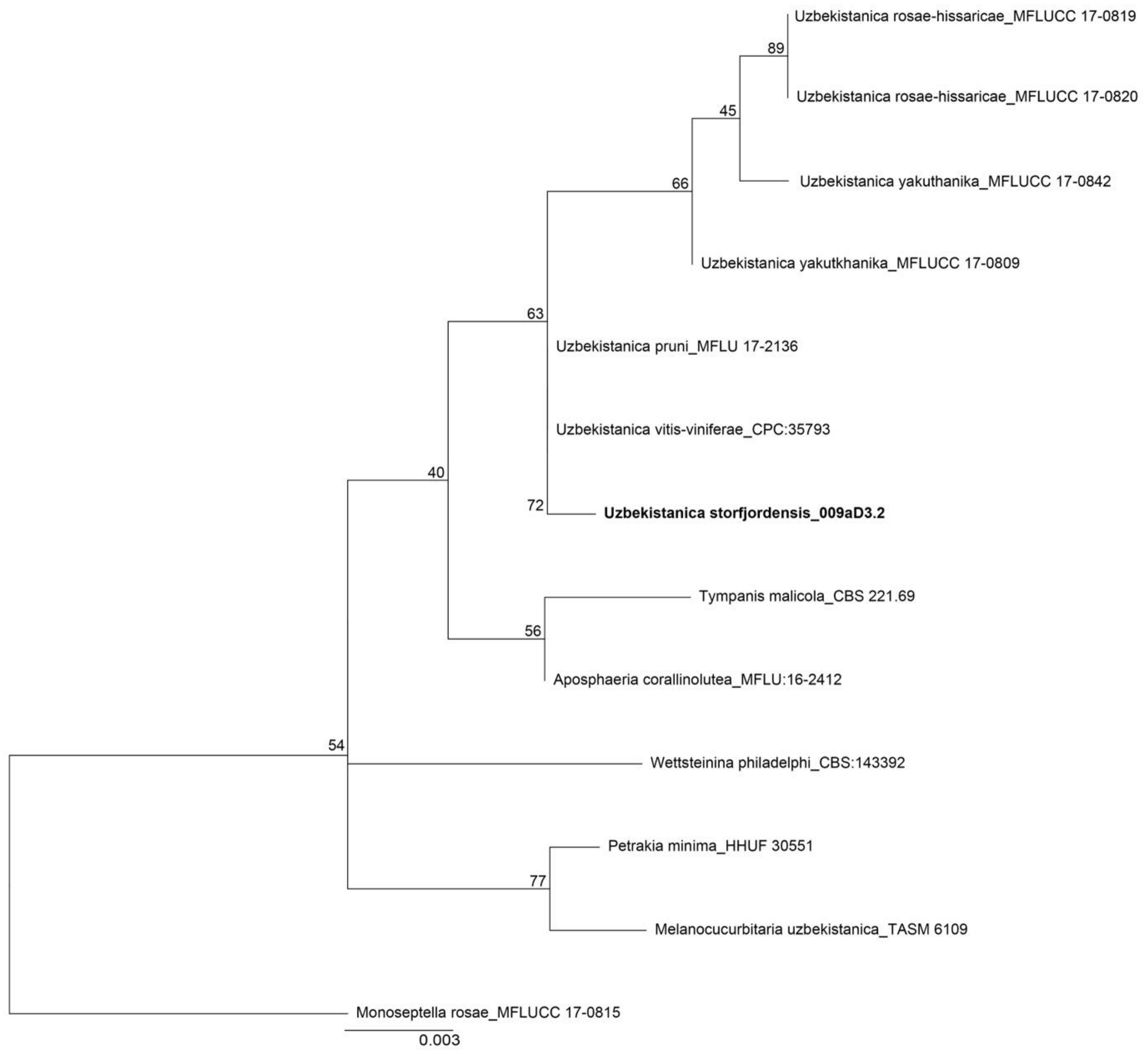

Figure S 33. A best-scoring ML tree showing the placement of *U. storfjordensis* (in bold) within the *Melanommataceae*. The tree was built using publicly available 28S sequences in GenBank (Table S 11) with isolate names following the taxon names[5]. *Monoseptella rosea* was used as an outgroup. The numbers at the nodes represent bootstrap support values from a 1000 generations analysis using RAxML version 8.2.11 (Stamatakis 2014) in Geneious Primeâ (available at www.geneious.com). A general time reversible model was used as a substitution matrix.

References

(1) Wanasinghe, D. N.; Phukhamsakda, C.; Hyde, K. D.; Jeewon, R.; Lee, H. B.; Gareth Jones, E. B.; Tibpromma, S.; Tennakoon, D. S.; Dissanayake, A. J.; Jayasiri, S. C.; et al. Fungal diversity notes 709–839: taxonomic and phylogenetic contributions to fungal taxa with an emphasis on fungi on Rosaceae. *Fungal diversity* **2018**, *89* (1), 1-236. DOI: 10.1007/s13225-018-0395-7.

(2) Hyde, K. D.; Dong, Y.; Phookamsak, R.; Jeewon, R.; Bhat, D. J.; Jones, E. B. G.; Liu, N.-G.; Abeywickrama, P. D.; Mapook, A.; Wei, D.; et al. Fungal diversity notes 1151–1276: taxonomic and phylogenetic contributions on genera and species of fungal taxa. *Fungal diversity* **2020**, *100* (1), 5-277. DOI: 10.1007/s13225-020-00439-5.
(3) Crous, P. W.; Wingfield, M. J.; Schumacher, R. K.; Akulov, A.; Bulgakov, T. S.; Carnegie, A. J.; Jurjević, Ž.; Decock, C.; Denman, S.; Lombard, L.; et al. New and Interesting Fungi. 3. *Fungal Systematics and Evolution* **2020**, *6* (1), 157-231. DOI: 10.3114/fuse.2020.06.09.
(4) Rämä, T.; Nordén, J.; Davey, M. L.; Mathiassen, G. H.; Spatafora, J. W.; Kauserud, H. Fungi ahoy! Diversity on marine wooden substrata in the high North. *Fungal Ecol* **2014**, *8*, 46-58. DOI: 10.1016/j.funeco.2013.12.002.
(5) Benson, D. A.; Karsch-Mizrachi, I.; Lipman, D. J.; Ostell, J.; Wheeler, D. L. GenBank. *Nucleic Acids Res.* **2005**, *33* (Database issue), D34-38. DOI: 10.1093/nar/gki063 From NLM Medline.

Table S 11. Sequences used to build the phylogenetic trees. Sequences in bold were created for this study.

Species	Isolate	ITS	LSU
Aposhaeria corallinolutea	MEND-F-0820	OQ357921	
A. corallinolutea	MEND-F-0844	OQ357923	
A. corallinolutea	MV:B2	OQ411094	
A. corallinolutea	MFLU:16-2412	MT177916	MT177943
A. corallinolutea	MEND-F-0819	OQ357920	
A. sp.	ICMP 13344	PP097810	
Dendrophoma juglandina	CBS:189.36	MH855766	
D. pleurospora	CBS:197.48	MH856307	
Fungal sp.	NLEndoHerit_1010_2006N7-10G	JX978239	
Melanocucurbitaria uzbekistanica	TASM 6109	NR_157519	NG_059865
Melanommataceae sp.	D5/5b	MG020344	
Melanommataceae sp.	18EPLE025	MT862346	
Melanommataceae sp.	18BPLE025	MT645938	
Monoseptella rosae	MFLUCC 17-0815	MG828916	MG829026
Petrakia minima	HHUF 30551	NR_155702	NG_059812
Pleosporales sp.	A65	KX611042	
Pseudotrichia sp.	H4/3c	MG020321	
Tympanis malicola	CBS 221.69	MK314579	MK314632
Uzbekistanica pruni	MFLU 17-2136	NR_168244	NG_068927
U. rosae-hissaricae	MFLUCC 17-0819	MG828975	MG829087
U. rosae-hissaricae	MFLUCC 17-0820	MG829088	MG828976
U. rosae-hissaricae	TASM 6116	NR_157550	
U. storfjordensis	**009aD3.2**	**PP820634**	**PP820633**
U. vitis-viniferae	CPC:35793	MT223867	MT223938
U. vitis-viniferae	SB435	OK560355	
U. yakuthanika	MFLUCC 17-0809	MG828977	MG829089
U. yakuthanika	MFLUCC 17-0842	MG828978	MG829090
U. yakuthanika	TASM 6118	NR_157550	
U. yakuthanika	SB425	OK560354	
Wettsteinina philadelphi	CBS:143392	NR_159071	NG_063961

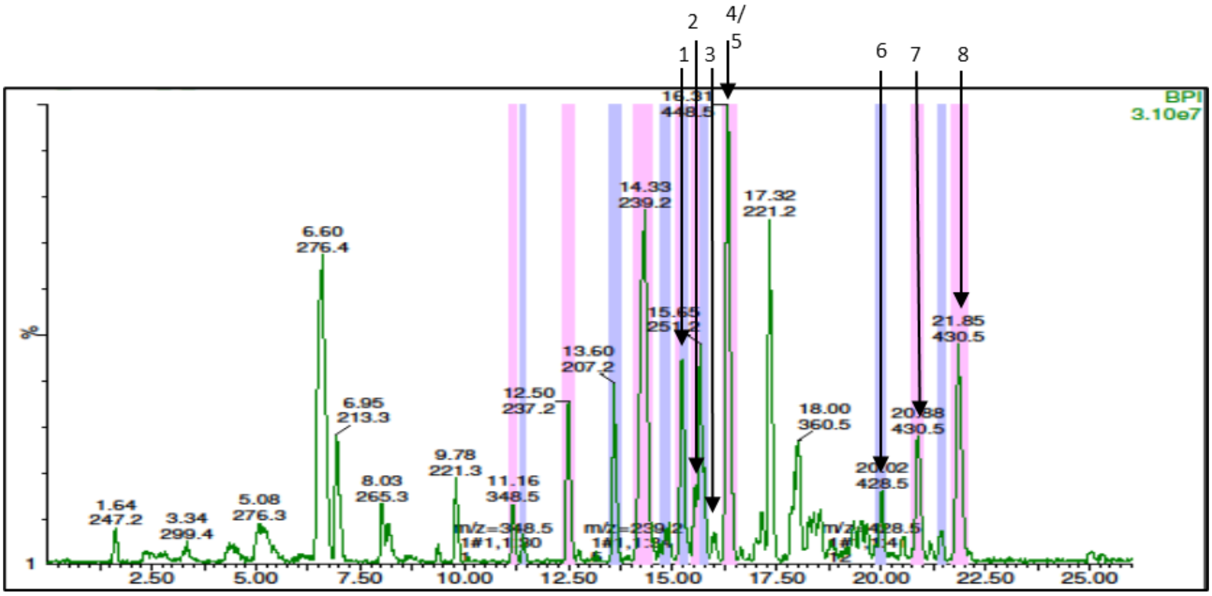

Figure S 44. BPI Chromatogram of the first round of purification.

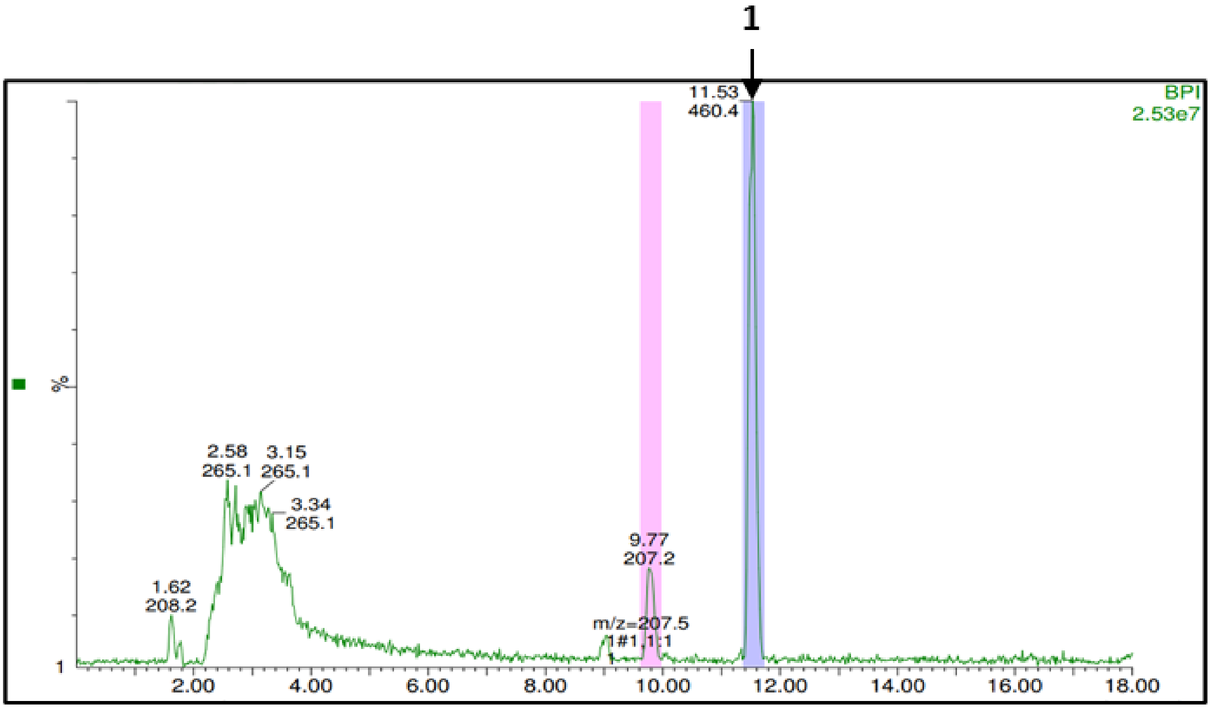

Figure S 55. BPI Chromatogram of the second round of purification of **1**.

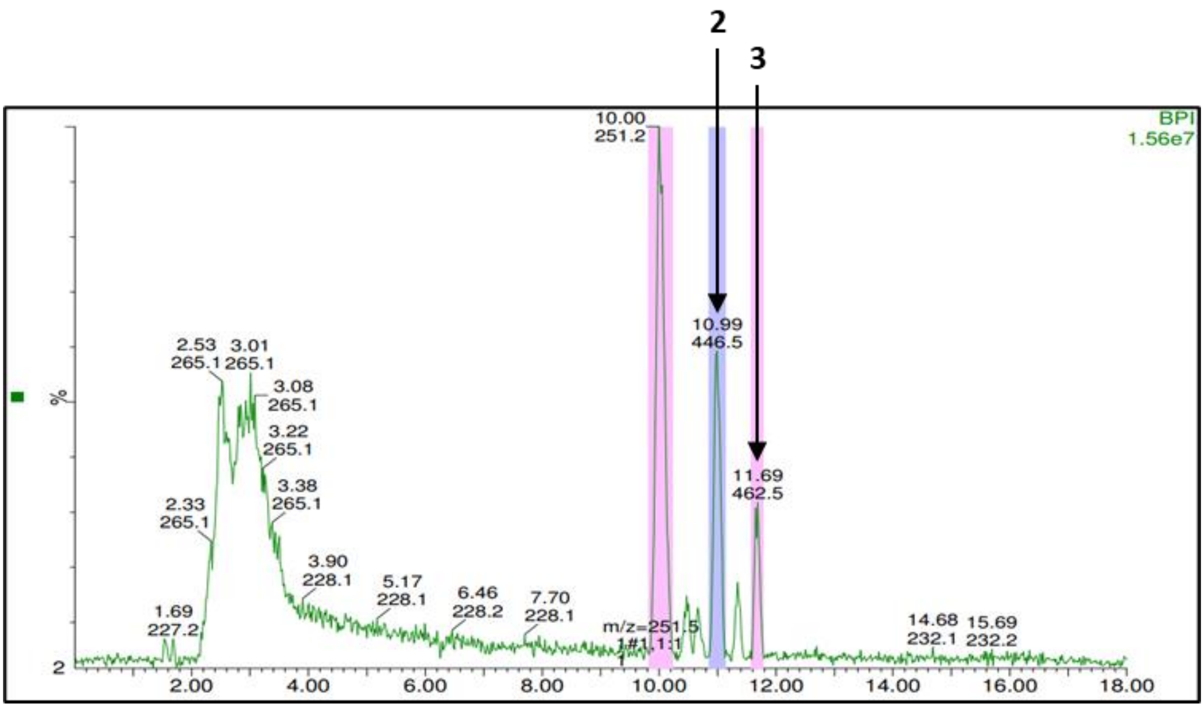

Figure S 66. BPI Chromatogram of the second round of purification of **2** and **3**.

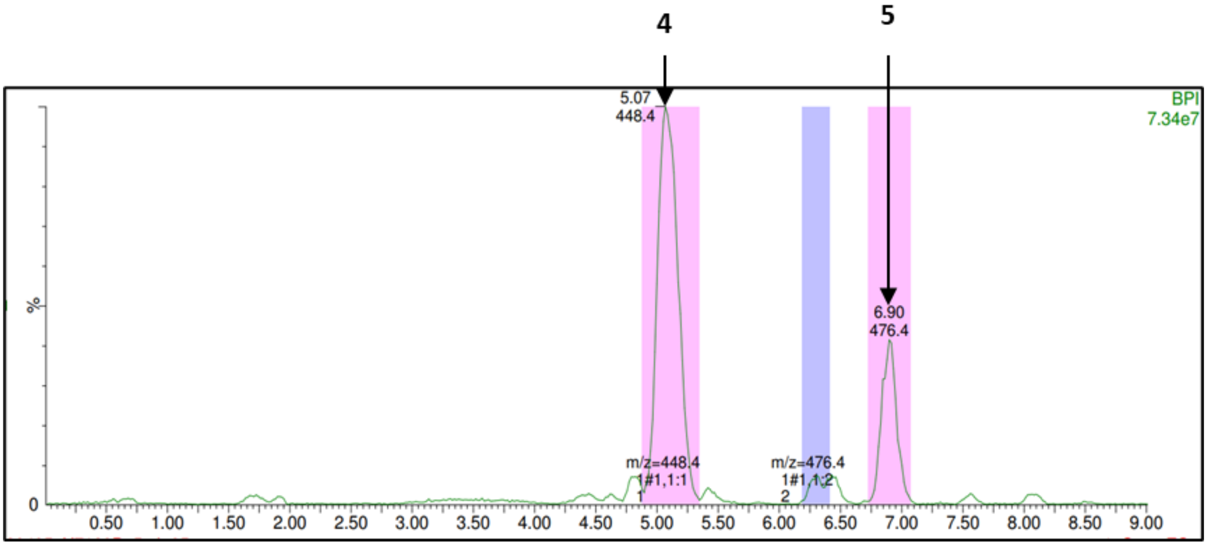

Figure S 77. BPI Chromatogram of the second round of purification of **4** and **5**.

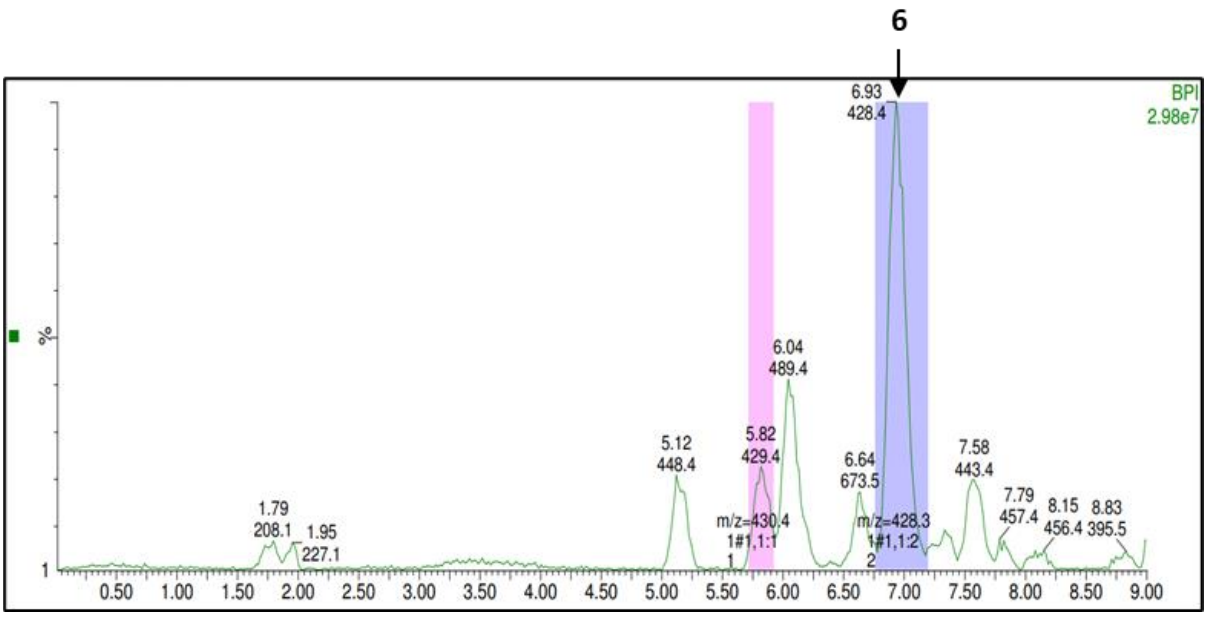

Figure S 88. BPI Chromatogram of the second round of purification of **6**.

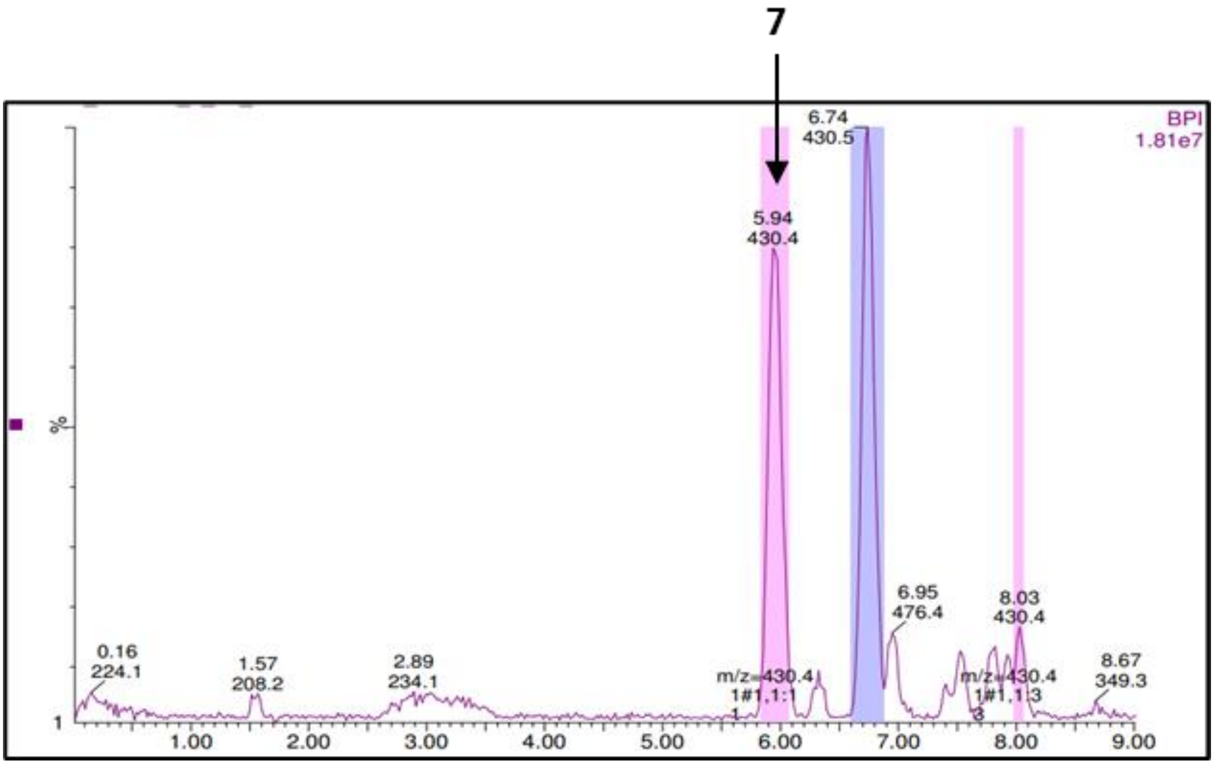

Figure S 99. BPI Chromatogram of the second round of purification of **7**.

8

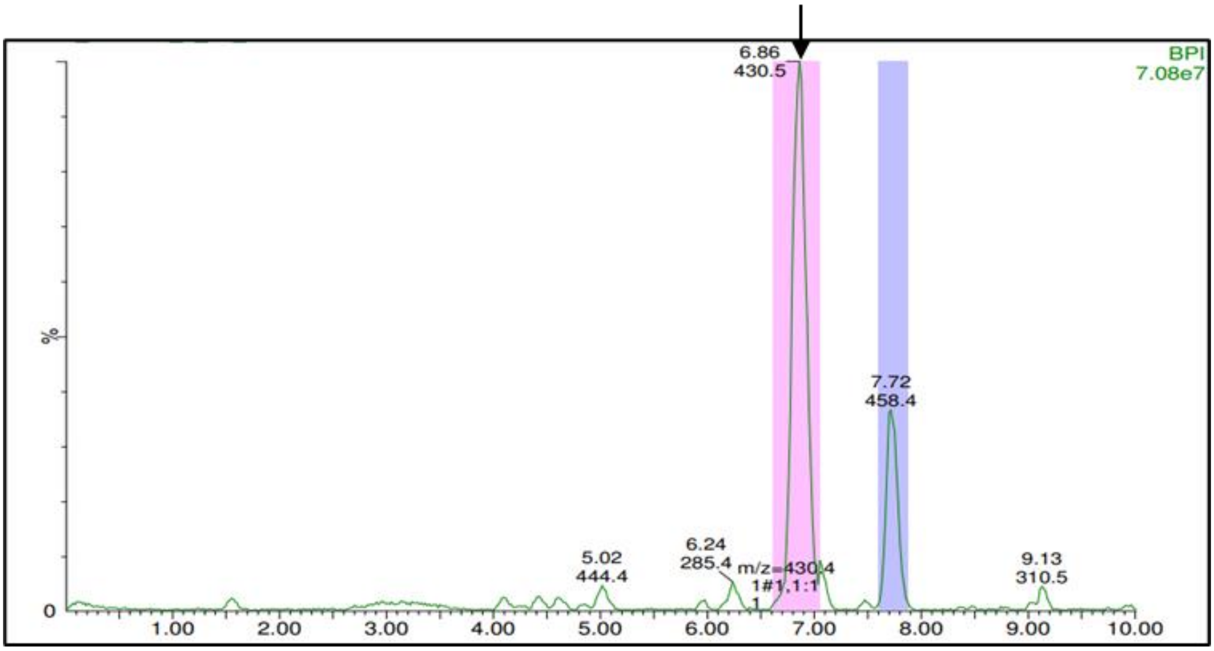

Figure S 1010. BPI Chromatogram of the second round of purification of **8**.

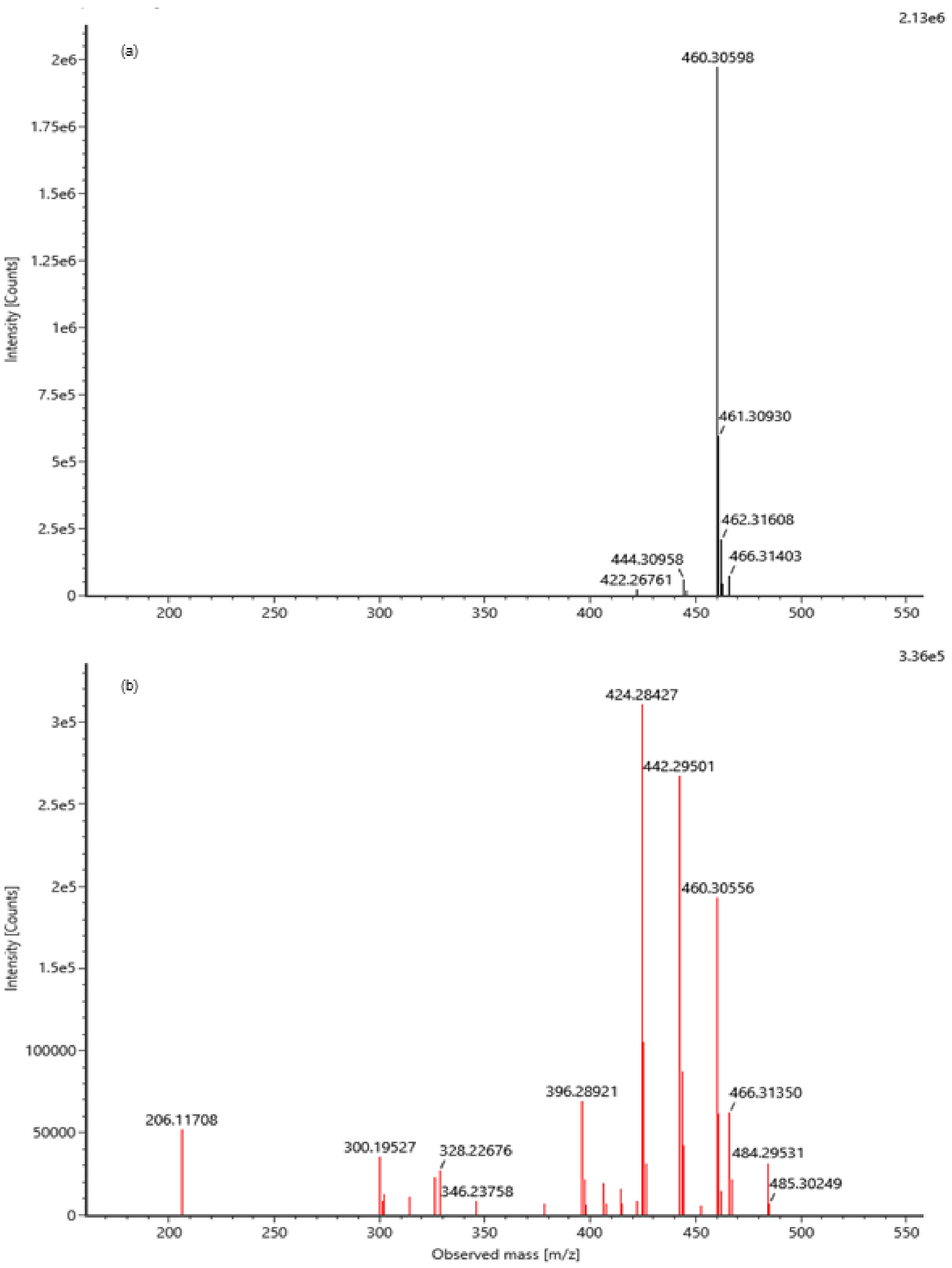

Figure S 1111. Low-collision (top) and High-collision (bottom) energy mass spectra of **1** in ESI^+ mode.

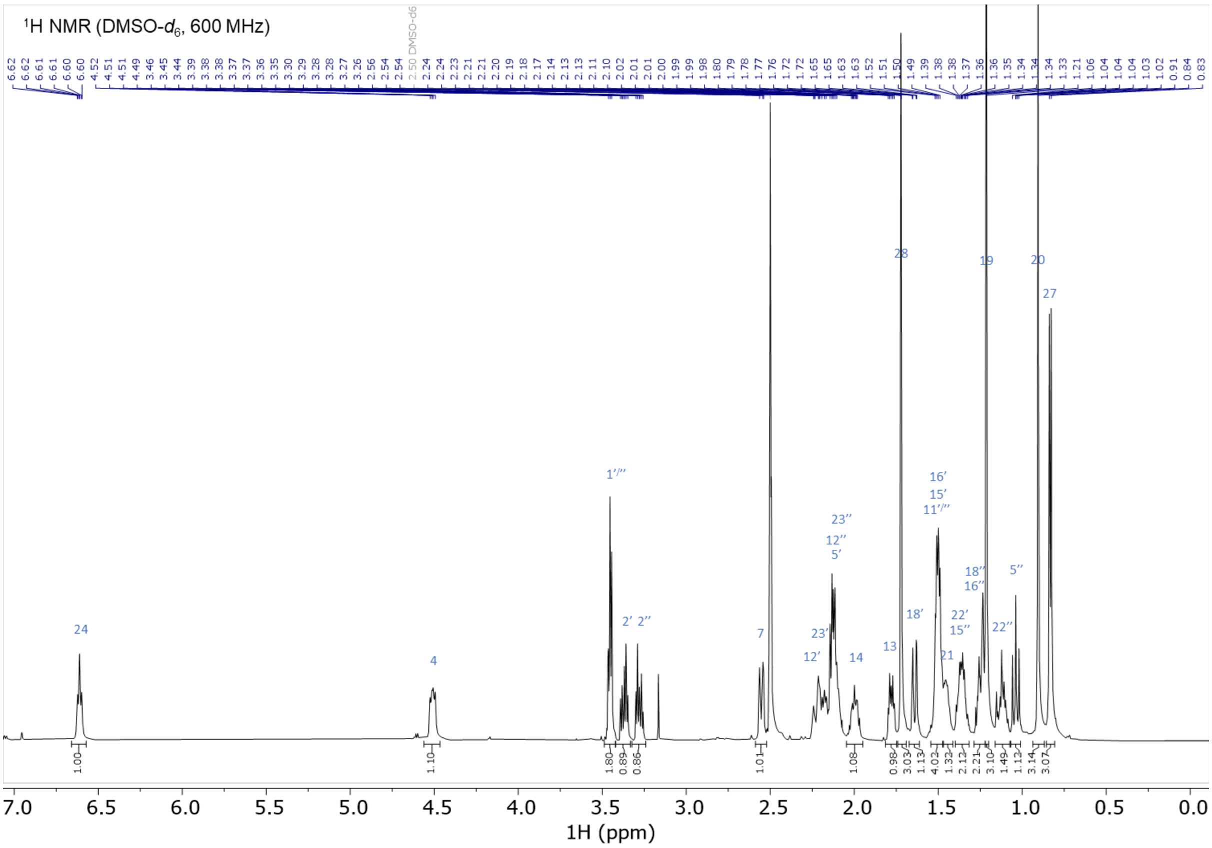

Figure S 1212. ^{1}H NMR spectrum of **1**.

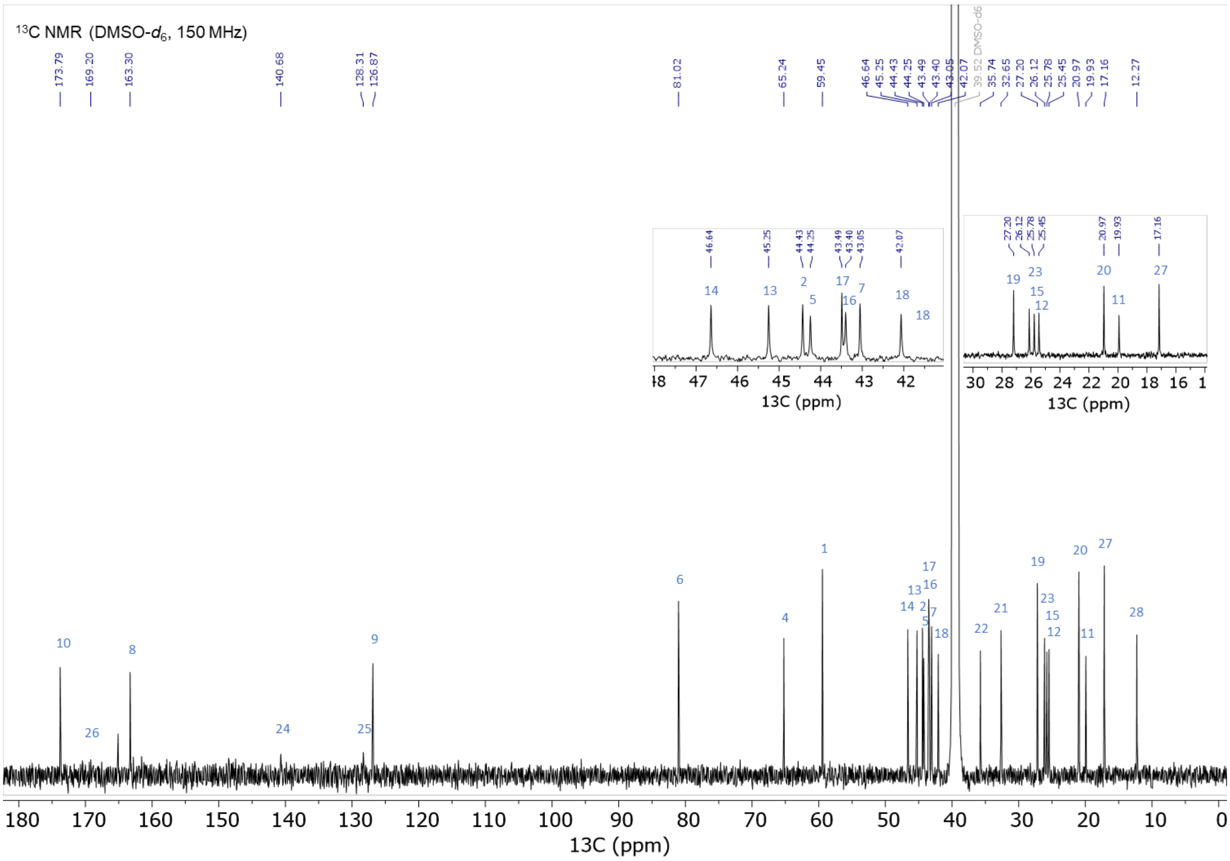

Figure S 1313. ^{13}C NMR spectrum of **1**.

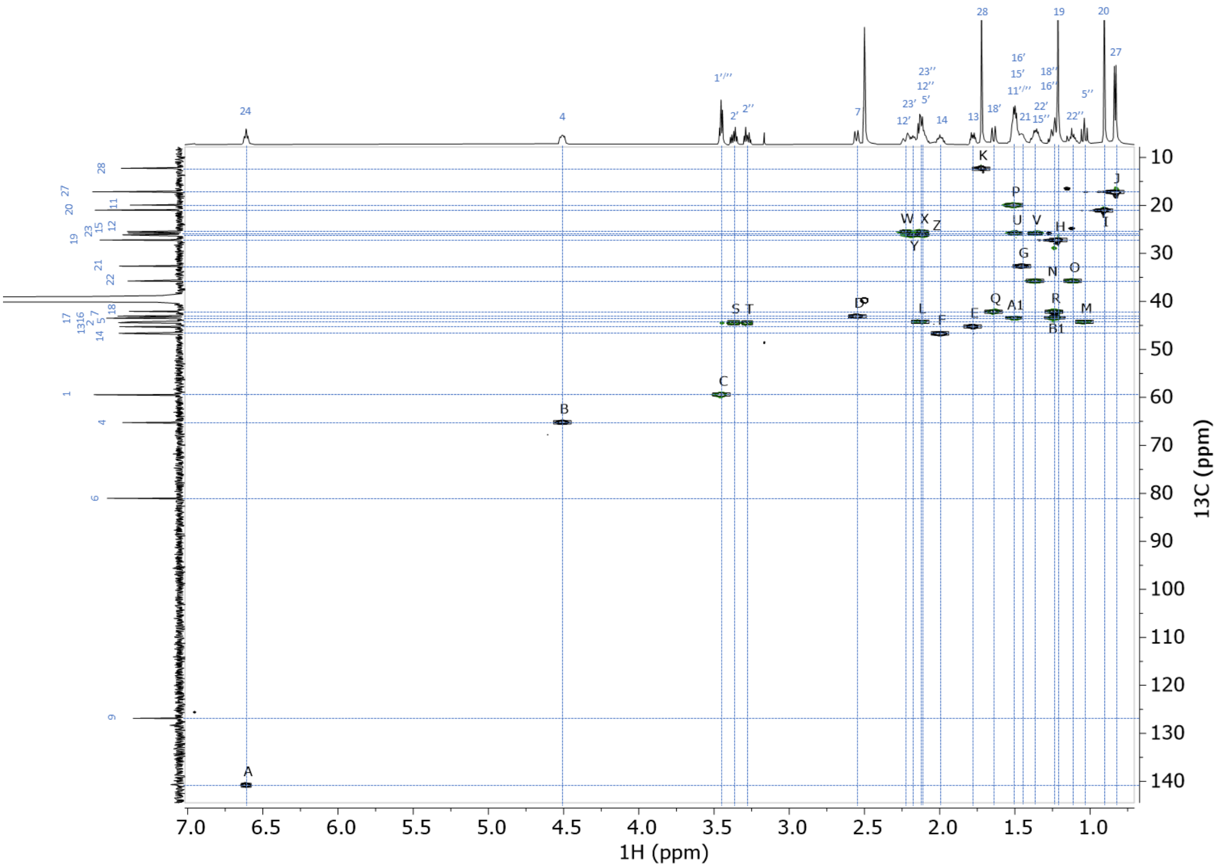

Figure S 1414. HSQC spectrum of **1**.

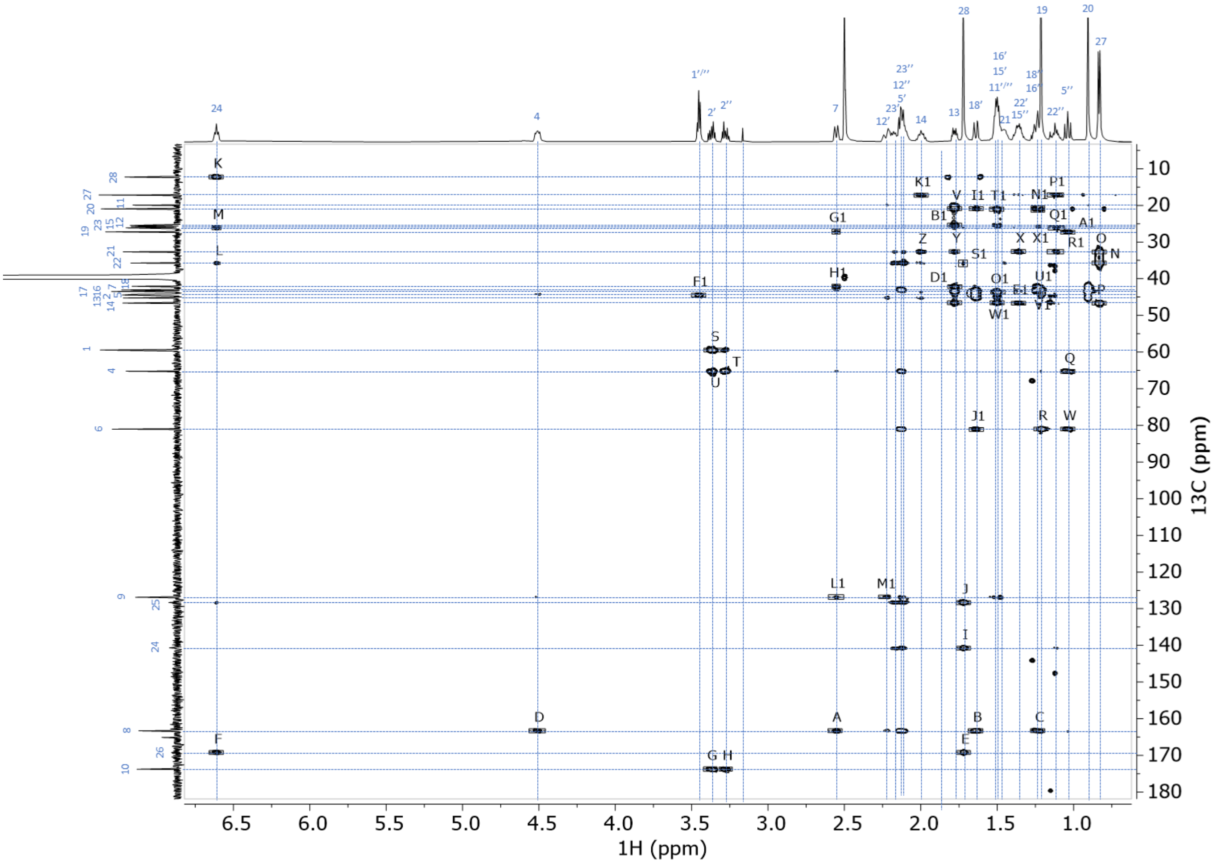

Figure S 1515. HMBC spectrum of **1**.

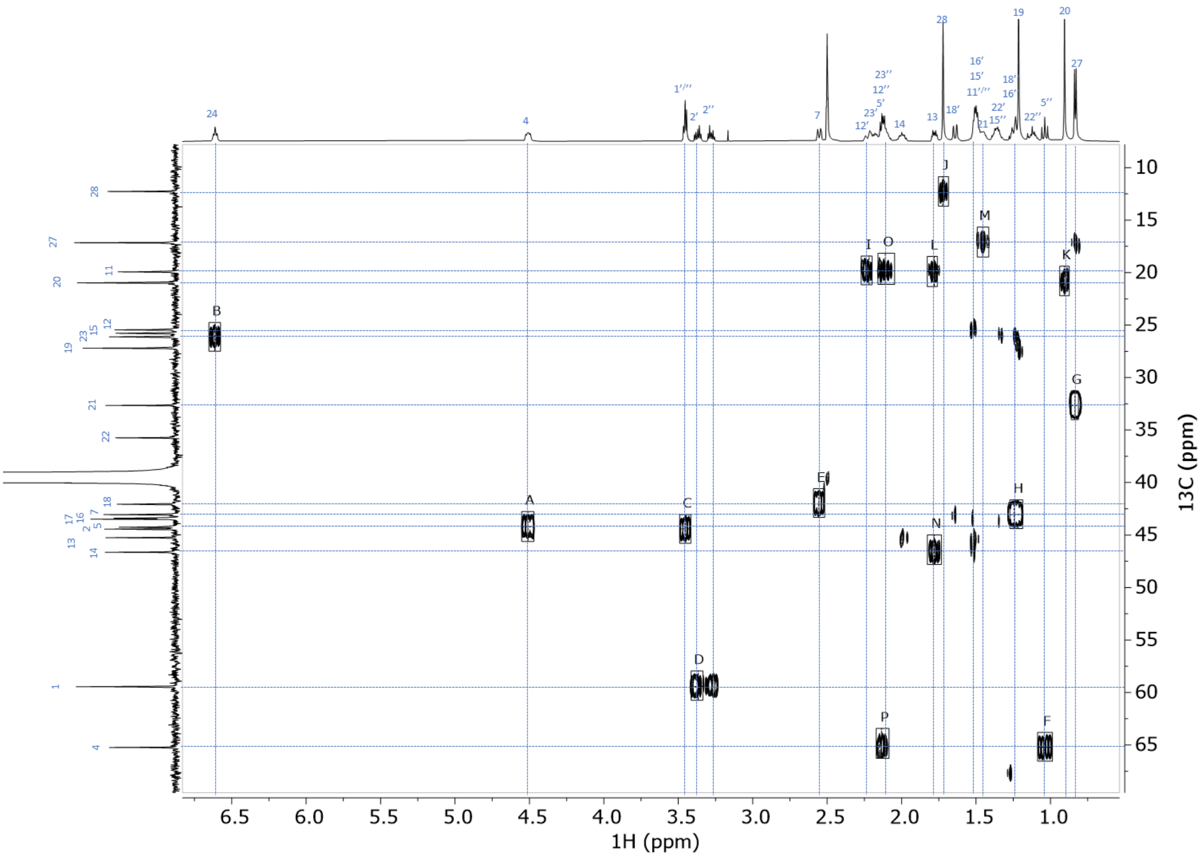

Figure S 1616. H2BC spectrum of **1**.

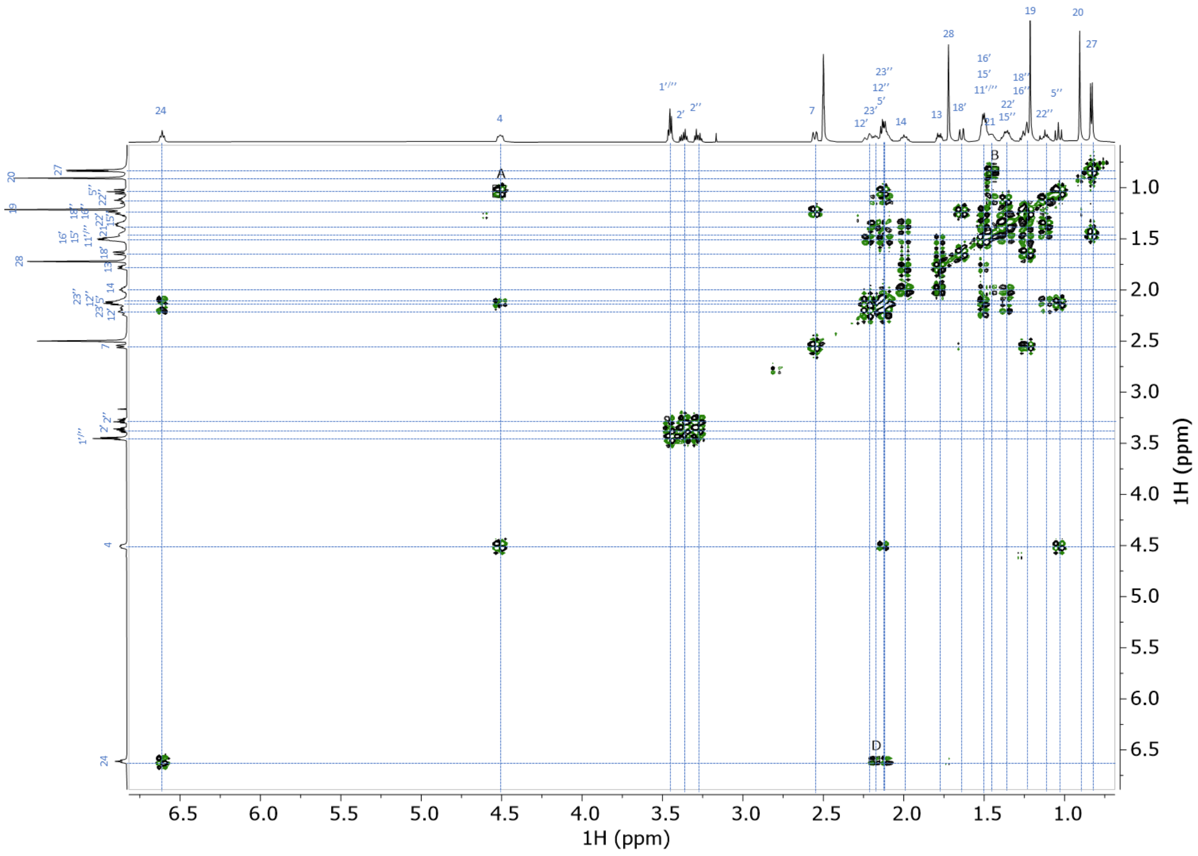

Figure S 1717. DQF-COSY spectrum of **1**.

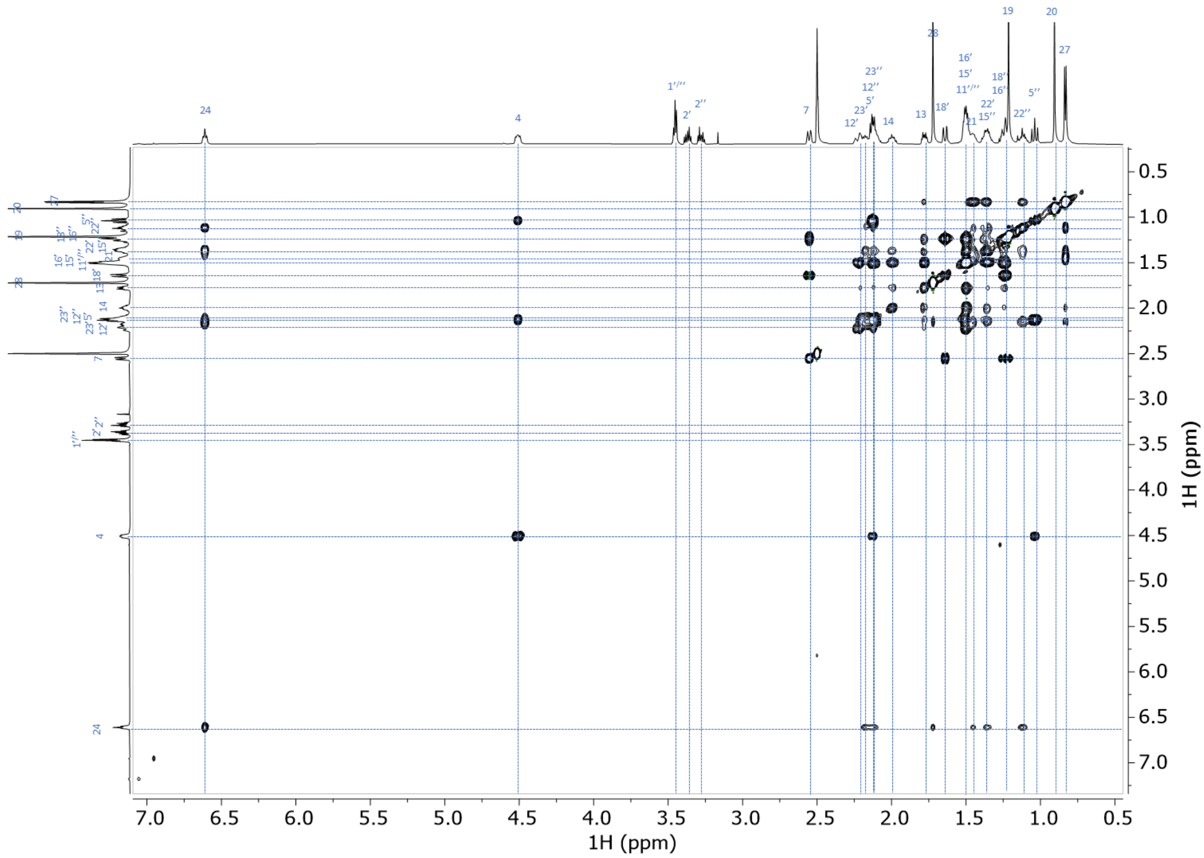

Figure S 1818. TOCSY ((DIPSI2) 60ms) spectrum of **1**.

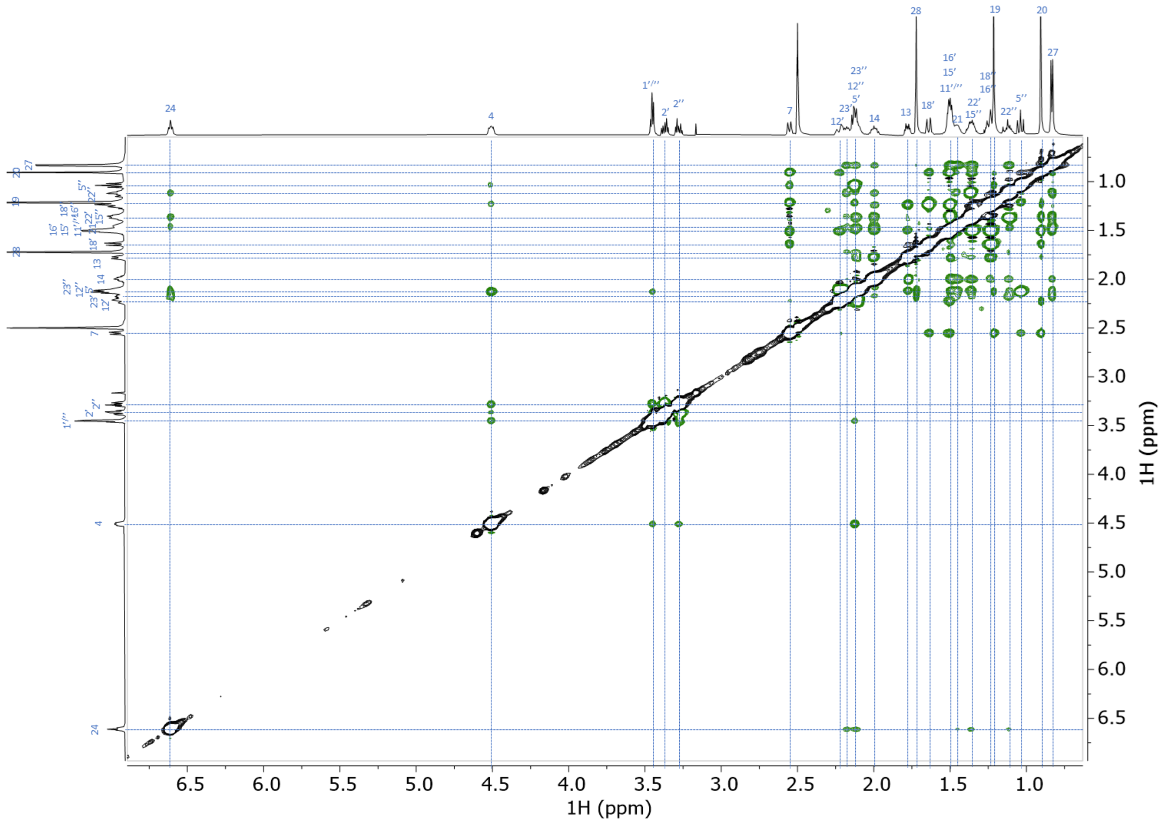

Figure S 1919. ROESY spectrum of **1**.

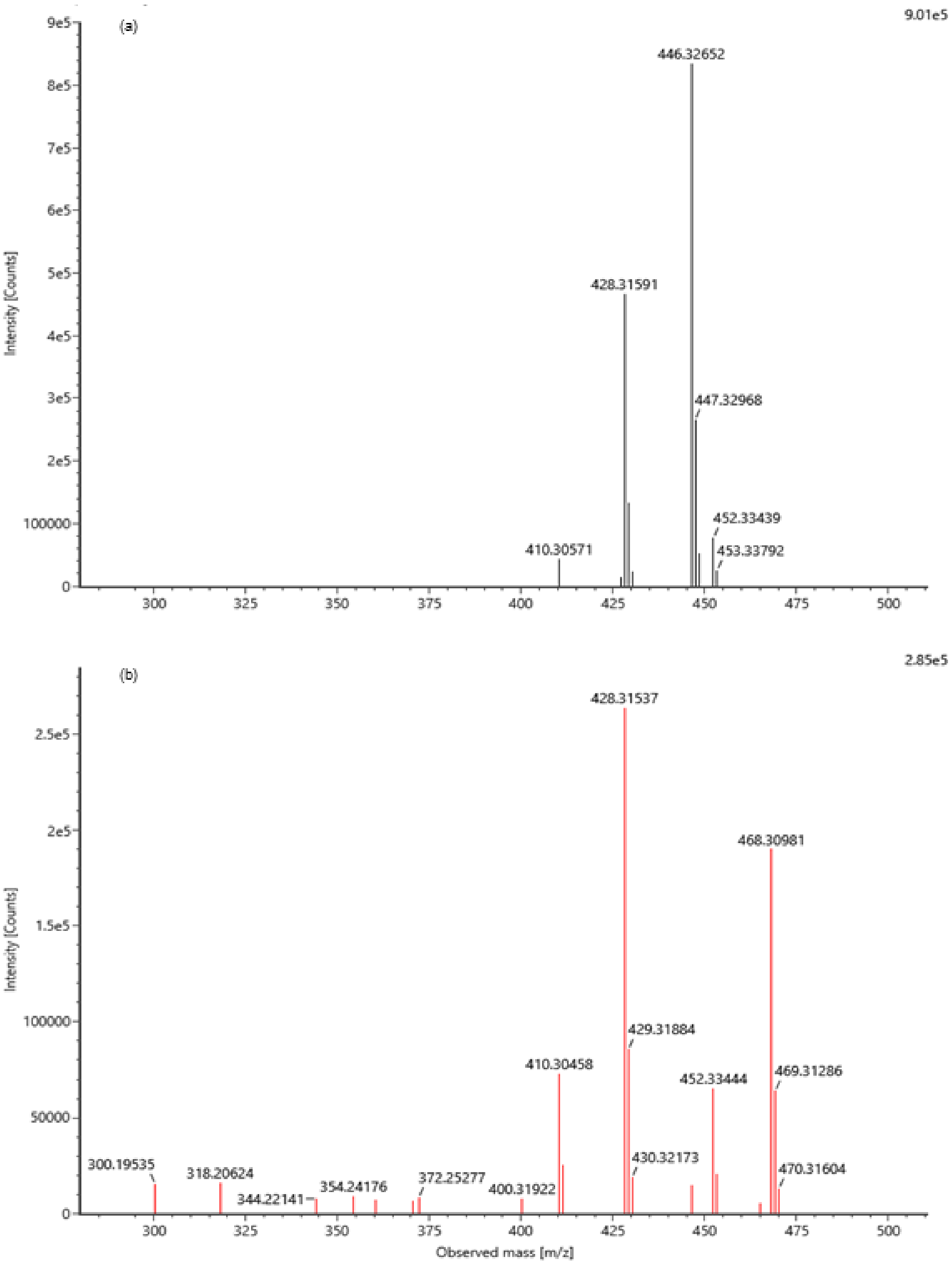

Figure S 2020. Low-collision (top) and High-collision (bottom) energy mass spectra of **2** in ESI^+ mode.

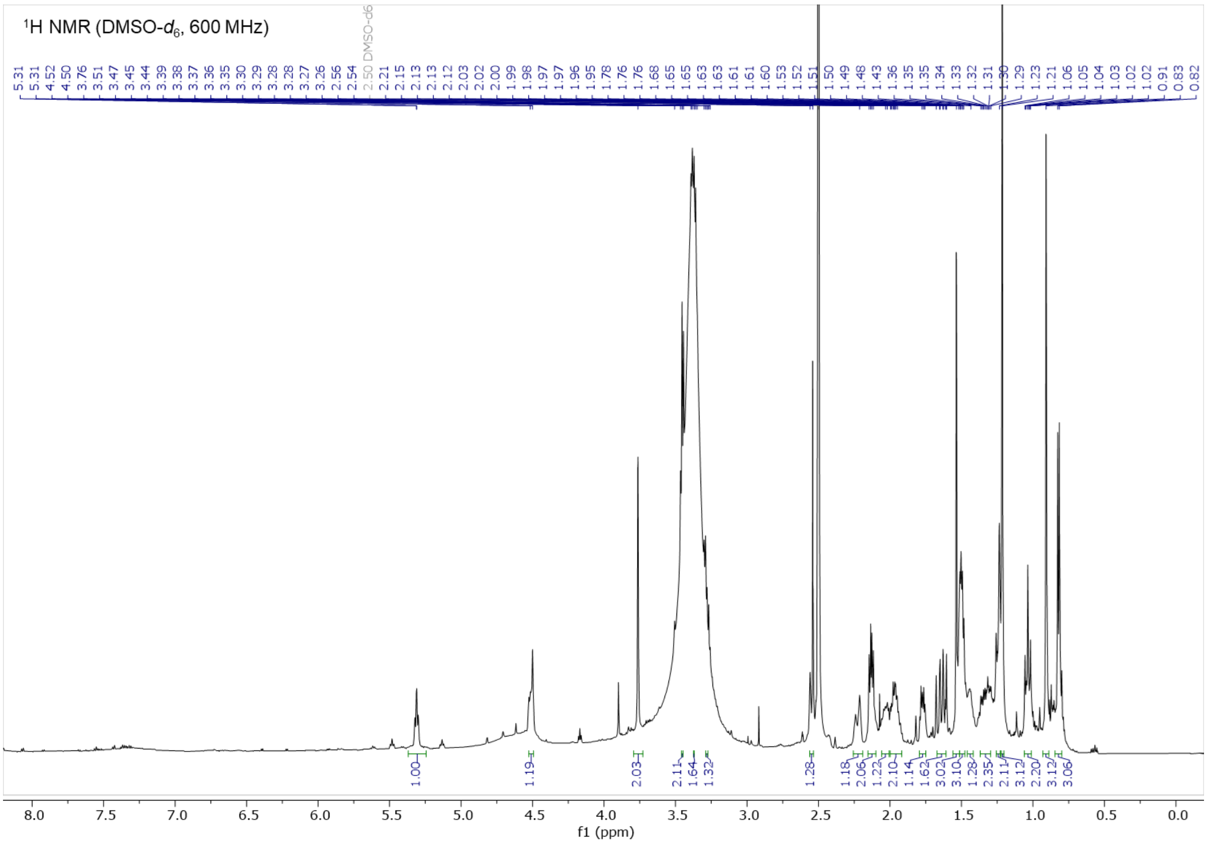

Figure S 2121. ^{1}H NMR spectrum of **2**.

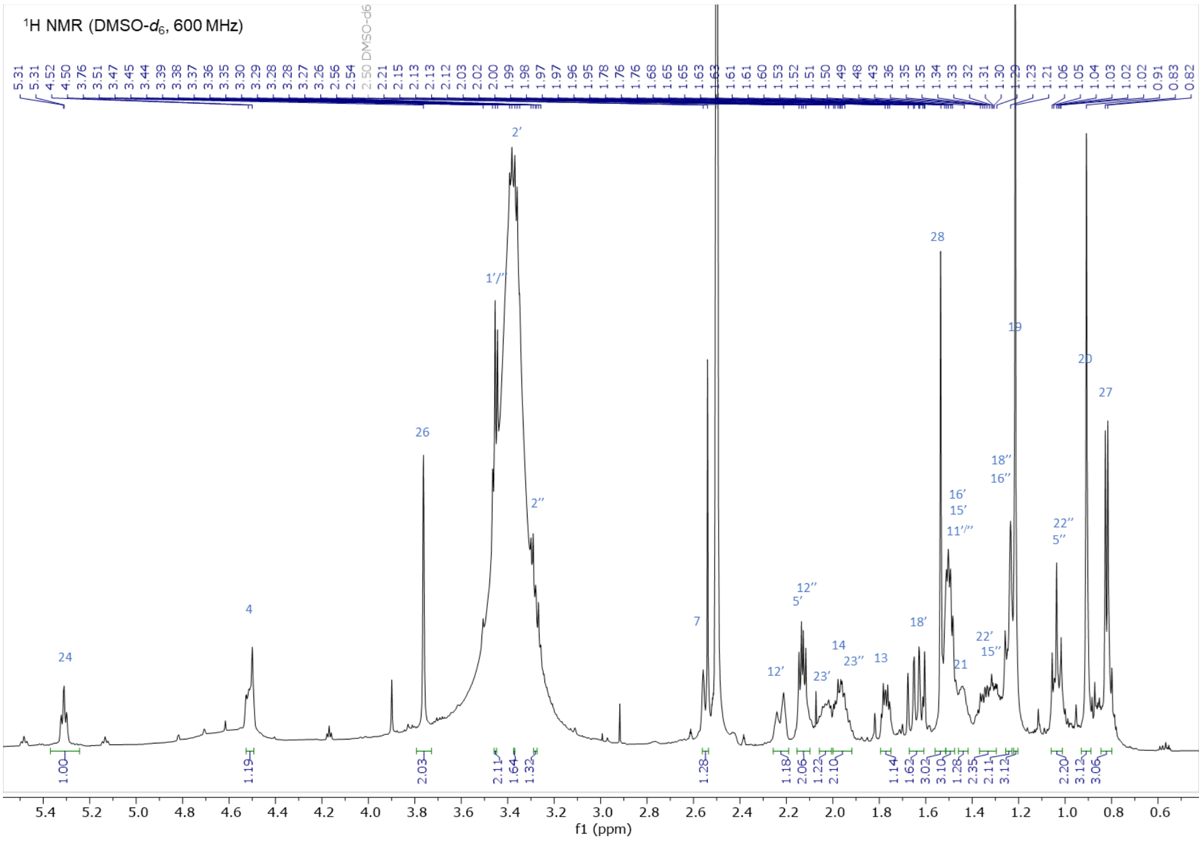

Figure S 2222. ^{1}H NMR (Expanded) spectrum of **2**.

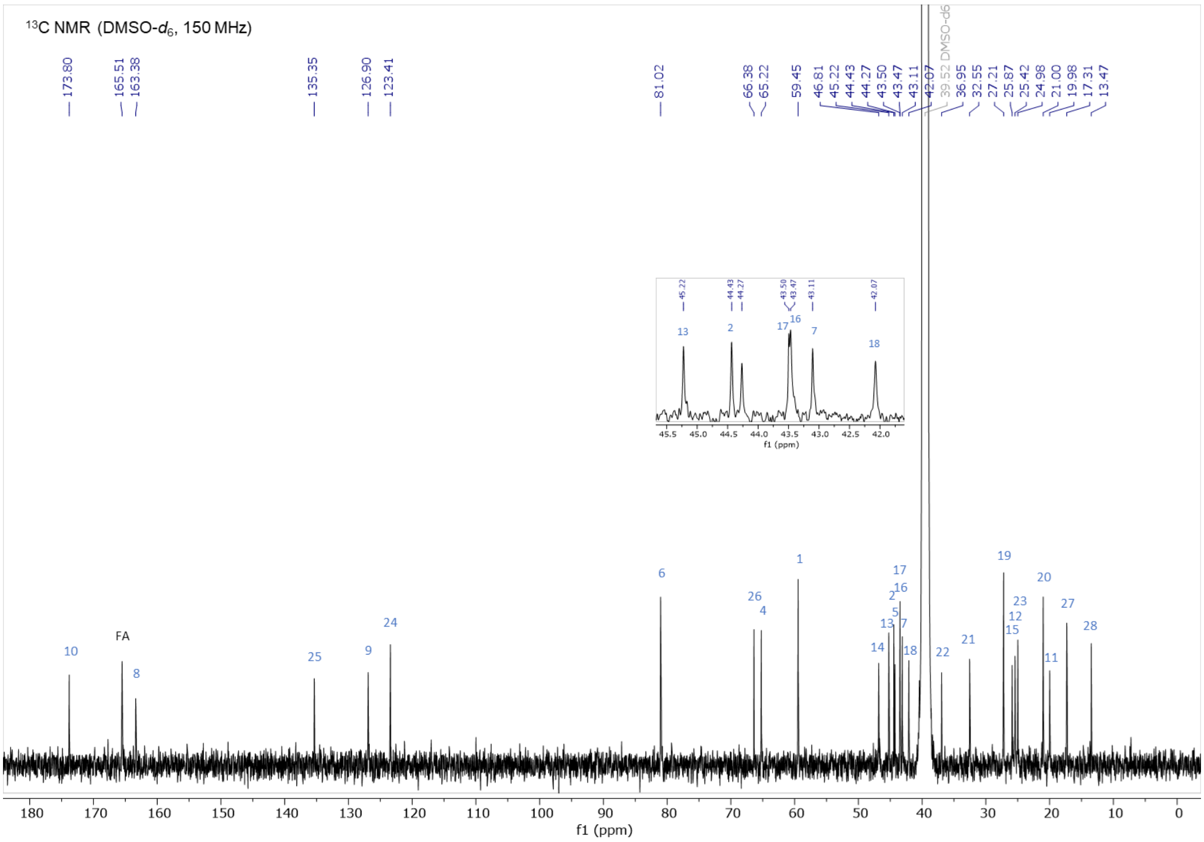

Figure S 2323. ^{13}C NMR spectrum of **2**.

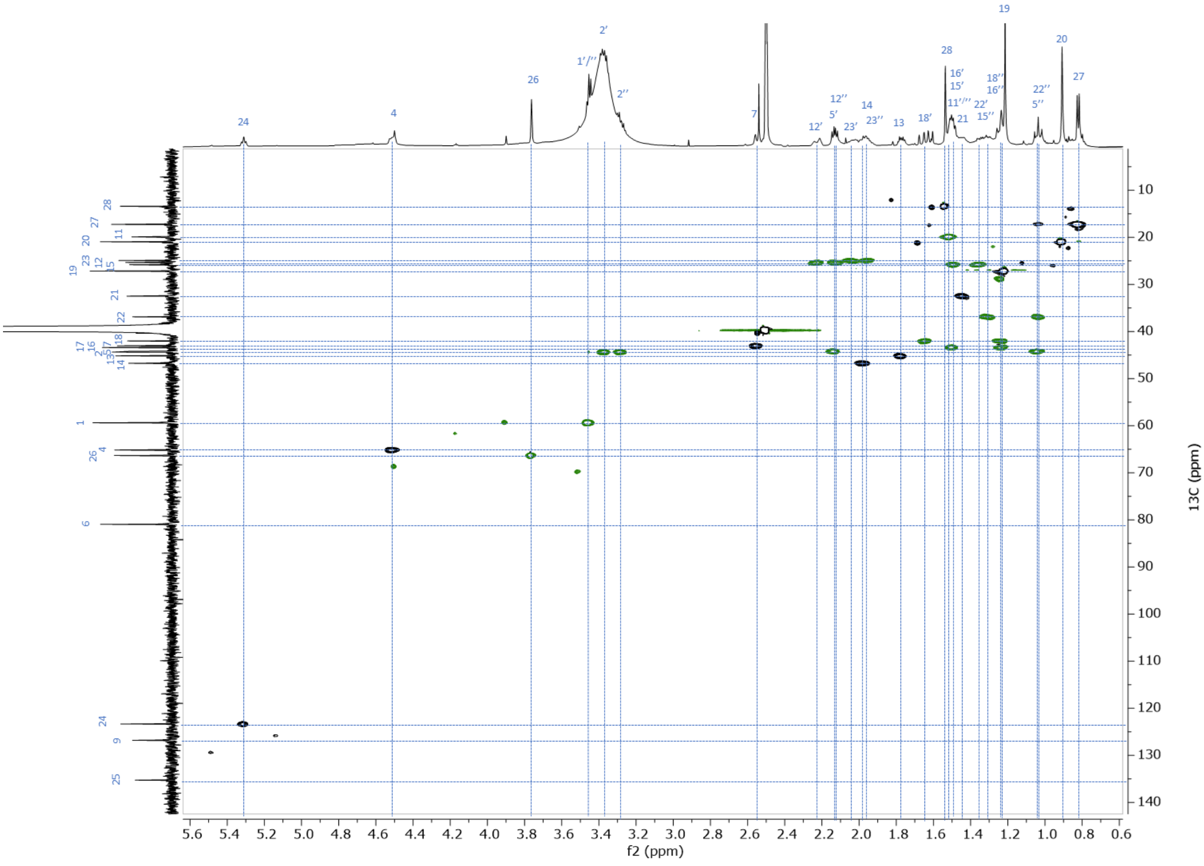

Figure S 2424. HSQC spectrum of **2**.

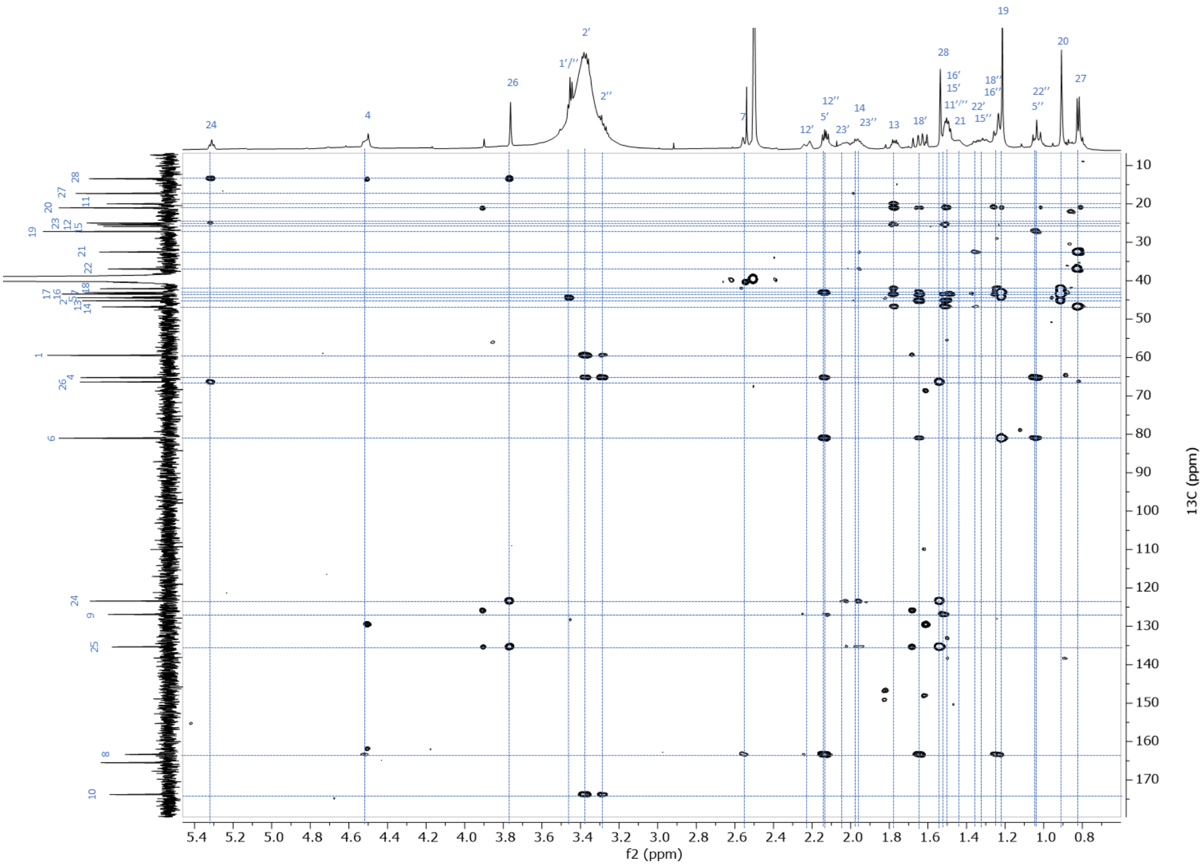

Figure S 2525. HMBC spectrum of **2**.

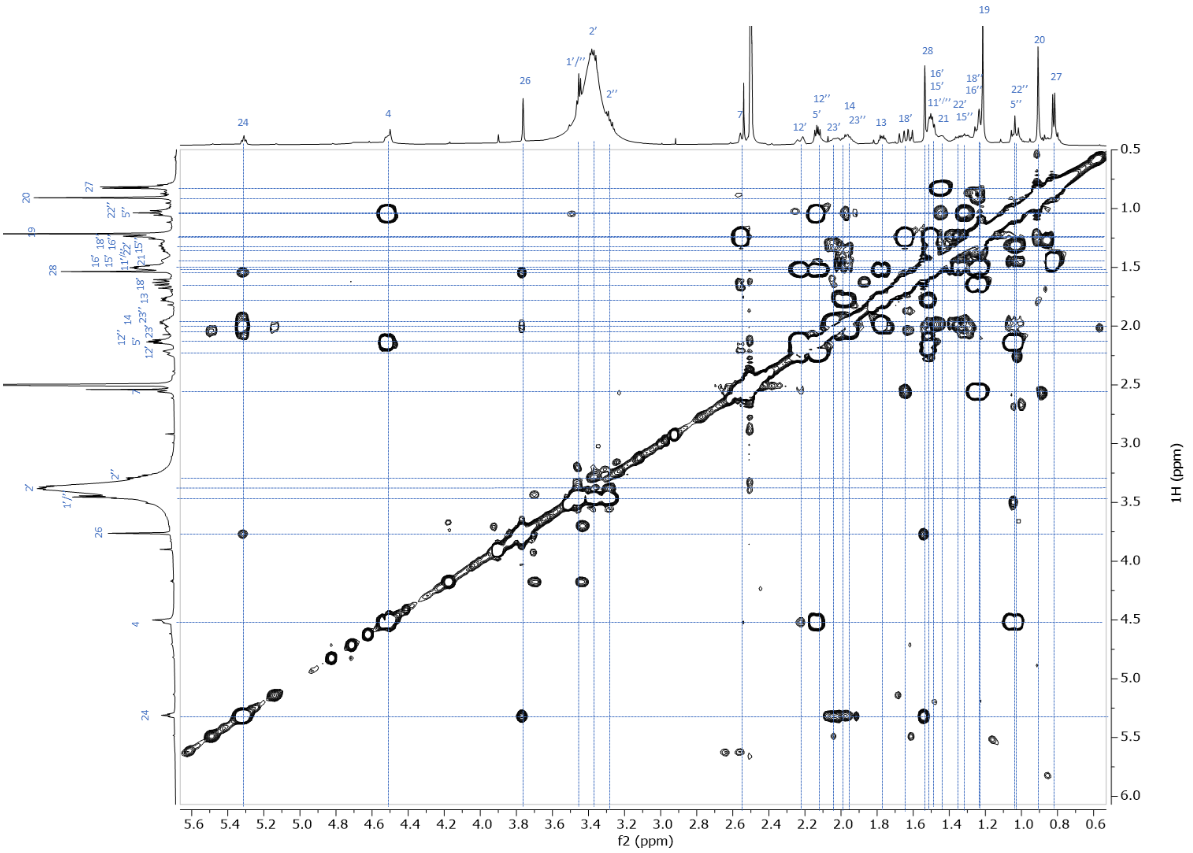

Figure S 2626. COSY spectrum of **2**.

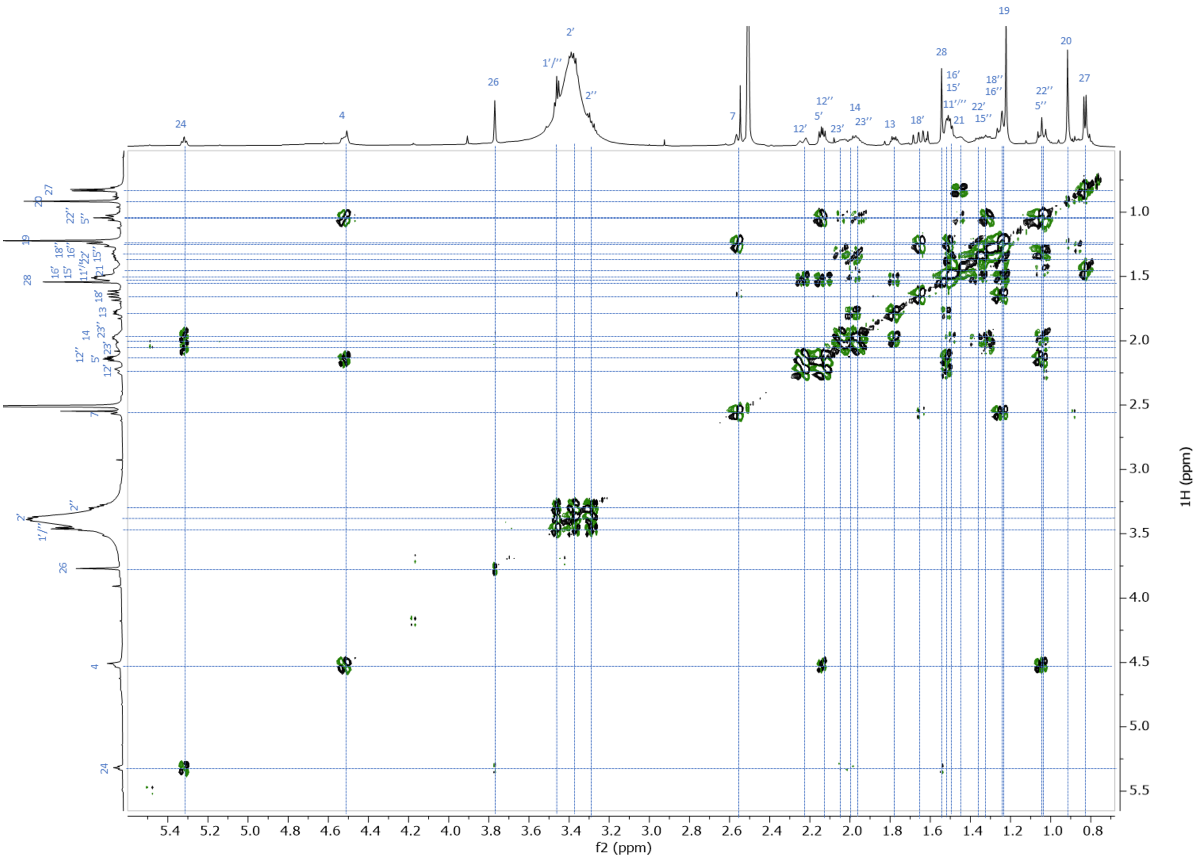

Figure S 2727. DQF-COSY spectrum of **2**.

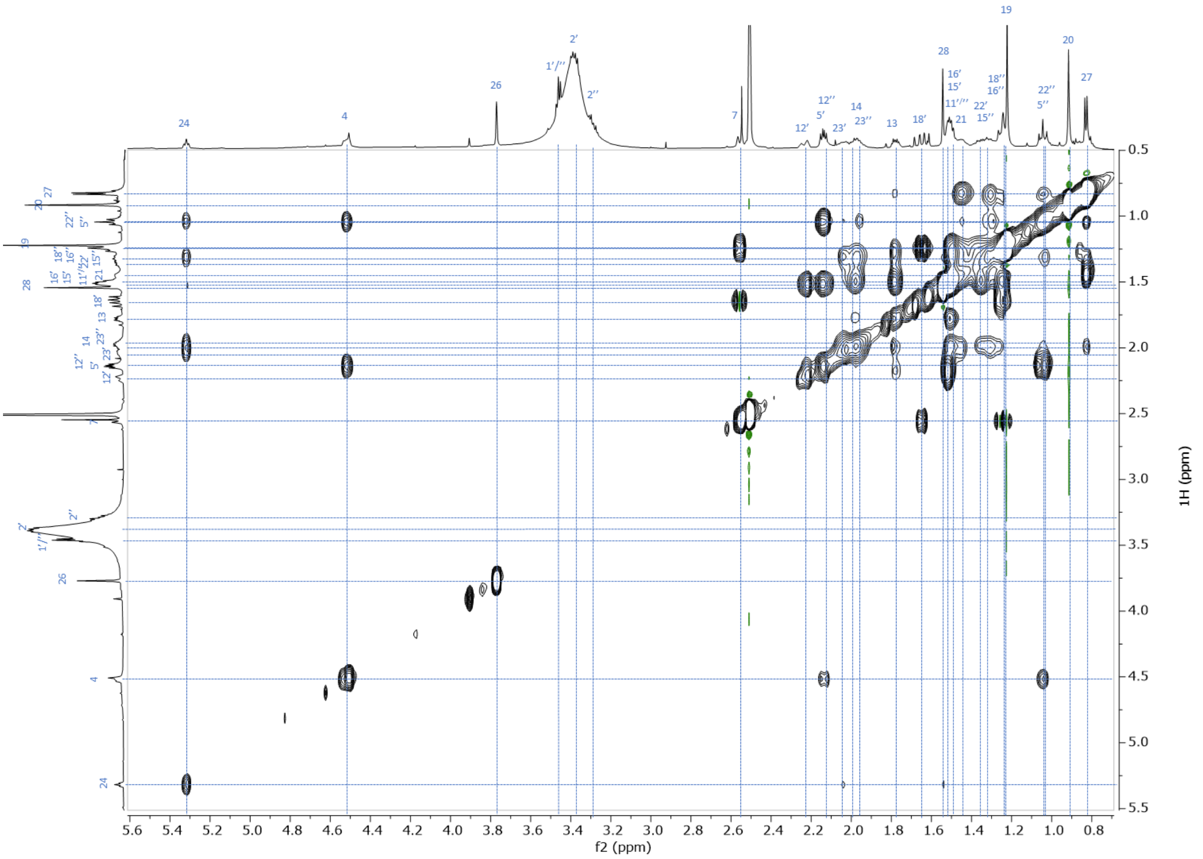

Figure S 2828. TCOSY ((DIPSI2) 60ms) spectrum of **2**.

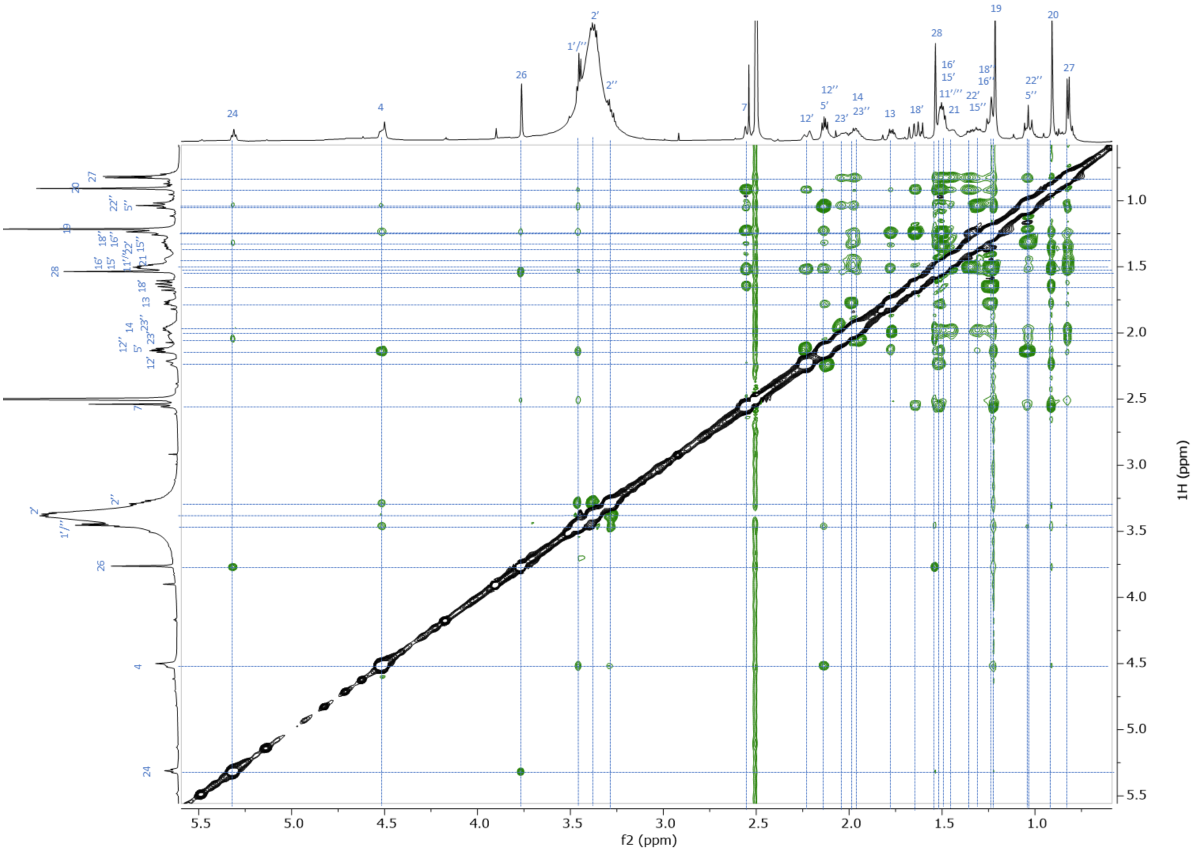

Figure S 2929. ROESY spectrum of **2**.

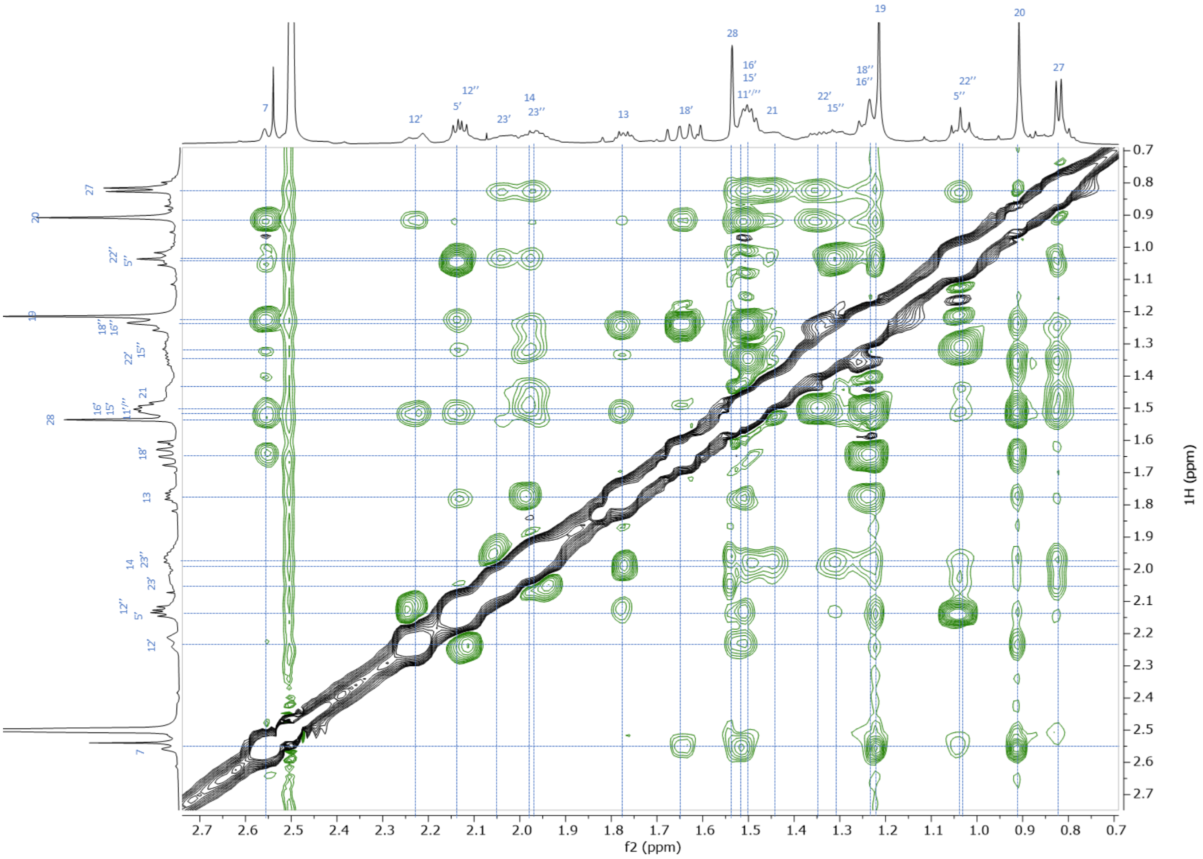

Figure S 3030. ROESY (Expanded) spectrum of **2**.

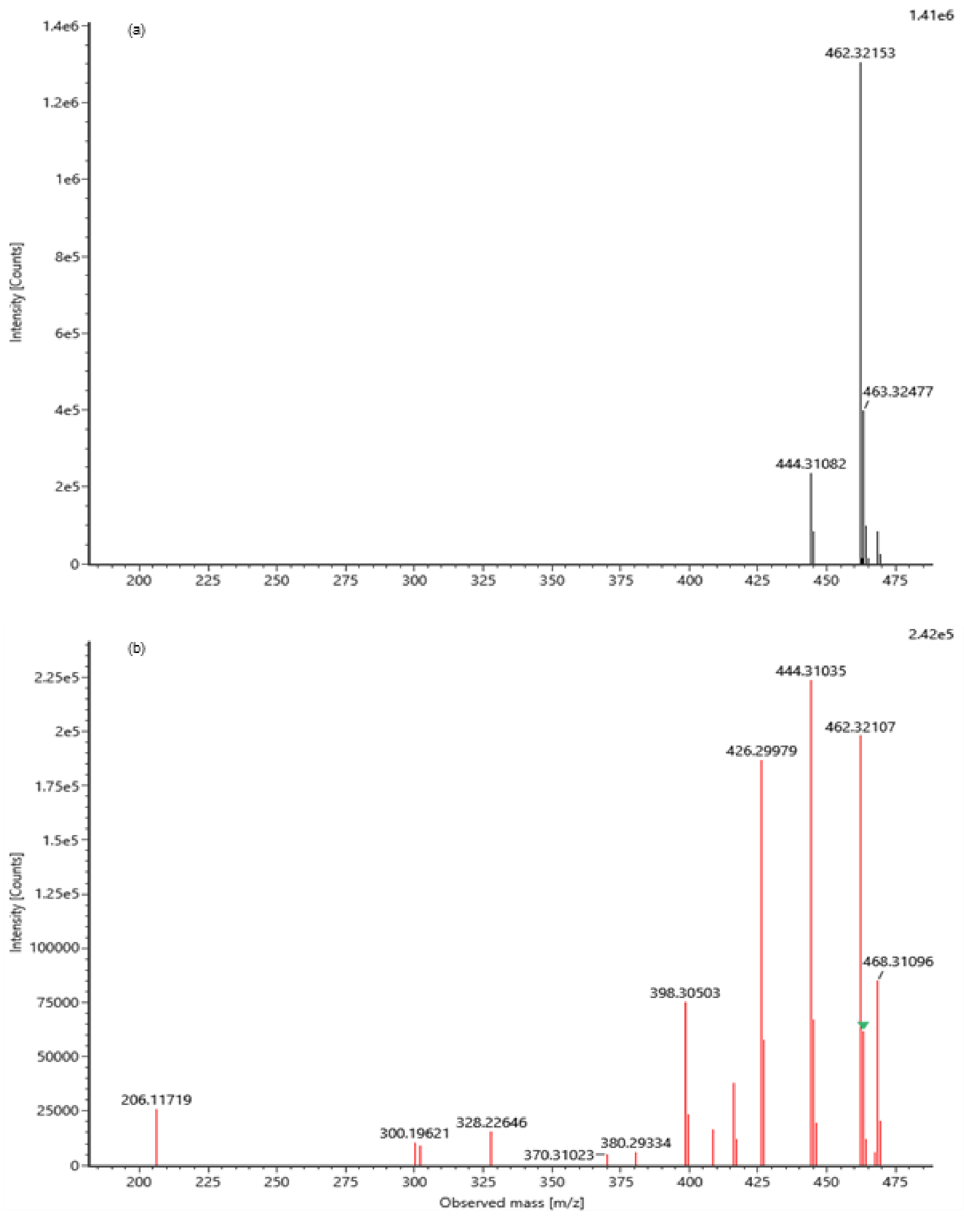

Figure S 3131. Low-collision (top) and High-collision (bottom) energy mass spectra of **3** in ESI$^+$ mode.

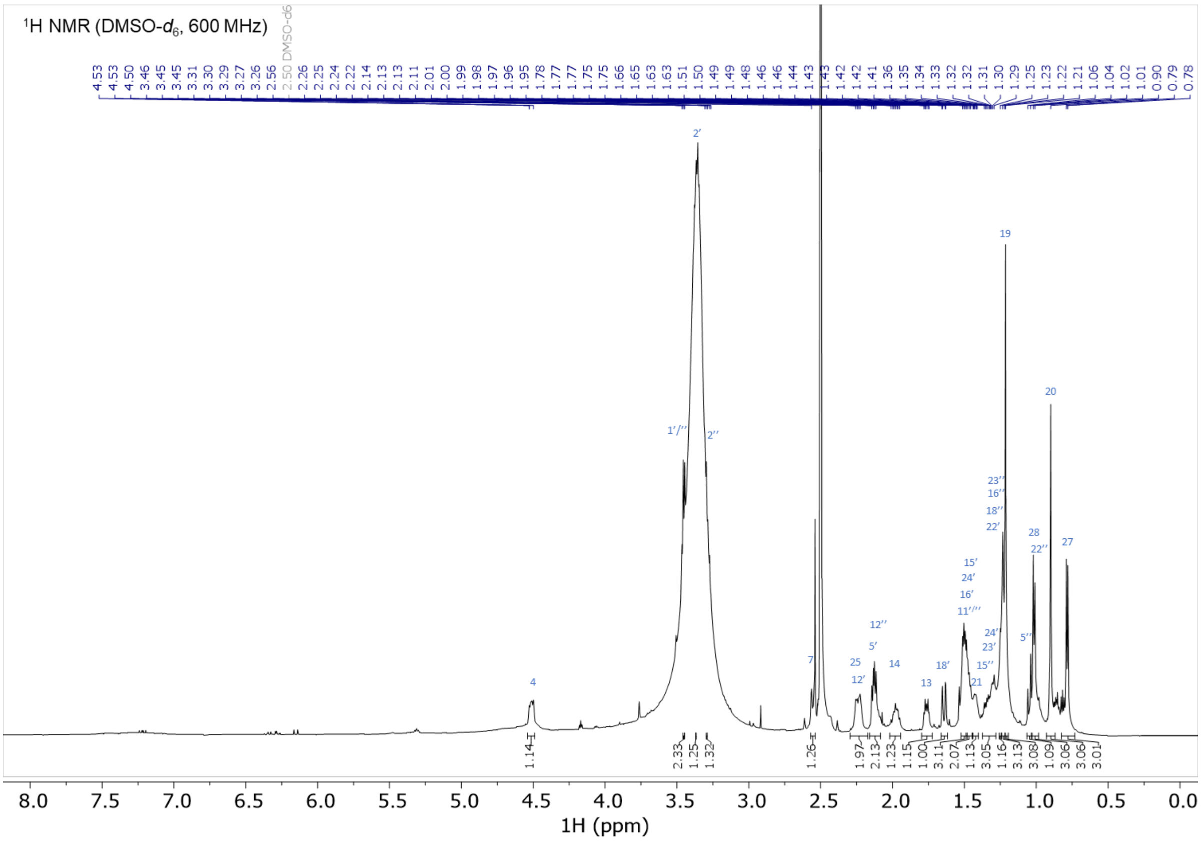

Figure S 3232. ^{1}H NMR spectrum of **3**.

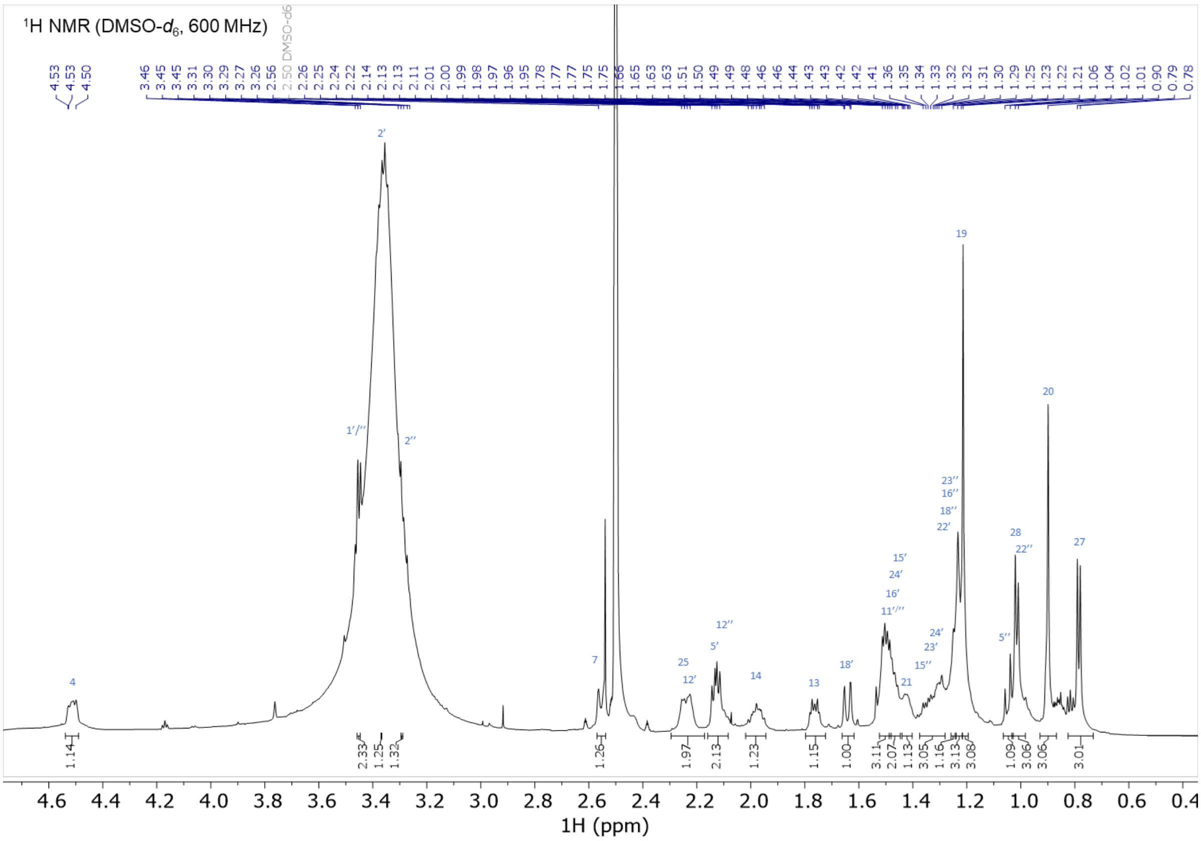

Figure S 3333. ^{1}H NMR (Expanded) spectrum of **3**.

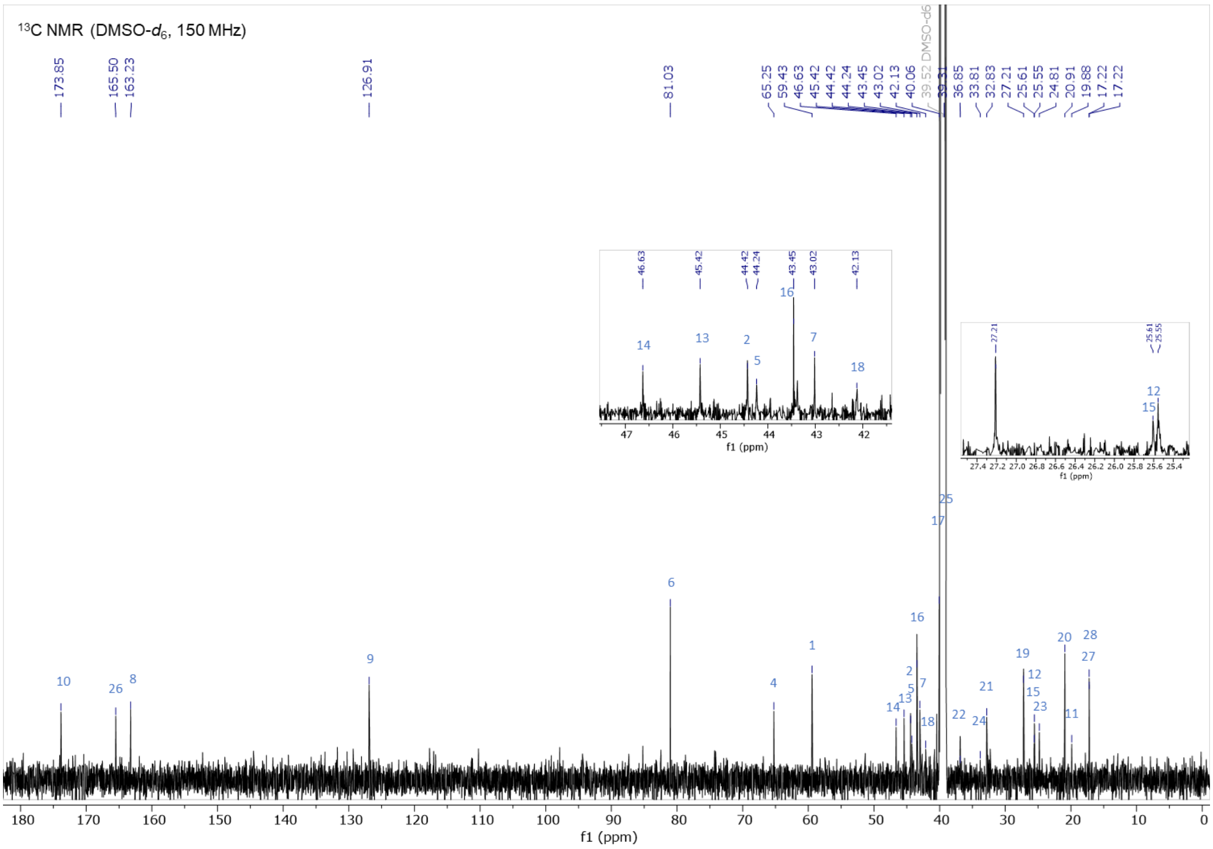

Figure S 3434. ^{13}C NMR spectrum of **3**.

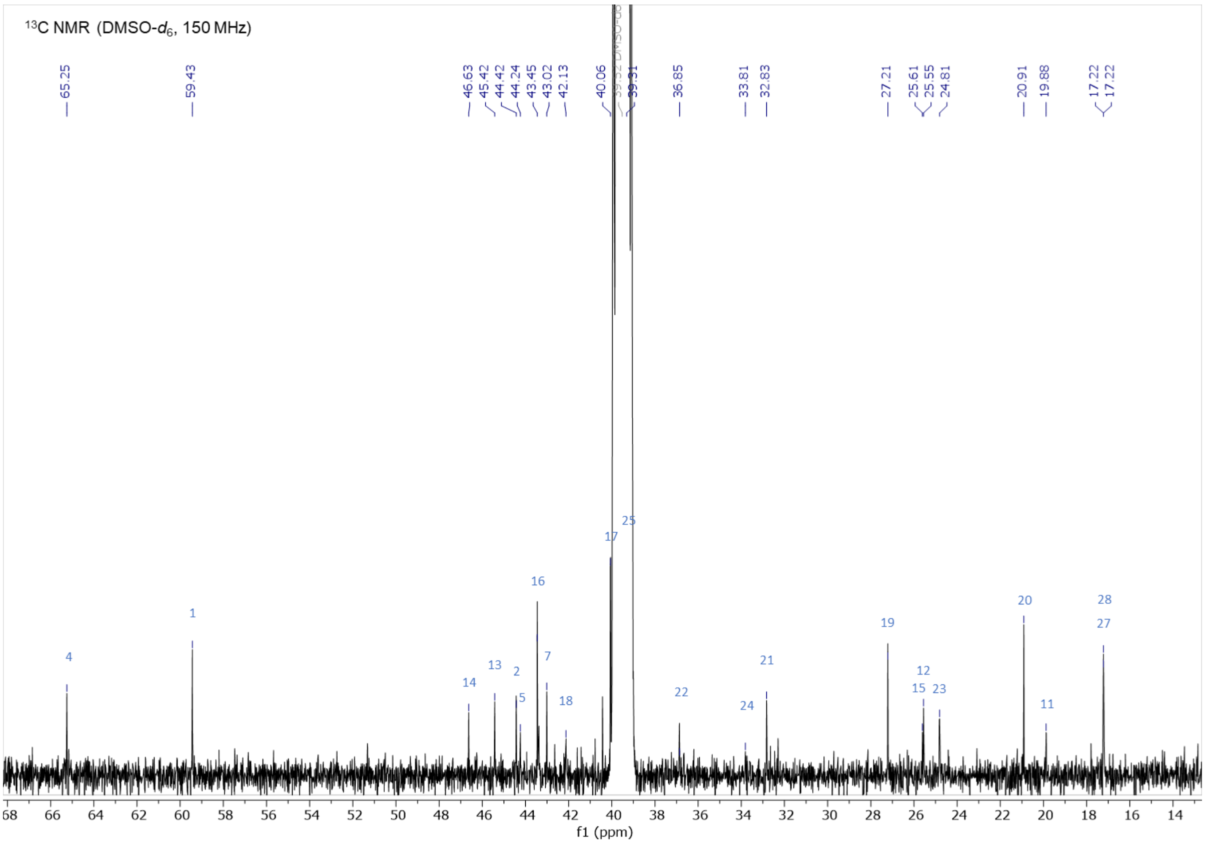

Figure S 3535. ^{13}C NMR (Expanded) spectrum of **3**.

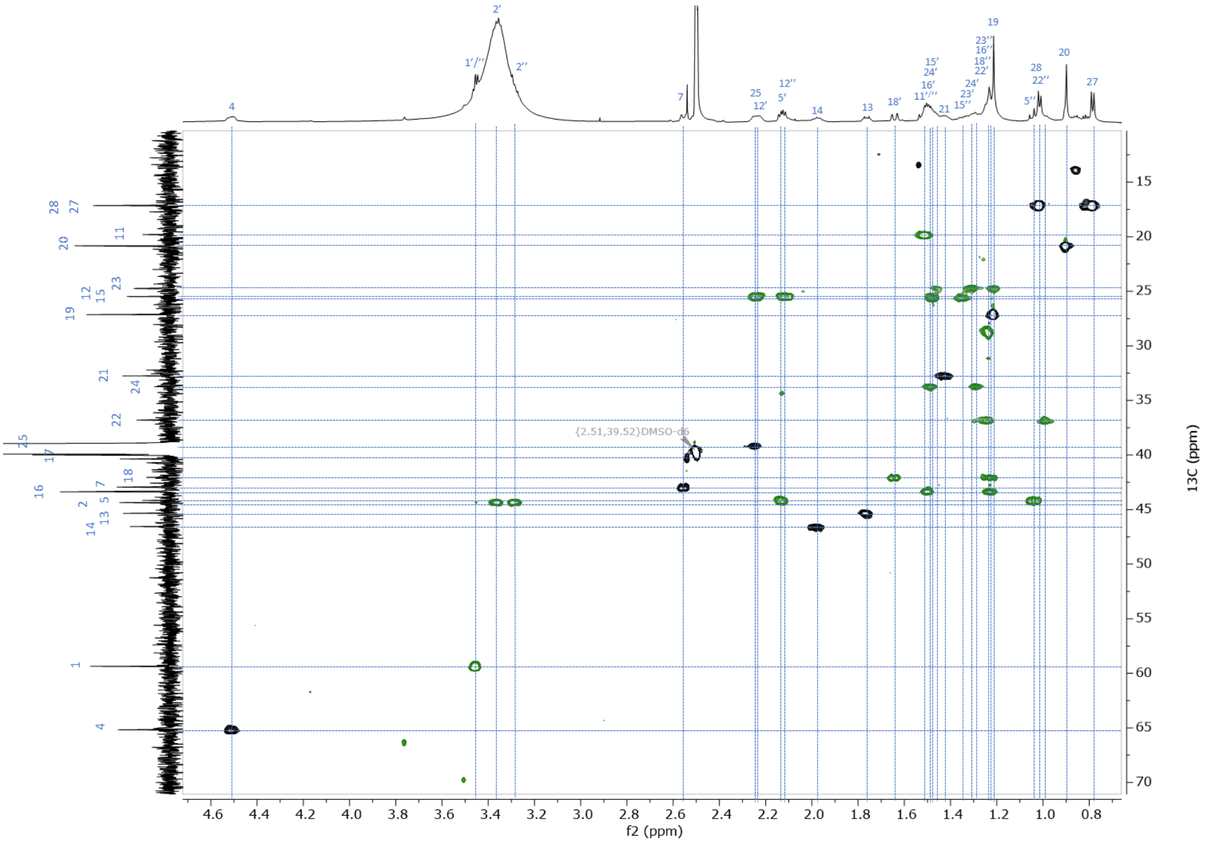

Figure S 3636. HSQC spectrum of **3**.

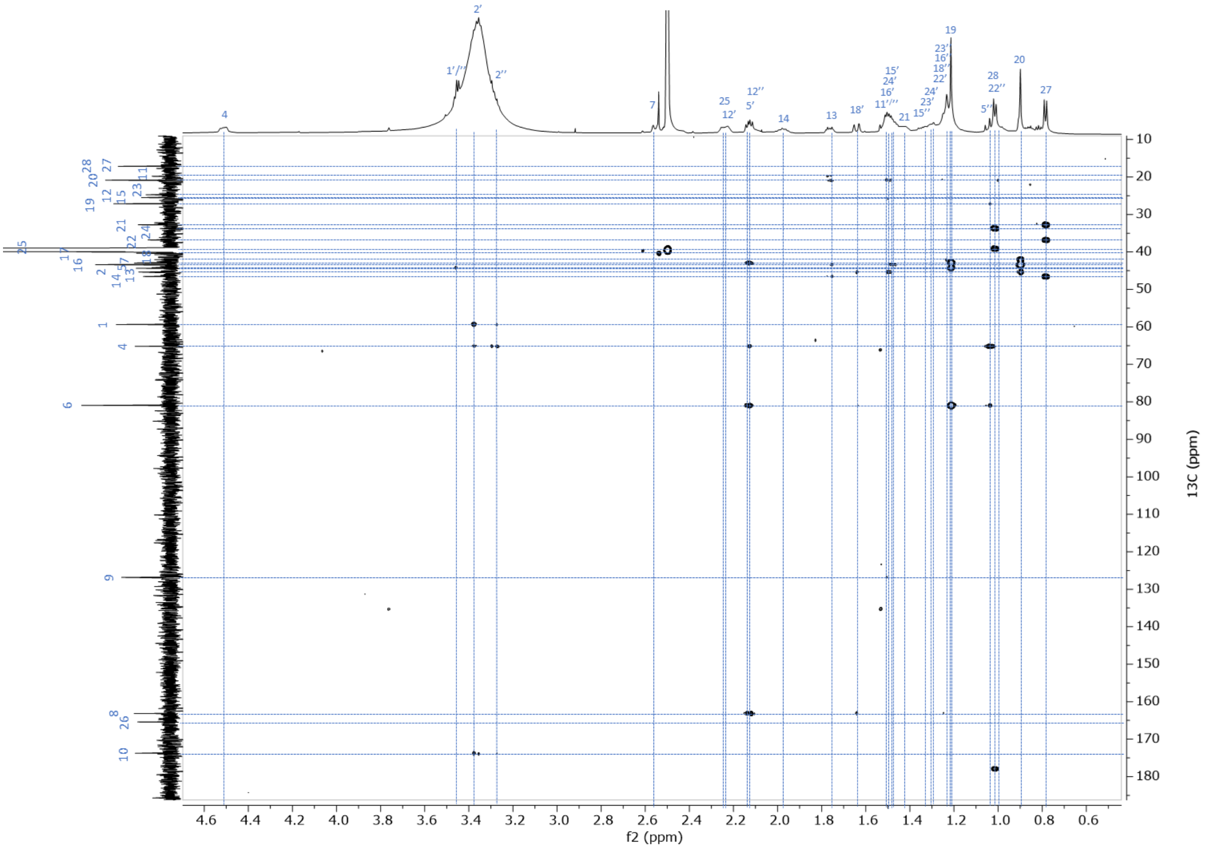

Figure S 3737. HMBC of spectrum **3**.

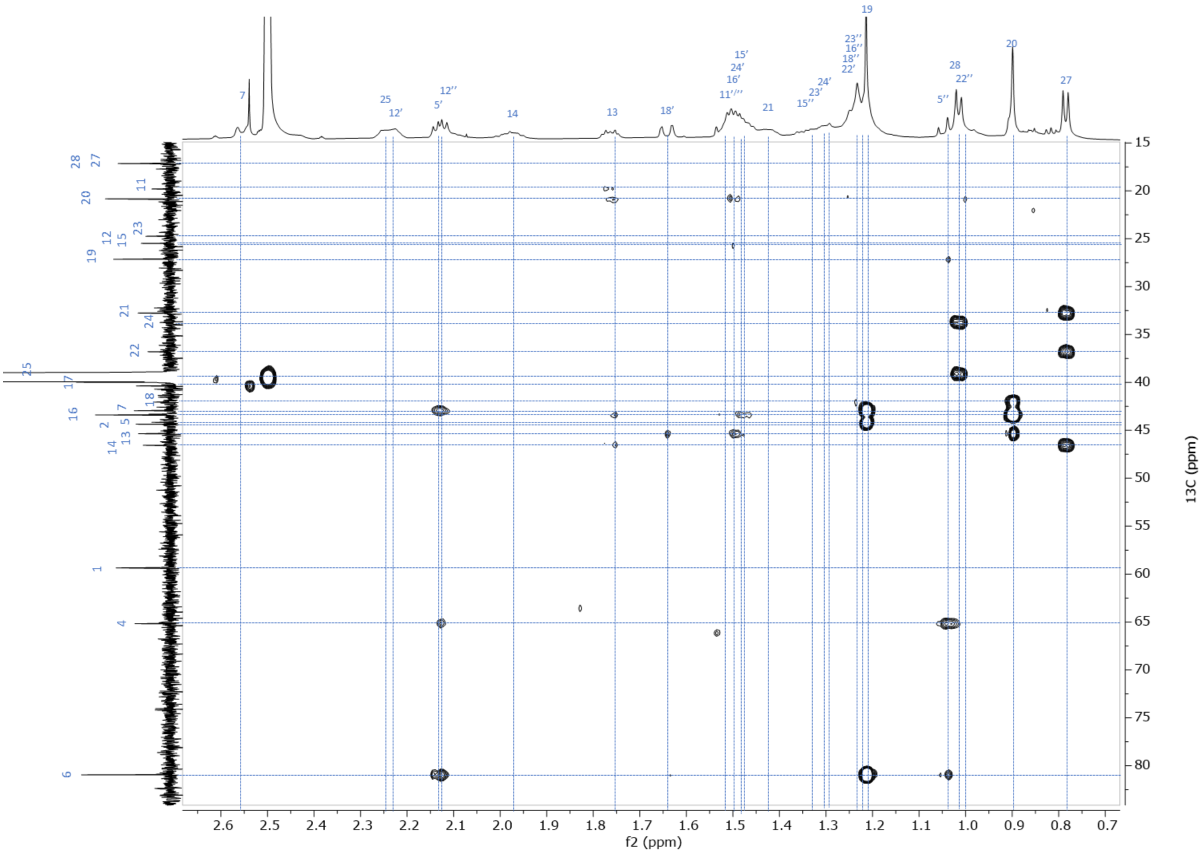

Figure S 3838. HMBC (Expanded) spectrum of **3**.

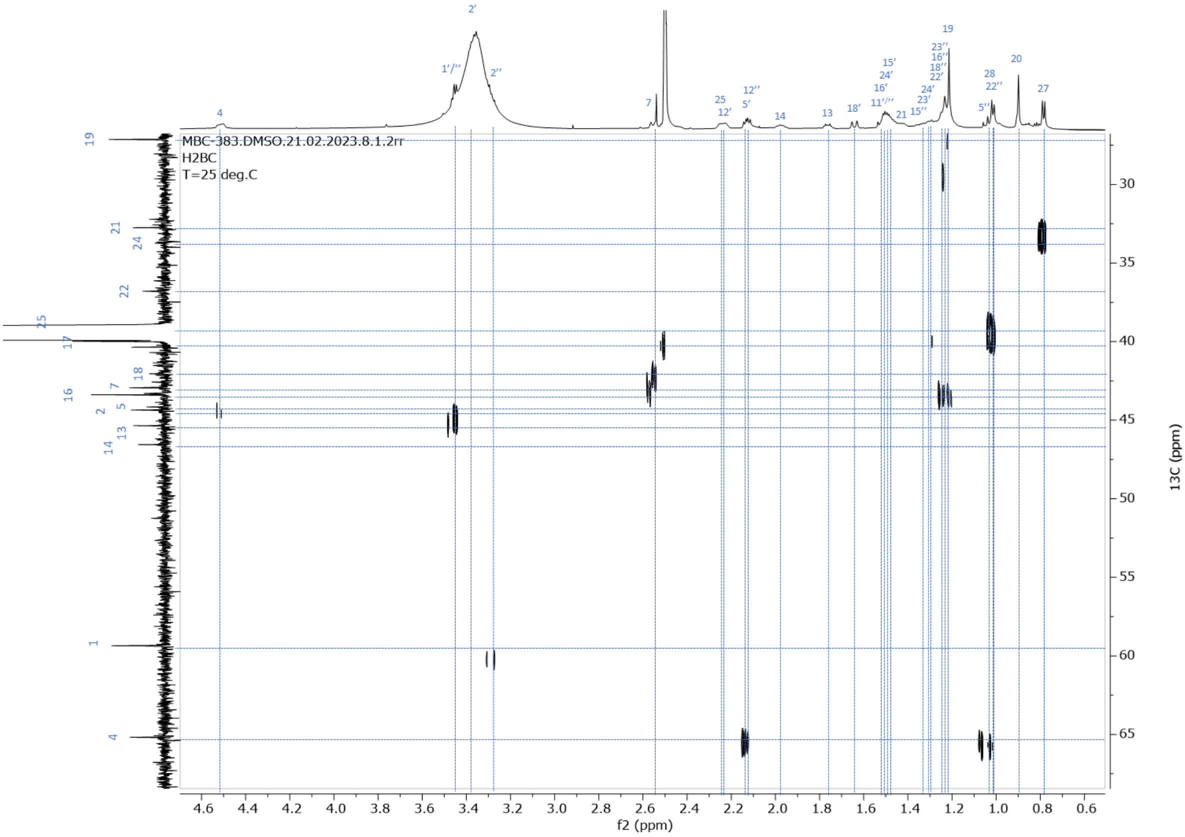

Figure S 3939. H2BC spectrum of **3**.

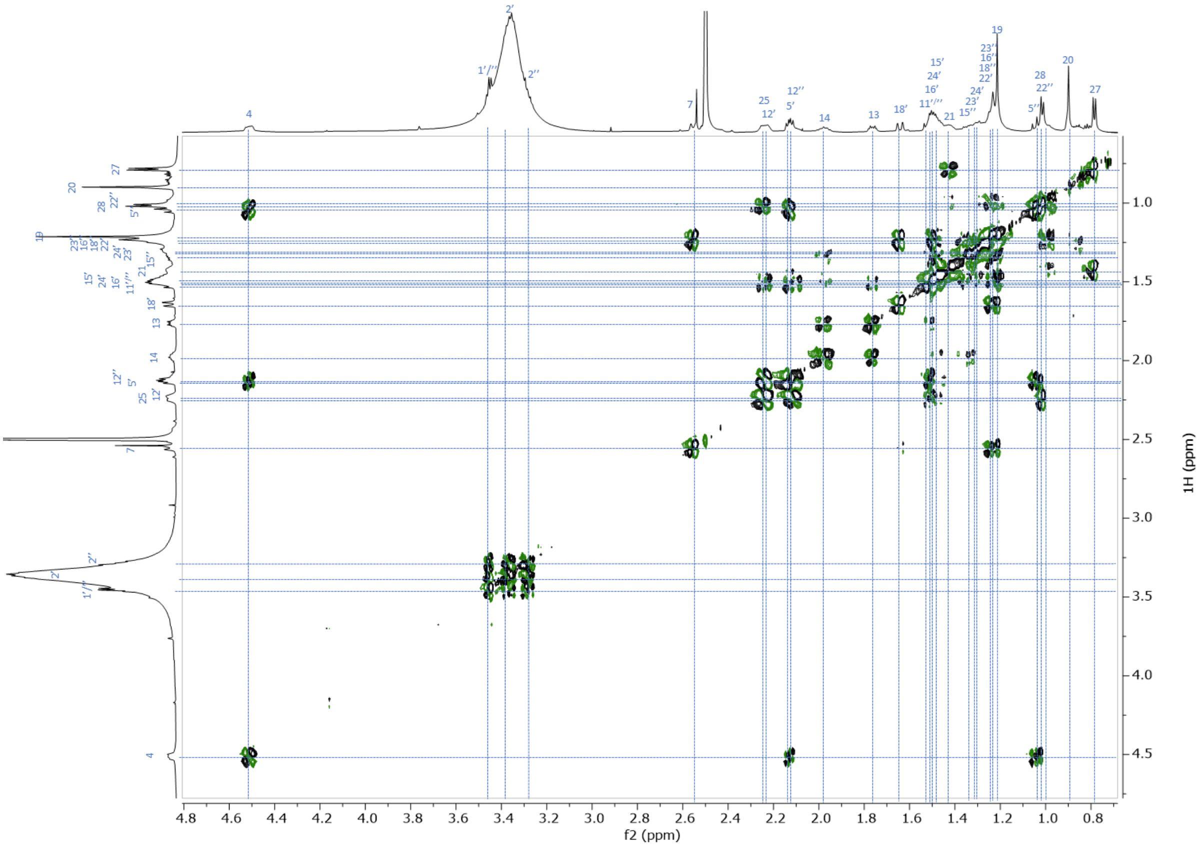

Figure S 4040. DQF-COSY spectrum of **3**.

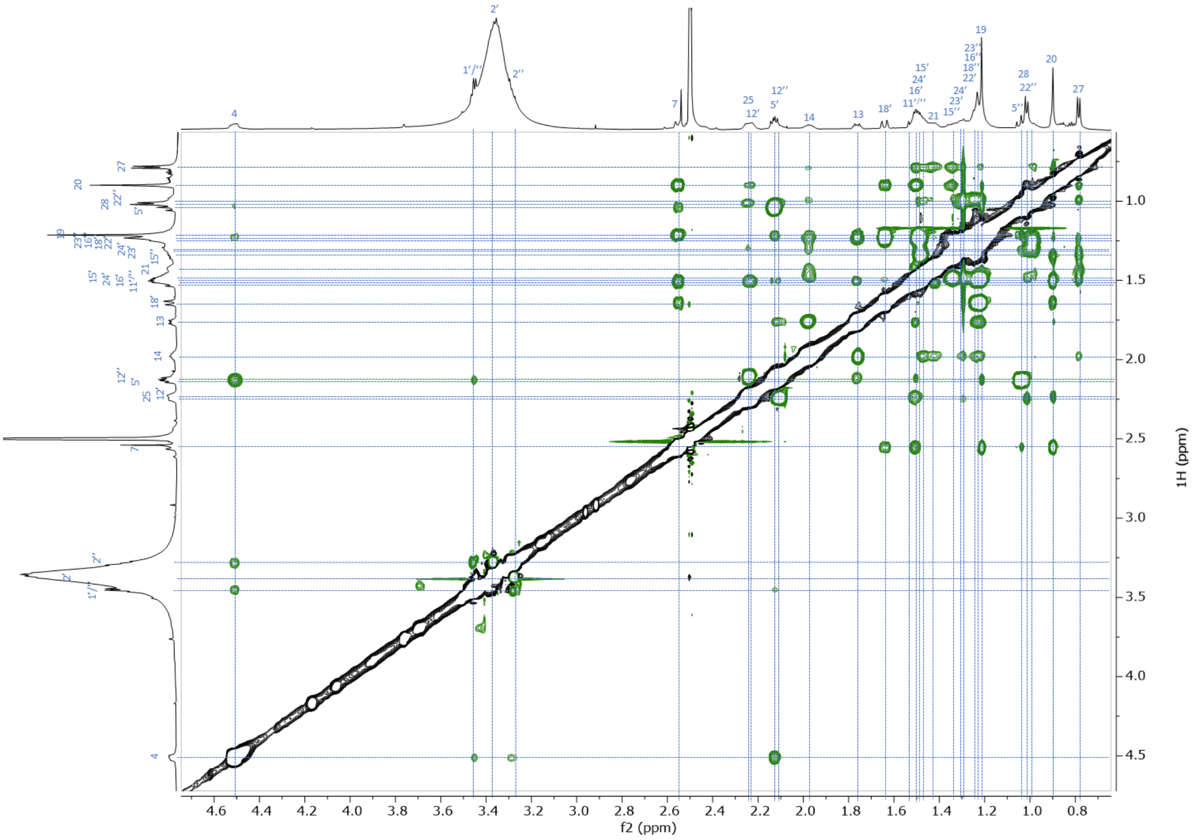

Figure S 4141. ROESY spectrum of **3**.

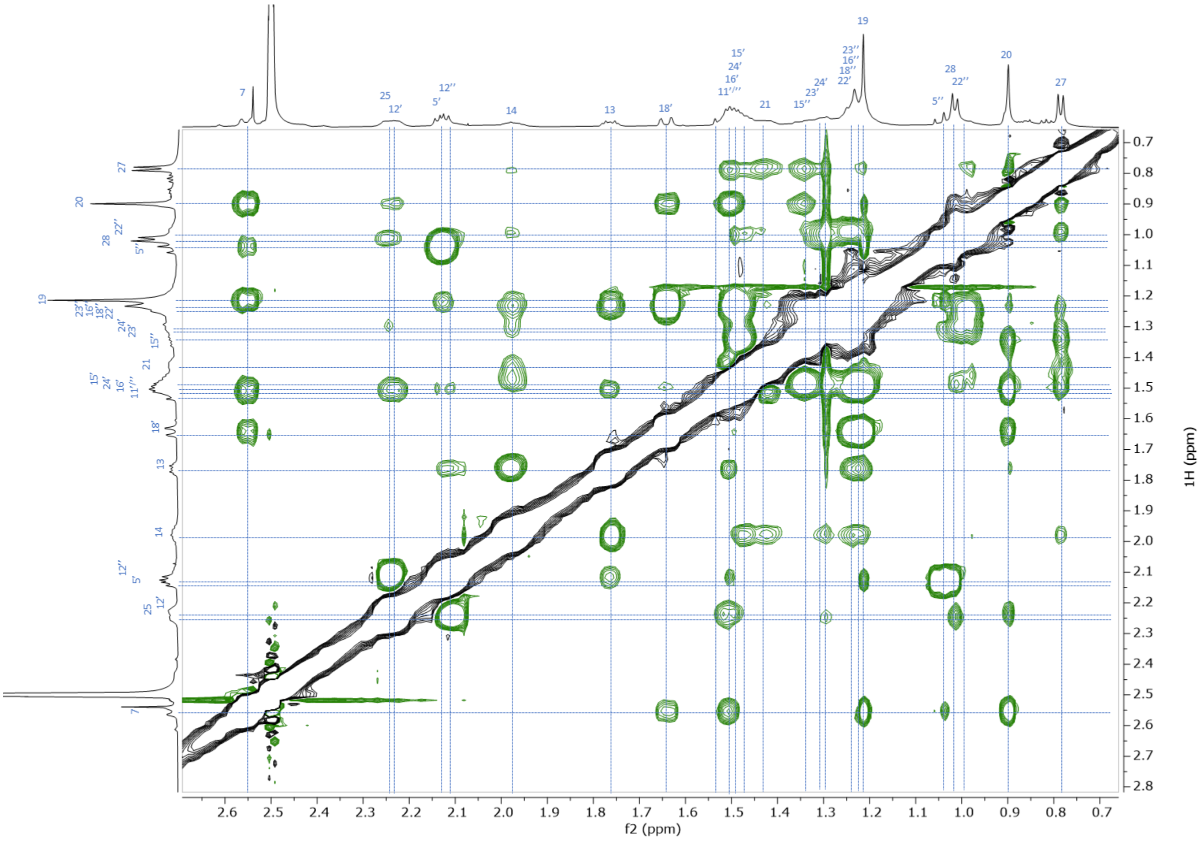

Figure S 4242. ROESY (Expanded) spectrum of **3**.

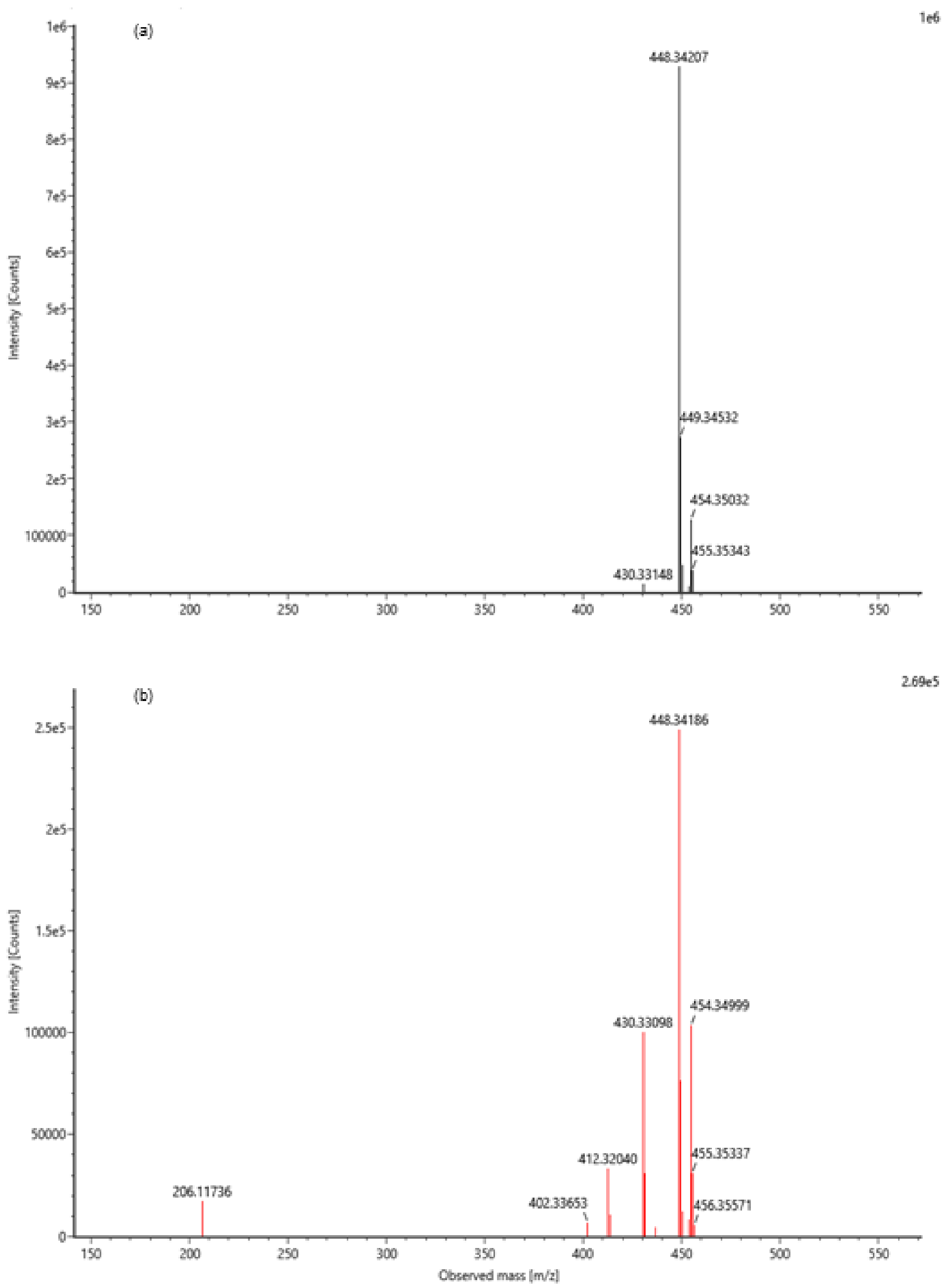

Figure S 4343. Low-collision (top) and High-collision (bottom) energy mass spectra of **4** in ESI$^+$ mode.

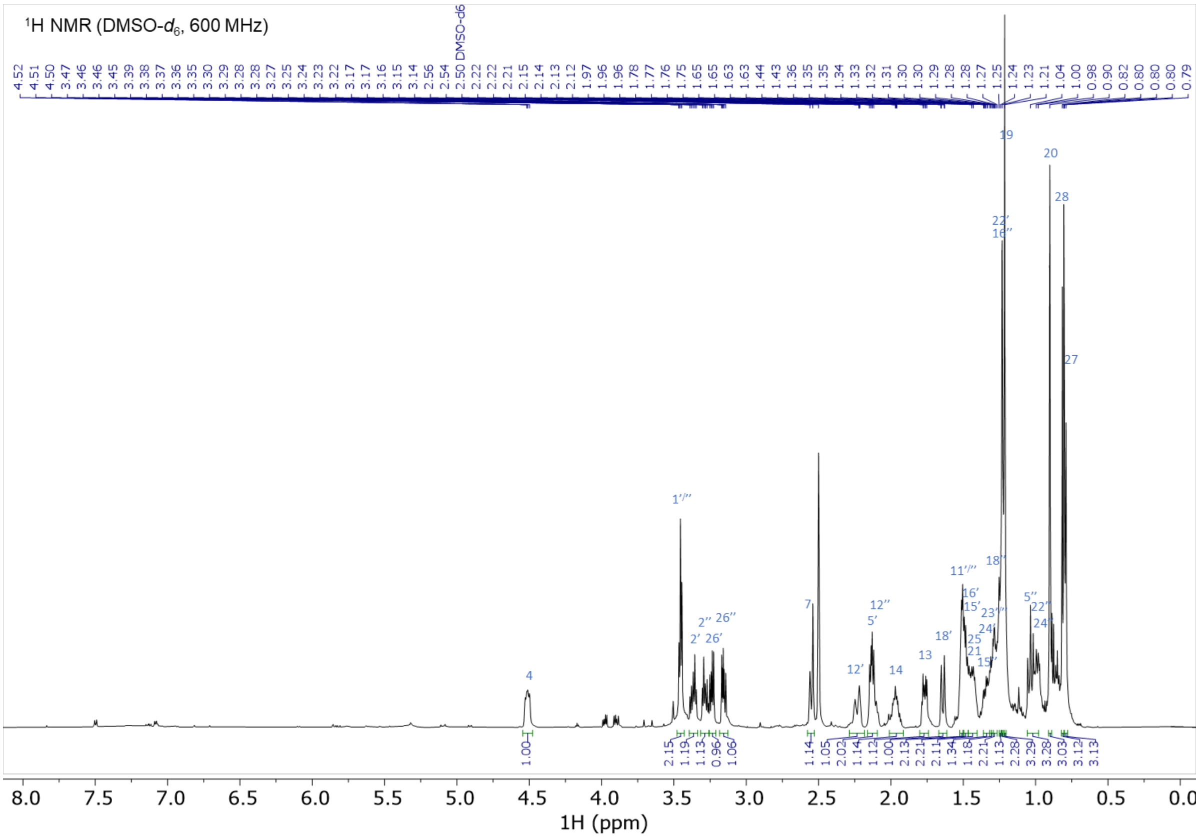

Figure S 4444. ^{1}H NMR spectrum of **4**.

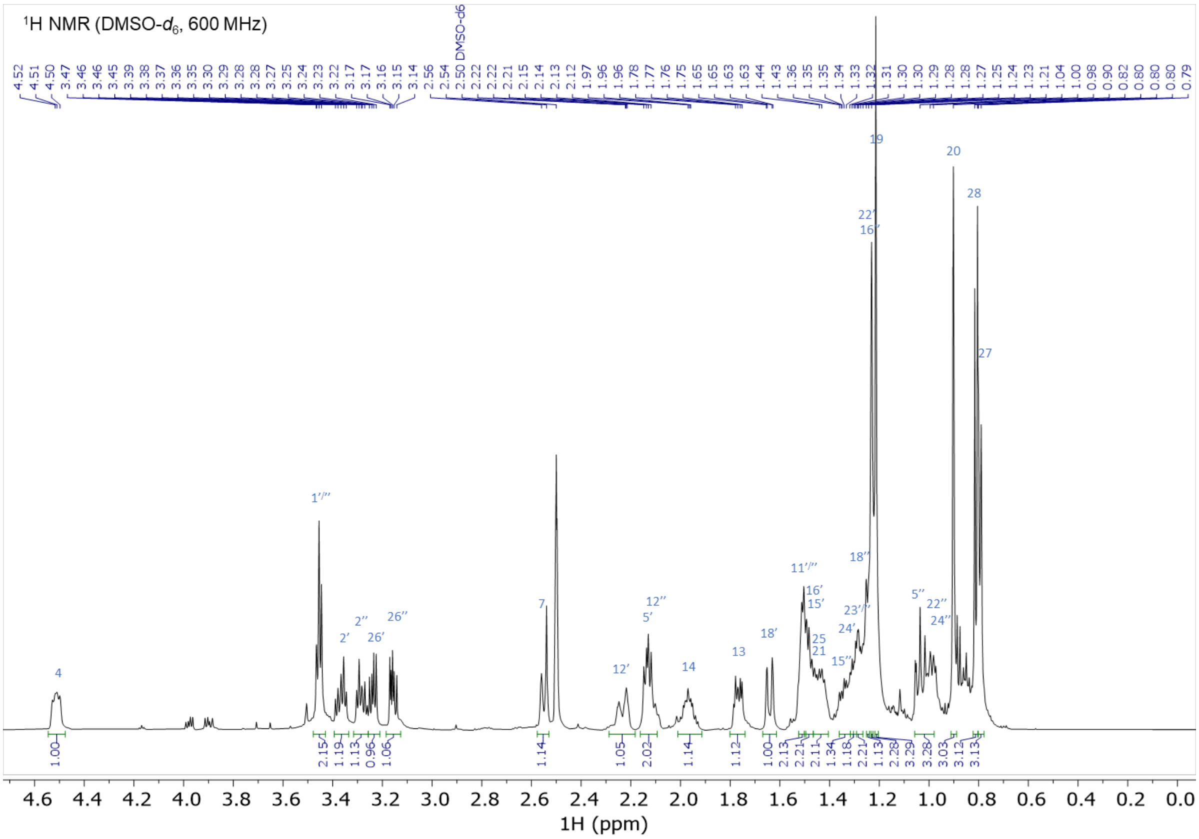

Figure S 4545. ^{1}H NMR (Expanded) spectrum of **4**.

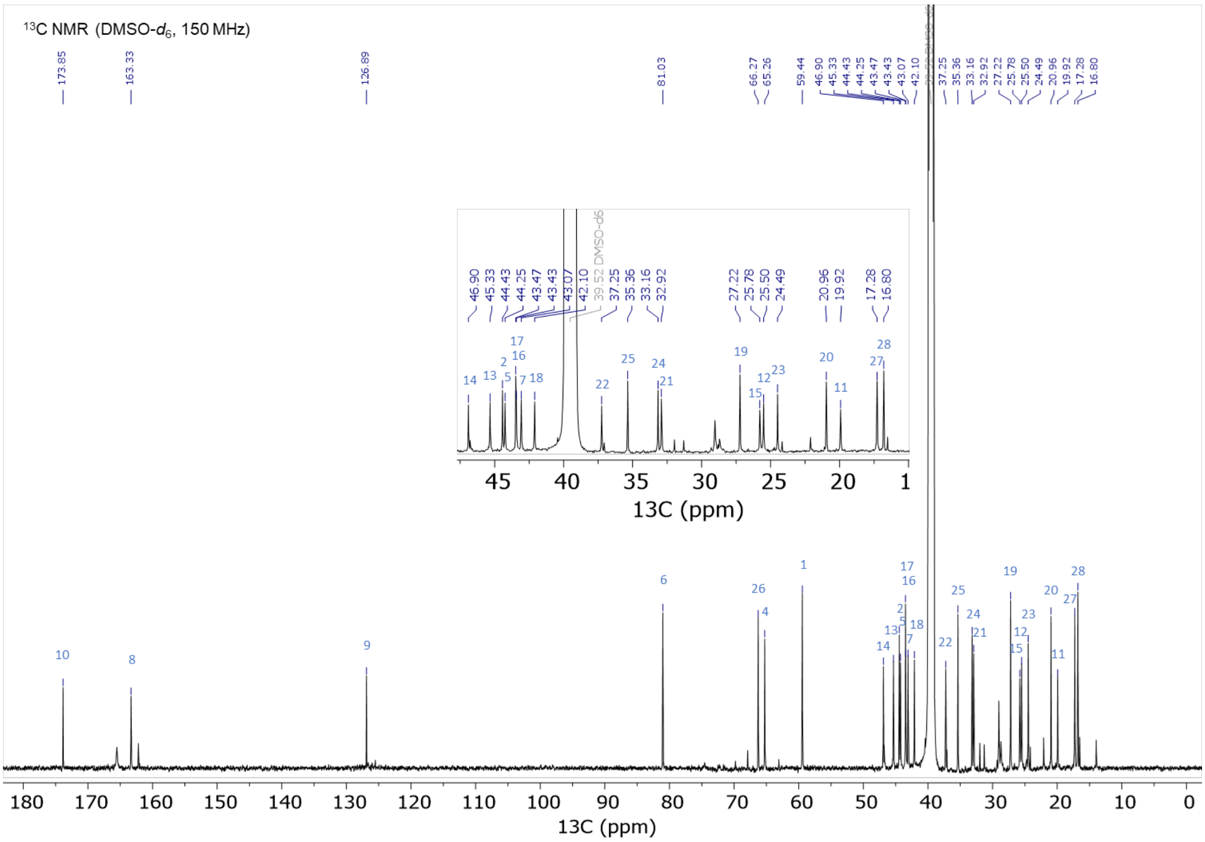

Figure S 4646. ^{13}C NMR spectrum of **4**.

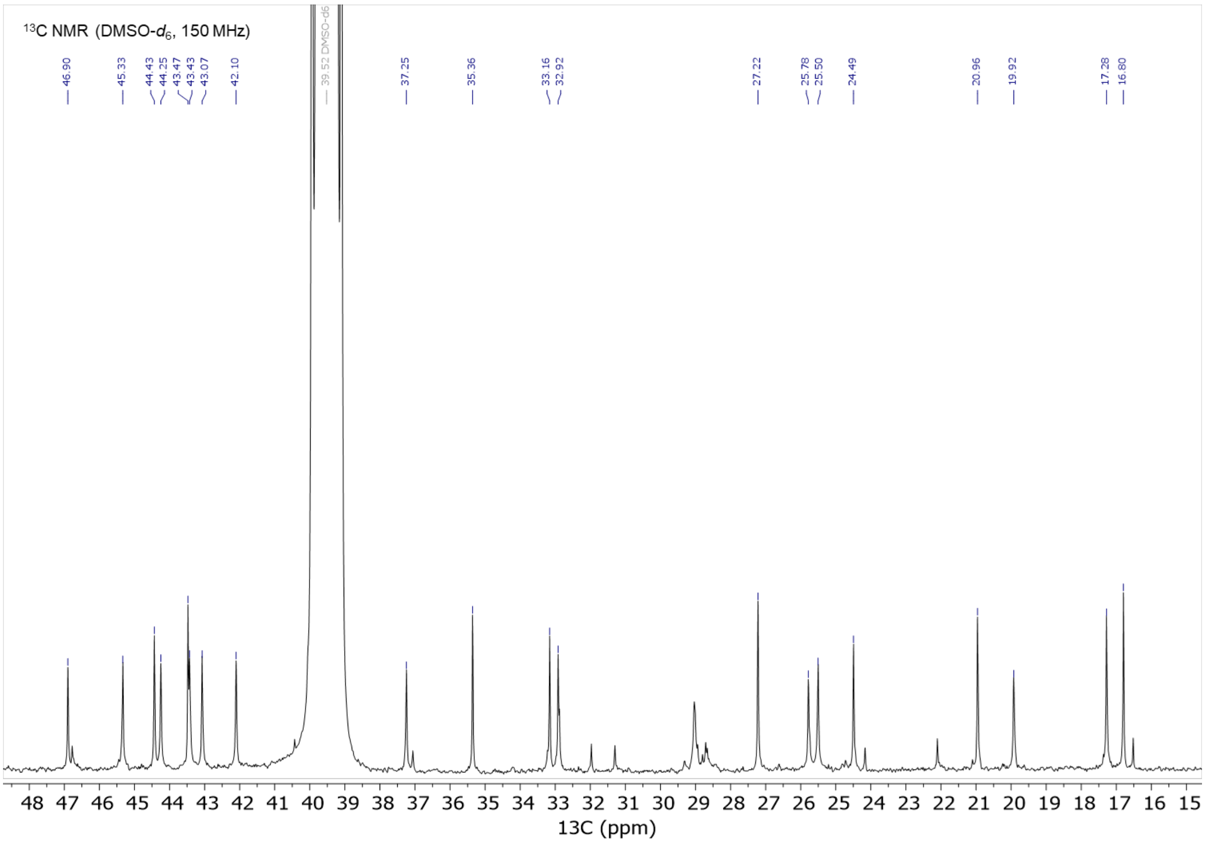

Figure S 4747. ^{13}C NMR (Expanded) spectrum of **4**.

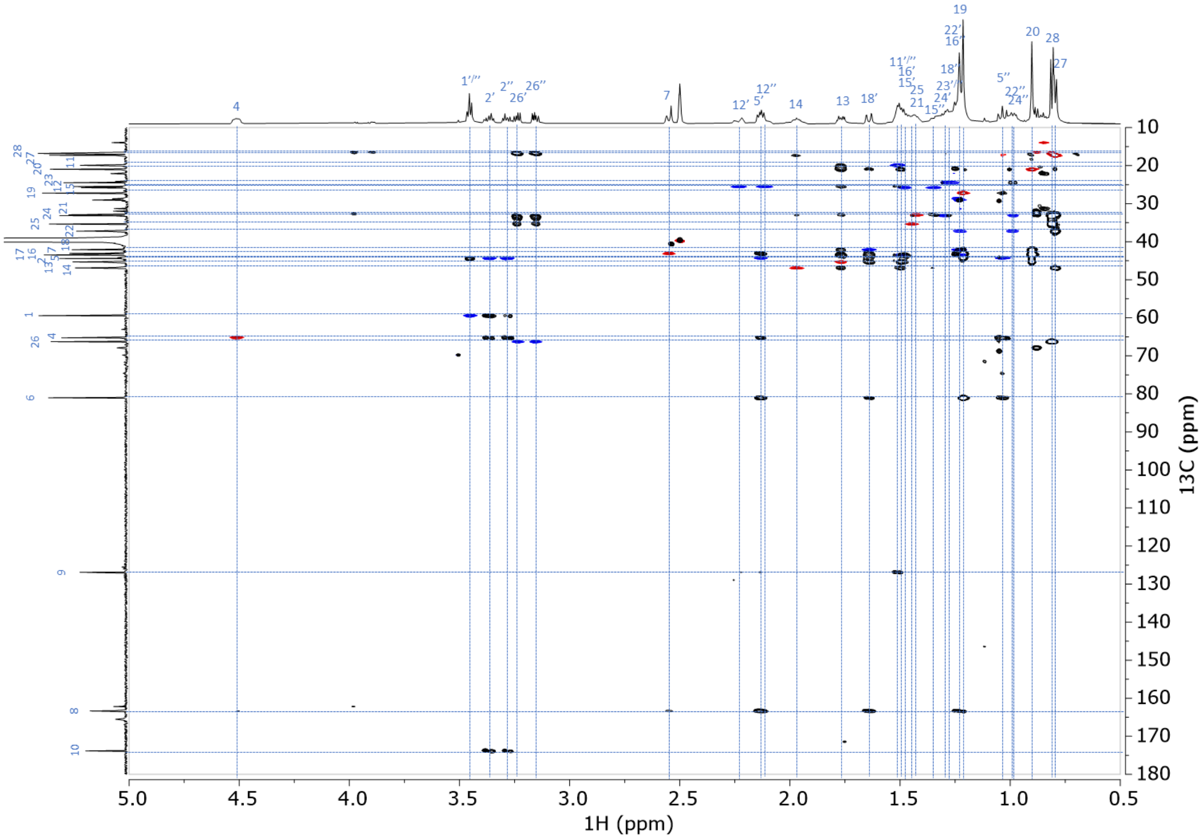

Figure S 4848. HSQC+ HMBC spectrum of **4**.

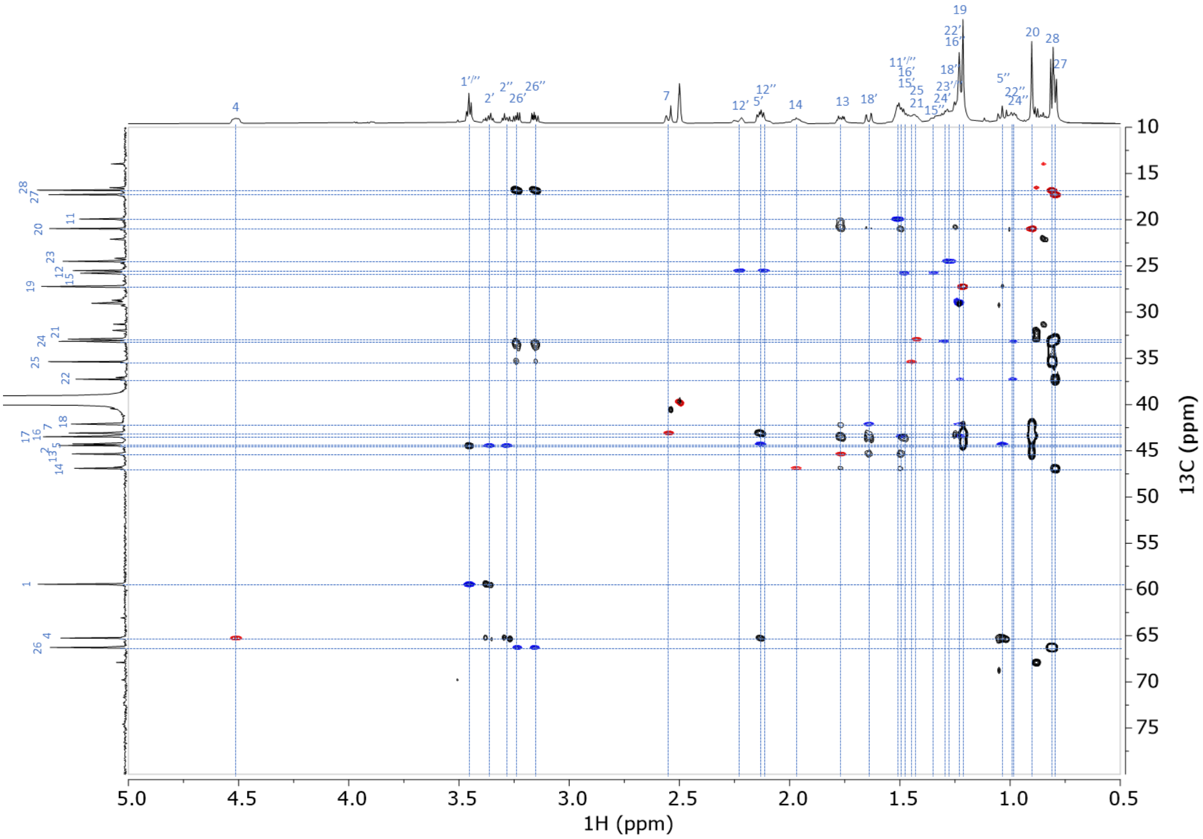

Figure S 4949. HSQC+ HMBC (Expanded) spectrum of **4**.

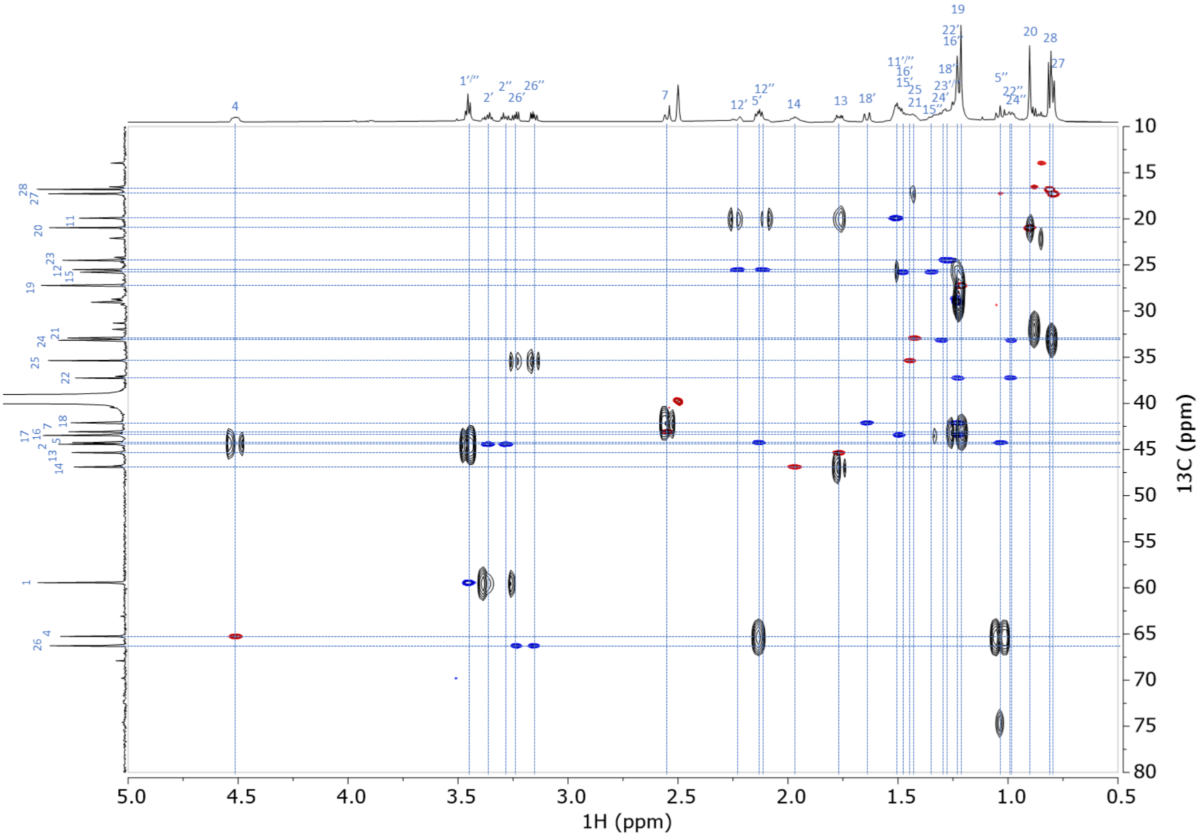

Figure S 5050. HSQC+ H2BC spectrum of **4**.

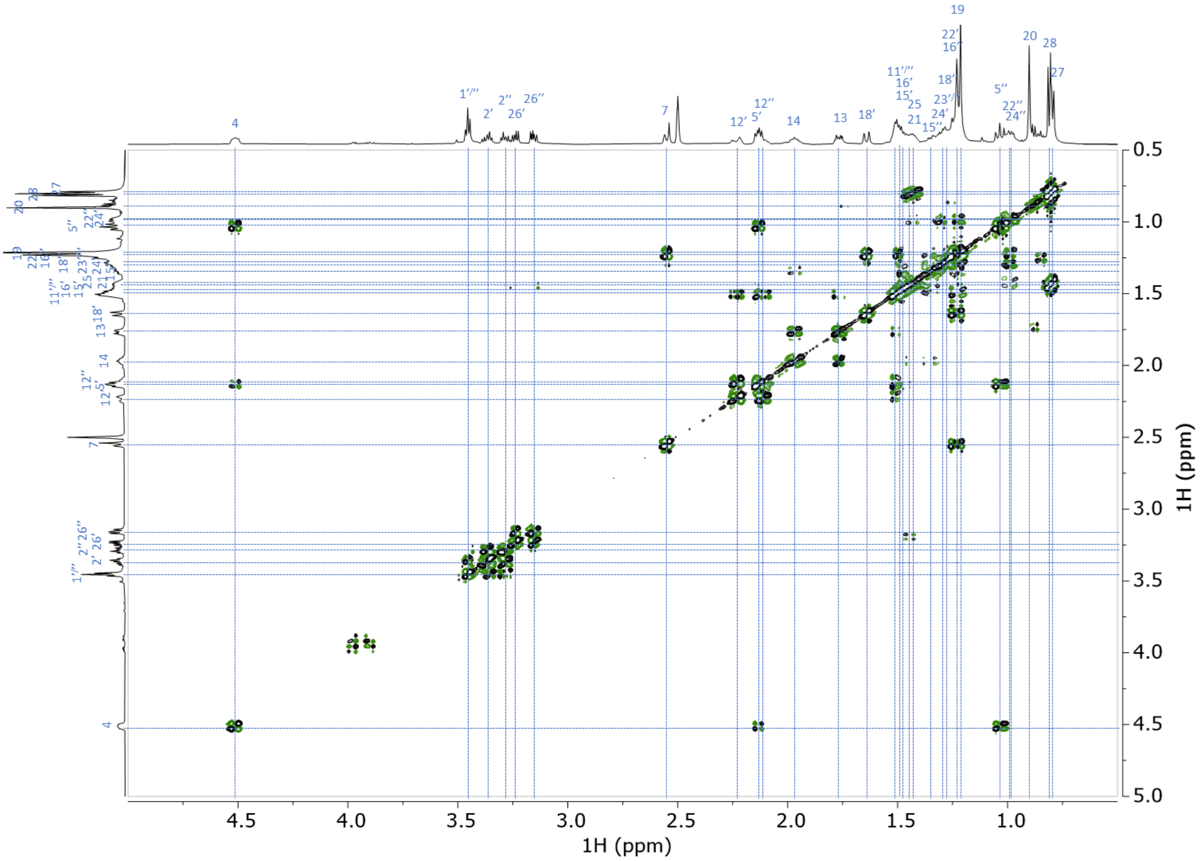

Figure S 5151. DQF-COSY spectrum of **4**.

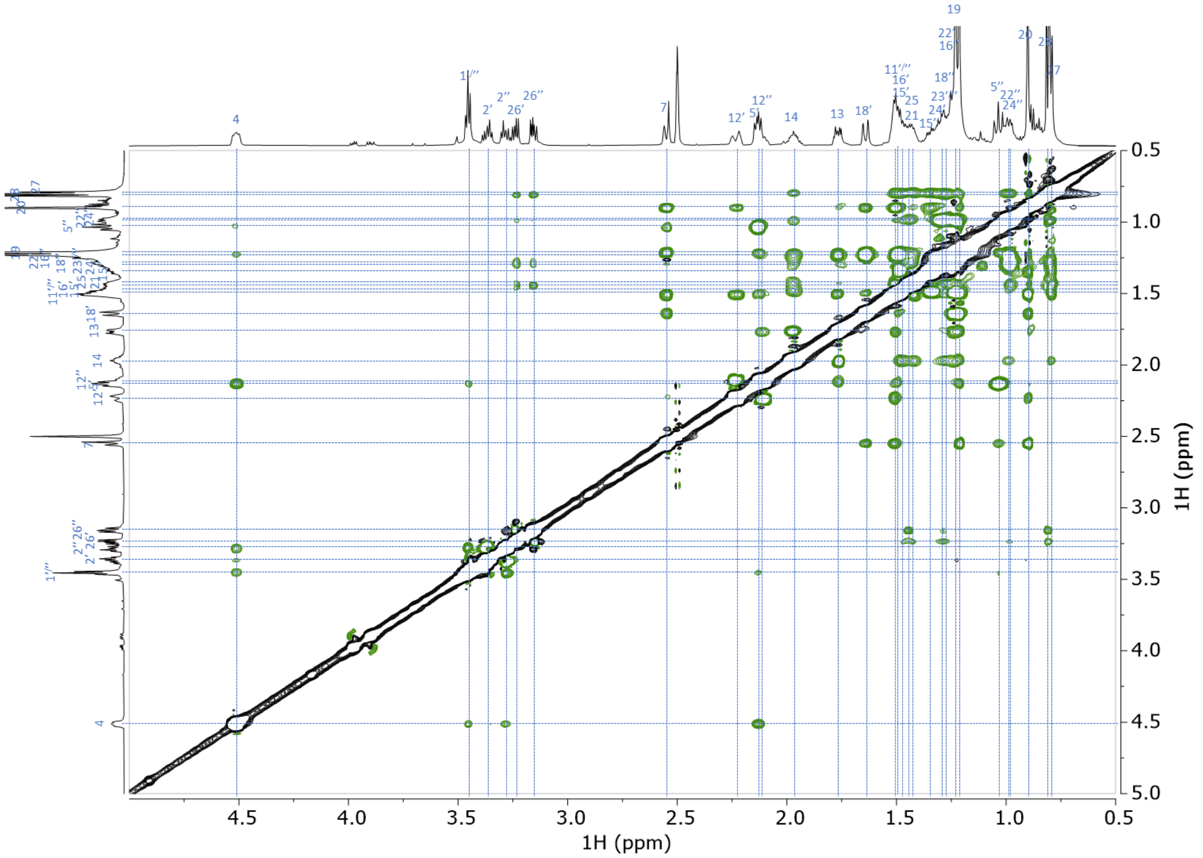

Figure S 5252. ROESY spectrum of **4**.

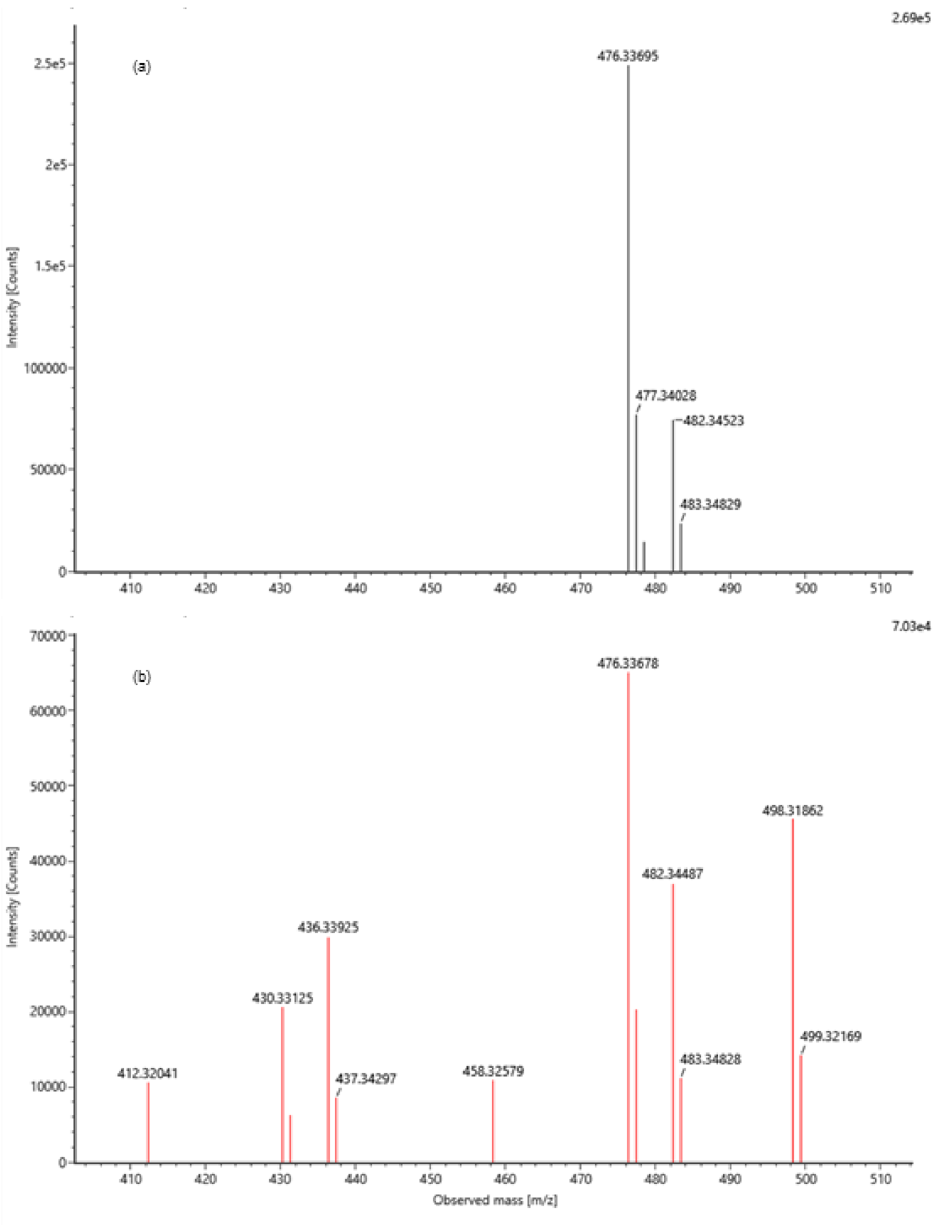

Figure S 5353. Low-collision (top) and High-collision (bottom) energy mass spectra of **5** in ESI^+ mode.

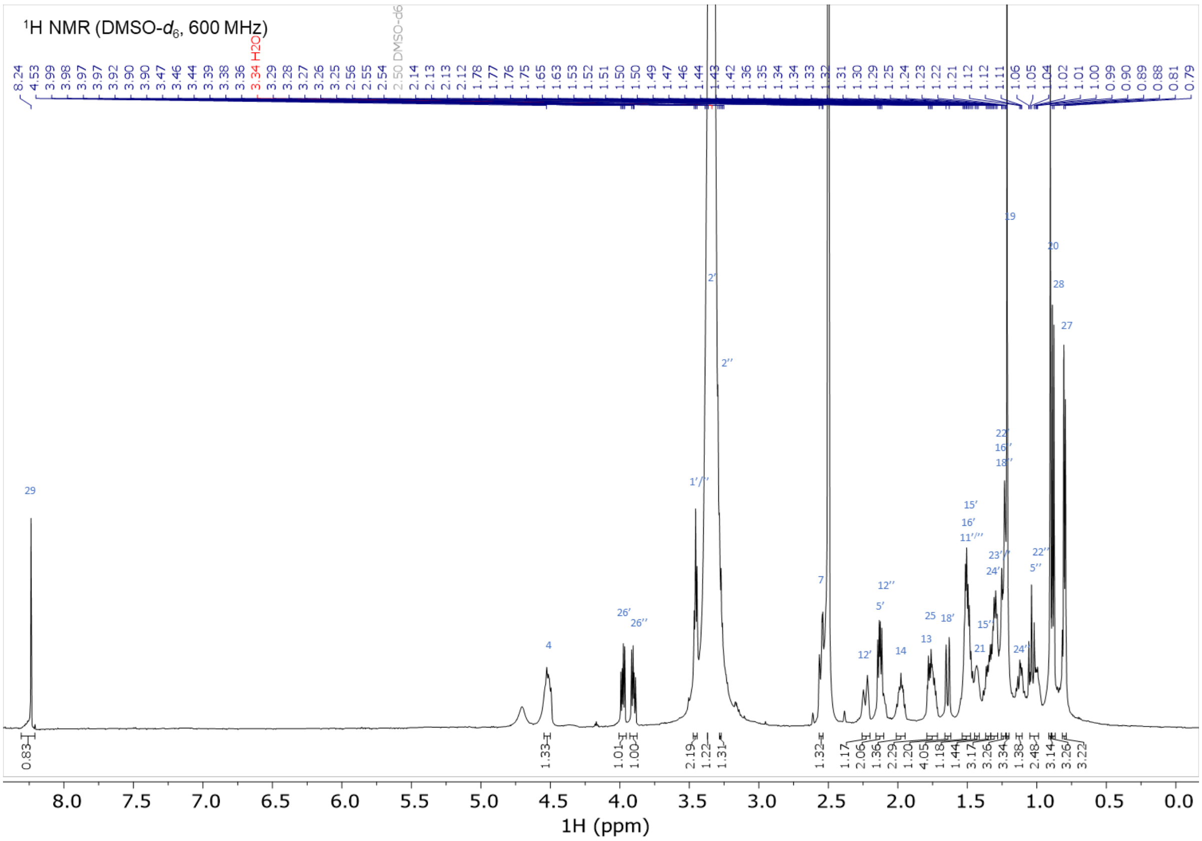

Figure S 5454. ^{1}H NMR spectrum of **5**.

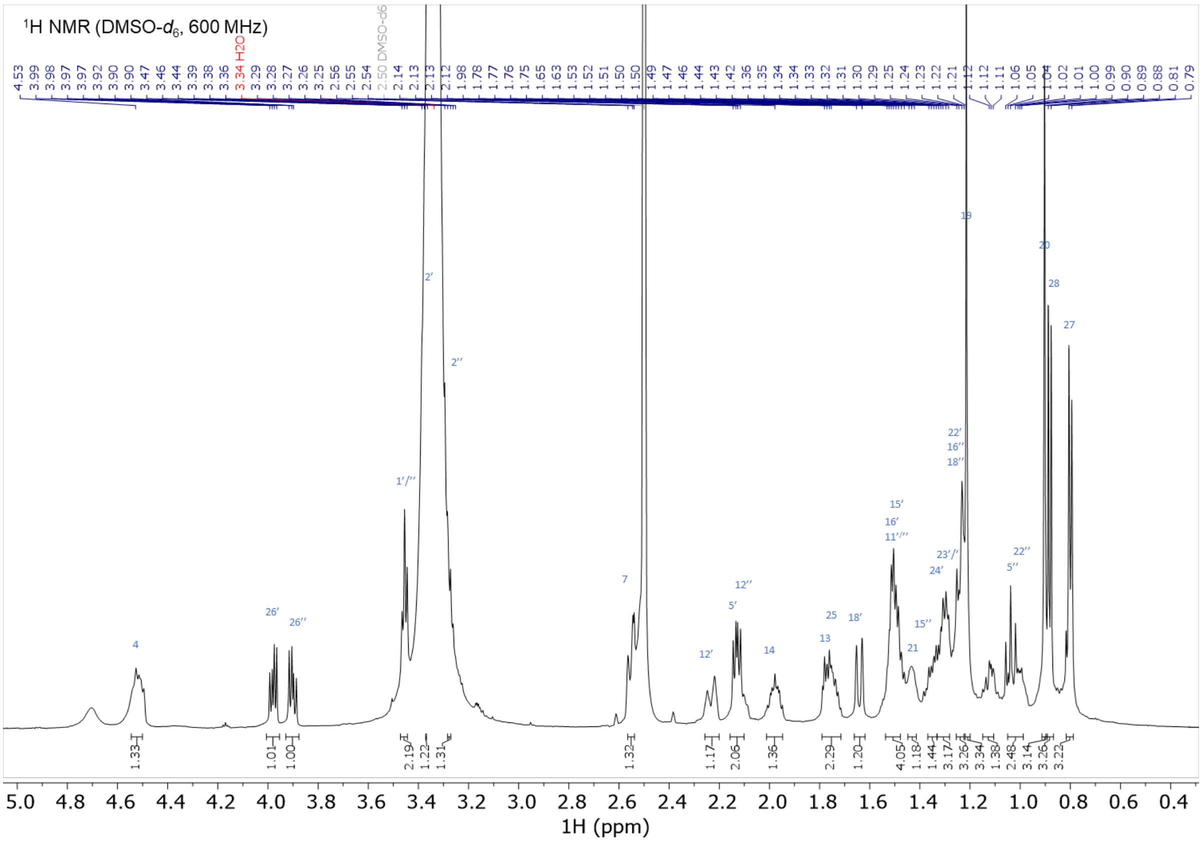

Figure S 5555. ^{1}H NMR spectrum of **5**.

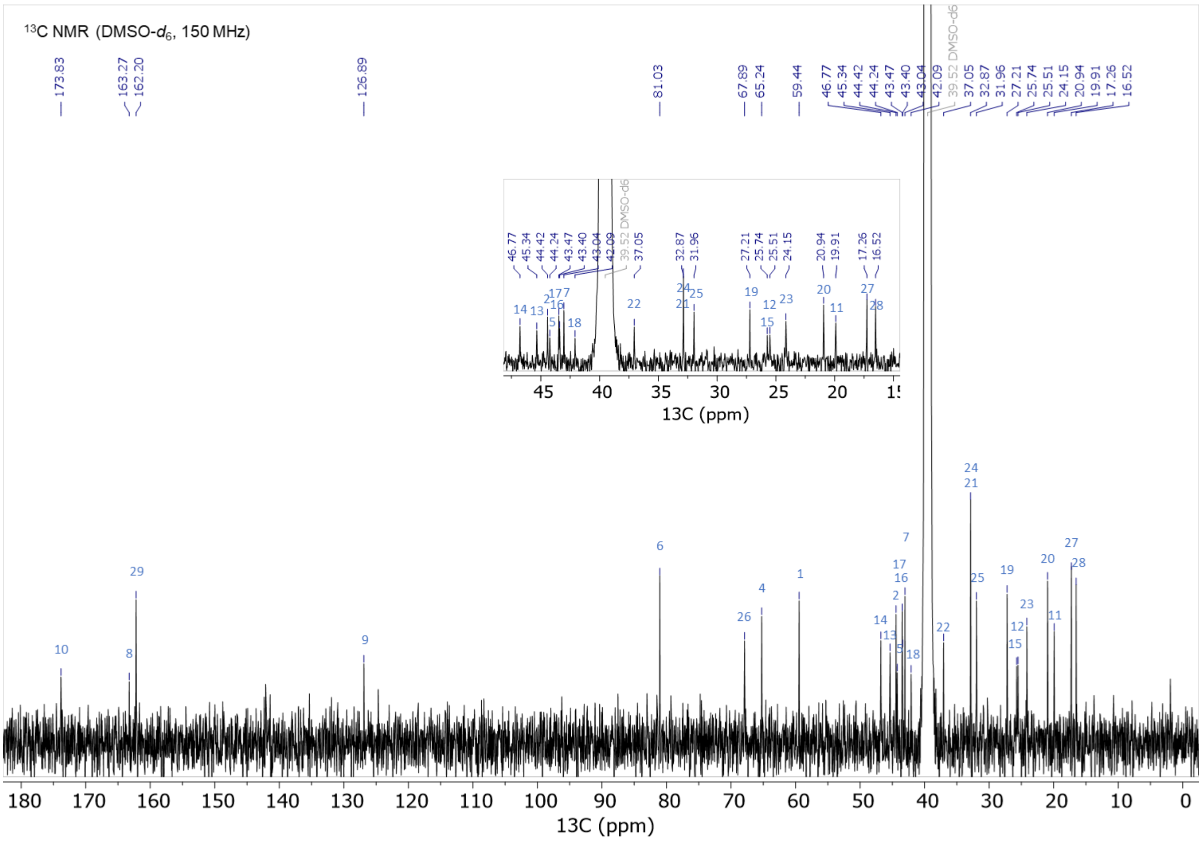

Figure S 5656. ^{13}C NMR spectrum of **5**.

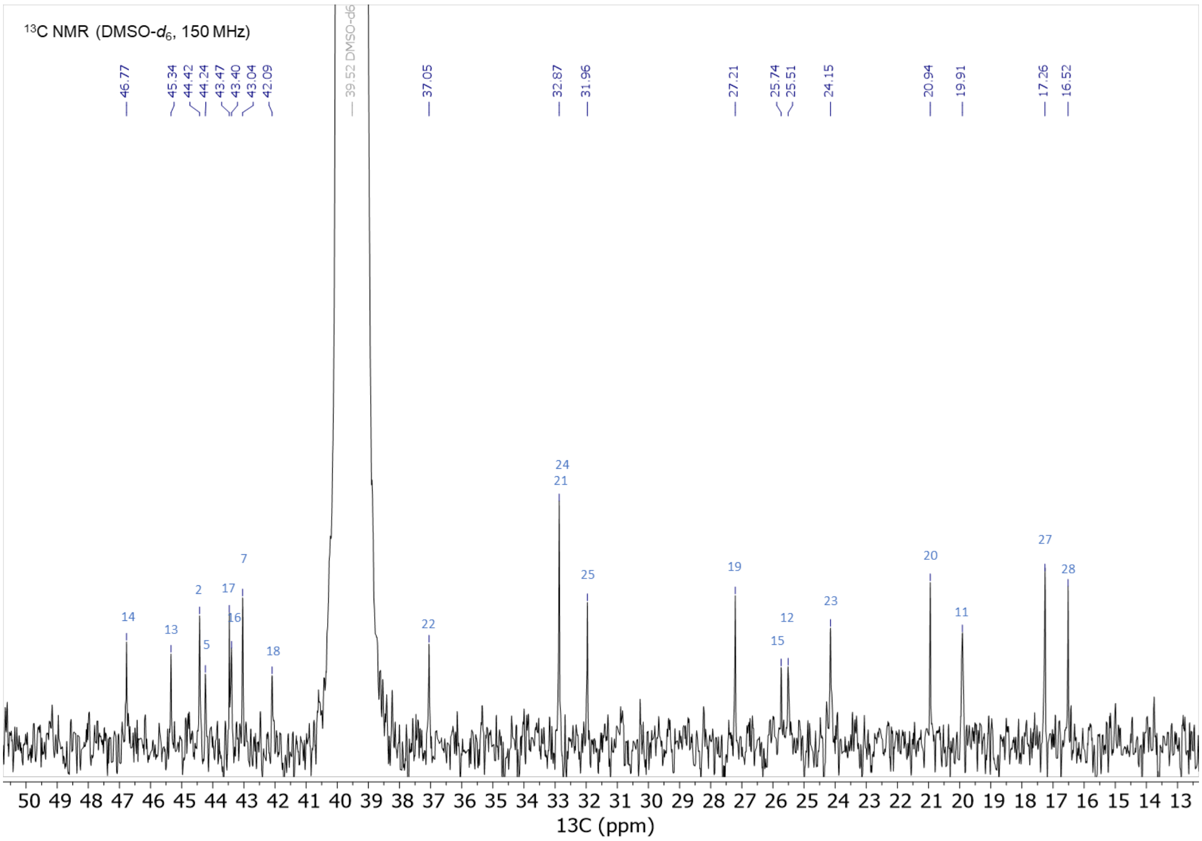

Figure S 5757. ^{13}C NMR (Expanded) spectrum of **5**.

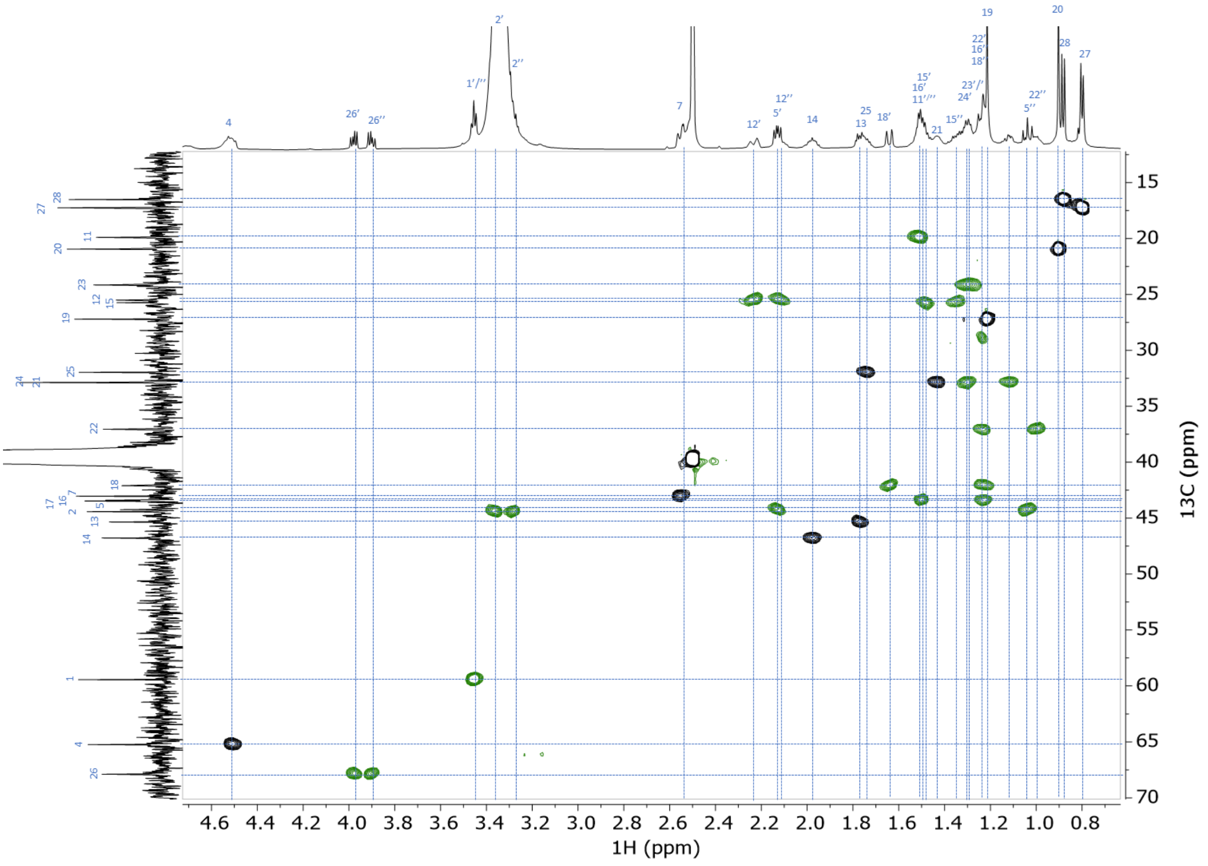

Figure S 5858. HSQC spectrum of **5**.

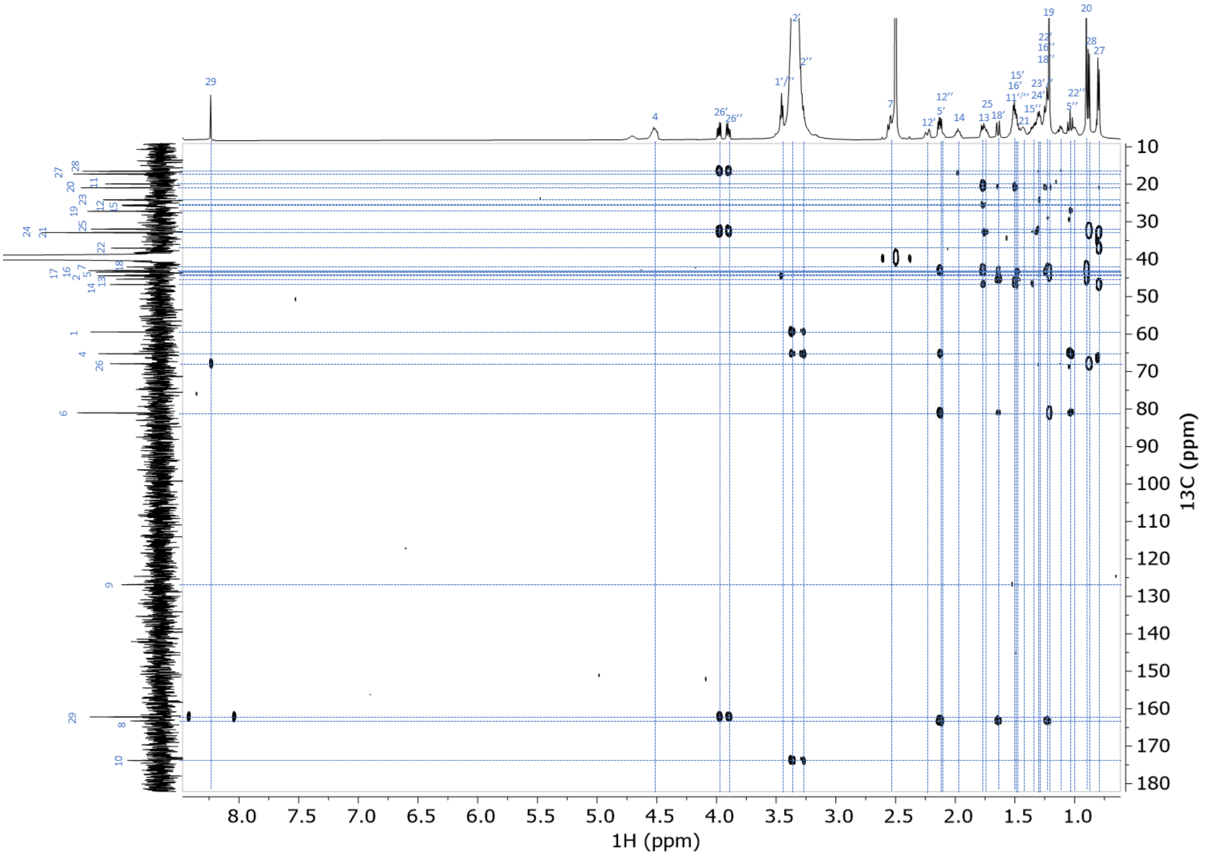

Figure S 5959. HMBC spectrum of **5**.

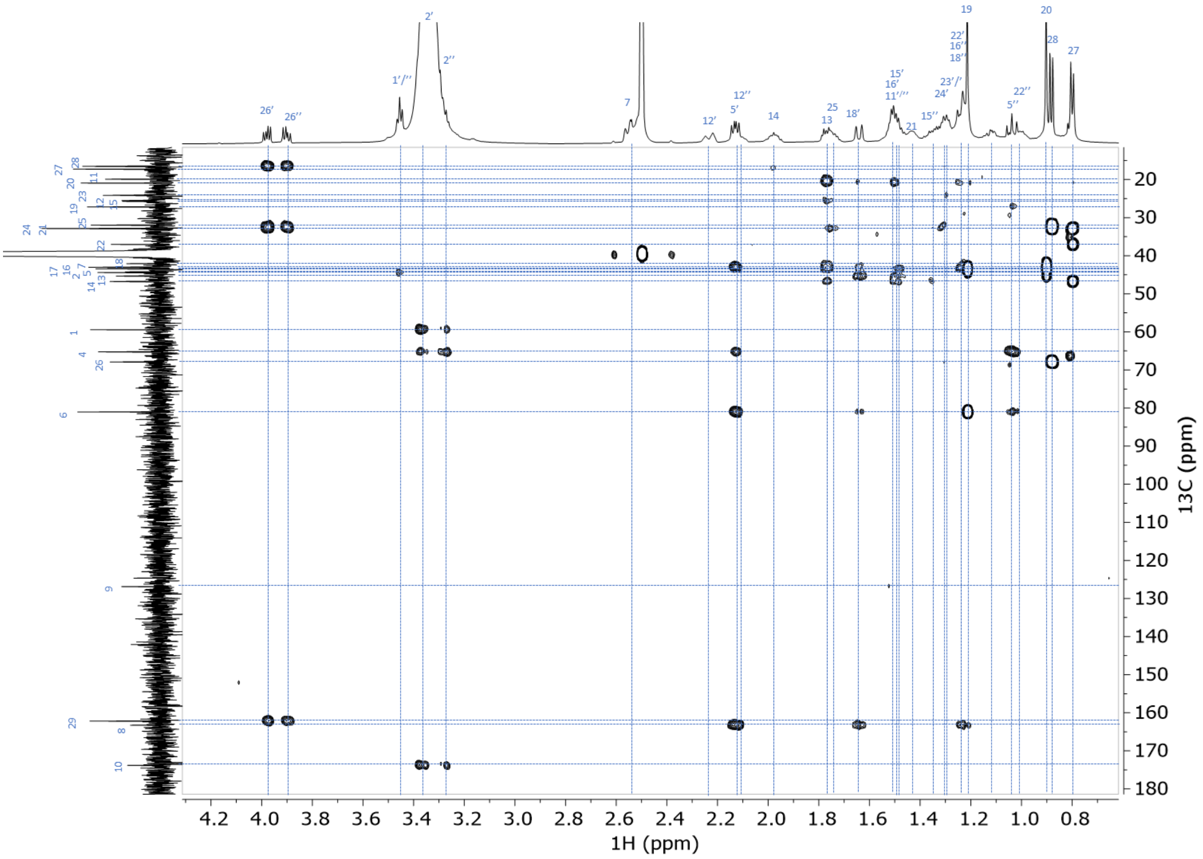

Figure S 6060. HMBC (Expanded) spectrum of **5**.

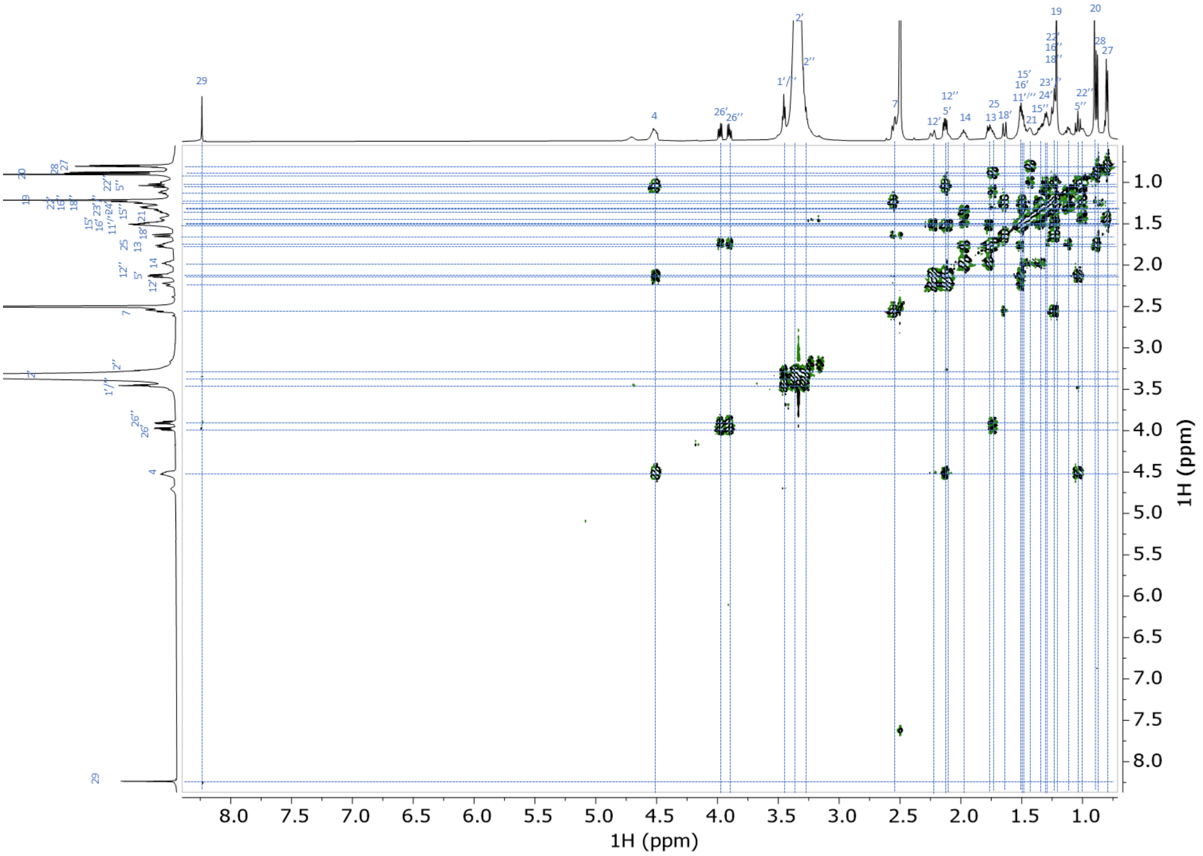

Figure S 6161. DQF-COSY spectrum of **5**.

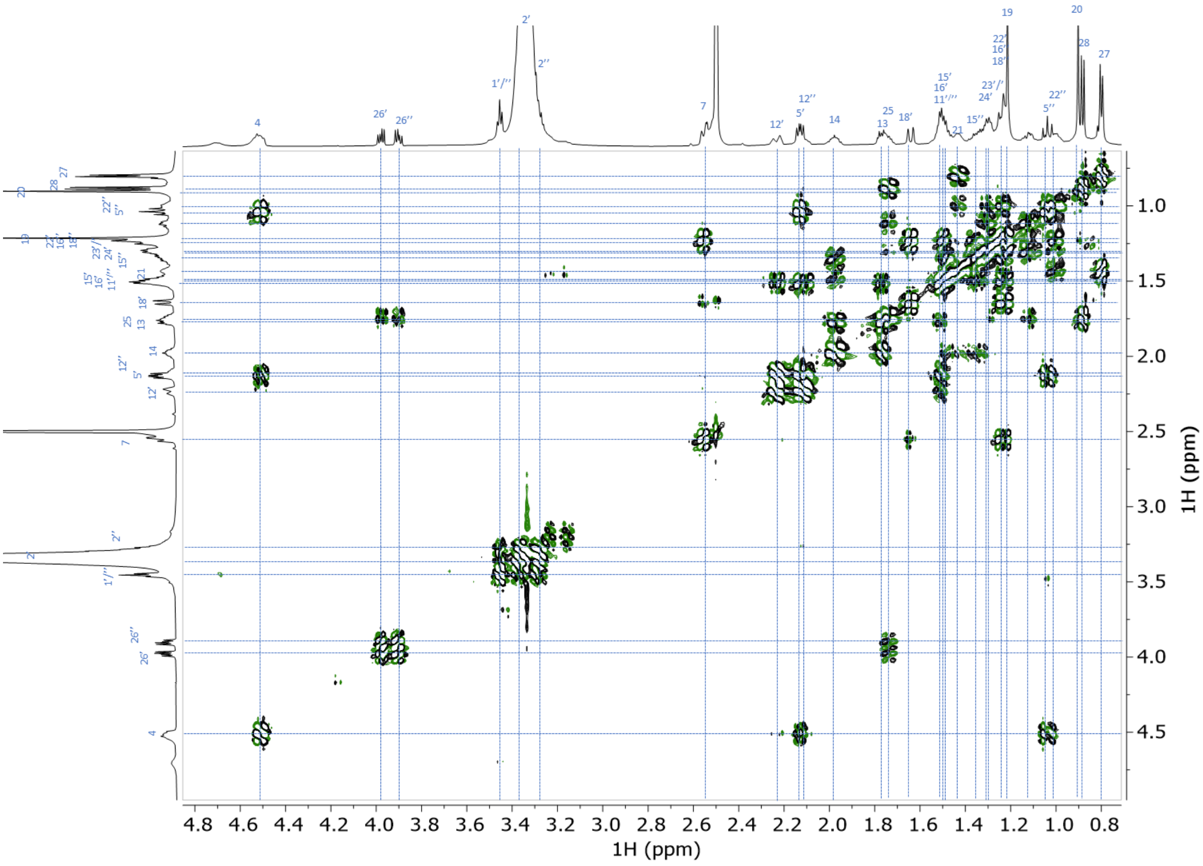

Figure S 6262. DQF-COSY (Expanded) spectrum of **5**.

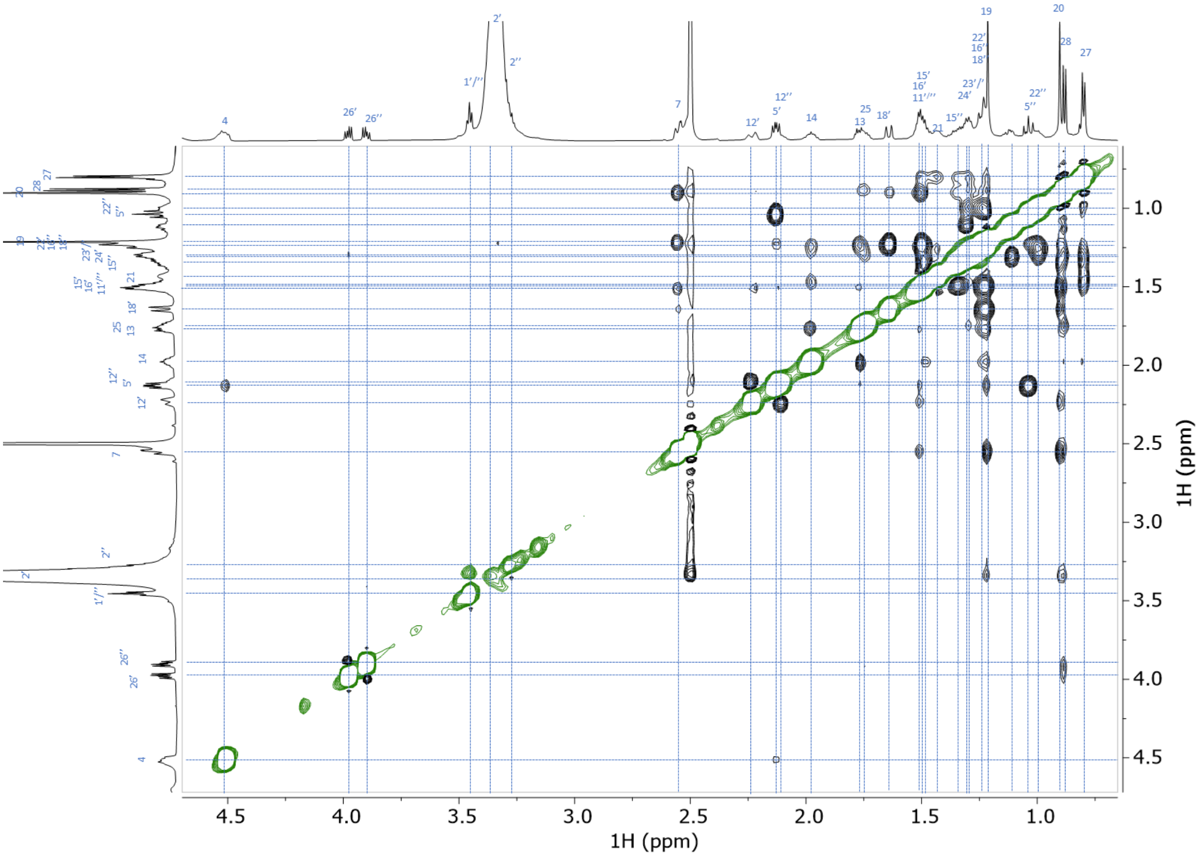

Figure S 6363. ROESY spectrum of **5**.

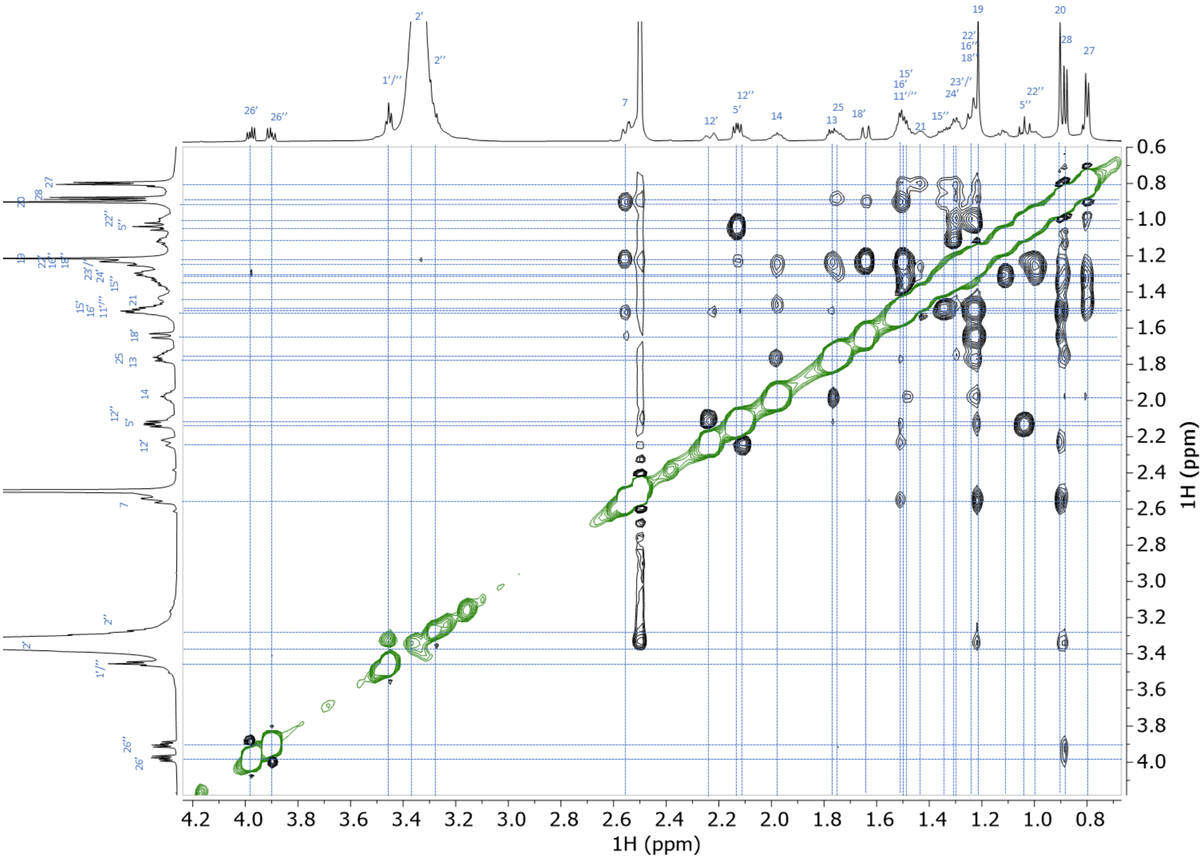

Figure S 6464. ROESY (Expanded) spectrum of **5**.

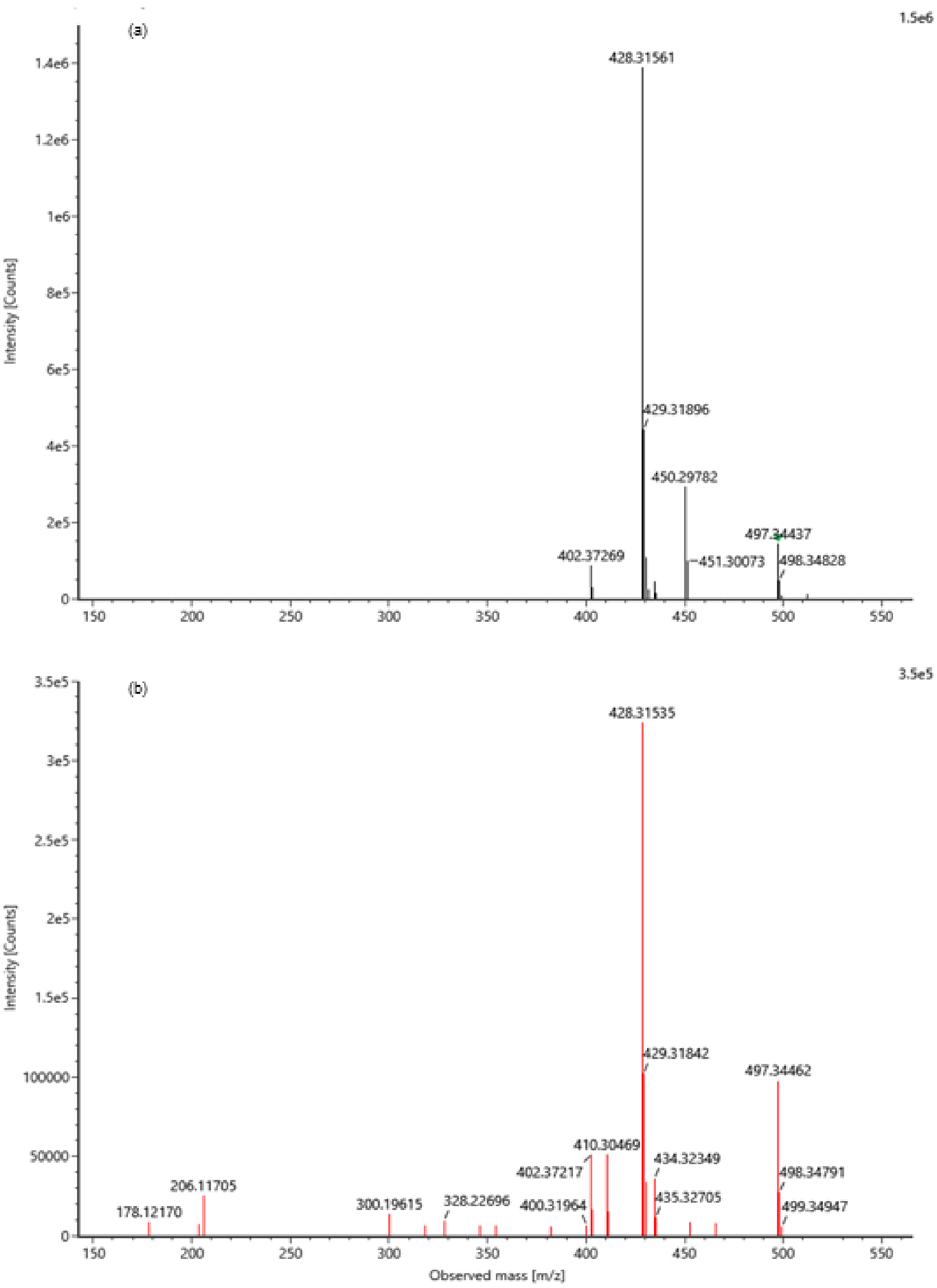

Figure S 6565. Low-collision (top) and High-collision (bottom) energy mass spectra of **6** in ESI^{+} mode.

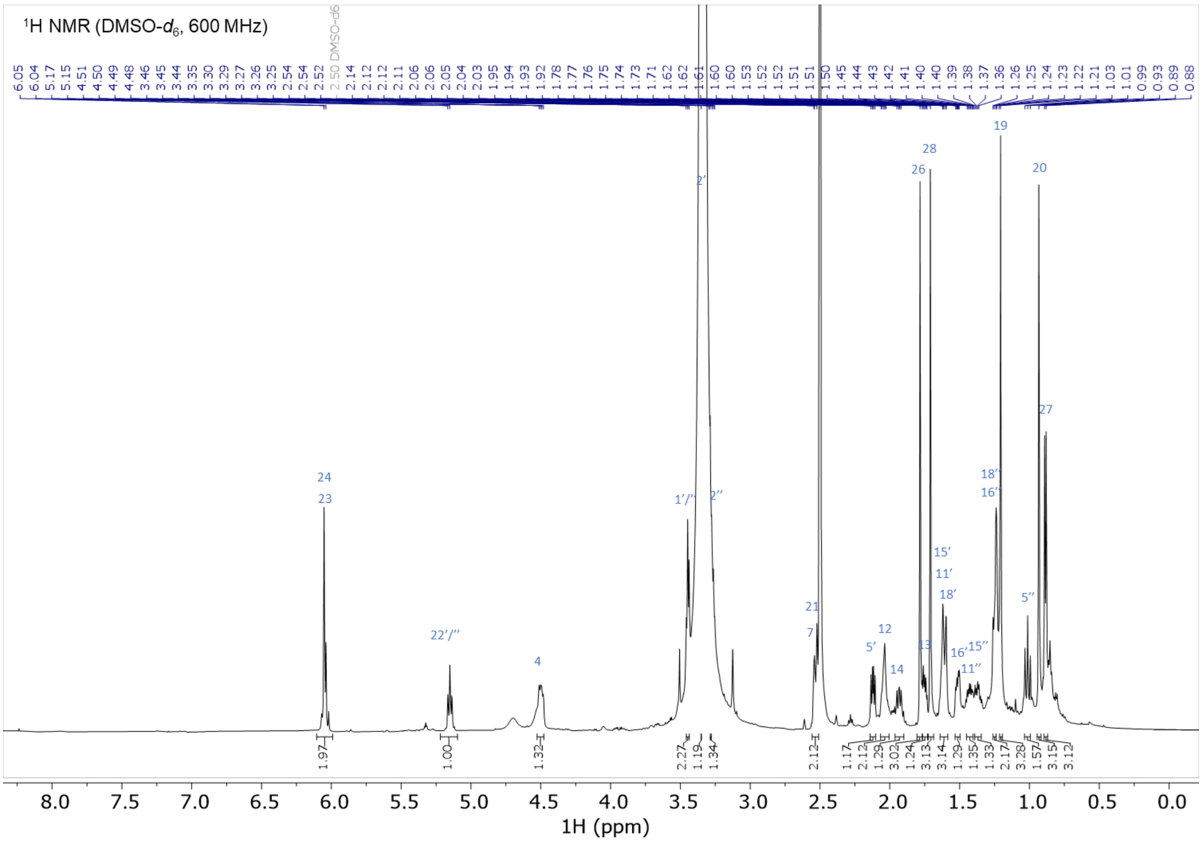

Figure S 6666. ^{1}H NMR spectrum of **6**.

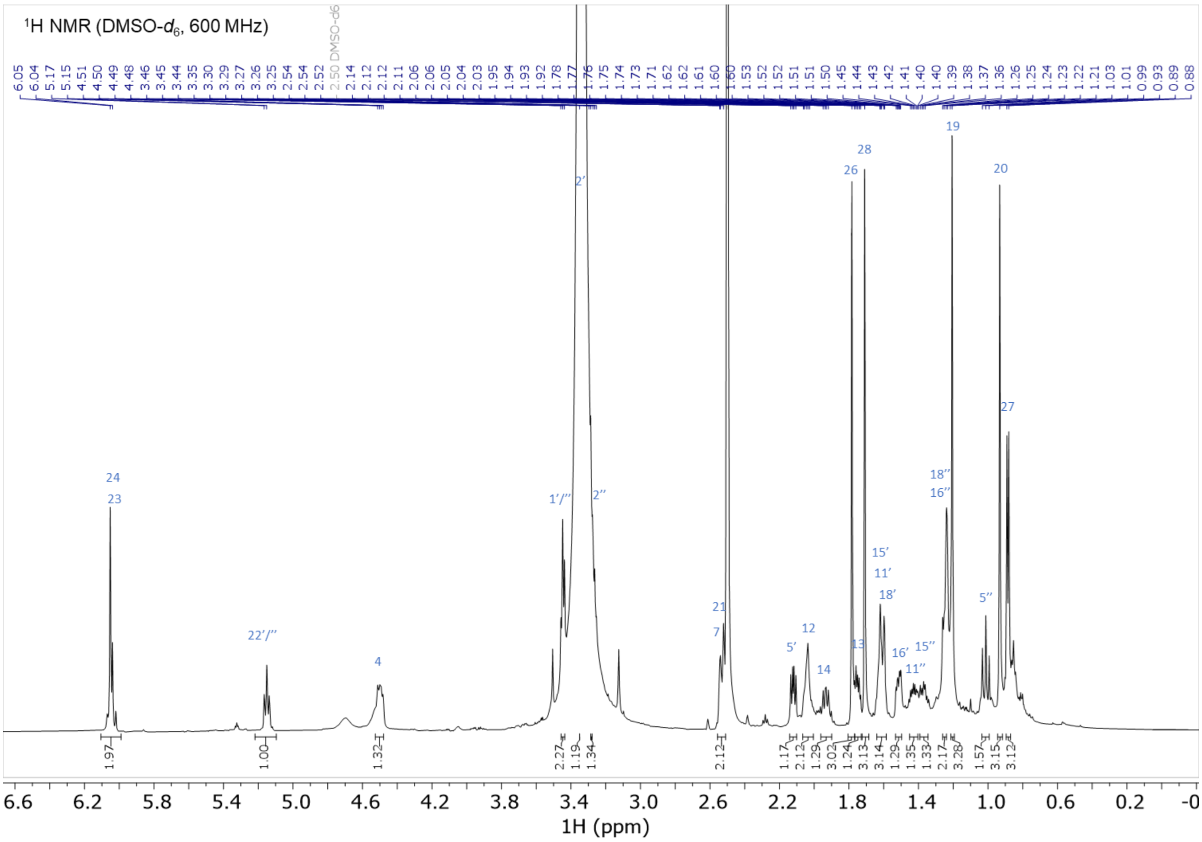

Figure S 6767. ^{1}H NMR (Expanded) spectrum of **6**.

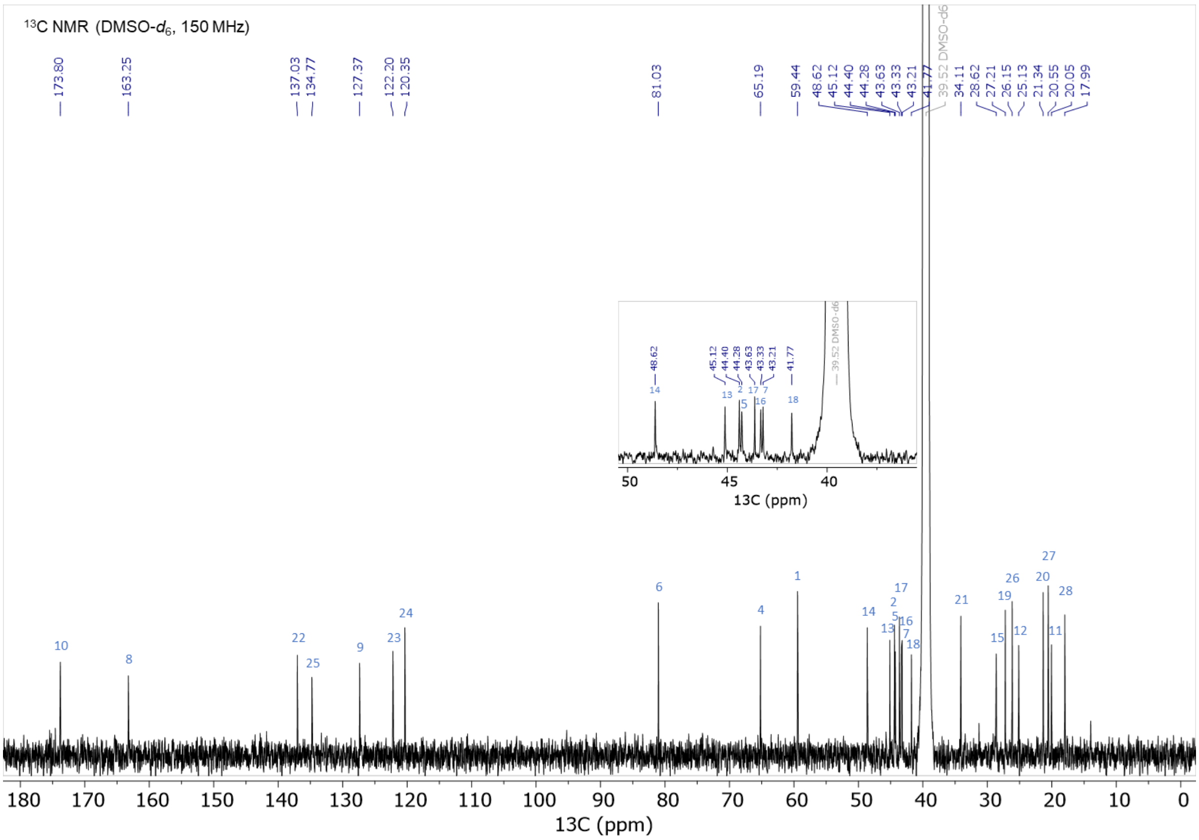

Figure S 6868. ^{13}C NMR spectrum **6**.

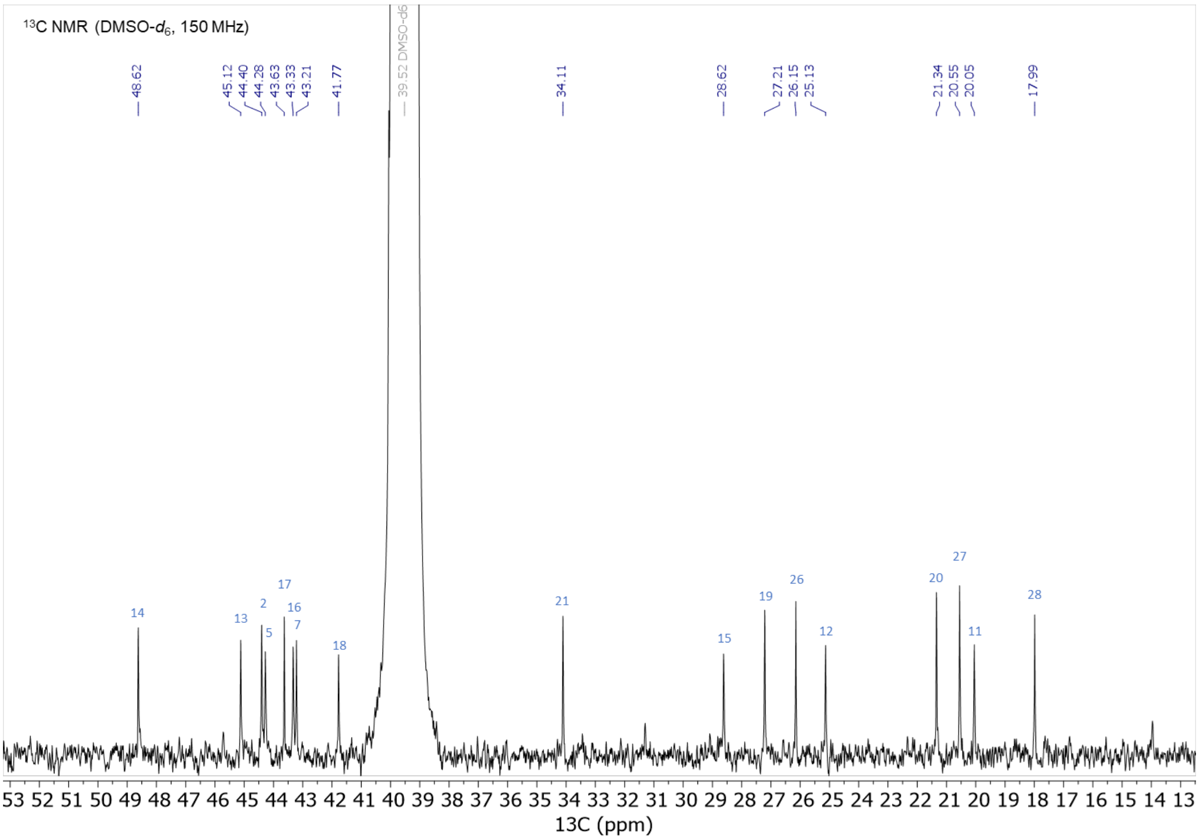

Figure S 6969. ^{13}C NMR (Expanded) spectrum of **6**.

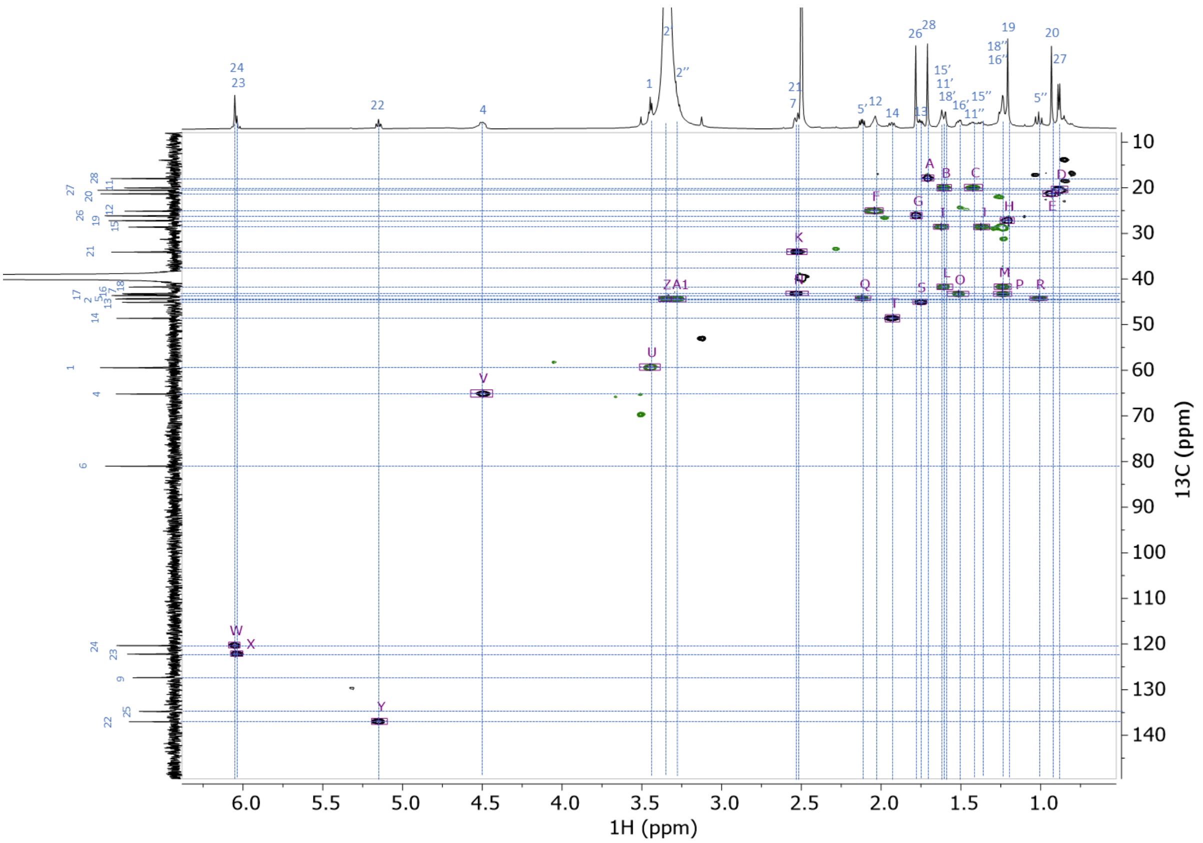

Figure S 7070. HSQC spectrum of **6**.

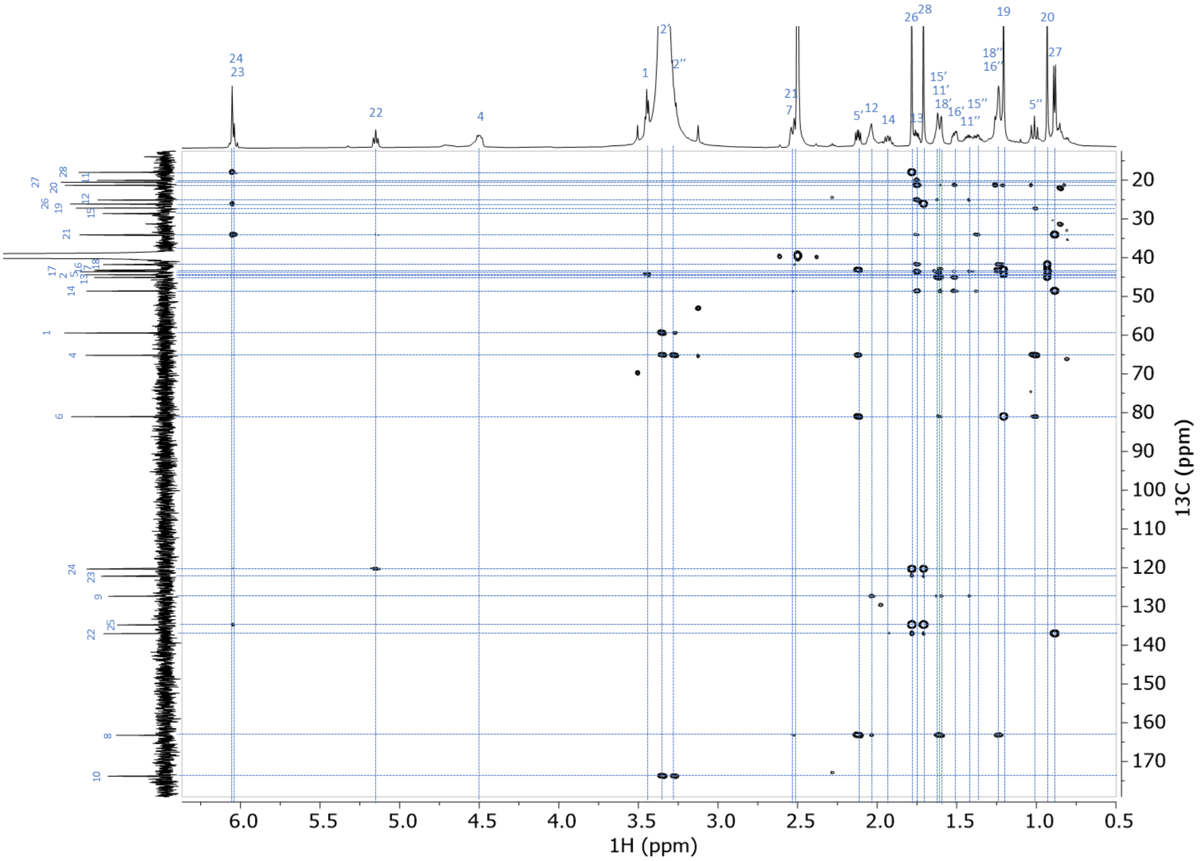

Figure S 7171. HMBC spectrum of **6**.

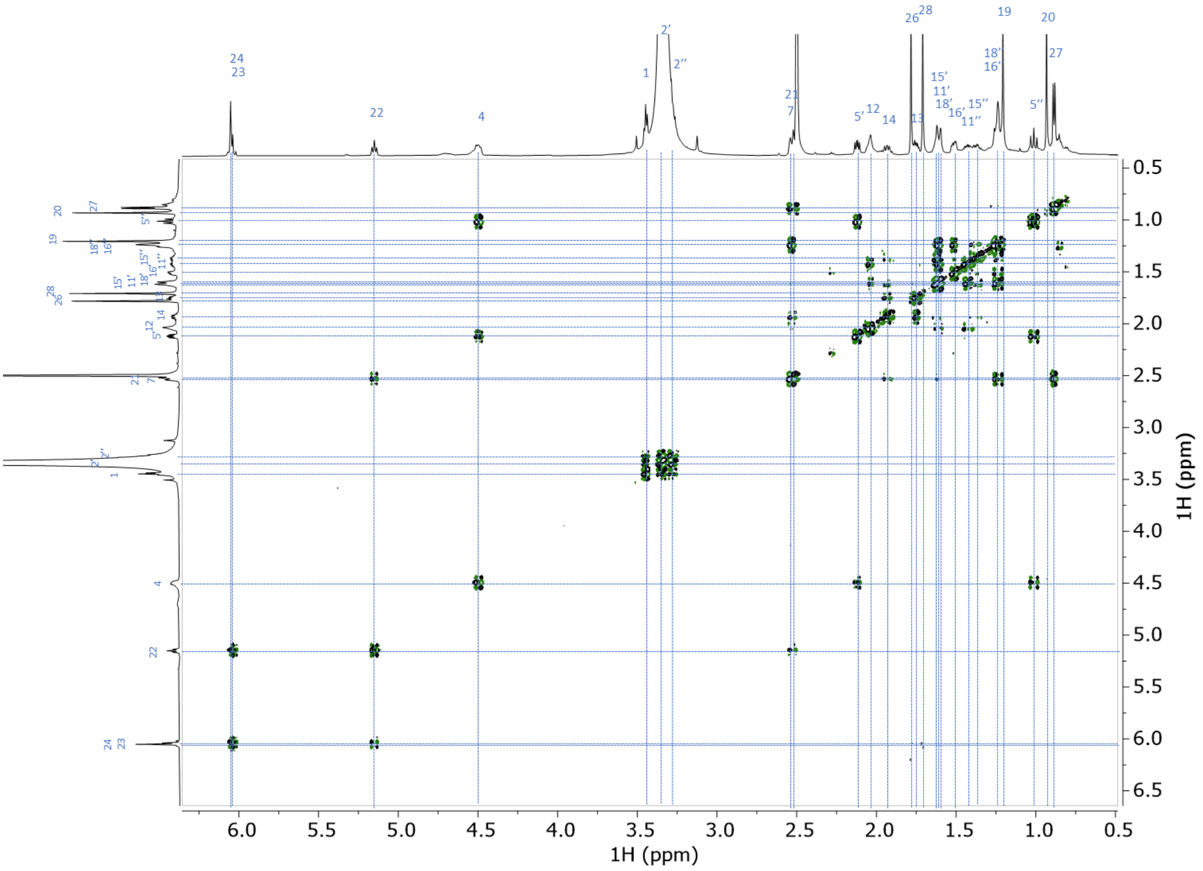

Figure S 7272. DQF-COSY spectrum of **6**.

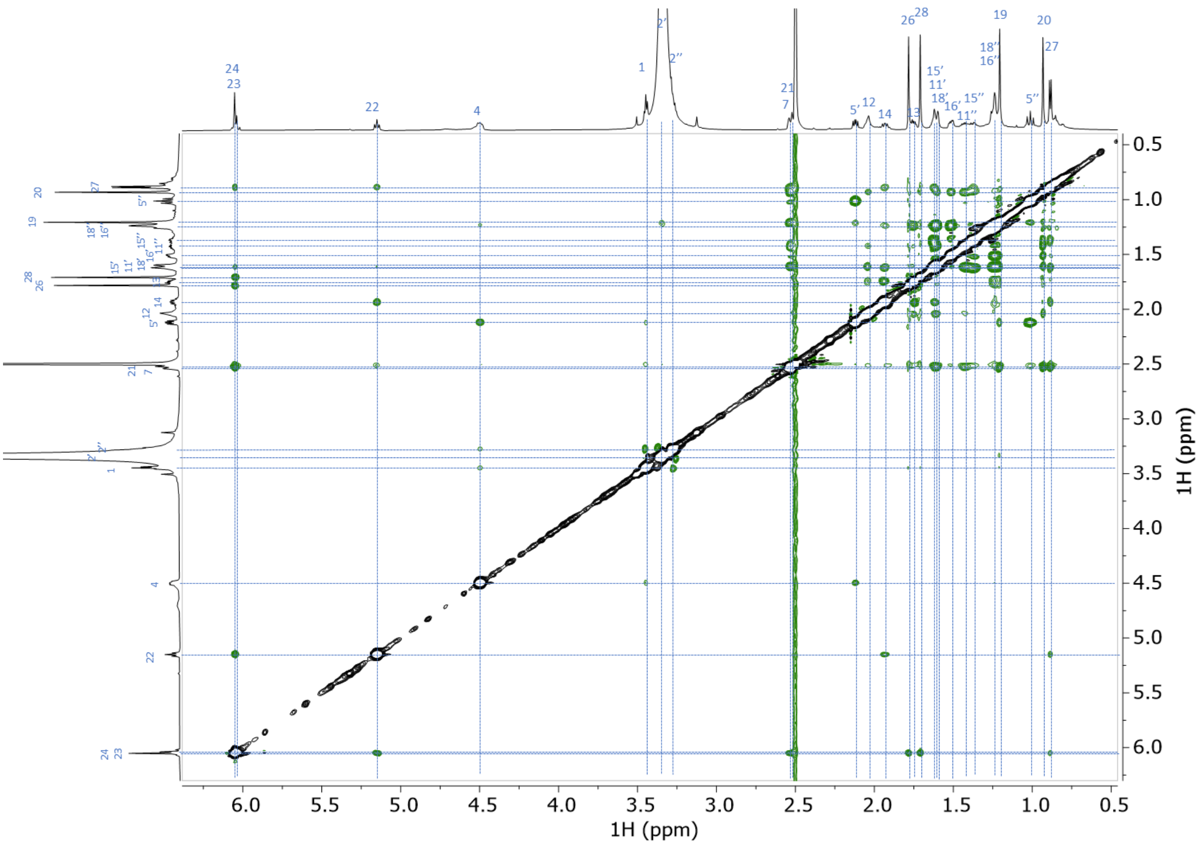

Figure S 7373. ROESY spectrum of **6**.

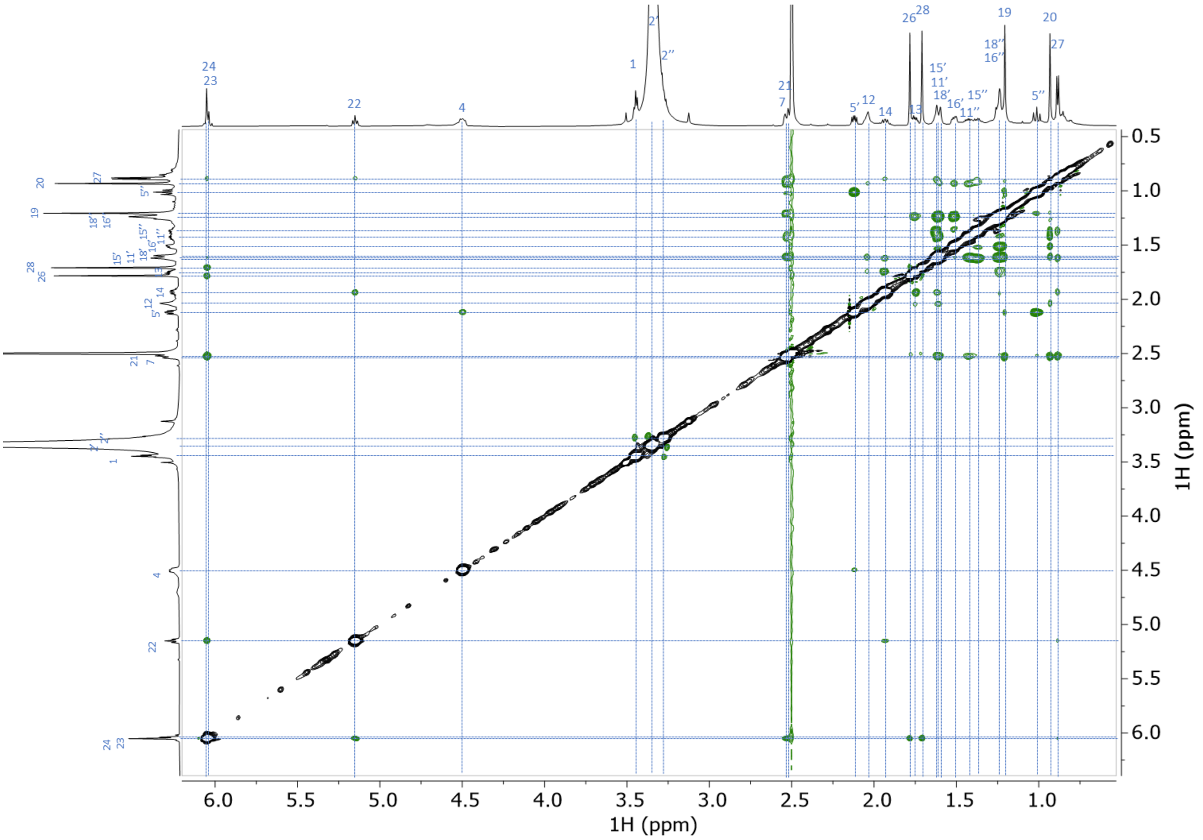

Figure S 7474. ROESY spectrum of **6**.

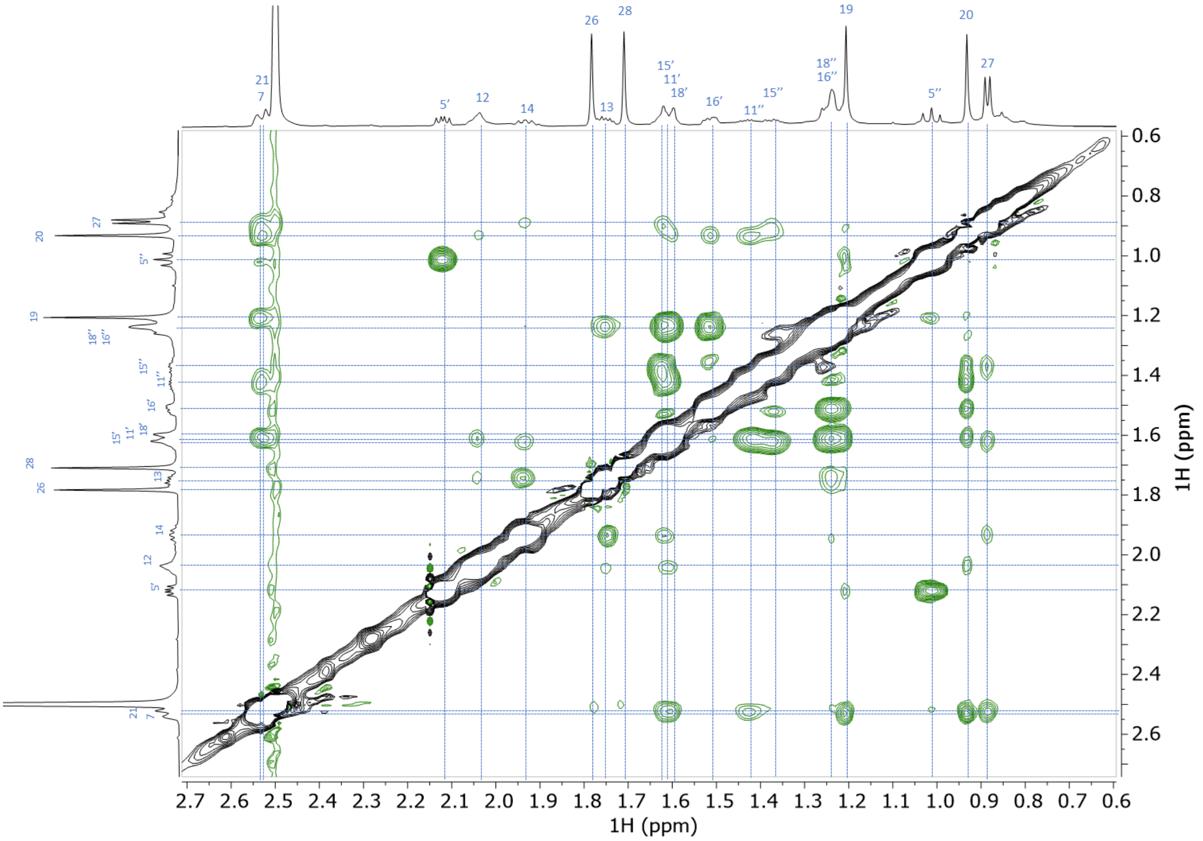

Figure S 7575. ROESY (Expanded) spectrum of **6**.

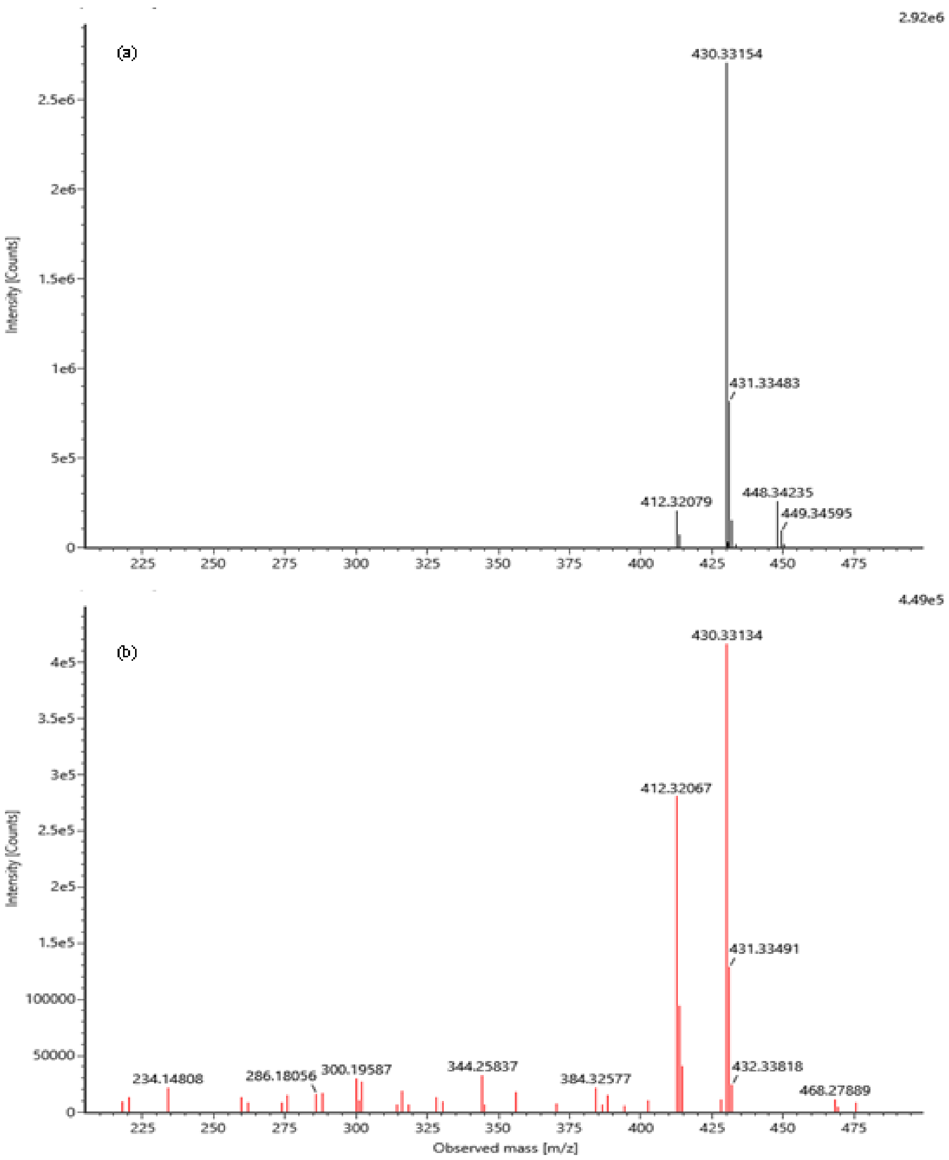

Figure S 7676. Low-collision (top) and High-collision (bottom) energy mass spectra of **7** in ESI^{+} mode.

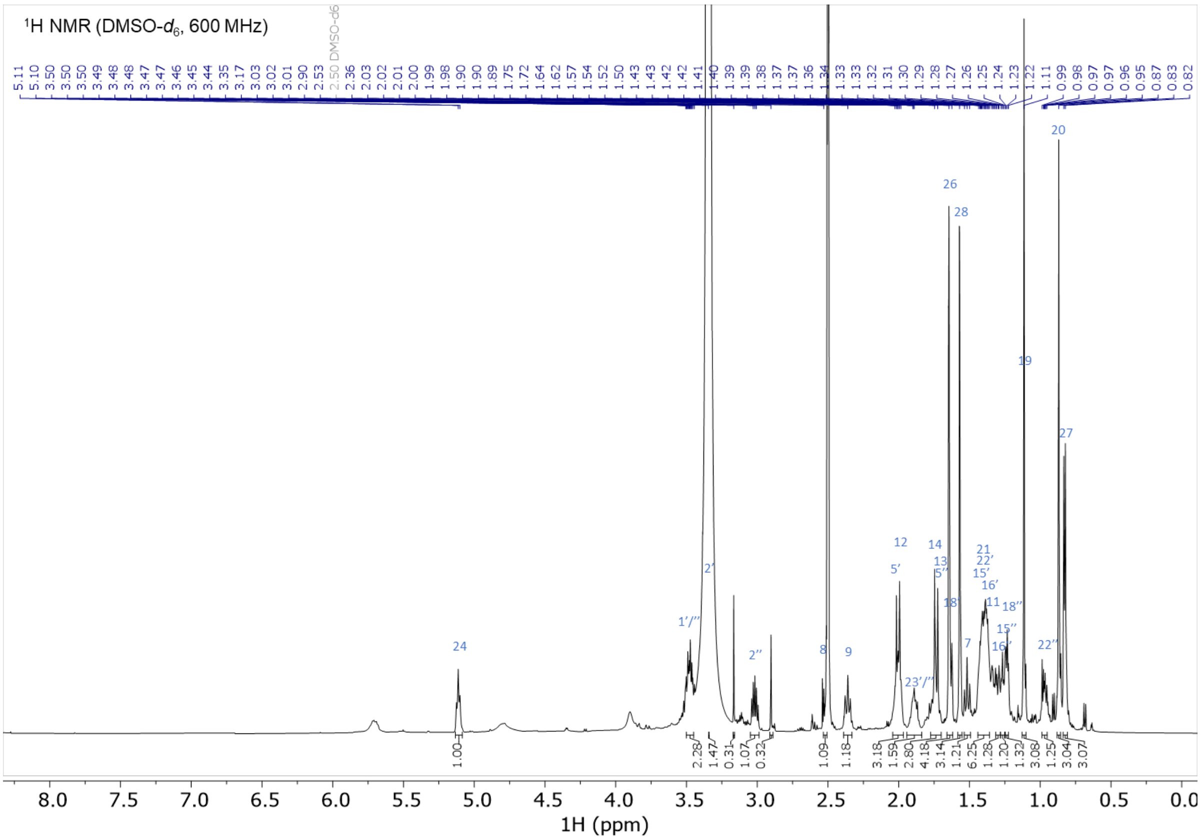

Figure S 7777. ¹H NMR spectrum of **7**.

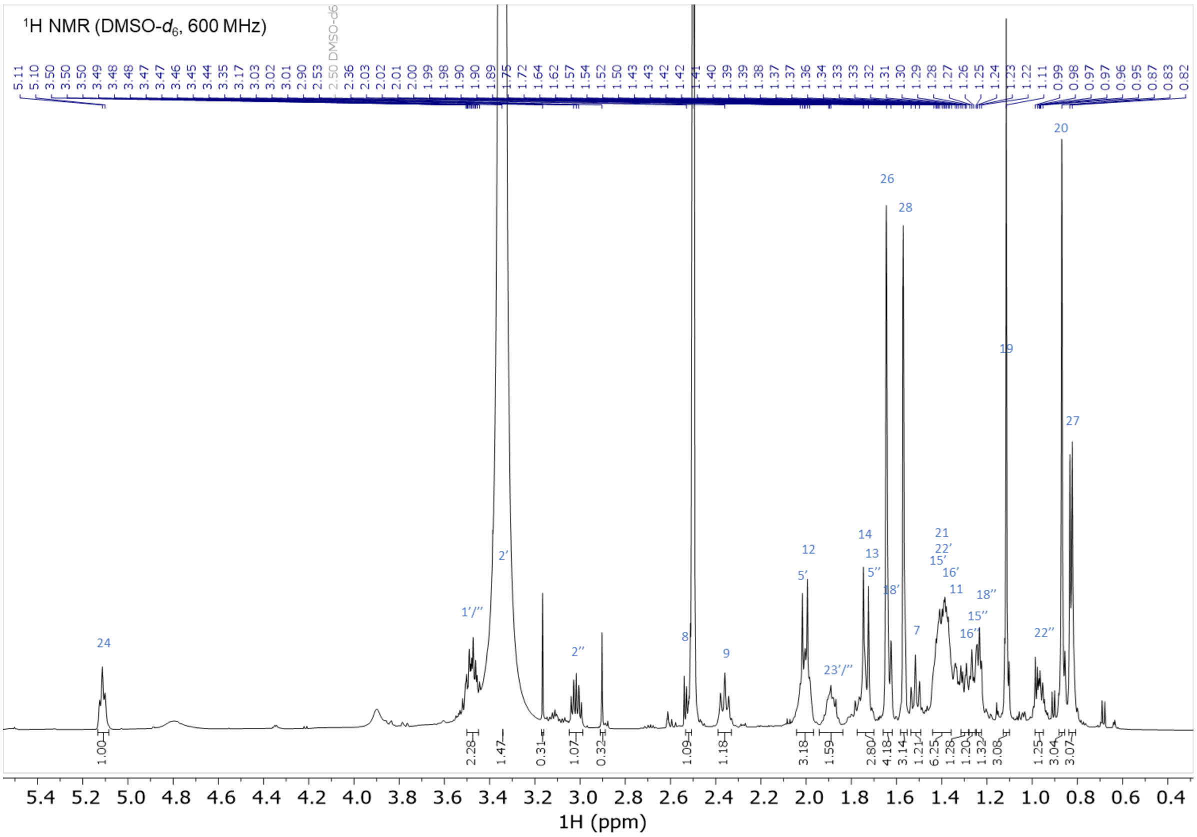

Figure S 7878. ¹H NMR (Expanded) spectrum of **7**.

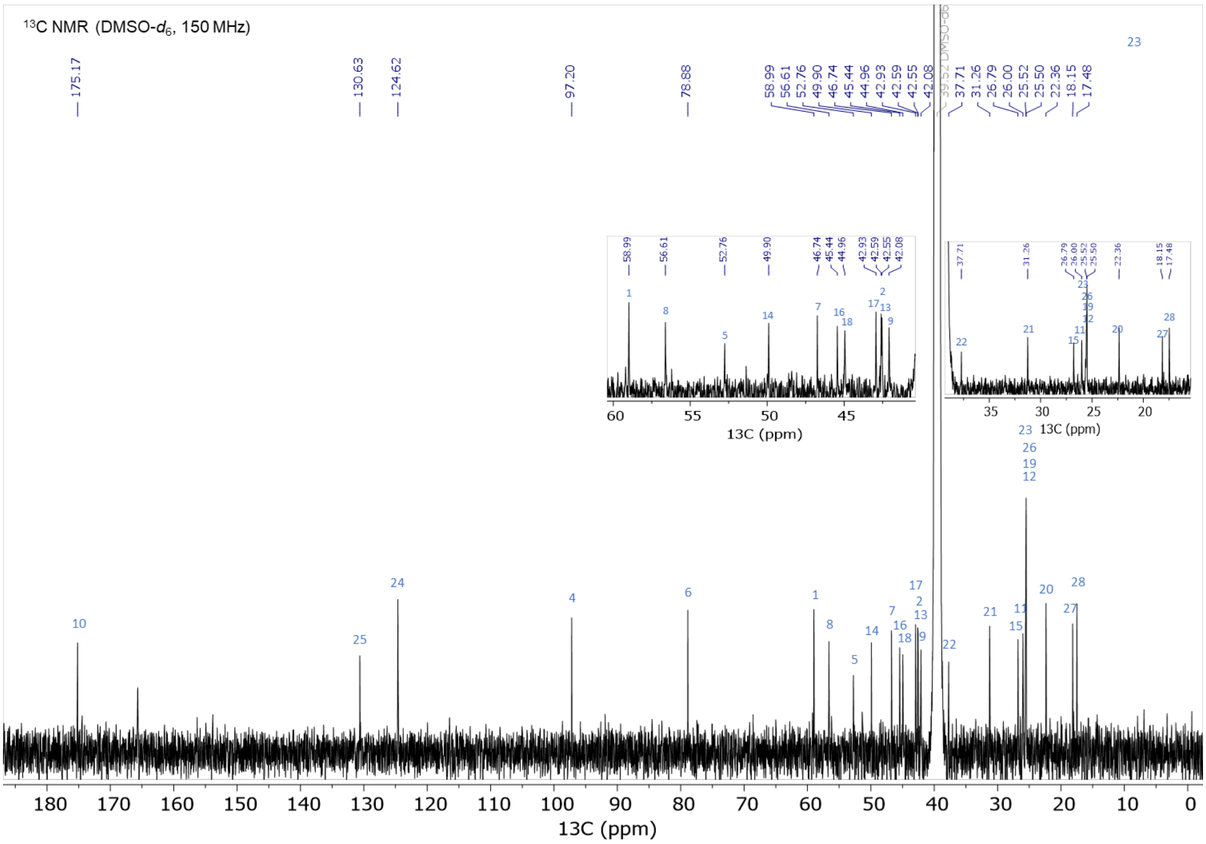

Figure S 7979. ^{13}C NMR spectrum of **7**.

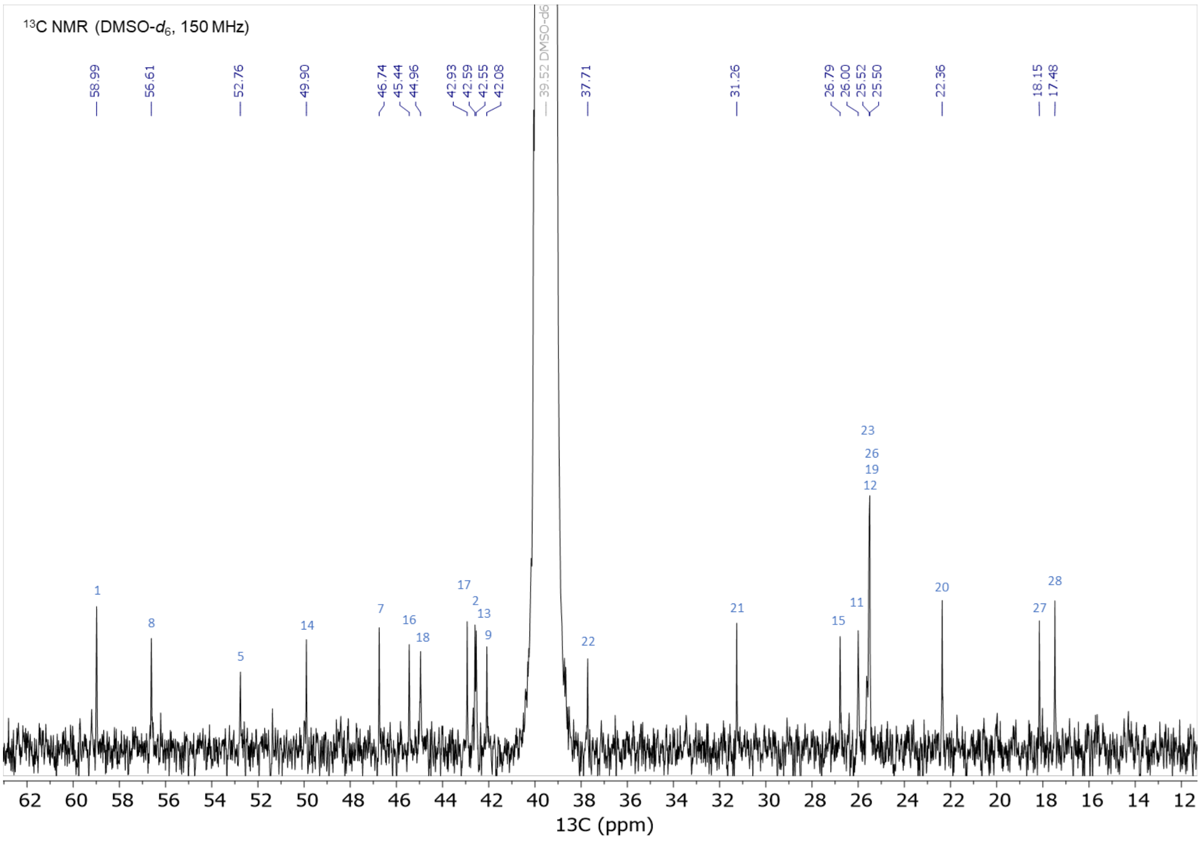

Figure S 8080. ^{13}C NMR spectrum of **7**.

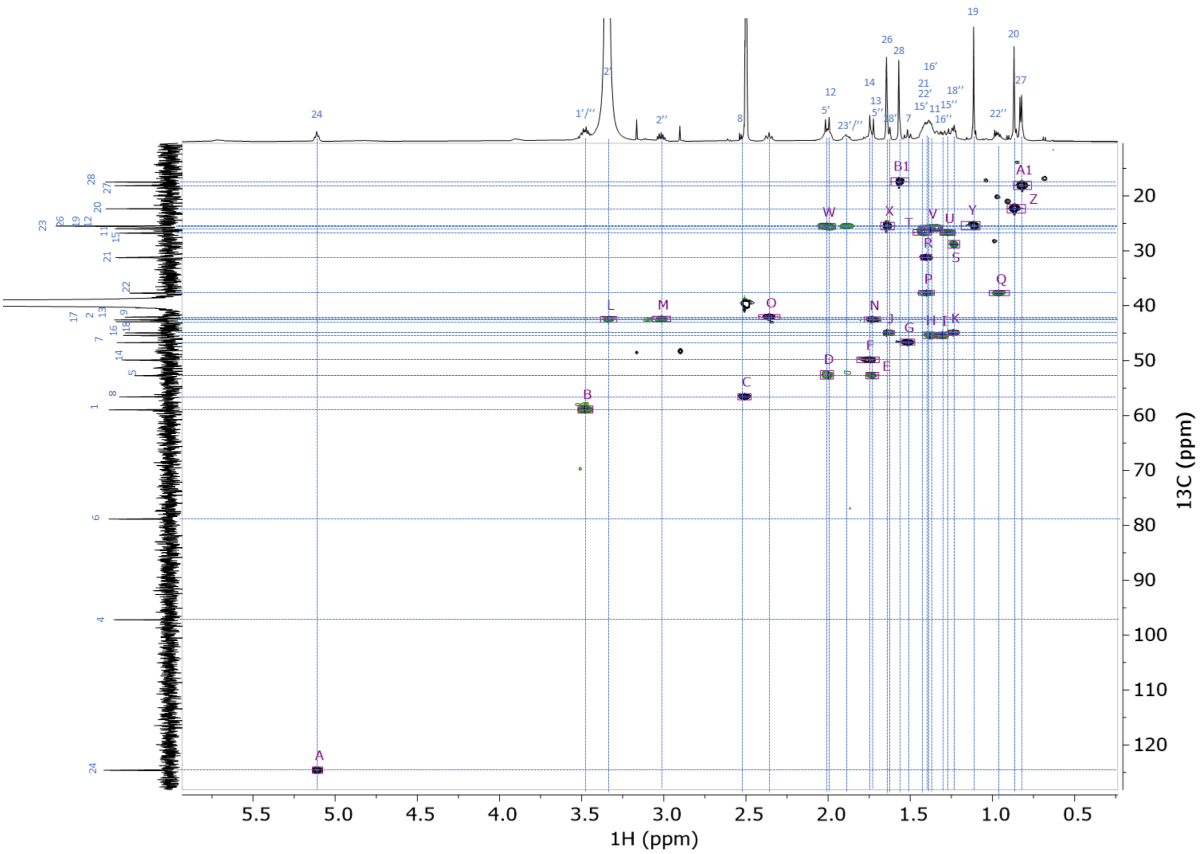

Figure S 8181. HSQC spectrum of **7**.

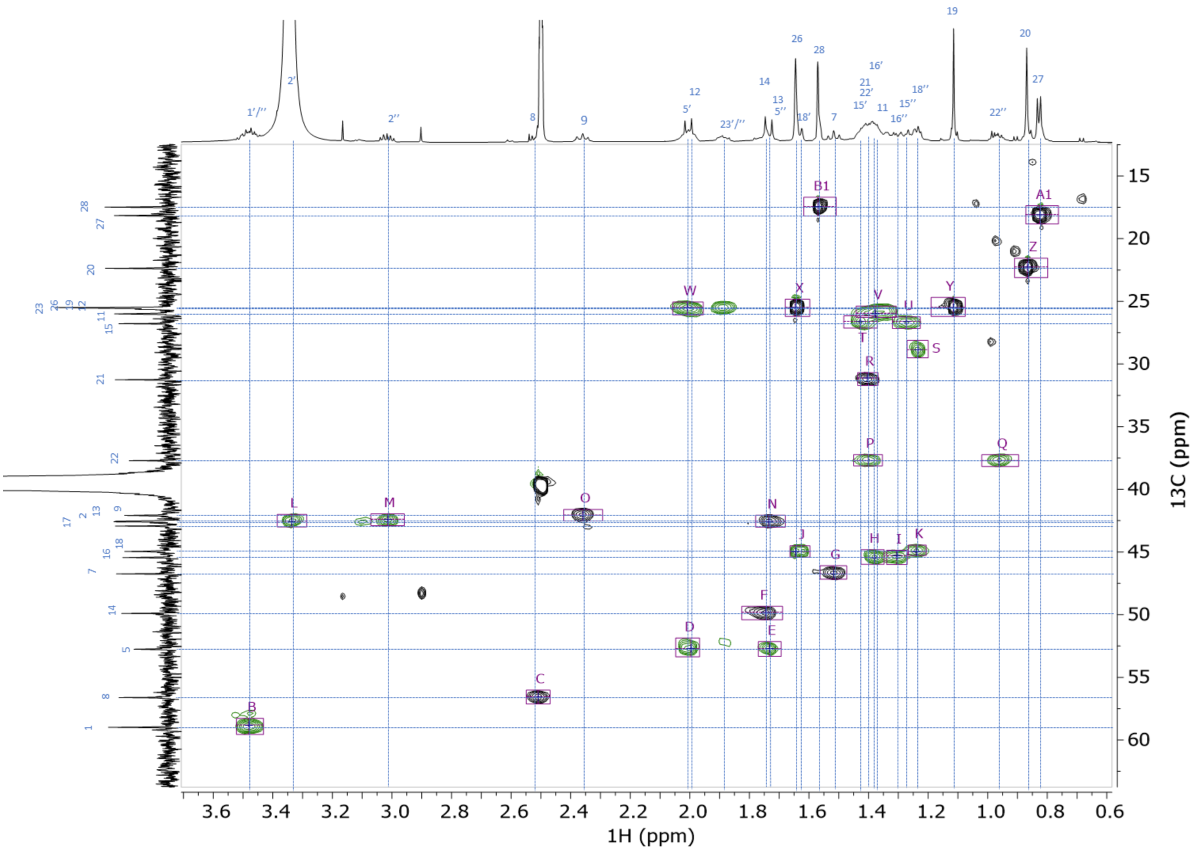

Figure S 8282. HSQC (Expanded) spectrum of **7**.

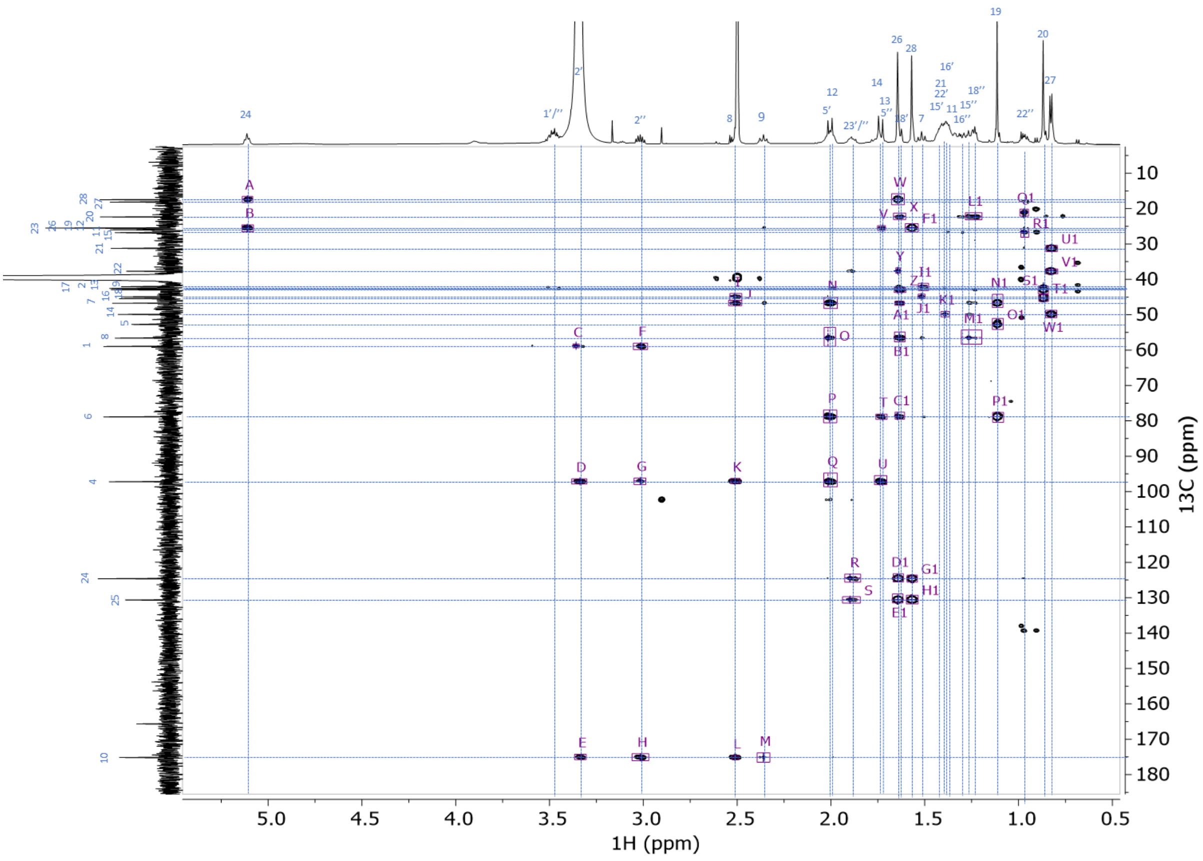

Figure S 8383. HMBC spectrum of **7**.

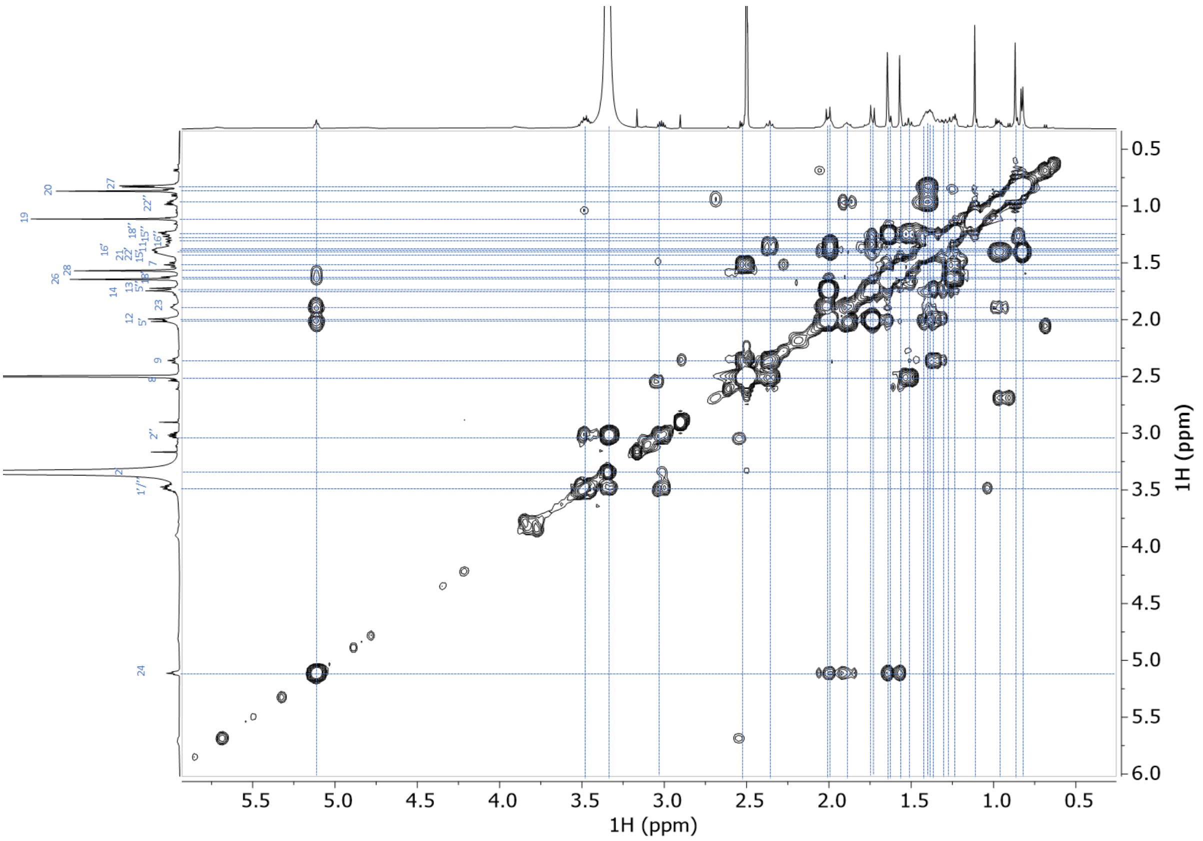

Figure S 8484. COSY spectrum of **7**.

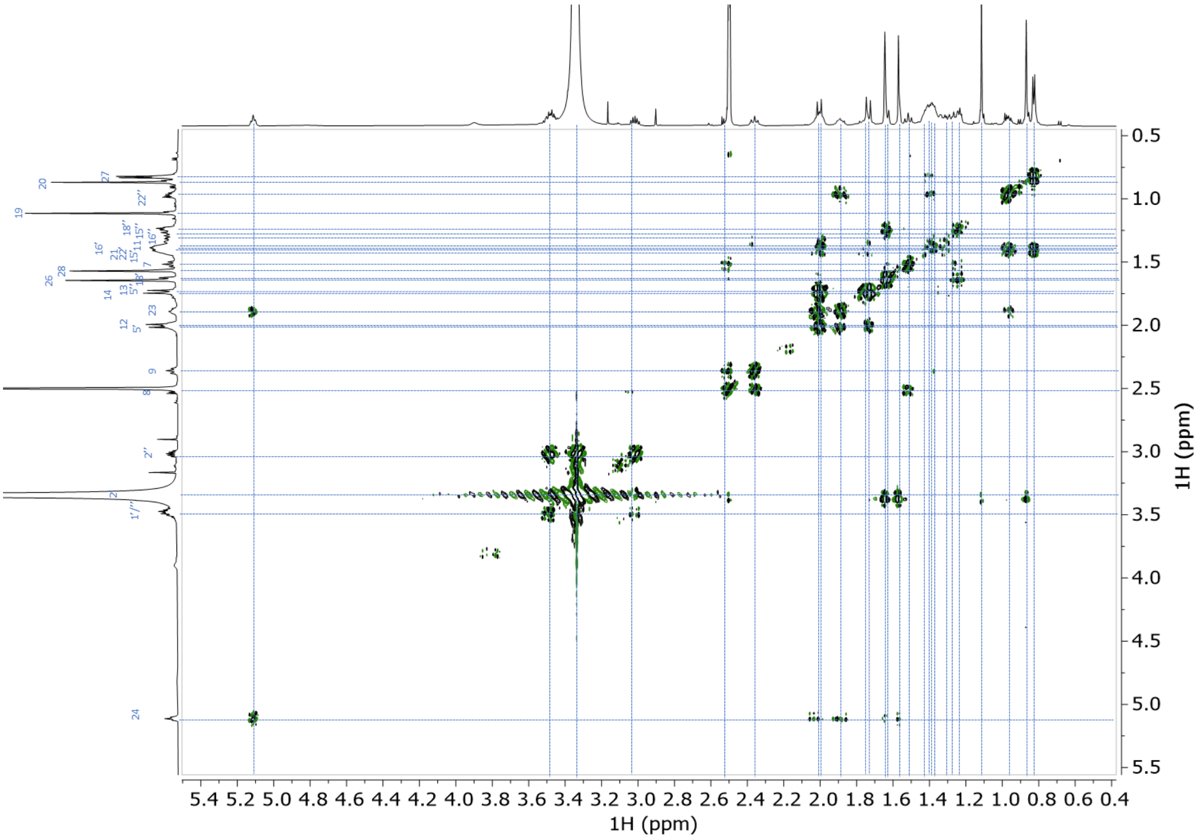

Figure S 8585. DQF-COSY spectrum of **7**.

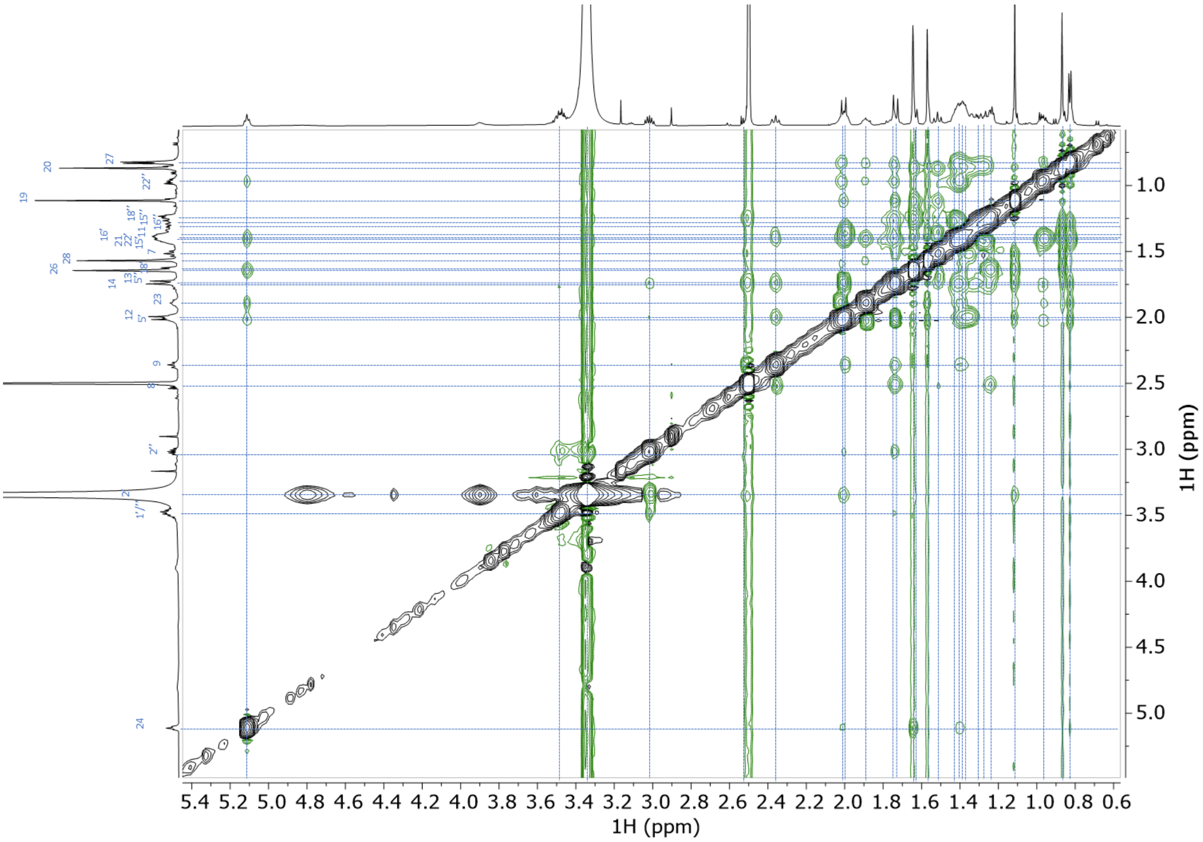

Figure S 8686. ROESY spectrum of **7**.

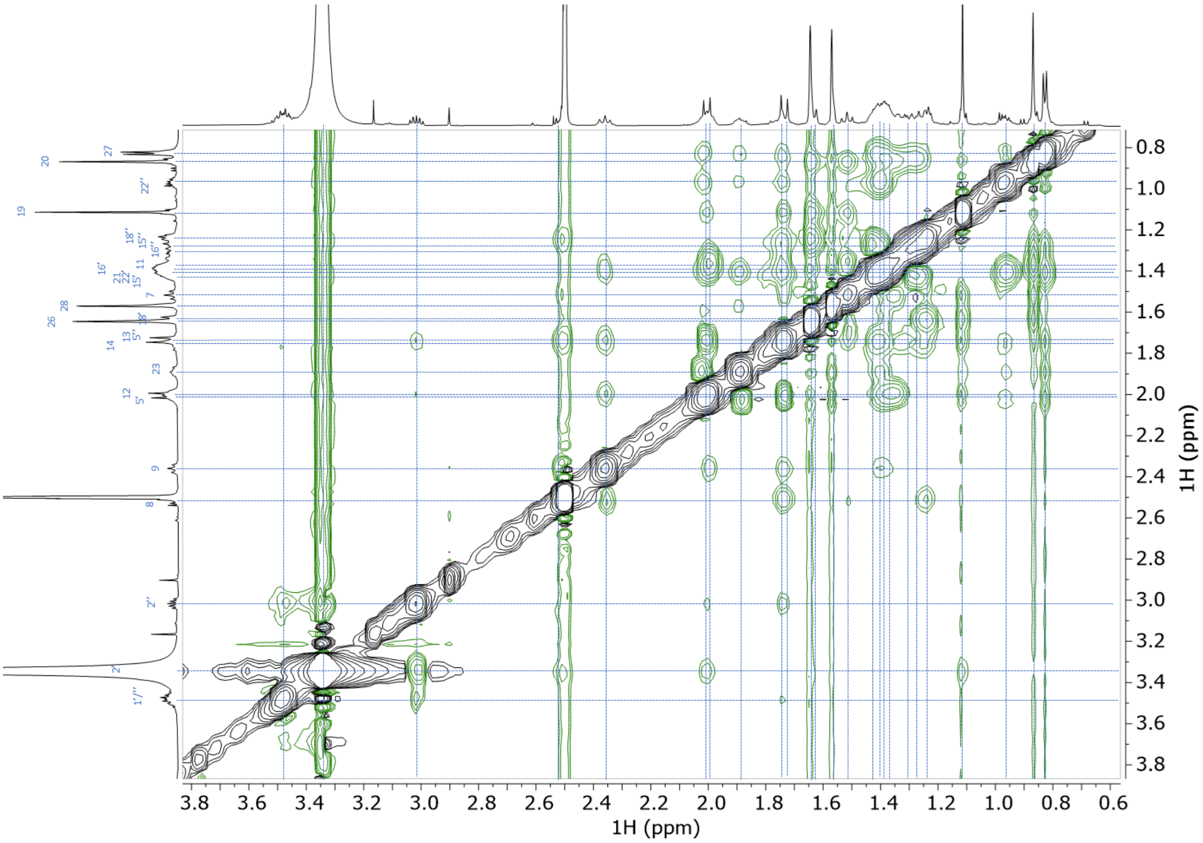

Figure S 8787. ROESY (Expanded) spectrum of **7**.

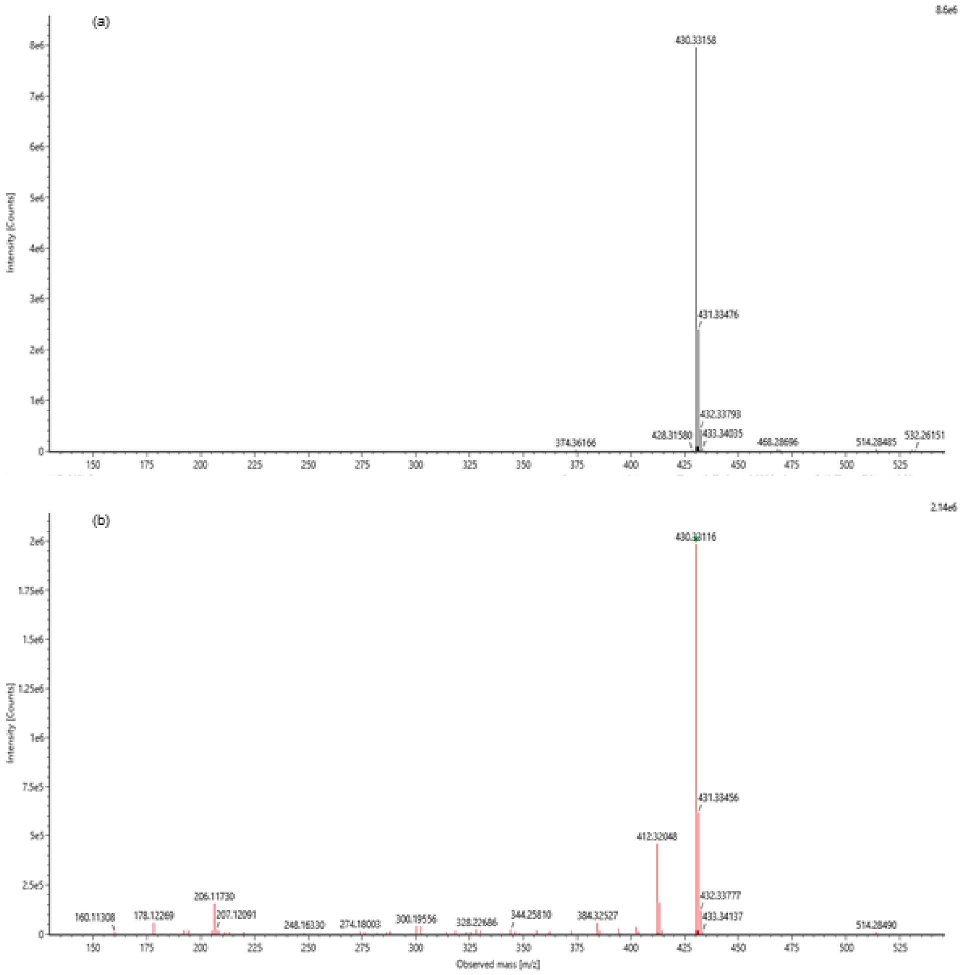

Figure S 8888. Low-collision (top) and High-collision (bottom) energy mass spectra of **8** in ESI^{+} mode.

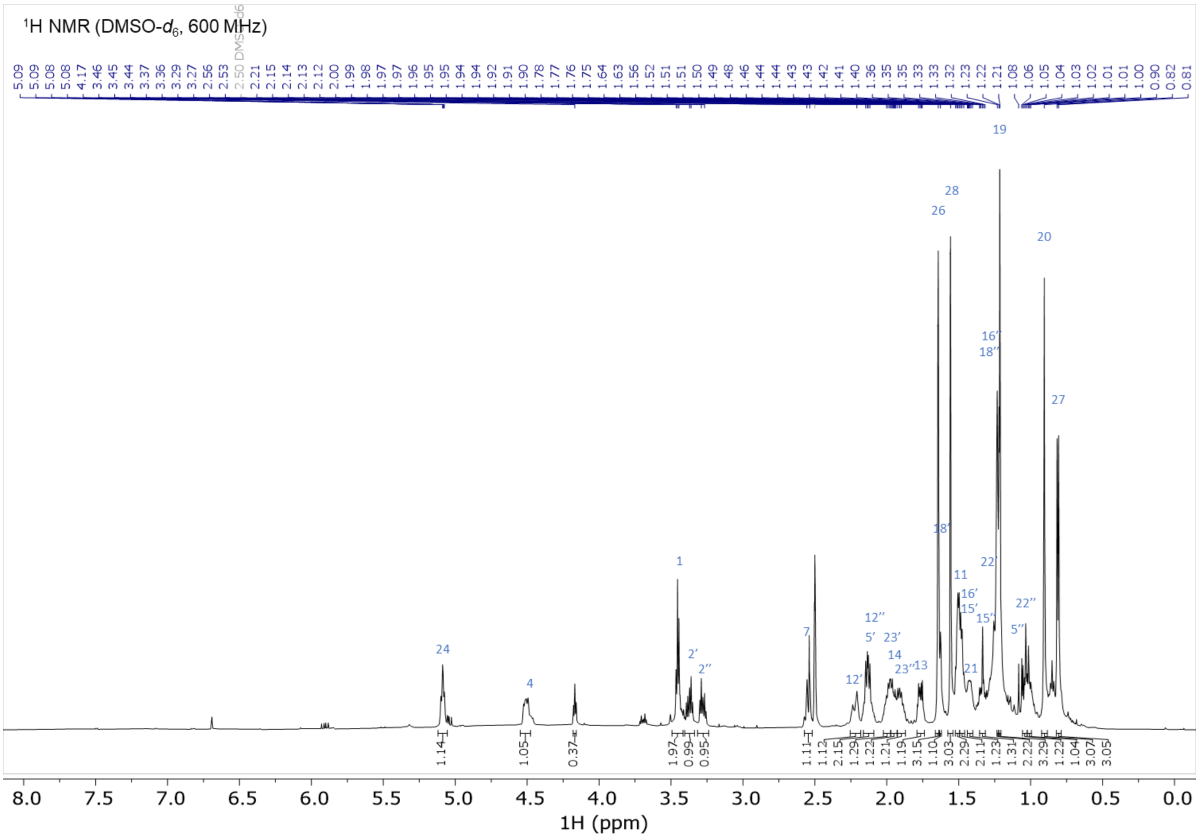

Figure S 8989. ^{1}H NMR spectrum of **8**.

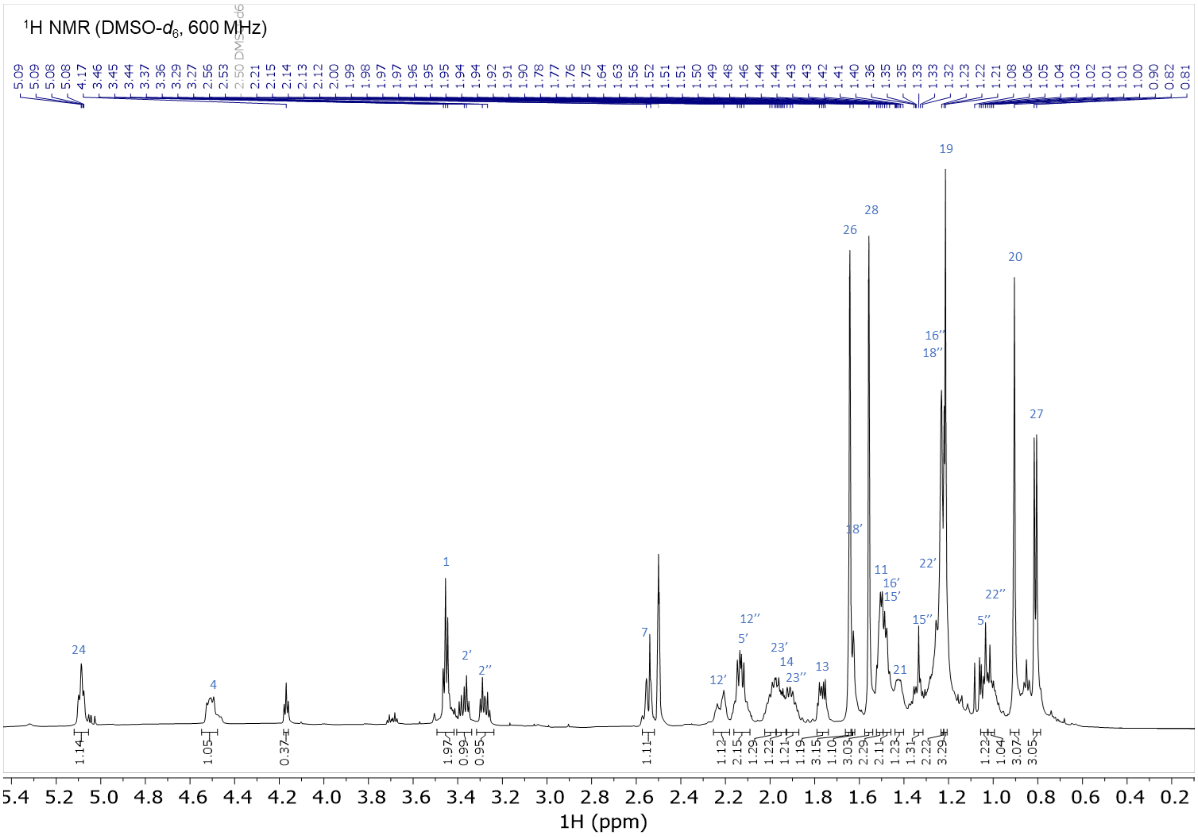

Figure S 9090. ^{1}H NMR spectrum of **8**.

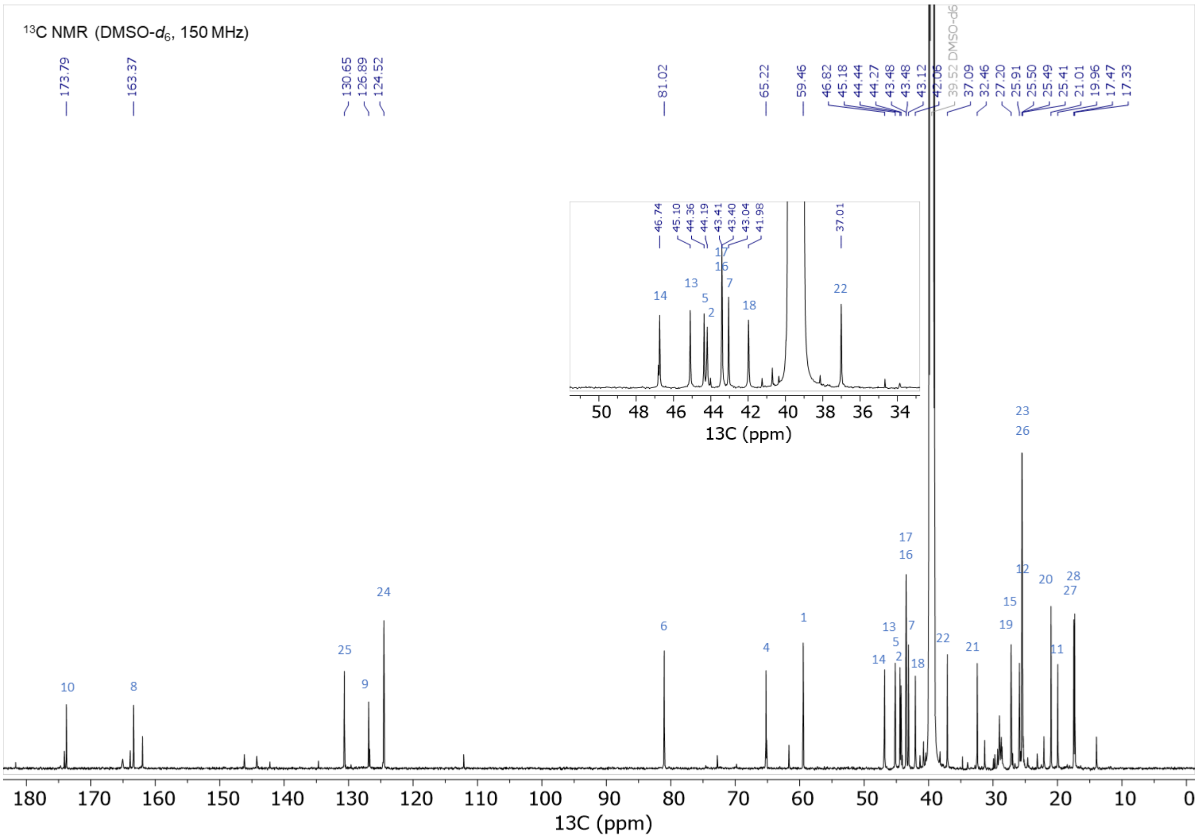

Figure S 9191. ^{13}C NMR spectrum of **8**.

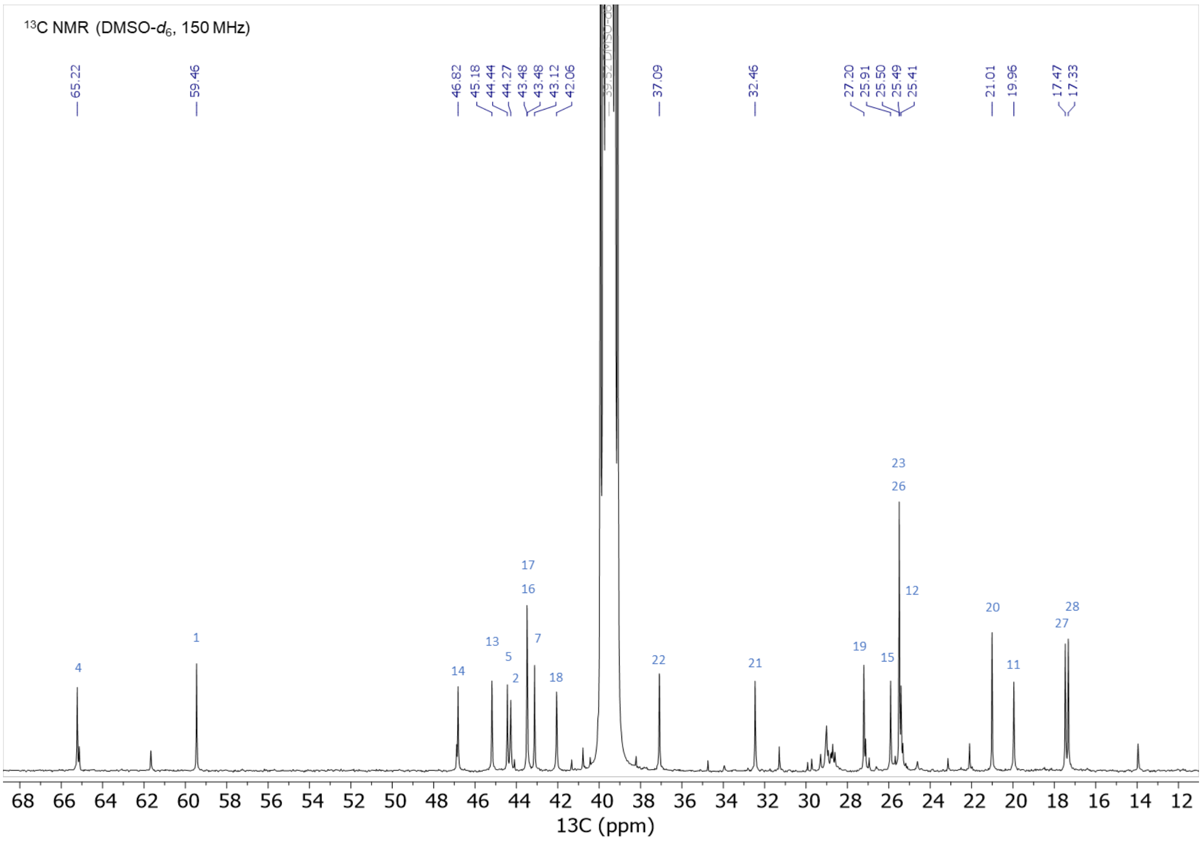

Figure S 9292. ^{13}C NMR spectrum of **8**.

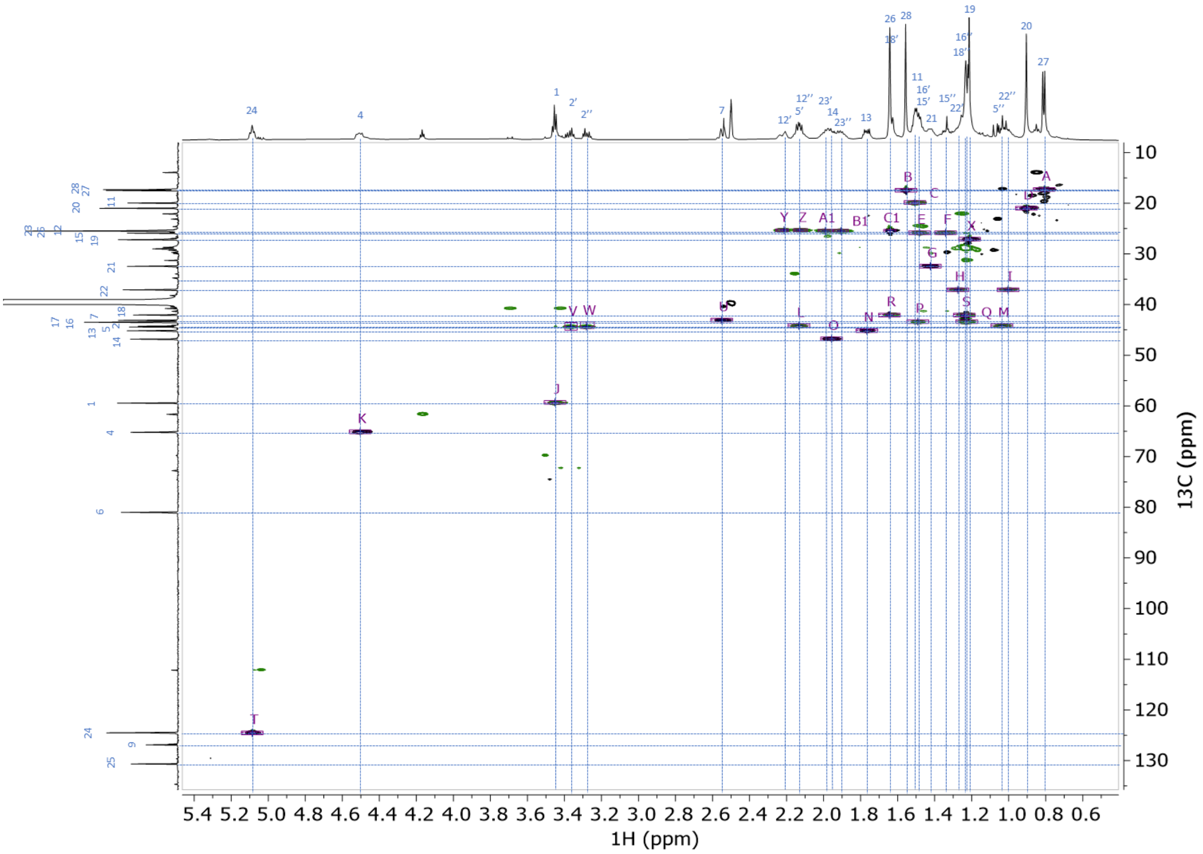

Figure S 9393. HSQC spectrum of **8**.

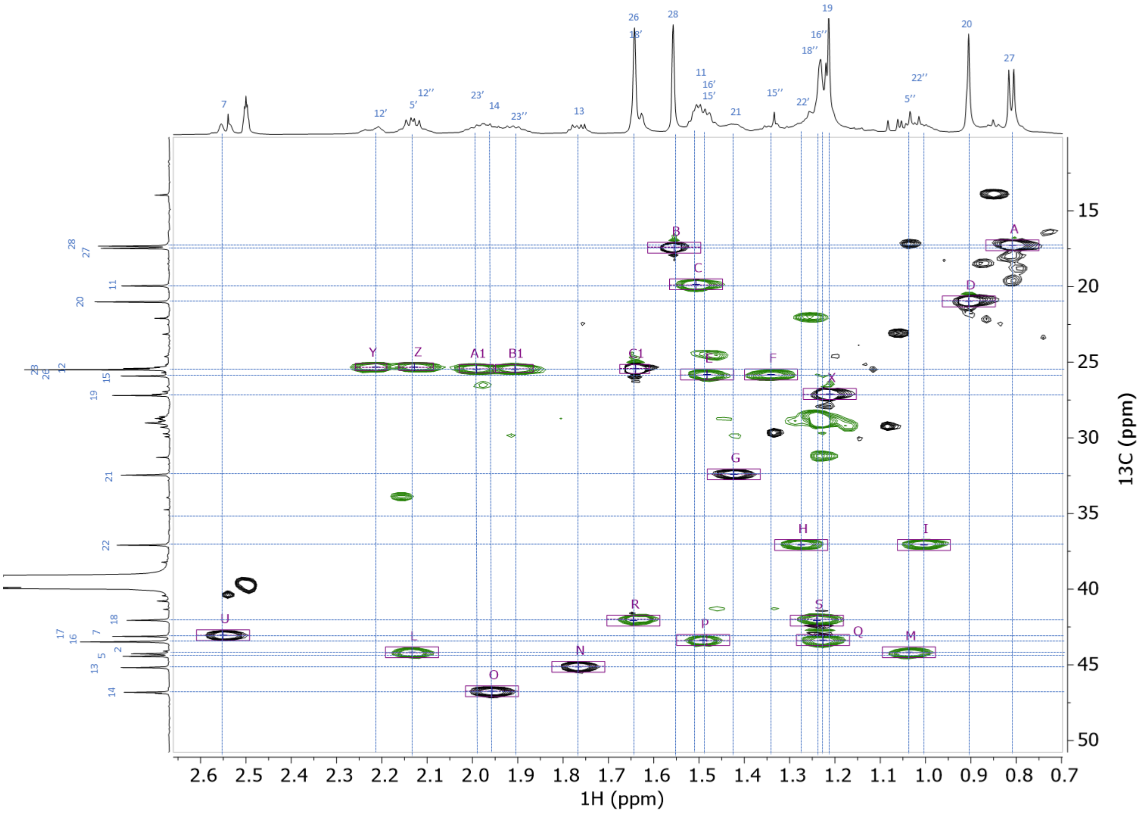

Figure S 9494. HSQC (Expanded) spectrum of **8**.

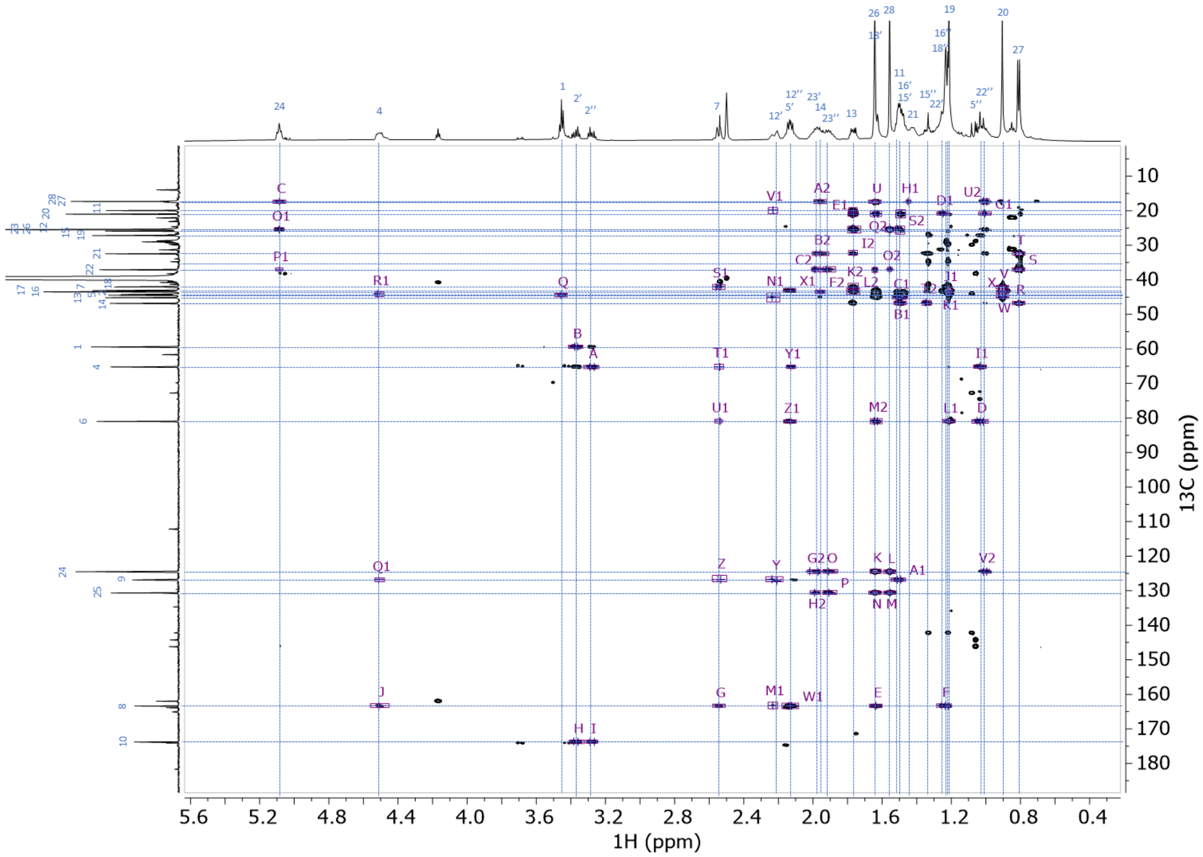

Figure S 9595. HMBC spectrum of **8**.

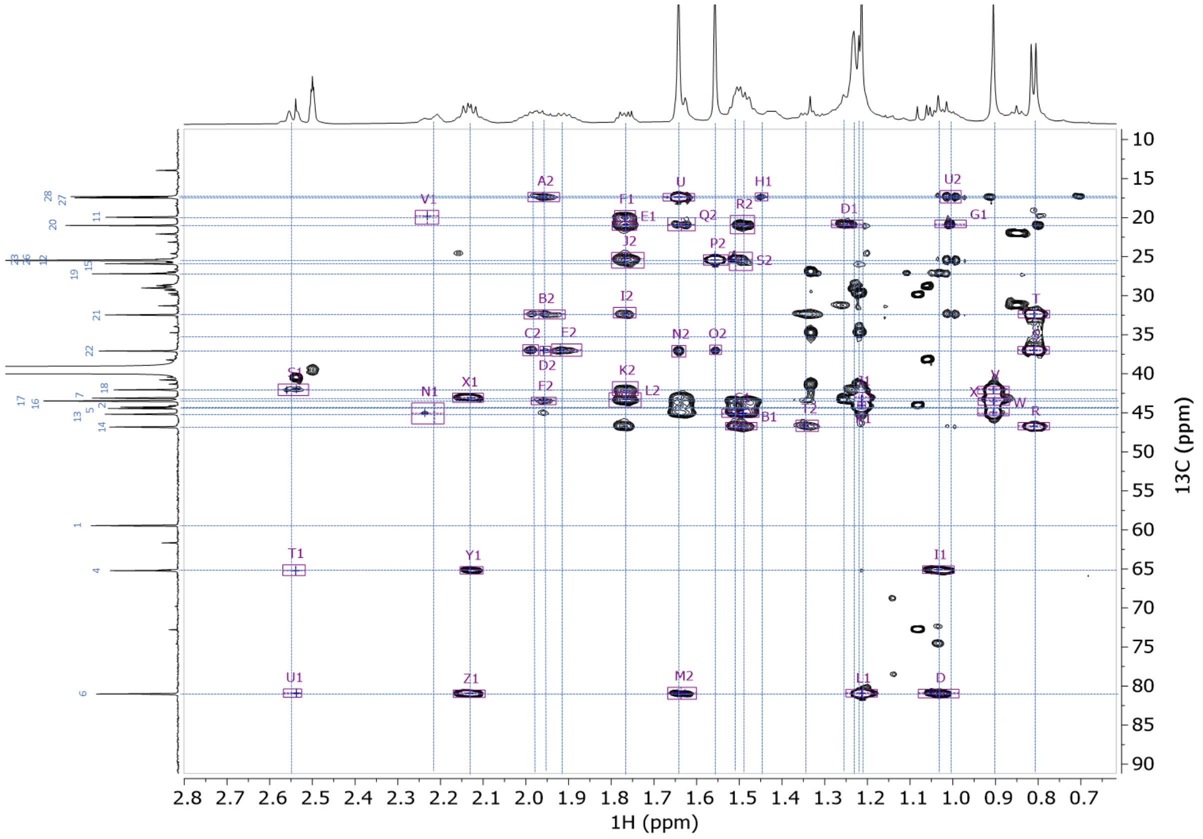

Figure S 9696. HMBC (Expanded) spectrum of **8**.

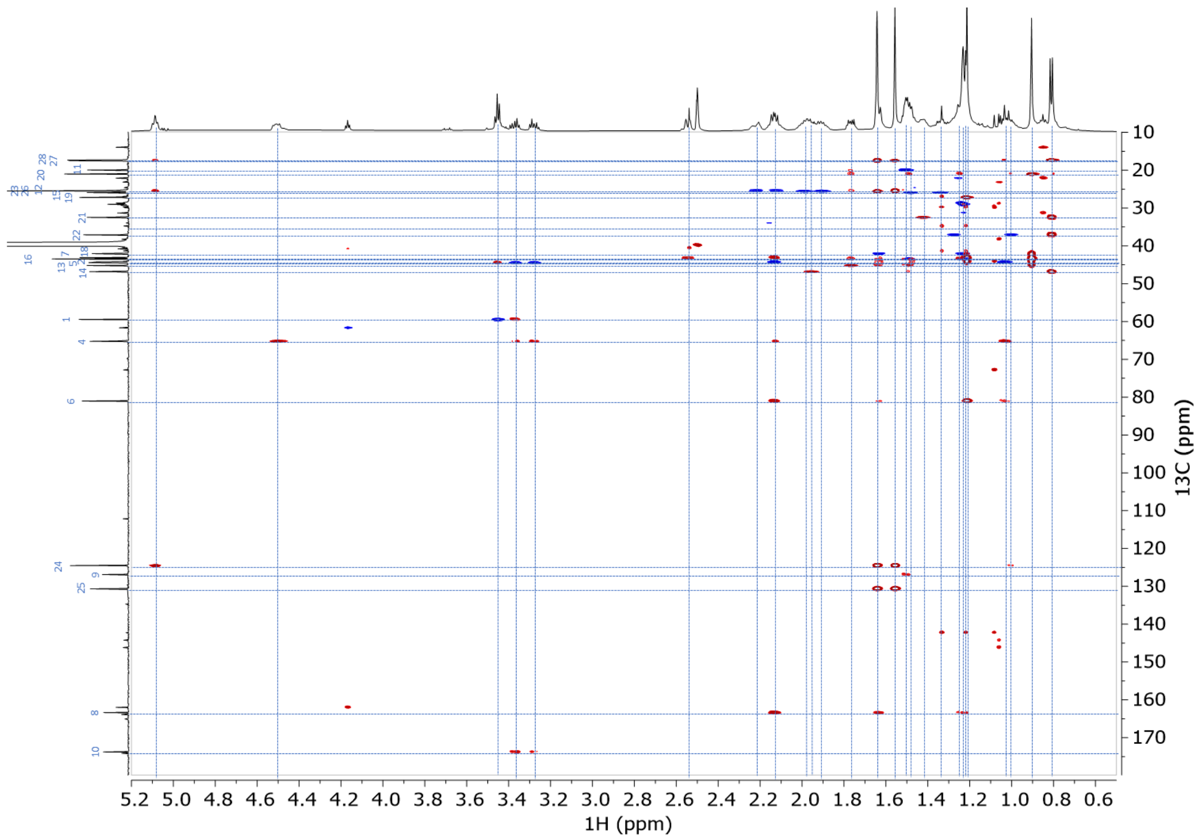

Figure S 9797. HSQC+ HMBC spectrum of **8**.

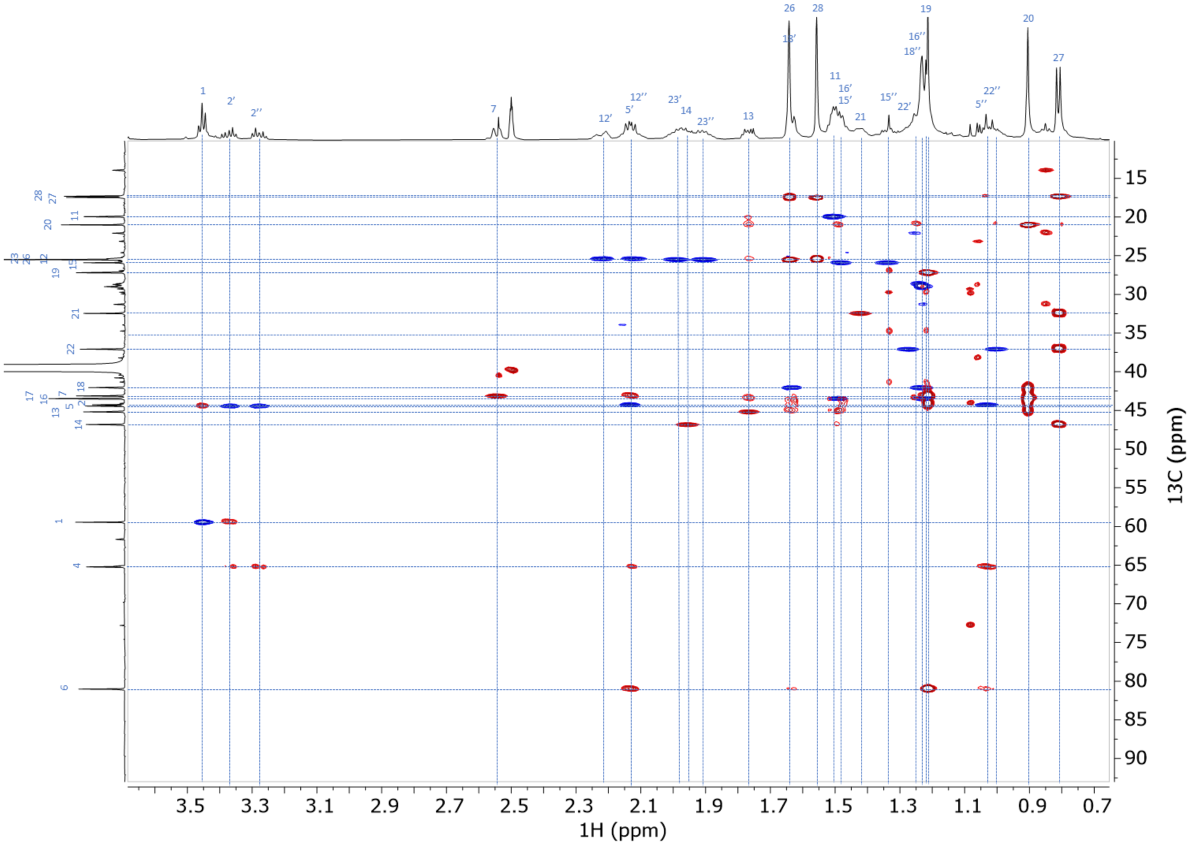

Figure S 9898. HSQC+ HMBC (Expanded1) spectrum of **8**.

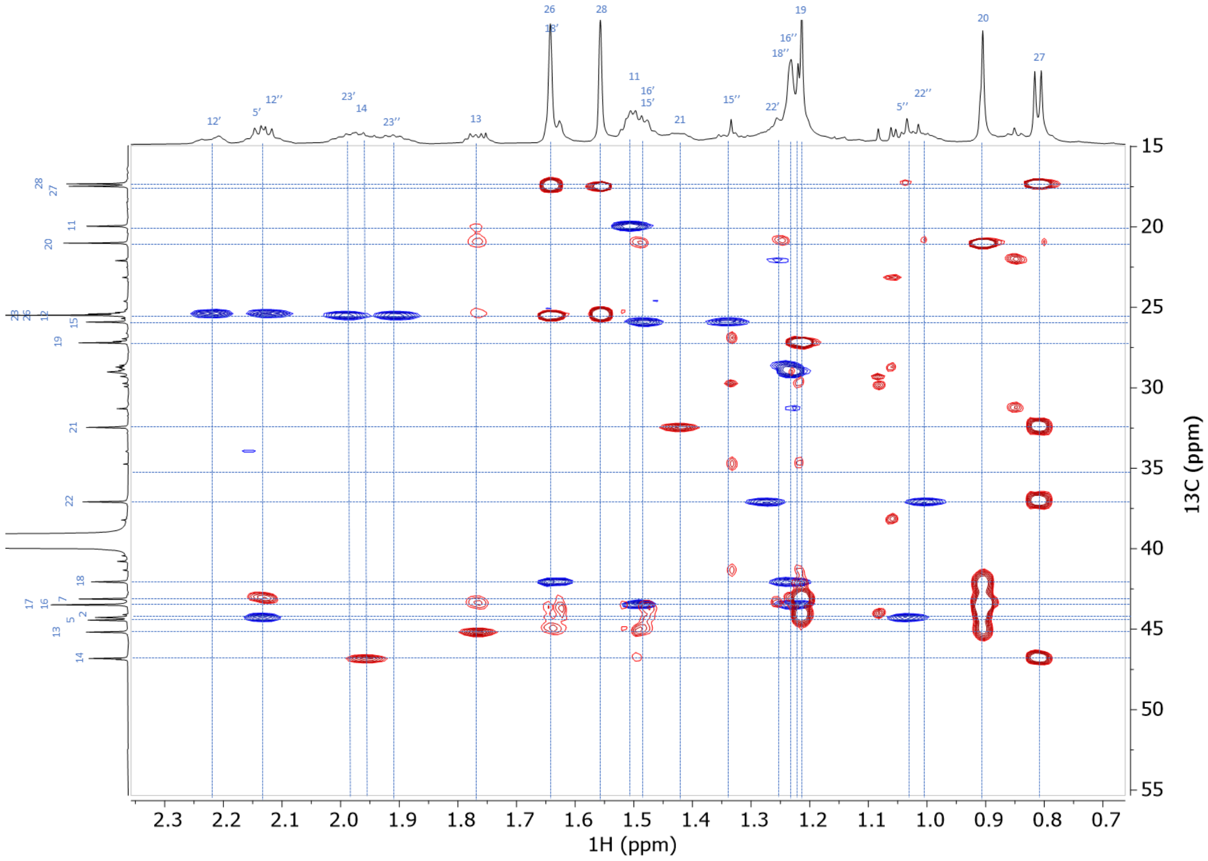

Figure S 9999. HSQC+ HMBC (Expanded2) spectrum **8**.

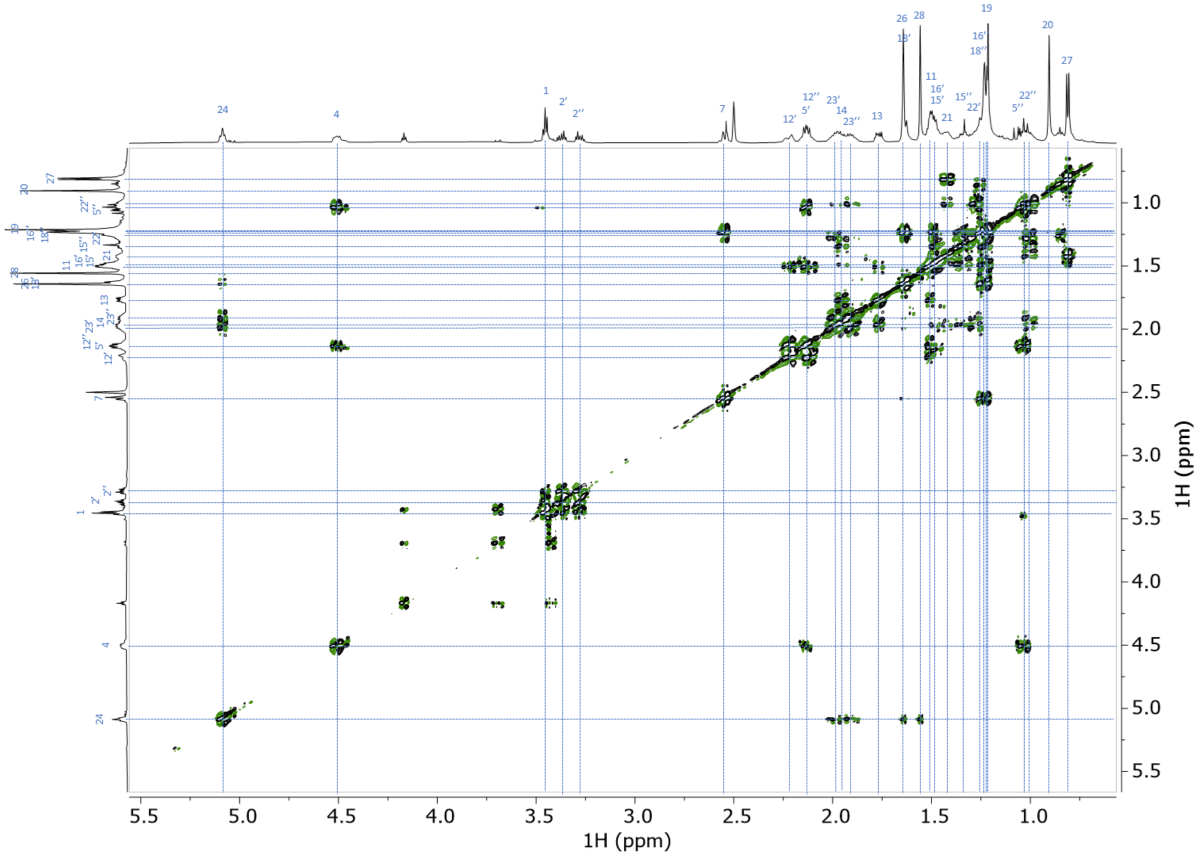

Figure S 100100. DQF-COSY spectrum of **8**.

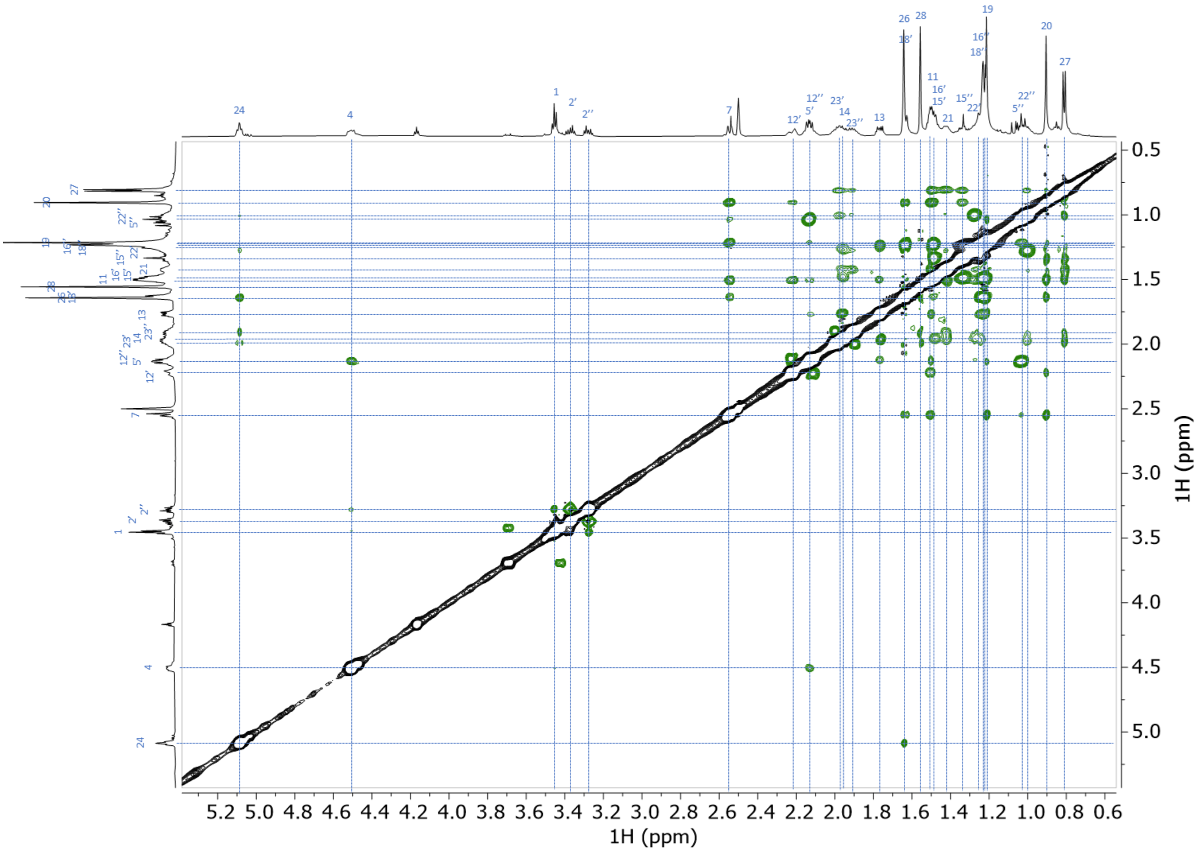

Figure S 101101. ROESY spectrum of **8**.

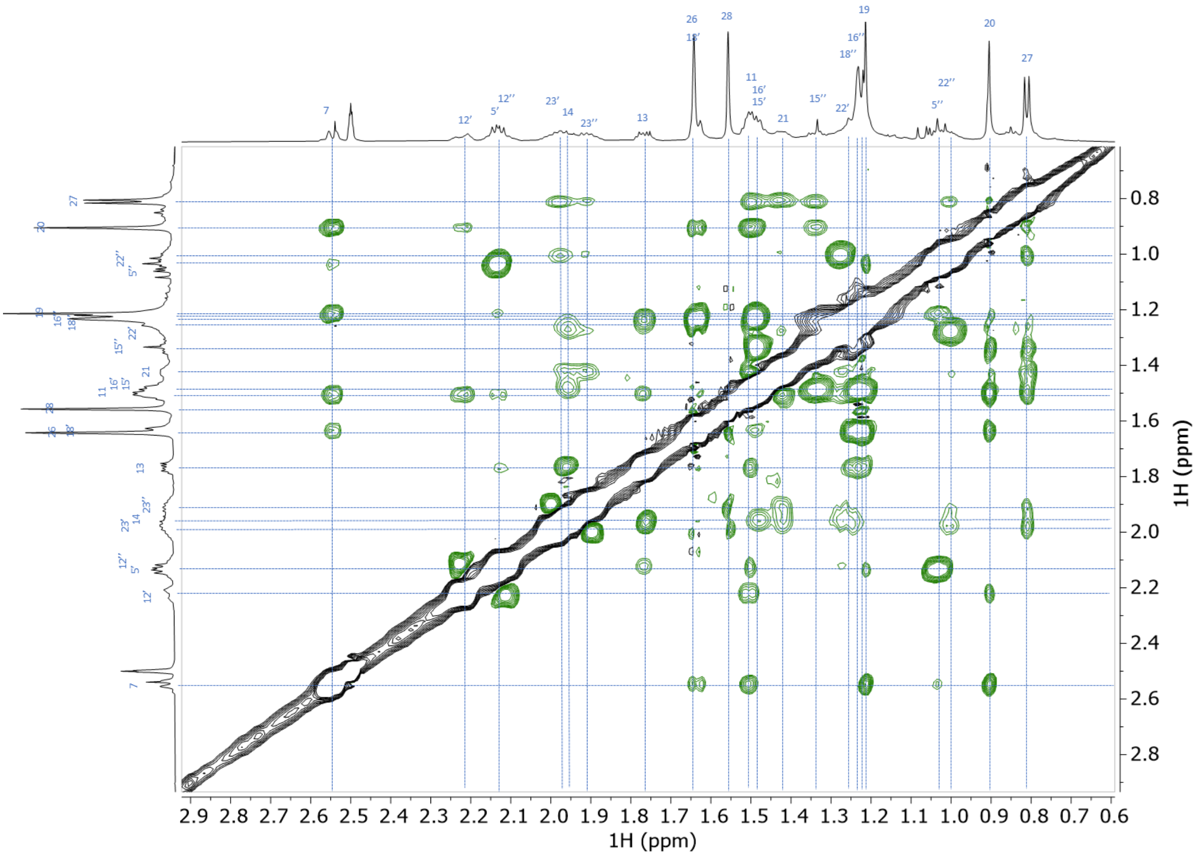

Figure S 102102. ROESY (Expanded) spectrum of **8**.

Measurement of UV and ECD

UV-Vis absorption and ECD spectra were measured using a JASCO J-815 spectropolarimeter in MeOH in a quartz cell with a 0.2 cm optical path length. The spectra were recorded in the 190-320 nm range using 0.5 nm resolution, 4 s response time, and a scanning speed of 50 nm/min. In total, three accumulations were averaged. Finally, the solvent spectra acquired at identical conditions were subtracted from those of the samples.

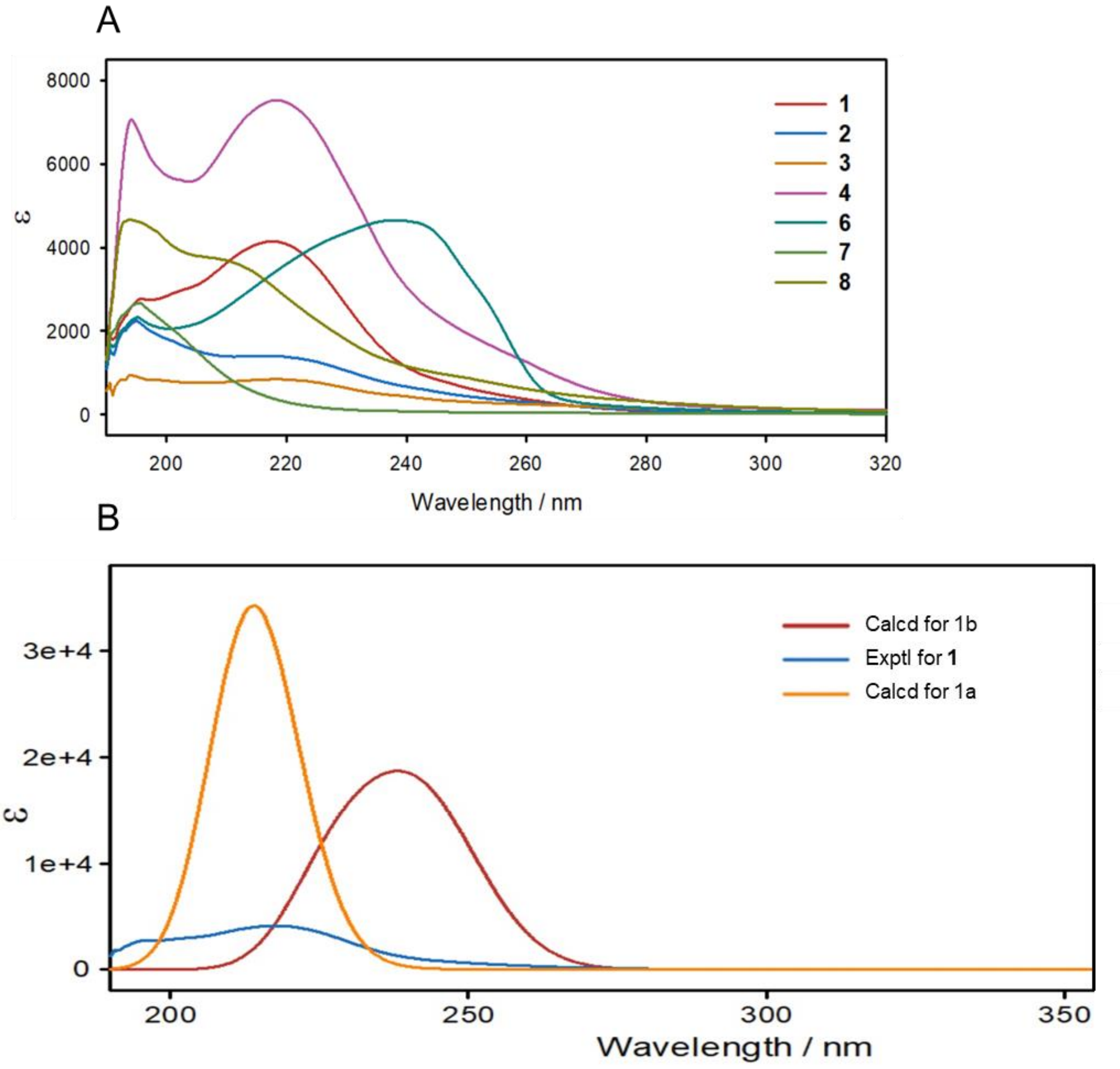

Figure S 103103. (A) Experimental UV-Vis absorption spectra of compounds **1**–**8**. (B) Calculated and Experimental UV-Vis absorption spectra of compound **1**. The calculated transition energies were lower than the experimental ones, therefore the calculated energies were multiplied by a scaling factor of 0.82.

Computational details for ECD calculations:

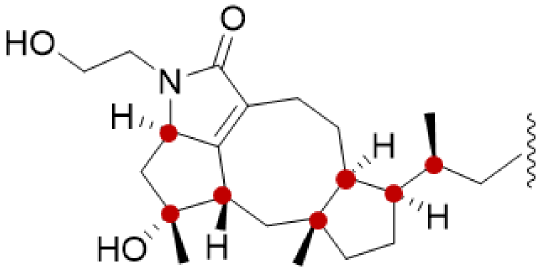

1a

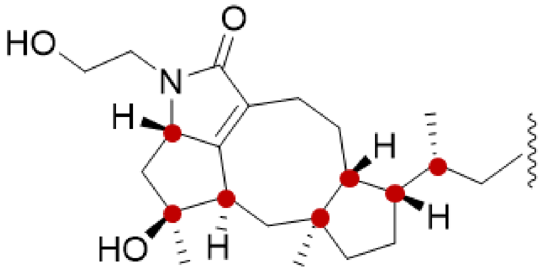

ent-1a

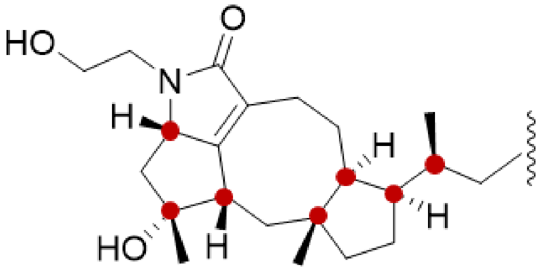

1b

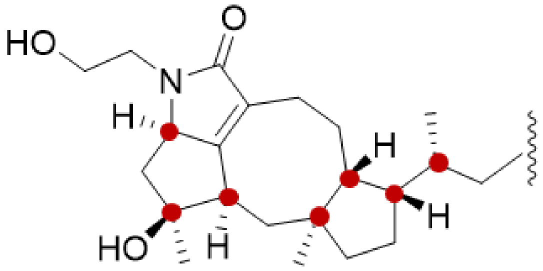

ent-1b

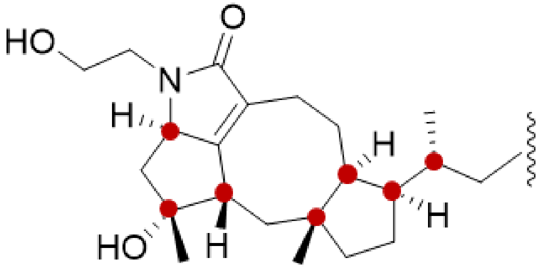

1c

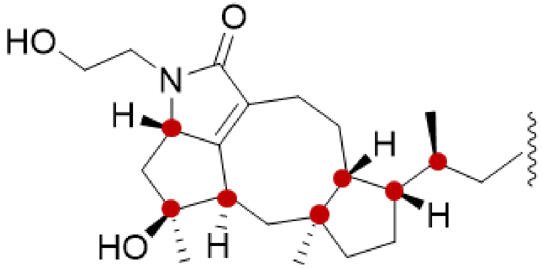

ent-1c

Figure S 104104. Structures (1a and 1b) used for ECD calculations and their enantiomers (*ent*-1a and *ent*-1b).

Table S 22. Geometry-optimized conformers of diastereomer 1a at wB97XD/6-311++G**/PCM(MeOH) level.

1a-01	1a-02	1a-03	1a-04
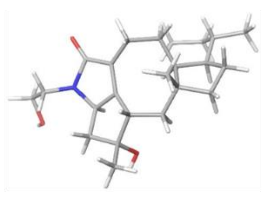	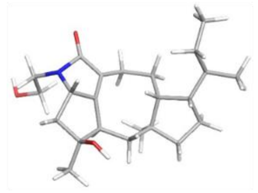	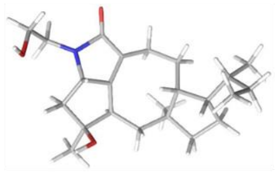	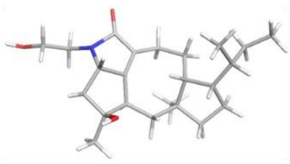
1a-05	1a-06	1a-07	1a-08
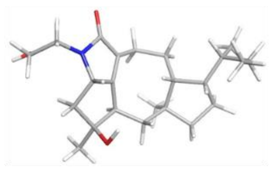	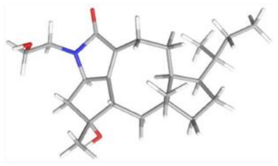	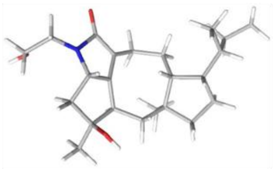	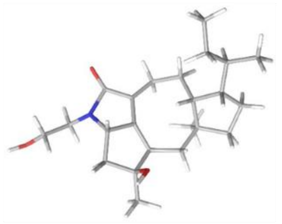

The conformational search provided eight conformers for 1a, of which only three (conf. 1a-02: 71.7%, conf. 1a-03: 6.6%, and conf. 1a-08: 19.1%) significantly contributed to the final spectra (Table S 22). The energies of the conformers and their Boltzmann weights used for calculating UV-Vis and ECD spectra are listed below (Table S 33. Gibbs free energy and Boltzmann population of eight geometry-optimized conformers of 1aTable S 33).

Table S 33. Gibbs free energy and Boltzmann population of eight geometry-optimized conformers of 1a.

Conformers	Energy (Hartree)	Boltzmann population (%)	No. of imaginary frequencies
1a-01	-1178.64628300	1.7520260	0
1a-02	-1178.64978800	71.7343200	0
1a-03	-1178.64754000	6.6331502	0
1a-04	-1178.64353400	0.0952990	0
1a-05	-1178.64421500	0.1960323	0
1a-06	-1178.64426800	0.2073509	0
1a-07	-1178.64445600	0.2530350	0
1a-08	-1178.64854000	19.1287870	0

Table S 44. Atomic coordinates for the geometry-optimized conformers of 1a (1a-01 to 04).

	1a-01			1a-02			1a-03			1a-04		
C	-1.441	-0.967	0.297	-0.729	-1.784	-0.012	-0.215	1.834	-0.238	0.374	1.339	-1.100
C	-0.817	-1.953	1.242	0.575	-2.360	-0.494	0.361	2.183	1.102	-0.300	2.397	-0.261
C	0.727	-1.965	1.353	1.543	-1.411	-1.231	0.535	1.032	2.121	-1.294	1.953	0.836
C	1.437	-0.649	1.695	1.978	-0.119	-0.527	1.389	-0.177	1.718	-0.851	0.864	1.818
C	1.358	0.332	0.458	0.775	0.908	-0.508	0.636	-1.032	0.621	-0.799	-0.527	1.062
C	0.617	1.651	0.678	0.238	1.317	0.865	1.294	-1.127	-0.756	0.549	-1.245	1.022
C	-0.887	1.534	0.986	-0.376	0.192	1.720	1.392	0.193	-1.548	1.696	-0.497	0.319
C	-1.646	0.449	0.285	-1.141	-0.886	1.016	0.271	1.173	-1.410	1.360	0.322	-0.891
C	-2.855	0.695	-0.361	-2.396	-1.297	1.472	-0.333	1.771	-2.515	2.167	0.272	-2.027
N	-3.433	-0.668	-0.589	-2.545	-2.671	0.860	-1.087	2.930	-1.939	1.757	1.493	-2.805
C	-2.394	-1.667	-0.655	-1.893	-2.681	-0.429	-1.418	2.702	-0.553	0.358	1.758	-2.572
C	-2.681	-2.999	0.032	-1.241	-3.977	-0.892	-1.257	3.892	0.390	-0.099	3.211	-2.558
C	-1.352	-3.330	0.731	0.129	-3.543	-1.427	-0.625	3.268	1.645	-1.009	3.292	-1.323
O	-3.511	1.683	-0.711	-3.269	-0.893	2.252	-0.343	1.607	-3.739	3.080	-0.436	-2.470
C	-1.505	-4.359	1.841	1.139	-4.680	-1.460	0.058	4.294	2.540	-1.246	4.720	-0.853
O	-0.429	-3.806	-0.256	-0.142	-3.089	-2.756	-1.651	2.587	2.377	-2.255	2.660	-1.637
H	-1.216	-1.800	2.257	1.129	-2.800	0.350	1.345	2.661	0.976	0.468	3.021	0.221
C	2.956	-0.917	1.860	3.060	0.587	-1.388	1.501	-1.138	2.936	-1.953	0.674	2.902
C	3.641	0.302	1.259	2.776	2.075	-1.236	1.473	-2.538	2.338	-2.030	-0.830	3.128
C	2.835	0.502	-0.029	1.256	2.112	-1.382	0.347	-2.400	1.314	-1.962	-1.349	1.694
C	0.930	-0.095	3.035	2.619	-0.436	0.832	2.817	0.258	1.355	0.432	1.296	2.546
H	0.807	-0.192	-0.327	-0.064	0.435	-1.026	-0.335	-0.556	0.444	-1.059	-0.339	0.016
C	3.202	1.727	-0.894	0.574	3.466	-1.150	0.045	-3.631	0.438	-2.066	-2.875	1.517
C	2.785	1.561	-2.368	-0.907	3.403	-1.566	-0.884	-4.615	1.173	-1.028	-3.733	2.263
C	4.714	1.989	-0.860	1.295	4.585	-1.908	1.298	-4.373	-0.035	-2.170	-3.257	0.036
C	1.292	1.521	-2.694	-1.768	4.518	-0.981	-2.329	-4.141	1.311	-1.545	-5.135	2.589
O	-4.960	-2.420	-2.325	-1.379	-4.928	3.563	-3.478	4.571	-2.025	3.660	3.987	-4.662
C	-5.460	-1.179	-1.862	-1.281	-3.871	2.638	-2.647	4.374	-3.152	2.632	3.028	-4.504
C	-4.847	-0.893	-0.493	-2.576	-3.763	1.794	-1.214	4.160	-2.664	2.774	2.474	-3.091
H	-2.022	-1.789	-1.687	-2.556	-2.227	-1.177	-2.399	2.208	-0.450	-0.258	1.108	-3.207
H	1.134	-2.327	0.408	1.085	-1.110	-2.178	-0.456	0.663	2.391	-2.208	1.610	0.349
H	0.984	-2.713	2.114	2.441	-1.995	-1.476	0.964	1.476	3.029	-1.558	2.854	1.404
H	0.708	2.241	-0.233	-0.549	2.058	0.706	0.705	-1.825	-1.357	0.402	-2.181	0.482
H	1.095	2.242	1.468	1.011	1.821	1.455	2.299	-1.555	-0.686	0.880	-1.525	2.029
H	-1.365	2.484	0.732	-1.052	0.648	2.448	1.491	-0.049	-2.611	2.443	-1.234	0.007
H	-1.039	1.413	2.062	0.404	-0.290	2.316	2.319	0.710	-1.282	2.212	0.146	1.035
H	-2.999	-3.791	-0.648	-1.809	-4.500	-1.661	-2.190	4.414	0.615	-0.625	3.512	-3.467
H	-3.451	-2.872	0.795	-1.090	-4.664	-0.058	-0.553	4.613	-0.032	0.742	3.892	-2.417
H	-2.196	-4.003	2.609	1.319	-5.070	-0.455	0.850	4.816	1.998	-0.304	5.199	-0.577
H	-0.538	-4.562	2.309	2.096	-4.339	-1.866	0.496	3.805	3.414	-1.912	4.731	0.013
H	-1.895	-5.300	1.440	0.770	-5.493	-2.089	-0.664	5.039	2.889	-1.709	5.311	-1.647
H	-0.793	-4.608	-0.637	0.687	-2.871	-3.188	-2.303	3.239	2.642	-2.678	3.166	-2.334
H	3.248	-1.804	1.284	2.940	0.313	-2.443	0.634	-1.014	3.595	-2.919	1.027	2.521
H	3.226	-1.104	2.902	4.070	0.295	-1.090	2.394	-0.931	3.531	-1.733	1.246	3.807
H	4.705	0.139	1.083	3.298	2.681	-1.980	1.289	-3.321	3.080	-2.938	-1.145	3.651

H	3.541	1.175	1.917	3.076	2.435	-0.243	2.425	-2.764	1.845	-1.170	-1.172	3.715
H	3.055	-0.380	-0.651	1.047	1.840	-2.428	-0.557	-2.218	1.908	-2.871	-0.961	1.209
H	1.419	0.848	3.295	1.967	-1.001	1.498	3.444	-0.600	1.098	0.803	0.514	3.215
H	1.161	-0.815	3.826	2.929	0.474	1.354	3.269	0.752	2.220	0.211	2.177	3.156
H	-0.146	0.066	3.061	3.513	-1.045	0.670	2.859	0.964	0.526	1.238	1.576	1.870
H	2.715	2.621	-0.488	0.623	3.709	-0.082	-0.504	-3.286	-0.448	-3.037	-3.124	1.971
H	3.259	0.646	-2.746	-1.334	2.439	-1.269	-0.465	-4.832	2.163	-0.744	-3.240	3.198
H	3.229	2.386	-2.937	-0.958	3.427	-2.663	-0.882	-5.566	0.628	-0.112	-3.821	1.674
H	5.064	2.232	0.145	2.304	4.749	-1.522	2.023	-3.711	-0.511	-2.982	-2.707	-0.450
H	4.970	2.829	-1.514	0.755	5.532	-1.826	1.028	-5.150	-0.757	-2.370	-4.324	-0.090
H	5.272	1.112	-1.208	1.378	4.338	-2.973	1.797	-4.864	0.807	-1.247	-3.028	-0.506
H	1.149	1.402	-3.772	-2.806	4.432	-1.311	-2.772	-3.958	0.327	-0.784	-5.724	3.108
H	0.794	2.448	-2.399	-1.410	5.509	-1.277	-2.405	-3.212	1.885	-1.825	-5.681	1.684
H	0.776	0.693	-2.203	-1.761	4.477	0.114	-2.941	-4.890	1.820	-2.428	-5.085	3.234
H	-5.200	-2.523	-3.248	-1.934	-4.655	4.296	-4.394	4.530	-2.306	3.642	4.313	-5.563
H	-6.549	-1.210	-1.740	-0.436	-4.095	1.985	-2.648	5.255	-3.806	2.734	2.208	-5.223
H	-5.205	-0.367	-2.551	-1.086	-2.913	3.132	-2.966	3.503	-3.735	1.644	3.484	-4.640
H	-5.049	-1.740	0.167	-3.410	-3.554	2.469	-0.930	5.002	-2.028	2.736	3.290	-2.359
H	-5.308	0.005	-0.079	-2.766	-4.700	1.271	-0.548	4.118	-3.528	3.738	1.975	-2.986

Table S 55. Atomic coordinates for the geometry-optimized conformers of 1a (1a-05 to 08).

	1a-05			1a-06			1a-07			1a-08		
C	0.659	0.962	-1.486	-0.824	0.168	1.685	0.756	0.453	-1.754	0.368	-1.126	-1.216
C	0.092	-0.024	-2.447	-1.532	-1.144	1.636	2.161	0.270	-1.298	1.864	-0.931	-1.197
C	-0.171	-1.450	-1.907	-1.932	-1.675	0.241	2.445	0.571	0.190	2.445	0.280	-0.434
C	-1.194	-1.605	-0.772	-0.808	-1.945	-0.770	1.732	-0.289	1.248	1.980	0.521	1.007
C	-0.654	-0.971	0.571	-0.148	-0.591	-1.260	0.179	0.017	1.278	0.482	1.027	0.992
C	-1.371	0.282	1.083	1.304	-0.342	-0.840	-0.751	-1.098	0.788	-0.546	0.171	1.733
C	-1.226	1.551	0.216	1.561	-0.177	0.672	-0.641	-1.477	-0.705	-0.735	-1.265	1.212
C	0.091	1.761	-0.455	0.523	0.565	1.447	-0.411	-0.352	-1.659	-0.642	-1.488	-0.267
C	0.849	2.920	-0.327	0.801	1.672	2.243	-1.232	-0.069	-2.745	-1.568	-2.301	-0.922
N	1.898	2.813	-1.413	-0.453	1.880	3.061	-0.441	0.927	-3.570	-0.902	-2.607	-2.236
C	1.996	1.473	-1.934	-1.578	1.170	2.508	0.651	1.498	-2.824	-0.092	-1.479	-2.631
C	1.928	1.293	-3.461	-2.387	0.276	3.460	2.053	1.456	-3.463	1.194	-1.761	-3.399
C	1.136	-0.023	-3.626	-2.780	-0.907	2.552	2.973	1.221	-2.256	2.230	-0.859	-2.711
O	0.848	3.926	0.381	1.751	2.431	2.432	-2.332	-0.422	-3.165	-2.664	-2.821	-0.677
C	0.468	-0.136	-4.985	-3.179	-2.150	3.337	4.325	0.635	-2.642	3.665	-1.272	-3.009
O	2.037	-1.131	-3.556	-3.857	-0.494	1.706	3.150	2.521	-1.686	2.008	0.491	-3.133
H	-0.864	0.350	-2.843	-0.911	-1.918	2.113	2.485	-0.764	-1.488	2.344	-1.837	-0.794
C	-1.344	-3.116	-0.442	-1.435	-2.548	-2.058	2.232	0.144	2.653	2.773	1.715	1.608
C	-1.616	-3.163	1.055	-0.569	-2.020	-3.193	1.034	-0.063	3.568	1.759	2.499	2.429
C	-0.583	-2.165	1.580	-0.417	-0.552	-2.799	-0.096	0.535	2.729	0.548	2.502	1.497
C	-2.579	-1.105	-1.209	0.178	-2.992	-0.230	2.097	-1.772	1.085	2.279	-0.711	1.875
H	0.381	-0.656	0.390	-0.722	0.228	-0.814	0.002	0.860	0.602	0.155	1.041	-0.053
C	-0.611	-1.871	3.091	0.434	0.304	-3.758	-1.495	0.468	3.371	-0.745	3.114	2.048
C	0.138	-2.959	3.882	1.902	-0.123	-3.952	-2.053	-0.937	3.674	-1.803	3.235	0.937
C	-2.025	-1.713	3.662	0.308	1.798	-3.444	-2.508	1.335	2.615	-0.479	4.480	2.689
C	1.657	-2.927	3.722	2.443	0.240	-5.333	-2.985	-0.949	4.885	-3.228	3.420	1.451

O	1.155	4.786	-3.326	0.413	1.059	5.644	0.313	-0.574	-5.870	-0.605	-5.662	-4.202
C	2.524	4.468	-3.175	-0.608	2.034	5.552	0.127	0.823	-6.000	-0.920	-4.296	-4.013
C	2.736	3.915	-1.764	-0.475	2.742	4.200	-0.830	1.282	-4.897	-0.643	-3.991	-2.545
H	2.902	0.971	-1.516	-2.251	1.868	1.962	0.403	2.533	-2.504	-0.723	-0.709	-3.095
H	0.773	-1.876	-1.553	-2.627	-0.962	-0.211	2.179	1.615	0.390	2.225	1.182	-1.008
H	-0.491	-2.065	-2.757	-2.494	-2.606	0.395	3.529	0.475	0.349	3.536	0.163	-0.437
H	-0.962	0.517	2.070	1.625	0.588	-1.312	-1.776	-0.759	0.946	-1.515	0.670	1.653
H	-2.439	0.093	1.231	1.967	-1.122	-1.225	-0.634	-2.004	1.389	-0.320	0.125	2.805
H	-1.439	2.423	0.840	2.517	0.337	0.804	-1.559	-1.996	-0.995	-1.721	-1.617	1.527
H	-1.997	1.551	-0.563	1.690	-1.159	1.137	0.164	-2.205	-0.846	-0.022	-1.933	1.703
H	2.895	1.234	-3.966	-3.267	0.752	3.906	2.342	2.359	-4.006	1.121	-1.547	-4.467
H	1.343	2.114	-3.884	-1.733	-0.089	4.255	2.115	0.591	-4.128	1.505	-2.800	-3.284
H	-0.227	0.692	-5.139	-4.051	-1.944	3.966	4.200	-0.332	-3.134	3.856	-2.290	-2.660
H	-0.087	-1.074	-5.056	-2.362	-2.478	3.983	4.945	0.485	-1.752	4.367	-0.597	-2.516
H	1.219	-0.116	-5.779	-3.432	-2.963	2.653	4.851	1.311	-3.320	3.856	-1.239	-4.085
H	2.509	-1.116	-2.720	-4.615	-0.305	2.264	3.723	2.451	-0.918	2.174	0.537	-4.077
H	-0.405	-3.642	-0.652	-2.458	-2.176	-2.189	2.498	1.208	2.649	3.147	2.359	0.804
H	-2.123	-3.584	-1.050	-1.488	-3.638	-2.007	3.122	-0.415	2.952	3.641	1.378	2.182
H	-1.504	-4.164	1.482	-1.024	-2.145	-4.180	1.139	0.430	4.537	2.111	3.499	2.688
H	-2.636	-2.823	1.269	0.400	-2.533	-3.207	0.872	-1.130	3.754	1.520	1.975	3.365
H	0.389	-2.631	1.379	-1.429	-0.131	-2.894	0.126	1.611	2.688	0.835	3.117	0.630
H	-2.607	-0.041	-1.443	0.947	-3.235	-0.968	1.654	-2.378	1.881	1.911	-0.589	2.898
H	-3.332	-1.295	-0.439	-0.369	-3.914	-0.005	3.184	-1.883	1.152	3.364	-0.850	1.927
H	-2.886	-1.642	-2.112	0.682	-2.688	0.686	1.787	-2.198	0.134	1.863	-1.633	1.473
H	-0.069	-0.933	3.262	-0.049	0.153	-4.735	-1.351	0.947	4.351	-1.141	2.456	2.832
H	-0.248	-3.944	3.592	1.997	-1.204	-3.809	-1.230	-1.635	3.857	-1.774	2.346	0.299
H	-0.102	-2.840	4.945	2.534	0.341	-3.190	-2.593	-1.324	2.805	-1.527	4.077	0.289
H	-2.550	-2.674	3.663	0.730	2.045	-2.465	-3.471	1.365	3.132	0.025	5.146	1.980
H	-2.630	-1.005	3.094	-0.743	2.106	-3.438	-2.687	0.962	1.602	0.154	4.394	3.575
H	-1.979	-1.362	4.697	0.826	2.407	-4.189	-2.147	2.365	2.528	-1.410	4.962	2.997
H	2.130	-3.703	4.328	1.876	-0.269	-6.120	-3.830	-0.268	4.748	-3.938	3.503	0.623
H	2.059	-1.960	4.041	3.493	-0.049	-5.436	-2.454	-0.639	5.791	-3.330	4.321	2.062
H	1.966	-3.088	2.686	2.378	1.315	-5.521	-3.393	-1.948	5.060	-3.530	2.566	2.065
H	1.027	5.185	-4.189	0.366	0.650	6.511	0.861	-0.877	-6.597	-0.831	-5.908	-5.101
H	3.157	5.357	-3.265	-0.500	2.804	6.324	-0.343	1.079	-6.955	-1.974	-4.095	-4.229
H	2.849	3.741	-3.927	-1.598	1.584	5.656	1.077	1.359	-5.933	-0.307	-3.658	-4.659
H	2.536	4.697	-1.031	0.462	3.302	4.176	-1.813	0.836	-5.057	0.393	-4.251	-2.295
H	3.784	3.595	-1.684	-1.321	3.436	4.100	-0.907	2.377	-4.957	-1.302	-4.588	-1.913

Table S 66. Geometry-optimized conformers of diastereomer 1b at wB97XD/6-311++G**/PCM(MeOH) level.

1b-01	1b-02	1b-03	1b-04
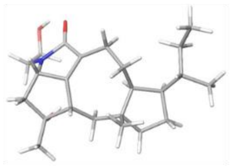	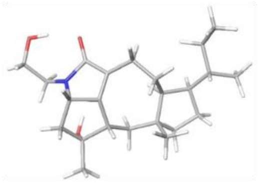	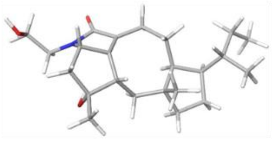	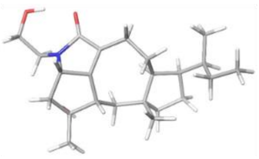
1b-05	1b-06	1b-07	1b-08
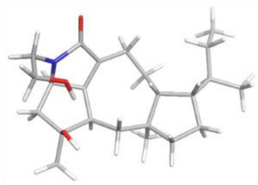	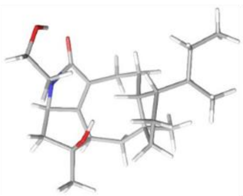	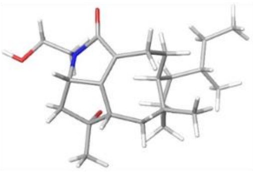	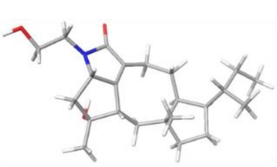
1b-09	1b-10		
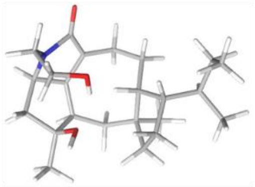	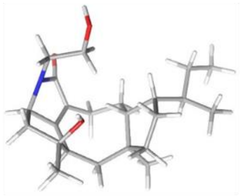		

The conformational search provided ten conformers for 1b, of which three (conf. 1b-01: 75.00%, 1b-04: 8.92%, and 1b-05:13.14%) significantly contributed to the final spectra (

Table S 66). The energies of the conformers and their Boltzmann weights used for calculating UV-Vis and ECD spectra are listed below (Table S 77).

Table S 77. Gibbs free energy and Boltzmann population of ten geometry-optimized conformers of 1b.

Conformer	Energy (Hartree)	Boltzmann population (%)	No. of imaginary frequencies

1b-01	-1178.64560000	75.007420	0
1b-02	-1178.64190100	1.4917090	1
1b-03	-1178.64153100	1.0080800	0
1b-04	-1178.64359000	8.9242010	0
1b-05	-1178.64395600	13.1497500	0
1b-06	-1178.63845300	0.0387004	0
1b-07	-1178.63695800	0.0079440	0
1b-08	-1178.64057300	0.3654660	0
1b-09	-1178.63622700	0.0036629	0
1b-10	-1178.63605900	0.0030659	0

Table S 88. Atomic coordinates for the geometry-optimized conformers of 1b (1b-01 to 04).

	1b-01			1b-02			1b-03			1b-04		
C	-0.479	0.373	-1.596	0.182	-1.430	-0.790	-0.265	1.612	0.550	0.573	1.588	-0.285
C	1.005	0.548	-1.453	0.695	-0.293	-1.628	-1.281	0.822	-0.215	-0.871	1.251	-0.502
C	1.586	1.017	-0.114	0.012	1.076	-1.539	-0.795	0.040	-1.436	-1.204	-0.095	-1.154
C	1.623	0.044	1.077	0.212	1.940	-0.281	-0.112	-1.324	-1.226	-1.146	-1.385	-0.314
C	0.222	-0.151	1.734	-0.635	1.445	0.932	1.387	-1.248	-0.834	0.306	-1.900	-0.081
C	-0.681	-1.187	1.035	0.007	0.303	1.745	1.740	-0.737	0.574	1.130	-1.157	0.987
C	-1.928	-0.552	0.425	-0.783	-1.000	1.643	2.222	0.714	0.621	2.267	-0.326	0.395
C	-1.639	0.340	-0.743	-0.776	-1.603	0.274	1.162	1.755	0.457	1.814	0.859	-0.395
C	-2.575	1.171	-1.338	-1.619	-2.626	-0.145	1.411	3.120	0.336	2.599	1.587	-1.274
N	-1.841	1.748	-2.506	-1.138	-2.950	-1.504	0.053	3.724	0.302	1.717	2.692	-1.710
C	-0.926	0.738	-3.003	0.298	-2.745	-1.553	-0.869	2.909	1.068	0.836	3.051	-0.616
C	0.334	1.179	-3.730	0.929	-2.385	-2.888	-2.324	2.879	0.618	-0.525	3.644	-0.938
C	1.362	1.520	-2.635	0.664	-0.882	-3.086	-2.376	1.895	-0.564	-1.414	2.465	-1.345
O	-3.779	1.446	-1.156	-2.553	-3.293	0.348	2.406	3.856	0.279	3.777	1.545	-1.695
C	-1.924	3.113	-2.990	-2.009	-3.404	-2.563	-0.237	4.940	-0.415	1.711	3.182	-3.068
C	-2.629	4.085	-2.051	-2.278	-4.918	-2.460	-0.271	6.147	0.518	2.824	4.220	-3.290
O	-4.033	3.968	-2.061	-3.185	-5.249	-1.440	-1.373	6.004	1.394	4.107	3.652	-3.382
C	2.414	0.697	2.226	-0.396	3.332	-0.536	0.007	-2.015	-2.596	-1.736	-2.535	-1.154
C	1.997	-0.090	3.459	-0.579	3.919	0.856	1.068	-3.105	-2.413	-1.214	-3.808	-0.497
C	0.477	-0.286	3.290	-1.119	2.745	1.694	1.988	-2.635	-1.252	0.222	-3.475	-0.032
C	-0.093	-1.521	4.004	-0.827	2.842	3.199	2.211	-3.645	-0.080	0.632	-4.290	1.220
C	0.525	-1.707	5.396	-1.019	4.270	3.722	1.742	-5.081	-0.339	2.155	-4.399	1.383
C	-1.625	-1.421	4.137	-1.729	1.879	3.997	3.694	-3.653	0.354	-0.085	-3.901	2.522
C	-2.322	-2.769	4.305	-1.168	1.492	5.363	4.643	-4.351	-0.619	0.226	-4.824	3.698
O	1.209	2.889	-2.238	-0.616	-0.680	-3.692	-2.057	2.567	-1.788	-1.167	2.262	-2.737
C	2.792	1.376	-3.125	1.672	-0.237	-4.019	-3.757	1.298	-0.767	-2.895	2.728	-1.112
C	2.310	-1.274	0.690	1.706	2.107	0.039	-0.934	-2.207	-0.278	-1.968	-1.222	0.970
H	1.487	-0.403	-1.730	1.766	-0.168	-1.407	-1.788	0.133	0.476	-1.395	1.321	0.462
H	1.059	1.918	0.221	-1.066	0.954	-1.689	-0.119	0.669	-2.027	-0.563	-0.227	-2.032
H	2.620	1.324	-0.311	0.374	1.660	-2.393	-1.672	-0.143	-2.068	-2.236	-0.026	-1.523
H	-0.279	0.816	1.612	-1.541	1.024	0.485	1.818	-0.527	-1.542	0.808	-1.695	-1.033
H	-0.990	-1.981	1.715	0.116	0.562	2.796	2.539	-1.345	0.999	1.568	-1.852	1.698
H	-0.139	-1.695	0.235	1.023	0.104	1.396	0.893	-0.854	1.258	0.492	-0.499	1.584
H	-2.486	0.008	1.185	-1.819	-0.847	1.968	3.011	0.867	-0.128	2.911	-0.959	-0.227

H	-2.608	-1.345	0.083	-0.359	-1.735	2.341	2.710	0.886	1.591	2.905	0.036	1.214
H	-1.483	-0.049	-3.524	0.819	-3.542	-1.014	-0.741	3.088	2.142	1.388	3.602	0.151
H	0.200	2.007	-4.429	0.581	-2.974	-3.737	-2.735	3.855	0.360	-0.519	4.428	-1.697
H	0.686	0.321	-4.307	2.003	-2.560	-2.791	-2.912	2.485	1.450	-0.906	4.087	-0.016
H	-2.435	3.091	-3.960	-1.541	-3.186	-3.521	-1.201	4.842	-0.919	0.743	3.631	-3.273
H	-0.903	3.461	-3.171	-2.959	-2.865	-2.498	0.546	5.089	-1.161	1.857	2.339	-3.747
H	-2.387	5.093	-2.391	-1.320	-5.437	-2.334	-0.370	7.046	-0.102	2.774	4.970	-2.488
H	-2.225	3.973	-1.035	-2.716	-5.235	-3.409	0.672	6.203	1.072	2.616	4.724	-4.236
H	-4.222	3.076	-1.718	-3.006	-4.669	-0.673	-1.310	6.672	2.079	4.158	2.913	-2.741
H	2.105	1.744	2.329	-1.373	3.217	-1.021	0.339	-1.276	-3.334	-1.354	-2.459	-2.179
H	3.493	0.688	2.042	0.229	3.944	-1.192	-0.953	-2.410	-2.941	-2.827	-2.488	-1.206
H	2.245	0.419	4.393	-1.251	4.780	0.871	1.638	-3.273	-3.330	-1.229	-4.666	-1.175
H	2.511	-1.056	3.479	0.385	4.261	1.247	0.585	-4.053	-2.179	-1.845	-4.075	0.357
H	-0.008	0.579	3.760	-2.213	2.768	1.602	2.977	-2.432	-1.670	0.899	-3.829	-0.817
H	0.155	-2.413	3.412	0.224	2.567	3.370	1.636	-3.292	0.782	0.291	-5.310	0.988
H	1.592	-1.931	5.347	-0.297	4.967	3.292	0.654	-5.155	-0.400	2.626	-4.626	0.422
H	0.041	-2.528	5.931	-0.904	4.306	4.808	2.064	-5.726	0.485	2.418	-5.204	2.074
H	0.399	-0.797	5.994	-2.024	4.633	3.481	2.155	-5.493	-1.264	2.612	-3.483	1.764
H	-2.048	-0.909	3.269	-1.916	0.969	3.420	3.770	-4.137	1.335	-1.165	-3.926	2.347
H	-1.850	-0.777	4.996	-2.709	2.357	4.119	4.032	-2.621	0.493	0.151	-2.870	2.800
H	-2.163	-3.398	3.423	-0.976	2.370	5.986	4.443	-5.424	-0.677	0.047	-5.872	3.433
H	-3.401	-2.644	4.436	-0.222	0.951	5.253	5.681	-4.226	-0.300	-0.405	-4.585	4.557
H	-1.944	-3.315	5.173	-1.861	0.846	5.907	4.559	-3.943	-1.632	1.268	-4.738	4.019
H	0.607	2.947	-1.492	-1.305	-0.793	-3.033	-1.106	2.681	-1.851	-1.710	1.535	-3.051
H	2.979	0.359	-3.476	2.686	-0.361	-3.635	-4.474	2.085	-1.007	-3.092	2.927	-0.056
H	3.501	1.609	-2.327	1.468	0.830	-4.133	-4.091	0.788	0.140	-3.498	1.866	-1.413
H	2.972	2.068	-3.953	1.615	-0.702	-5.006	-3.748	0.578	-1.590	-3.217	3.592	-1.699
H	3.348	-1.080	0.402	2.206	2.621	-0.788	-1.981	-2.226	-0.597	-2.978	-0.881	0.721
H	1.829	-1.777	-0.151	2.219	1.155	0.187	-0.908	-1.853	0.755	-1.538	-0.495	1.661
H	2.317	-1.983	1.522	1.861	2.696	0.946	-0.571	-3.237	-0.275	-2.060	-2.163	1.513

Table S 99. Optimized atomic coordinates for the geometry-optimized conformers of 1b (1b-05 to 08).

	1b-05			1b-06			1b-07			1b-08		
C	1.534	0.776	0.066	-1.186	1.733	0.602	-0.346	-1.548	1.437	-0.665	1.514	0.011
C	0.945	0.441	1.399	-1.069	0.890	1.828	0.947	-0.880	1.774	-1.521	0.538	-0.691
C	-0.578	0.466	1.576	0.236	0.112	2.069	1.075	0.640	1.588	-0.929	-0.349	-1.776
C	-1.440	-0.656	0.974	0.821	-0.990	1.138	0.958	1.393	0.234	0.053	-1.462	-1.335
C	-1.591	-0.532	-0.573	0.582	-0.815	-0.393	-0.019	0.772	-0.810	1.555	-1.085	-1.440
C	-0.418	-1.121	-1.382	1.367	0.259	-1.147	-1.522	0.941	-0.589	2.133	-0.054	-0.466
C	0.346	-0.053	-2.159	1.058	1.704	-0.697	-2.061	0.247	0.681	1.828	1.414	-0.818
C	1.074	0.924	-1.288	-0.414	1.919	-0.570	-1.539	-1.149	0.782	0.638	2.030	-0.149
C	1.620	2.117	-1.758	-1.306	2.082	-1.628	-1.974	-2.223	0.009	0.671	3.337	0.336
N	2.386	2.649	-0.591	-2.627	1.706	-1.013	-0.797	-3.172	0.079	-0.767	3.636	0.774
C	2.834	1.540	0.223	-2.636	2.129	0.376	-0.148	-3.050	1.368	-1.540	2.429	0.818
C	3.010	1.758	1.722	-3.422	1.324	1.414	1.368	-3.250	1.473	-2.913	2.409	0.064
C	1.652	1.453	2.373	-2.475	0.208	1.919	2.009	-1.867	1.207	-2.689	1.485	-1.154
O	1.638	2.735	-2.827	-1.265	2.405	-2.833	-2.955	-2.531	-0.686	1.505	4.221	0.492
C	2.727	4.041	-0.473	-3.376	0.619	-1.601	-0.189	-3.590	-1.161	-1.041	4.790	1.563
C	1.874	4.842	0.531	-4.250	1.118	-2.767	-0.380	-5.083	-1.395	-2.458	5.337	1.420

O	0.498	4.582	0.400	-3.525	1.391	-3.938	0.368	-5.793	-0.423	-2.505	6.489	2.240
C	-2.895	-0.472	1.442	0.205	-2.393	1.430	2.314	1.434	-0.537	0.004	-2.601	-2.369
C	-3.710	-1.295	0.455	-0.121	-3.041	0.072	2.020	1.059	-1.999	1.270	-3.429	-2.125
C	-3.066	-0.993	-0.913	0.645	-2.240	-0.995	0.503	1.228	-2.196	2.319	-2.452	-1.526
C	-3.245	-2.101	-1.963	2.072	-2.733	-1.392	-0.017	2.616	-2.686	3.041	-2.910	-0.216
C	-4.662	-2.687	-1.929	2.444	-4.108	-0.835	1.079	3.569	-3.167	2.818	-4.370	0.191
C	-2.945	-1.572	-3.378	2.220	-2.692	-2.922	-1.088	2.409	-3.769	4.555	-2.619	-0.297
C	-2.552	-2.657	-4.376	3.640	-2.917	-3.435	-1.861	3.668	-4.154	5.337	-3.537	-1.237
O	0.927	2.691	2.410	-2.589	-0.942	1.080	2.109	-1.645	-0.200	-2.285	2.249	-2.290
C	1.795	0.916	3.790	-2.821	-0.265	3.320	3.377	-1.713	1.856	-3.952	0.748	-1.562
C	-0.943	-2.035	1.433	2.320	-1.033	1.486	0.575	2.835	0.619	-0.307	-2.012	0.052
H	1.317	-0.549	1.706	-1.104	1.595	2.677	1.070	-1.012	2.864	-2.004	-0.109	0.062
H	-0.958	1.422	1.197	0.998	0.893	2.171	0.321	1.059	2.264	-0.450	0.268	-2.545
H	-0.774	0.458	2.657	0.163	-0.333	3.068	2.034	0.937	2.031	-1.774	-0.843	-2.270
H	-1.579	0.547	-0.768	-0.470	-0.527	-0.515	0.187	-0.301	-0.793	1.658	-0.642	-2.440
H	-0.750	-1.890	-2.078	1.105	0.174	-2.206	-2.034	0.497	-1.451	3.220	-0.163	-0.478
H	0.291	-1.623	-0.722	2.445	0.091	-1.077	-1.813	1.995	-0.566	1.822	-0.254	0.563
H	-0.333	0.494	-2.827	1.477	2.398	-1.432	-3.154	0.240	0.642	1.720	1.502	-1.909
H	1.077	-0.543	-2.818	1.546	1.918	0.257	-1.780	0.815	1.573	2.692	2.031	-0.559
H	3.711	1.062	-0.239	-2.827	3.206	0.425	-0.710	-3.626	2.114	-1.712	2.141	1.881
H	3.370	2.750	1.999	-4.368	0.915	1.059	1.756	-4.021	0.807	-3.231	3.393	-0.278
H	3.750	1.037	2.078	-3.656	2.005	2.236	1.587	-3.565	2.496	-3.684	2.008	0.726
H	2.580	4.450	-1.475	-4.013	0.167	-0.843	0.872	-3.336	-1.147	-0.303	5.554	1.313
H	3.782	4.134	-0.200	-2.676	-0.133	-1.985	-0.672	-3.043	-1.972	-0.889	4.512	2.623
H	2.044	5.900	0.325	-4.818	1.993	-2.427	-0.028	-5.309	-2.408	-3.192	4.595	1.749
H	2.201	4.635	1.553	-4.961	0.321	-2.996	-1.445	-5.330	-1.330	-2.652	5.589	0.371
H	0.342	3.781	0.921	-2.717	1.881	-3.688	0.153	-6.725	-0.492	-3.382	6.872	2.178
H	-3.171	0.587	1.361	-0.695	-2.310	2.040	3.036	0.741	-0.103	0.019	-2.160	-3.373
H	-3.037	-0.770	2.485	0.921	-2.996	1.996	2.746	2.437	-0.465	-0.915	-3.190	-2.287
H	-4.775	-1.051	0.473	-1.192	-2.940	-0.131	2.255	0.001	-2.142	1.634	-3.895	-3.044
H	-3.625	-2.359	0.696	0.091	-4.110	0.055	2.624	1.622	-2.711	1.044	-4.245	-1.439
H	-3.582	-0.113	-1.319	0.054	-2.241	-1.917	0.170	0.495	-2.941	3.107	-2.314	-2.271
H	-2.545	-2.916	-1.733	2.808	-2.026	-0.997	-0.520	3.113	-1.853	2.658	-2.303	0.610
H	-4.872	-3.211	-0.995	2.356	-4.140	0.254	1.832	3.741	-2.392	1.786	-4.558	0.502
H	-4.808	-3.401	-2.745	3.479	-4.360	-1.083	0.659	4.543	-3.432	3.460	-4.616	1.041
H	-5.406	-1.892	-2.045	1.803	-4.893	-1.248	1.591	3.175	-4.052	3.053	-5.068	-0.616
H	-2.151	-0.821	-3.341	1.870	-1.716	-3.277	-1.800	1.657	-3.412	4.982	-2.696	0.710
H	-3.836	-1.040	-3.737	1.543	-3.437	-3.360	-0.611	1.979	-4.660	4.705	-1.581	-0.612
H	-1.624	-3.150	-4.066	3.691	-2.772	-4.517	-2.308	4.135	-3.270	5.345	-4.568	-0.877
H	-2.391	-2.240	-5.374	3.998	-3.929	-3.223	-2.668	3.433	-4.852	6.375	-3.208	-1.320
H	-3.323	-3.428	-4.460	4.337	-2.212	-2.970	-1.220	4.413	-4.634	4.910	-3.541	-2.246
H	0.145	2.574	2.955	-2.004	-0.851	0.325	2.904	-2.079	-0.519	-1.432	2.650	-2.092
H	2.361	-0.018	3.792	-2.790	0.564	4.029	3.794	-0.726	1.640	-4.753	1.460	-1.778
H	0.816	0.717	4.237	-2.115	-1.034	3.646	4.070	-2.469	1.473	-4.284	0.088	-0.758
H	2.316	1.644	4.416	-3.826	-0.695	3.332	3.312	-1.835	2.939	-3.775	0.150	-2.459
H	-1.036	-2.118	2.521	2.439	-1.124	2.570	-0.434	2.891	1.040	-1.350	-2.348	0.071
H	0.103	-2.217	1.182	2.840	-0.122	1.172	0.627	3.517	-0.231	-0.182	-1.260	0.834
H	-1.524	-2.843	0.982	2.825	-1.888	1.034	1.272	3.208	1.377	0.317	-2.866	0.317

Table S 1010. Optimized atomic coordinates for the geometry-optimized conformers of 1b (1b-09 to 10).

	1b-09			1b-10		
C	0.016	2.184	-0.452	-0.718	-2.124	-0.280
C	0.445	2.035	0.963	-1.078	-1.402	-1.534
C	1.347	0.853	1.356	-0.132	-0.315	-2.070
C	0.966	-0.648	1.235	0.243	0.995	-1.322
C	0.035	-1.019	0.044	0.303	0.886	0.231
C	0.616	-1.058	-1.368	1.497	0.182	0.875
C	1.095	0.313	-1.894	1.604	-1.325	0.547
C	0.090	1.385	-1.625	0.280	-1.994	0.716
C	-1.083	1.589	-2.342	-0.293	-2.349	1.937
N	-1.905	2.489	-1.431	-1.774	-2.427	1.587
C	-1.041	3.254	-0.562	-1.915	-2.905	0.228
C	-1.477	3.517	0.892	-3.086	-2.437	-0.643
C	-0.858	2.379	1.742	-2.607	-1.157	-1.368
O	-1.575	1.231	-3.415	0.056	-2.547	3.109
C	-3.334	2.430	-1.444	-2.698	-1.651	2.367
C	-3.983	1.494	-0.393	-2.260	-0.195	2.616
O	-3.361	0.238	-0.315	-3.137	0.428	3.533
C	0.163	-1.151	2.474	-0.806	2.125	-1.552
C	-1.039	-1.958	1.951	-1.130	2.736	-0.179
C	-0.732	-2.292	0.480	0.003	2.305	0.768
C	-0.006	-3.643	0.187	1.240	3.248	0.898
C	0.160	-4.568	1.394	1.060	4.623	0.253
C	-0.679	-4.382	-0.987	1.635	3.375	2.378
C	-2.060	-4.963	-0.684	2.975	4.064	2.627
O	-1.713	1.229	1.711	-2.783	-0.030	-0.512
C	-0.607	2.785	3.186	-3.324	-0.919	-2.690
C	2.311	-1.396	1.183	1.584	1.436	-1.938
H	1.078	2.916	1.179	-1.056	-2.167	-2.331
H	2.261	1.008	0.770	0.800	-0.851	-2.283
H	1.657	1.017	2.395	-0.508	-0.018	-3.058
H	-0.729	-0.241	0.029	-0.580	0.309	0.503
H	-0.175	-1.415	-2.037	1.386	0.275	1.961
H	1.440	-1.774	-1.452	2.445	0.667	0.619
H	1.279	0.231	-2.970	2.344	-1.779	1.211
H	2.049	0.579	-1.430	1.965	-1.465	-0.475
H	-0.726	4.184	-1.066	-1.789	-3.996	0.217
H	-2.557	3.584	1.032	-4.015	-2.267	-0.098
H	-1.054	4.476	1.201	-3.286	-3.225	-1.373
H	-3.592	2.070	-2.443	-2.755	-2.156	3.341
H	-3.737	3.438	-1.305	-3.687	-1.679	1.911
H	-5.019	1.347	-0.703	-2.325	0.358	1.681
H	-3.989	1.985	0.583	-1.225	-0.169	2.972
H	-2.609	0.355	0.284	-2.943	0.109	4.417
H	-0.176	-0.317	3.090	-1.711	1.734	-2.018
H	0.811	-1.771	3.103	-0.390	2.876	-2.232
H	-1.930	-1.326	1.975	-2.059	2.293	0.190
H	-1.256	-2.834	2.563	-1.285	3.815	-0.221
H	-1.675	-2.311	-0.074	-0.413	2.204	1.778
H	1.004	-3.414	-0.160	2.091	2.776	0.395
H	0.740	-4.090	2.189	0.799	4.540	-0.805
H	0.690	-5.479	1.097	1.983	5.206	0.313

H	-0.799	-4.870	1.823	0.271	5.198	0.750
H	-0.018	-5.194	-1.313	1.671	2.371	2.814
H	-0.756	-3.690	-1.833	0.837	3.910	2.910
H	-2.006	-5.762	0.060	3.775	3.569	2.067
H	-2.508	-5.384	-1.589	3.239	4.031	3.687
H	-2.745	-4.200	-0.302	2.959	5.115	2.326
H	-2.354	1.306	2.423	-3.724	0.149	-0.438
H	0.080	3.632	3.234	-2.957	-0.004	-3.161
H	-0.176	1.954	3.748	-4.401	-0.813	-2.528
H	-1.542	3.083	3.671	-3.166	-1.755	-3.376
H	2.936	-1.087	2.028	2.394	0.743	-1.690
H	2.864	-1.175	0.265	1.877	2.435	-1.611
H	2.184	-2.477	1.252	1.495	1.465	-3.029

Table S 1111. Geometry-optimized conformers of diastereomer 1c at wB97XD/6-311++G**/PCM(MeOH) level.

1c-01	1c-02	1c-03	1c-04
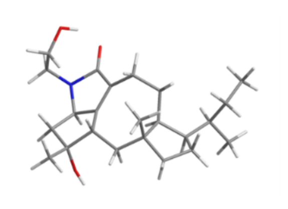	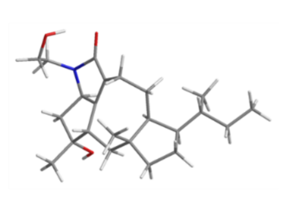	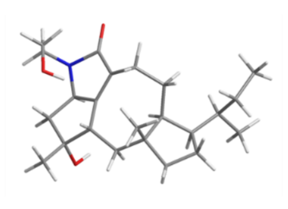	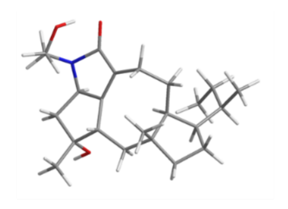
1c-05	1c-06	1c-07	1c-08
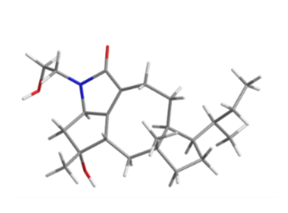	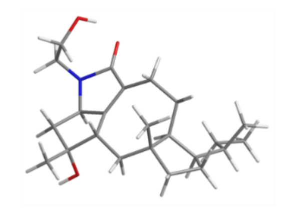	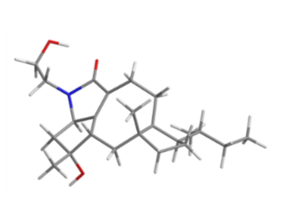	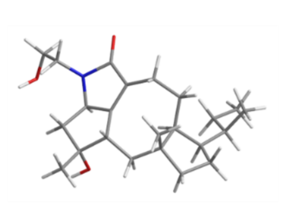
1c-09	1c-10	1c-11	1c-12
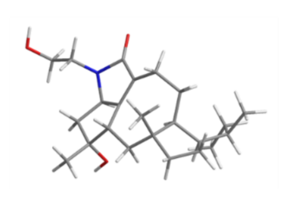	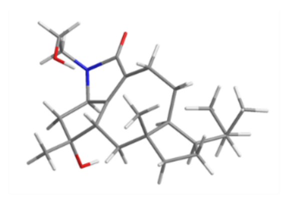	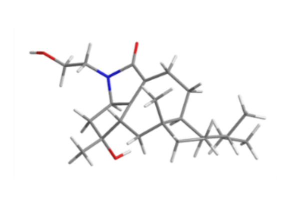	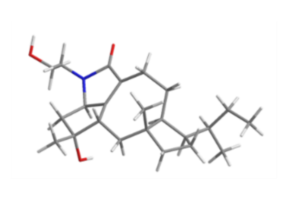

The conformational search provided twelve conformers for 1c, of which two (conf. 1c-02: 6.05% and 1c-07: 91.35%) significantly contributed to the final spectra (

Table S 66). The energies of the conformers and their Boltzmann weights used for calculating UV-Vis and ECD spectra are listed below (Table S 77).

Table S 1212. Gibbs free energy and Boltzmann population of twelve geometry-optimized conformers of 1c.

Conformer	Energy (Hartree)	Boltzmann population (%)	No. of imaginary frequencies
1c-01	-1178.48902400	0.89500	0
1c-02	-1178.49082800	6.05000	0
1c-03	-1178.48902500	0.89600	0
1c-04	-1178.48598600	0.03580	1
1c-05	-1178.48836000	0.44300	0
1c-06	-1178.48751300	0.18100	0
1c-07	-1178.49339100	91.350000	0
1c-08	-1178.48614800	0.042600	0
1c-09	-1178.48375700	0.003830	0
1c-10	-1178.48698700	0.103000	0
1c-11	-1178.48363200	0.002960	0
1c-12	-1178.48154100	0.000324	0

Table S 1313. Atomic coordinates for the geometry-optimized conformers of 1c (1c-01 to 04).

	1c-01			1c-02			1c-03			1c-04		
N	-3.206	-1.217	-1.969	-3.181	-1.215	-2.036	-3.062	-1.295	-2.194	-2.901	-0.925	-2.296
C	-3.001	-1.307	-0.538	-2.876	-1.395	-0.636	-2.885	-1.417	-0.765	-2.634	-1.314	-0.931
C	-1.854	-0.310	-0.474	-1.807	-0.309	-0.562	-1.807	-0.346	-0.641	-1.605	-0.222	-0.649
C	-1.167	-0.218	-1.710	-1.187	-0.120	-1.829	-1.074	-0.225	-1.853	-0.948	0.175	-1.849
C	-1.887	-0.926	-2.663	-1.924	-0.820	-2.776	-1.719	-0.986	-2.832	-1.635	-0.386	-2.919
C	-1.975	0.576	0.729	-1.975	0.506	0.693	-2.013	0.479	0.601	-1.847	0.384	0.709
C	-0.757	0.610	1.675	-0.729	0.743	1.574	-0.821	0.594	1.575	-0.645	0.535	1.667
C	0.595	1.109	1.141	0.512	1.366	0.924	0.506	1.162	1.053	0.594	1.279	1.157
C	1.222	0.062	0.138	1.205	0.310	-0.028	1.212	0.116	0.102	1.346	0.378	0.094
C	1.313	0.483	-1.331	1.269	0.668	-1.514	1.378	0.515	-1.366	1.475	0.951	-1.318
C	-0.028	0.684	-2.065	-0.085	0.810	-2.235	0.077	0.677	-2.178	0.150	1.171	-2.072
O	-1.786	-1.188	-3.875	-1.869	-1.027	-4.003	-1.514	-1.351	-3.994	-1.534	-0.399	-4.160
C	-4.059	-0.690	0.373	-3.917	-0.956	0.386	-3.997	-0.928	0.153	-3.722	-1.071	0.107
C	-3.243	0.007	1.469	-3.075	-0.276	1.478	-3.247	-0.212	1.295	-2.939	-0.547	1.322
C	-4.369	-0.672	-2.619	-4.411	-0.700	-2.584	-4.211	-0.763	-2.868	-4.131	-0.373	-2.804
C	-4.208	0.797	-3.088	-4.349	0.790	-2.995	-4.093	0.738	-3.224	-4.113	1.164	-2.981
O	-3.622	0.924	-4.362	-3.844	1.000	-4.294	-4.011	1.575	-2.095	-3.569	1.587	-4.211
C	1.602	1.146	2.323	1.580	1.635	2.020	1.486	1.308	2.251	1.617	1.432	2.322
C	2.948	0.800	1.704	2.916	1.336	1.355	2.857	0.964	1.687	2.980	1.200	1.683
C	2.573	-0.374	0.798	2.601	0.033	0.619	2.533	-0.268	0.841	2.681	-0.006	0.795

C	0.479	2.540	0.600	0.166	2.723	0.293	0.306	2.563	0.458	0.219	2.697	0.703
H	-2.729	-2.334	-0.257	-2.493	-2.409	-0.460	-2.536	-2.435	-0.534	-2.223	-2.331	-0.901
O	-2.898	-1.043	2.375	-2.409	-1.291	2.236	-2.846	-1.174	2.273	-2.259	-1.648	1.932
C	-4.027	1.089	2.198	-3.903	0.609	2.398	-4.125	0.778	2.038	-3.826	0.154	2.340
H	-2.197	1.609	0.425	-2.396	1.491	0.441	-2.317	1.506	0.350	-2.303	1.379	0.590
C	3.691	-0.938	-0.097	3.696	-0.545	-0.290	3.691	-0.879	0.030	3.875	-0.586	0.013
C	4.591	-1.873	0.721	3.315	-1.962	-0.734	4.544	-1.773	0.938	4.669	0.414	-0.836
C	4.536	0.139	-0.799	5.063	-0.536	0.410	4.574	0.160	-0.681	3.520	-1.842	-0.801
C	5.276	-0.377	-2.031	6.200	-1.128	-0.421	5.387	-0.422	-1.836	2.846	-2.953	0.002
H	0.577	-0.823	0.144	0.625	-0.616	0.032	0.590	-0.786	0.085	0.769	-0.546	-0.022
H	2.299	-1.191	1.479	2.432	-0.719	1.404	2.234	-1.043	1.560	2.401	-0.802	1.498
H	-0.592	-0.401	2.057	-0.434	-0.211	2.014	-0.605	-0.393	1.993	-0.331	-0.462	1.984
H	-1.035	1.240	2.532	-1.049	1.386	2.404	-1.162	1.213	2.415	-1.020	1.046	2.563
H	1.865	-0.294	-1.866	1.818	-0.125	-2.027	1.973	-0.262	-1.855	2.061	0.247	-1.909
H	1.892	1.403	-1.445	1.847	1.585	-1.673	1.948	1.443	-1.463	2.037	1.891	-1.317
H	-0.379	1.710	-1.914	-0.459	1.832	-2.132	-0.279	1.708	-2.093	-0.250	2.163	-1.847
H	0.150	0.592	-3.140	0.080	0.670	-3.306	0.313	0.539	-3.237	0.362	1.183	-3.144
H	-4.757	-1.411	0.802	-4.520	-1.774	0.786	-4.638	-1.720	0.543	-4.309	-1.959	0.352
H	-4.628	0.072	-0.162	-4.592	-0.211	-0.039	-4.615	-0.192	-0.364	-4.408	-0.288	-0.222
H	-4.534	-1.283	-3.514	-4.610	-1.283	-3.490	-4.276	-1.302	-3.820	-4.278	-0.816	-3.796
H	-5.234	-0.783	-1.968	-5.223	-0.881	-1.883	-5.110	-0.962	-2.287	-4.958	-0.685	-2.171
H	-3.642	1.351	-2.328	-3.768	1.342	-2.245	-5.005	1.005	-3.759	-3.580	1.612	-2.132
H	-5.208	1.228	-3.154	-5.371	1.173	-2.987	-3.241	0.888	-3.893	-5.148	1.507	-2.957
H	-2.866	0.319	-4.417	-3.062	0.438	-4.416	-3.124	1.497	-1.731	-2.765	1.074	-4.387
H	1.584	2.112	2.835	1.502	2.648	2.422	1.428	2.304	2.698	1.520	2.402	2.817
H	1.342	0.380	3.063	1.441	0.941	2.858	1.232	0.584	3.035	1.443	0.661	3.081
H	3.708	0.542	2.447	3.730	1.241	2.076	3.605	0.771	2.461	3.774	1.007	2.410
H	3.330	1.643	1.117	3.190	2.126	0.643	3.231	1.781	1.059	3.277	2.069	1.084
H	1.453	2.926	0.284	-0.170	3.403	1.082	-0.111	3.221	1.227	-0.164	3.254	1.563
H	-0.205	2.633	-0.243	1.035	3.179	-0.189	1.255	2.997	0.130	1.086	3.241	0.317
H	0.105	3.195	1.393	-0.637	2.669	-0.440	-0.381	2.583	-0.387	-0.559	2.720	-0.058
H	-2.439	-0.669	3.130	-3.078	-1.813	2.686	-2.286	-1.841	1.866	-2.921	-2.262	2.260
H	-3.414	1.552	2.977	-3.268	1.068	3.159	-3.557	1.267	2.834	-4.330	1.013	1.890
H	-4.342	1.873	1.506	-4.399	1.401	1.833	-4.498	1.544	1.356	-4.592	-0.530	2.717
H	-4.914	0.659	2.669	-4.672	0.018	2.905	-4.977	0.263	2.488	-3.232	0.502	3.188
H	3.215	-1.551	-0.874	3.792	0.090	-1.180	3.250	-1.528	-0.738	4.566	-0.925	0.799
H	5.401	-2.286	0.114	3.979	-2.338	-1.516	3.949	-2.589	1.358	5.589	-0.057	-1.197
H	4.019	-2.712	1.128	2.294	-2.009	-1.118	4.958	-1.195	1.771	4.957	1.293	-0.254
H	5.045	-1.336	1.560	3.377	-2.650	0.118	5.382	-2.219	0.395	4.113	0.759	-1.709
H	3.906	0.979	-1.099	5.322	0.496	0.667	3.960	0.977	-1.066	2.881	-1.575	-1.650
H	5.257	0.546	-0.079	4.980	-1.079	1.361	5.249	0.612	0.056	4.448	-2.230	-1.237
H	5.947	-1.205	-1.786	6.074	-2.201	-0.582	4.725	-0.832	-2.605	3.435	-3.208	0.889
H	4.567	-0.736	-2.784	6.256	-0.647	-1.403	6.010	0.345	-2.305	1.846	-2.664	0.336
H	5.879	0.411	-2.489	7.162	-0.981	0.078	6.049	-1.226	-1.504	2.742	-3.859	-0.602

Table S 1414. Atomic coordinates for the geometry-optimized conformers of 1c (1c-05 to 08).

	1c-05			1c-06			1c-07			1c-08		
N	-3.009	-0.236	-2.369	-2.980	-1.029	-2.199	-3.150	-0.537	-2.124	-2.823	0.025	-2.425

C	-2.886	-0.681	-0.999	-2.748	-1.340	-0.807	-2.820	-1.008	-0.798	-2.646	-0.674	-1.176
C	-1.731	0.224	-0.637	-1.706	-0.252	-0.567	-1.719	0.025	-0.542	-1.592	0.264	-0.615
C	-0.965	0.597	-1.781	-1.026	0.078	-1.772	-1.102	0.400	-1.770	-0.854	0.897	-1.667
C	-1.689	0.236	-2.915	-1.696	-0.533	-2.825	-1.883	-0.112	-2.802	-1.549	0.683	-2.856
C	-1.884	0.779	0.743	-1.955	0.431	0.751	-1.873	0.606	0.841	-1.859	0.558	0.833
C	-0.737	0.471	1.732	-0.761	0.590	1.718	-0.621	0.731	1.738	-0.700	0.349	1.836
C	0.678	0.972	1.406	0.499	1.301	1.211	0.625	1.401	1.150	0.626	1.070	1.568
C	1.287	0.169	0.190	1.271	0.369	0.189	1.282	0.433	0.086	1.357	0.402	0.331
C	1.520	0.948	-1.107	1.392	0.877	-1.248	1.401	0.962	-1.344	1.591	1.289	-0.892
C	0.256	1.456	-1.830	0.071	1.068	-2.017	0.075	1.284	-2.056	0.326	1.814	-1.598
O	-1.534	0.265	-4.139	-1.574	-0.607	-4.062	-1.769	-0.263	-4.038	-1.387	0.932	-4.056
C	-3.994	-0.244	-0.037	-3.852	-1.016	0.192	-3.823	-0.771	0.321	-3.790	-0.579	-0.171
C	-3.240	0.148	1.241	-3.084	-0.437	1.392	-2.937	-0.329	1.496	-3.069	-0.372	1.171
C	-4.224	-0.020	-3.091	-4.191	-0.487	-2.759	-4.388	0.083	-2.536	-4.057	0.296	-3.097
C	-4.801	-1.345	-3.623	-4.151	1.042	-2.998	-4.932	-0.540	-3.838	-4.528	-0.925	-3.908
O	-5.166	-2.236	-2.593	-3.579	1.409	-4.233	-4.268	-0.116	-4.999	-4.814	-2.044	-3.097
C	1.610	0.617	2.598	1.493	1.471	2.396	1.709	1.533	2.256	1.589	0.817	2.764
C	2.959	0.307	1.965	2.870	1.270	1.782	3.029	1.221	1.566	2.965	0.656	2.132
C	2.543	-0.557	0.774	2.624	0.049	0.899	2.646	0.010	0.716	2.638	-0.244	0.943
C	0.700	2.500	1.255	0.159	2.713	0.710	0.289	2.821	0.670	0.408	2.588	1.483
H	-2.683	-1.764	-0.946	-2.357	-2.362	-0.707	-2.450	-2.040	-0.843	-2.309	-1.711	-1.342
O	-3.030	-1.092	1.922	-2.446	-1.515	2.085	-2.238	-1.474	1.994	-2.532	-1.631	1.594
C	-4.028	1.099	2.131	-3.976	0.346	2.344	-3.723	0.337	2.615	-3.976	0.198	2.252
H	-2.006	1.872	0.709	-2.377	1.432	0.576	-2.328	1.606	0.765	-2.204	1.598	0.945
C	3.664	-0.994	-0.186	3.848	-0.483	0.127	3.714	-0.552	-0.233	3.835	-0.671	0.070
C	4.414	-2.196	0.402	4.548	0.512	-0.808	3.243	-1.885	-0.826	4.679	0.462	-0.528
C	4.652	0.128	-0.548	3.539	-1.823	-0.558	5.064	-0.713	0.482	3.417	-1.716	-0.977
C	5.430	-0.140	-1.835	4.769	-2.537	-1.114	6.173	-1.298	-0.390	4.589	-2.381	-1.697
H	0.570	-0.616	-0.075	0.717	-0.573	0.121	0.652	-0.459	0.022	0.720	-0.416	-0.022
H	2.150	-1.485	1.211	2.382	-0.762	1.602	2.419	-0.798	1.430	2.287	-1.186	1.390
H	-0.668	-0.614	1.850	-0.468	-0.402	2.067	-0.337	-0.269	2.071	-0.492	-0.721	1.900
H	-1.030	0.888	2.707	-1.139	1.132	2.595	-0.930	1.286	2.633	-1.075	0.656	2.822
H	2.049	0.288	-1.800	1.976	0.143	-1.806	1.908	0.200	-1.941	2.136	0.694	-1.627
H	2.178	1.807	-0.942	1.958	1.813	-1.292	2.042	1.849	-1.382	2.237	2.139	-0.649
H	-0.021	2.437	-1.429	-0.334	2.065	-1.824	-0.219	2.315	-1.846	0.004	2.750	-1.132
H	0.506	1.630	-2.880	0.293	1.049	-3.087	0.246	1.248	-3.135	0.592	2.081	-2.625
H	-4.741	-1.011	0.170	-4.463	-1.876	0.474	-4.427	-1.646	0.569	-4.443	-1.455	-0.151
H	-4.497	0.644	-0.427	-4.513	-0.240	-0.198	-4.496	0.053	0.079	-4.403	0.302	-0.375
H	-4.966	0.458	-2.444	-4.318	-0.968	-3.736	-5.137	-0.061	-1.760	-4.830	0.566	-2.373
H	-4.009	0.637	-3.936	-5.036	-0.764	-2.134	-4.225	1.157	-2.694	-3.900	1.133	-3.780
H	-4.044	-1.851	-4.224	-3.628	1.518	-2.158	-5.974	-0.225	-3.924	-3.736	-1.227	-4.593
H	-5.652	-1.100	-4.268	-5.182	1.398	-3.008	-4.914	-1.632	-3.735	-5.400	-0.618	-4.496
H	-5.955	-1.906	-2.158	-2.778	0.880	-4.373	-3.309	-0.233	-4.845	-5.632	-1.885	-2.622
H	1.641	1.422	3.337	1.364	2.436	2.894	1.683	2.516	2.735	1.532	1.620	3.503
H	1.241	-0.280	3.109	1.321	0.691	3.148	1.537	0.786	3.040	1.321	-0.115	3.275
H	3.646	-0.202	2.647	3.658	1.104	2.523	3.833	1.019	2.276	3.705	0.216	2.807
H	3.447	1.229	1.629	3.154	2.143	1.182	3.349	2.054	0.927	3.350	1.627	1.800
H	1.717	2.869	1.093	-0.238	3.298	1.544	-0.005	3.425	1.535	0.030	2.946	2.445
H	0.080	2.865	0.437	1.045	3.232	0.333	1.151	3.308	0.204	1.342	3.119	1.276

H	0.324	2.958	2.175	-0.597	2.726	-0.073	-0.541	2.856	-0.034	-0.320	2.880	0.729
H	-2.595	-0.921	2.760	-3.132	-2.086	2.440	-2.886	-2.086	2.351	-3.267	-2.232	1.734
H	-3.459	1.345	3.033	-4.451	1.185	1.830	-4.475	-0.351	3.014	-3.422	0.329	3.184
H	-4.246	2.032	1.605	-4.764	-0.298	2.746	-3.057	0.625	3.431	-4.382	1.166	1.948
H	-4.971	0.638	2.434	-3.392	0.734	3.182	-4.238	1.229	2.252	-4.814	-0.479	2.444
H	3.189	-1.337	-1.115	4.576	-0.711	0.920	3.872	0.162	-1.052	4.497	-1.197	0.774
H	5.222	-2.529	-0.255	5.567	0.179	-1.023	3.902	-2.233	-1.624	5.662	0.088	-0.825
H	3.740	-3.044	0.554	4.622	1.503	-0.354	2.234	-1.814	-1.240	4.845	1.261	0.199
H	4.856	-1.940	1.370	4.033	0.621	-1.765	3.224	-2.656	-0.046	4.218	0.907	-1.412
H	4.124	1.078	-0.653	3.047	-2.476	0.174	5.390	0.266	0.846	2.824	-2.487	-0.472
H	5.352	0.263	0.285	2.814	-1.675	-1.366	4.923	-1.342	1.371	2.752	-1.260	-1.718
H	6.005	-1.069	-1.779	5.226	-1.976	-1.934	5.976	-2.338	-0.662	5.141	-1.667	-2.314
H	4.747	-0.224	-2.687	5.528	-2.670	-0.336	6.283	-0.726	-1.317	5.292	-2.816	-0.979
H	6.132	0.670	-2.049	4.506	-3.526	-1.499	7.133	-1.271	0.134	4.240	-3.182	-2.353

Table S 1515. Atomic coordinates for the geometry-optimized conformers of 1c (1c-09 to 12).

	1c-09			1c-10			1c-11			1c-12		
N	-2.679	-0.104	-2.582	-2.763	-1.166	-2.369	-2.766	-0.087	-2.547	-3.088	-0.381	-2.257
C	-2.435	-0.900	-1.404	-2.594	-1.413	-0.957	-2.442	-0.976	-1.461	-2.882	-0.808	-0.893
C	-1.397	0.052	-0.805	-1.616	-0.266	-0.705	-1.393	-0.037	-0.834	-1.711	0.113	-0.608
C	-0.681	0.713	-1.857	-0.876	0.026	-1.886	-0.728	0.684	-1.884	-1.005	0.438	-1.810
C	-1.384	0.500	-3.044	-1.444	-0.687	-2.945	-1.503	0.549	-3.038	-1.803	0.054	-2.888
C	-1.742	0.334	0.637	-1.934	0.422	0.599	-1.776	0.228	0.607	-1.832	0.740	0.748
C	-0.613	0.350	1.692	-0.785	0.606	1.614	-0.679	0.393	1.681	-0.628	0.592	1.708
C	0.648	1.160	1.374	0.494	1.307	1.139	0.543	1.246	1.327	0.742	1.109	1.250
C	1.463	0.403	0.248	1.297	0.340	0.173	1.411	0.461	0.262	1.319	0.182	0.108
C	1.748	1.183	-1.034	1.510	0.830	-1.259	1.702	1.176	-1.057	1.511	0.819	-1.269
C	0.511	1.624	-1.839	0.234	1.010	-2.105	0.479	1.577	-1.900	0.225	1.268	-1.991
O	-1.228	0.723	-4.252	-1.191	-0.925	-4.130	-1.413	0.858	-4.232	-1.714	0.041	-4.119
C	-3.539	-0.989	-0.358	-3.758	-1.121	-0.021	-3.481	-1.214	-0.374	-3.940	-0.378	0.123
C	-2.798	-0.763	0.968	-3.090	-0.460	1.201	-2.707	-0.983	0.934	-3.120	0.066	1.349
C	-3.901	0.591	-2.905	-3.949	-0.684	-3.017	-4.002	0.642	-2.711	-4.344	-0.188	-2.912
C	-5.003	-0.384	-3.340	-3.967	0.846	-3.236	-5.121	-0.271	-3.200	-4.912	-1.542	-3.394
O	-6.196	0.310	-3.634	-3.977	1.581	-2.035	-6.283	0.532	-3.279	-6.173	-1.369	-4.000
C	1.598	1.154	2.611	1.445	1.513	2.353	1.473	1.369	2.573	1.755	0.943	2.419
C	2.994	0.928	2.043	2.840	1.241	1.808	2.886	1.164	2.043	3.070	0.566	1.751
C	2.711	-0.145	0.995	2.594	-0.011	0.966	2.669	0.016	1.061	2.600	-0.466	0.726
C	0.276	2.623	1.093	0.170	2.702	0.584	0.092	2.664	0.947	0.677	2.607	0.920
H	-2.020	-1.874	-1.691	-2.154	-2.412	-0.818	-2.003	-1.894	-1.869	-2.655	-1.890	-0.851
O	-2.073	-1.954	1.292	-2.589	-1.477	2.073	-1.940	-2.151	1.239	-2.808	-1.078	2.148
C	-3.730	-0.387	2.110	-4.067	0.346	2.036	-3.616	-0.755	2.126	-3.893	0.998	2.265
H	-2.263	1.304	0.699	-2.358	1.422	0.419	-2.406	1.133	0.638	-2.035	1.817	0.647
C	3.930	-0.701	0.236	3.864	-0.613	0.323	3.923	-0.534	0.355	3.645	-1.081	-0.226
C	4.852	0.342	-0.404	4.599	0.248	-0.712	4.788	0.491	-0.389	4.349	-2.262	0.453
C	3.565	-1.806	-0.769	3.703	-2.077	-0.130	3.580	-1.758	-0.508	4.665	-0.091	-0.834
C	2.770	-2.968	-0.177	2.828	-2.363	-1.350	4.799	-2.529	-1.012	5.898	0.223	0.017
H	0.869	-0.462	-0.066	0.716	-0.582	0.083	0.862	-0.444	-0.014	0.602	-0.632	-0.049
H	2.331	-1.000	1.571	2.278	-0.778	1.689	2.320	-0.826	1.678	2.222	-1.308	1.322

H	-0.309	-0.682	1.884	-0.493	-0.372	2.005	-0.320	-0.600	1.967	-0.505	-0.464	1.961
H	-1.054	0.728	2.623	-1.202	1.163	2.463	-1.164	0.815	2.569	-0.904	1.101	2.641
H	2.341	0.540	-1.686	2.119	0.087	-1.769	2.303	0.499	-1.667	2.002	0.082	-1.910
H	2.366	2.065	-0.833	2.083	1.762	-1.281	2.318	2.066	-0.896	2.186	1.678	-1.218
H	0.183	2.610	-1.499	-0.169	2.016	-1.968	0.160	2.587	-1.629	-0.022	2.292	-1.692
H	0.813	1.770	-2.880	0.514	0.953	-3.161	0.793	1.656	-2.945	0.431	1.322	-3.063
H	-4.069	-1.944	-0.366	-4.328	-2.003	0.274	-3.913	-2.215	-0.404	-4.657	-1.156	0.389
H	-4.276	-0.195	-0.486	-4.433	-0.394	-0.475	-4.295	-0.490	-0.430	-4.486	0.489	-0.257
H	-4.243	1.186	-2.050	-3.952	-1.140	-4.014	-4.290	1.120	-1.767	-5.067	0.264	-2.229
H	-3.667	1.276	-3.721	-4.831	-1.016	-2.474	-3.818	1.428	-3.444	-4.189	0.473	-3.766
H	-5.238	-1.079	-2.532	-4.893	1.076	-3.763	-5.265	-1.106	-2.506	-5.061	-2.201	-2.536
H	-4.653	-0.967	-4.198	-3.124	1.135	-3.871	-4.849	-0.679	-4.180	-4.196	-2.013	-4.074
H	-6.082	0.792	-4.456	-3.087	1.567	-1.670	-6.995	0.006	-3.650	-6.051	-0.971	-4.865
H	1.504	2.073	3.195	1.327	2.507	2.792	1.333	2.323	3.088	1.813	1.846	3.032
H	1.345	0.320	3.276	1.222	0.782	3.138	1.243	0.576	3.294	1.442	0.123	3.075
H	3.723	0.611	2.795	3.587	1.084	2.591	3.613	0.926	2.824	3.810	0.174	2.453
H	3.372	1.845	1.576	3.180	2.075	1.182	3.235	2.064	1.524	3.509	1.438	1.252
H	1.149	3.218	0.809	1.070	3.216	0.236	0.932	3.286	0.623	-0.031	2.849	0.130
H	-0.480	2.735	0.316	-0.549	2.690	-0.233	-0.667	2.678	0.165	0.360	3.155	1.813
H	-0.140	3.061	2.005	-0.269	3.306	1.384	-0.351	3.139	1.827	1.656	2.995	0.625
H	-2.710	-2.657	1.445	-1.952	-2.023	1.606	-1.247	-2.253	0.579	-2.317	-1.717	1.625
H	-3.168	-0.258	3.037	-3.556	0.798	2.890	-4.225	0.140	1.976	-3.276	1.289	3.119
H	-4.259	0.544	1.889	-4.515	1.142	1.437	-4.282	-1.611	2.260	-4.192	1.900	1.729
H	-4.476	-1.172	2.268	-4.864	-0.299	2.412	-3.029	-0.631	3.038	-4.790	0.499	2.641
H	4.530	-1.191	1.017	4.551	-0.675	1.178	4.547	-0.908	1.180	3.083	-1.505	-1.067
H	5.146	1.112	0.314	4.667	1.290	-0.388	5.784	0.081	-0.578	3.629	-3.045	0.707
H	4.393	0.842	-1.259	4.129	0.240	-1.695	4.921	1.402	0.200	4.847	-1.954	1.378
H	5.767	-0.142	-0.759	5.621	-0.125	-0.837	4.364	0.776	-1.354	5.108	-2.701	-0.203
H	3.008	-1.381	-1.611	4.707	-2.472	-0.327	2.953	-2.432	0.088	5.010	-0.509	-1.787
H	4.499	-2.194	-1.193	3.316	-2.651	0.720	2.968	-1.452	-1.364	4.161	0.845	-1.083
H	2.655	-3.772	-0.909	3.185	-1.838	-2.241	5.448	-2.820	-0.180	6.516	-0.665	0.173
H	3.276	-3.384	0.701	2.845	-3.433	-1.578	4.496	-3.440	-1.535	6.519	0.969	-0.487
H	1.767	-2.660	0.132	1.786	-2.079	-1.186	5.395	-1.932	-1.708	5.636	0.619	0.999

Comparison of stereoisomers to determine absolute configuration

NMR data indicated an α-orientation of H-4, suggesting a configuration of 1a (4*R**, 6*R**, 7*S**, 13*S**, 14*R**, 17*R**, 21*S**). To verify the assignment of C-4 as 4*R**, we also considered the configuration of 1b (4*S**, 6*R**, 7*S**, 13*S**, 14*R**, 17*R**, 21*S**), which is the 4S-epimer of 1a, during ECD analysis. Similarly, to verify the assignment of C-21 as 21*S*, we considered the configuration of 1c (4*R**, 6*R**, 7*S**, 13*S**, 14*R**, 17*R**, 21*R**), which is the 21*R*-epimer of 1a. Consequently, we examined six stereoisomers: three diastereomers (1a, 1b, and 1c), along with their enantiomers *ent*-1a (4*S*, 6*S*, 7*R*, 13*R*, 14*S*, 17*S*, 21*R*), *ent*-1b (4*R*, 6*S*, 7*R*, 13*R*, 14*S*, 17*S*, 21*R*), and *ent*-1c (4*S*, 6*S*, 7*R*, 13*R*, 14*S*, 17*S*, 21*S*) as noted in the manuscript. The geometry-optimized structures of these stereoisomers were analyzed by focusing on the calculated distances between key protons: H-4/H-7, H-4/H_3-19, and H-4/H-13. The distance H-4/H-13 was

less than 4 Å in the case of 1a/*ent*-1a, which supported the observed ROESY correlation of H-4/H-13.

Ultimately, the absolute configuration was assigned as 1a based on the comparison of the experimental ECD curve of **1** with the calculated ECD curves of 1a and *ent*-1a. Although the calculated ECD curve of *ent*-1b was somewhat similar to that of the experimental ECD curve, the configuration of 1a was favored because the distance between H-4 and H-13 was greater than 5Å, which failed to explain the observed ROESY of H-4/H-13.

To support the assignment of C-21 as 21*S*, the ECD spectra of 1c (4*R*, 6*R*, 7*S*, 13*S*, 14*R*, 17*R*, 21*R*) configuration and its enantiomer (*ent*-1c) were calculated. Even though the chiral center on C-21 is far from the examined chromophore in the molecular structure, its configuration affects the overall conformation and conformation equilibria, which in turn translates to differences in ECD spectra. Since the calculated spectra for 1c and *ent*-1c did not match the experimental spectrum well, we suggest the absolute configuration at C-21 be 21*S*. This was further supported by NMR data. In the geometry-optimized structure, the measured distances H_3-27/H-15'/", H-22"/H-12" were greater (4-5 Å) than expected, which failed to explain observed ROESY correlations. Therefore, the absolute configuration was assigned as 1a (4*R*, 6*R*, 7*S*, 13*S*, 14*R*, 17*R*, 21*S*).

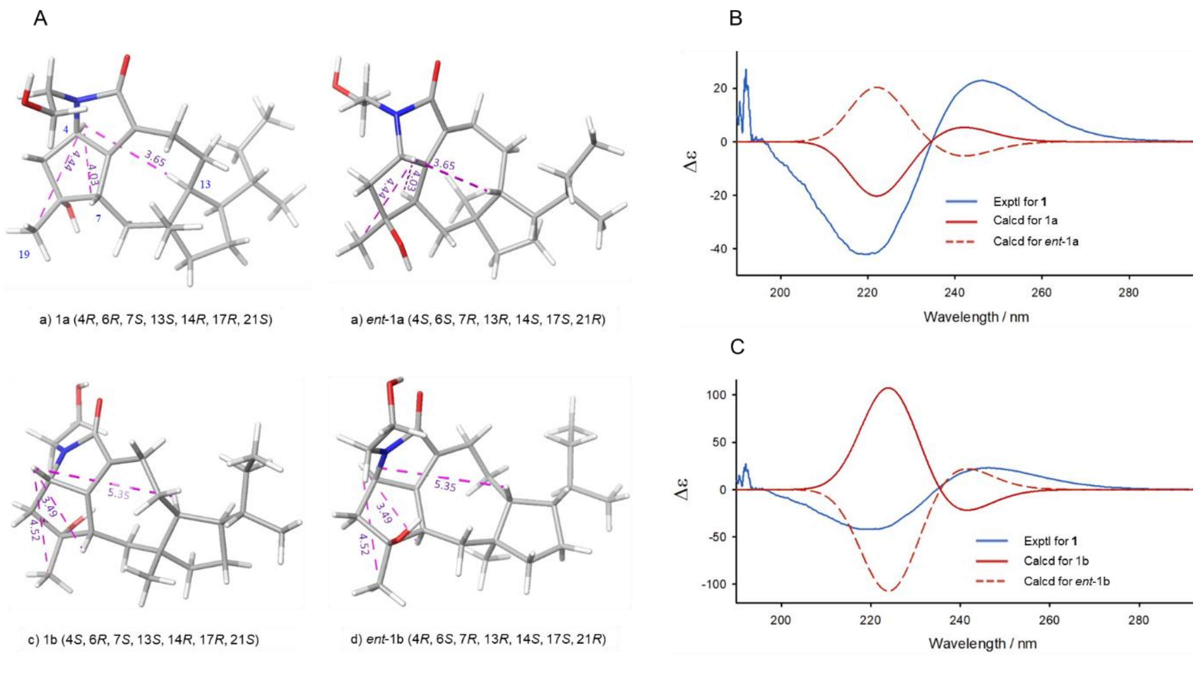

Figure S 105105. (A) Geometry-optimized structures of four stereoisomers of **1** showing the distances H-4/H-7, H-4/H_3-19, and H-4/H-13**.** (B) Comparison of calculated (for 1a and *ent*-1a) and experimental ECD spectra of **1**. (C) Comparison of calculated (for 1b and *ent*-1b) and experimental ECD spectra of **1**. The energies of the calculated spectra were scaled by a scaling factor of 0.82 in both B and C.

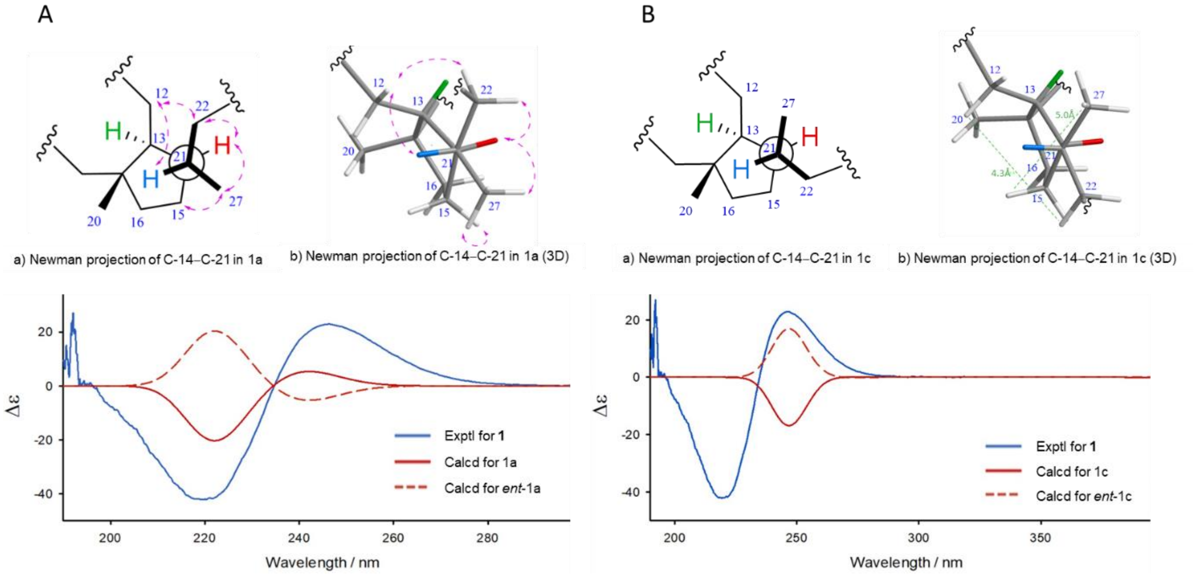

Figure S 106106. Geometry-optimized structure of C-21 epimers. (A) Newman projection of C-14–C-21 showing key ROESY correlations in 2D and 3D (geometry-optimized) structures of 1a with 21*S* configuration. Comparison of calculated (for 1a and *ent*-1a) and experimental ECD spectra of **1**. The calculated energies were scaled by a scaling factor of 0.82. (B) Newman projection of C-14–C-21 in 2D and 3D (geometry-optimized structure showing measured distances H-22"/H-12"and H_3-27/H-15'/") structures of 1c with 21*R* configuration. Comparison of calculated (for 1c and *ent*-1c) and experimental ECD spectra of **1**. The calculated energies were scaled by a scaling factor of 0.88.

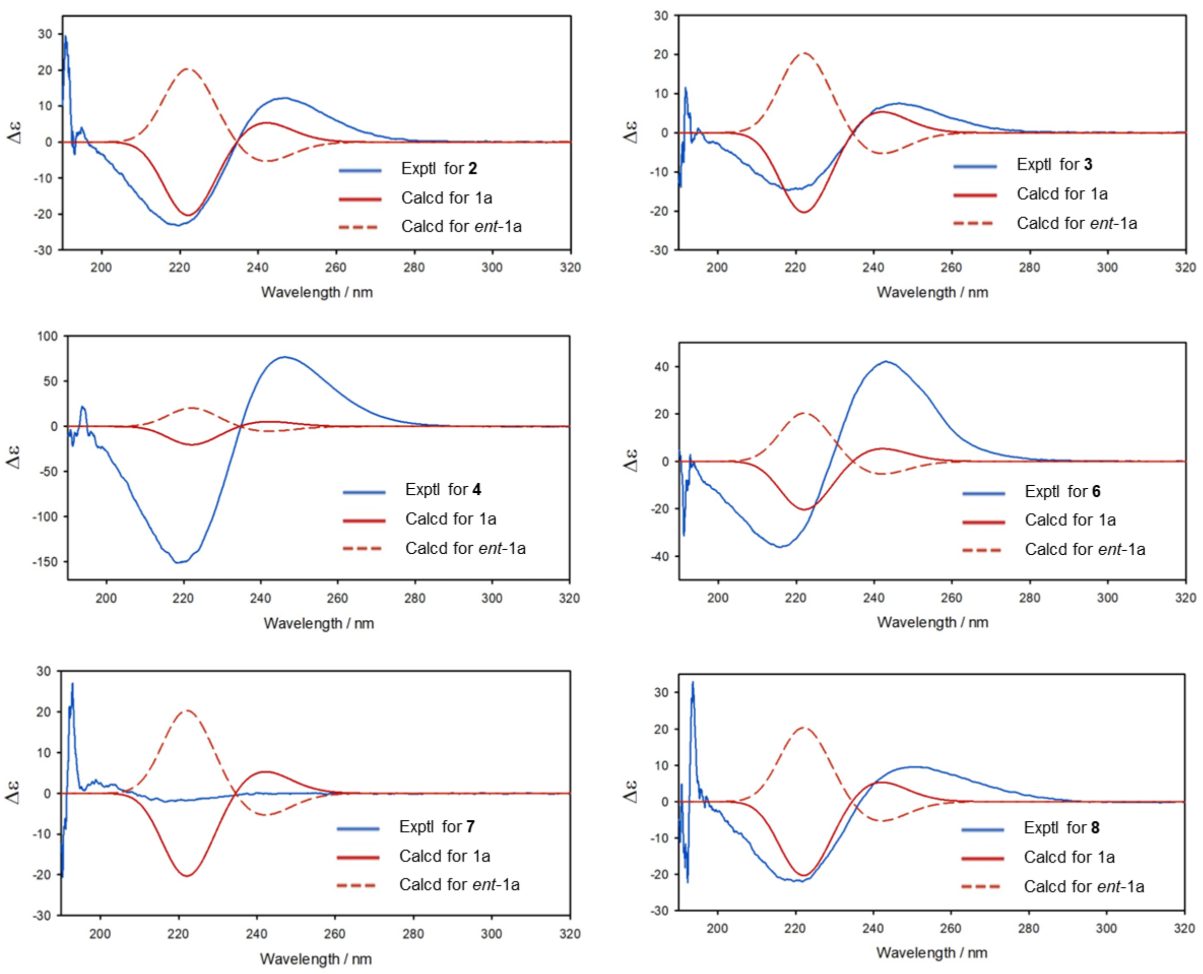

Figure S 107107. (B) Experimental or calculated ECD spectra of **2–8**, except **5**. The energies of the calculated spectra were scaled by a scaling factor of 0.82.

Antibacterial activity of flash fractions

The fungal extract was fractionated into eight flash fractions by flash chromatography. Screening of the antibacterial activity of the flash fractions (Fr. 1 to 8) was performed at a concentration of 100 µg/mL in duplicate. Fractions 5 and 6 were active against only two Gram-positive bacteria: *Enterococcus faecalis* and *Streptococcus agalactiae*. The remaining six fractions (Fr. 1-4, Fr.7, and Fr. 8) were inactive against any of the five bacterial strains (Figure S 108108).

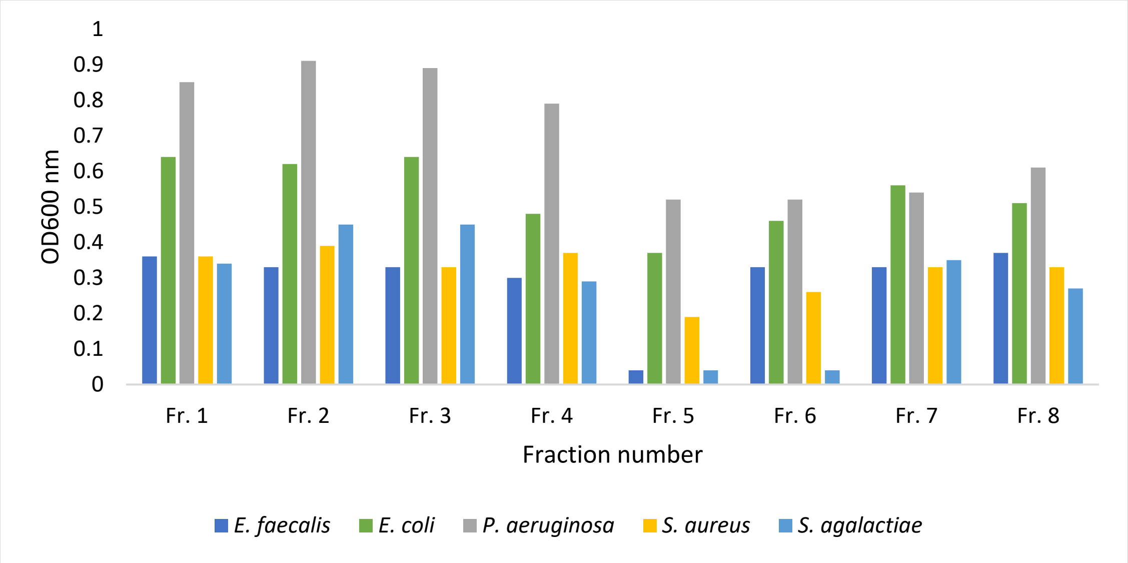

Figure S 108108. Antibacterial effect of flash fractions (Fr. 1- 8).

Determination of minimum inhibitory concentration (MIC) values

The MIC values for the antibacterial activity against *Streptococcus agalactiae* were determined using the broth microdilution method. Compounds **5**, **6**, and **8** were tested at various concentrations (125, 100, 85, 70, 50, 25, 12.5 µM). The results obtained were plotted in a concentration-effect curve and the MIC values were recorded as the concentration (µM) corresponding to 0.05 OD600 nm reading (Figure S 109109). This cut-off value was used to calculate the MIC values of the test compounds. Data are expressed as mean ± SD.

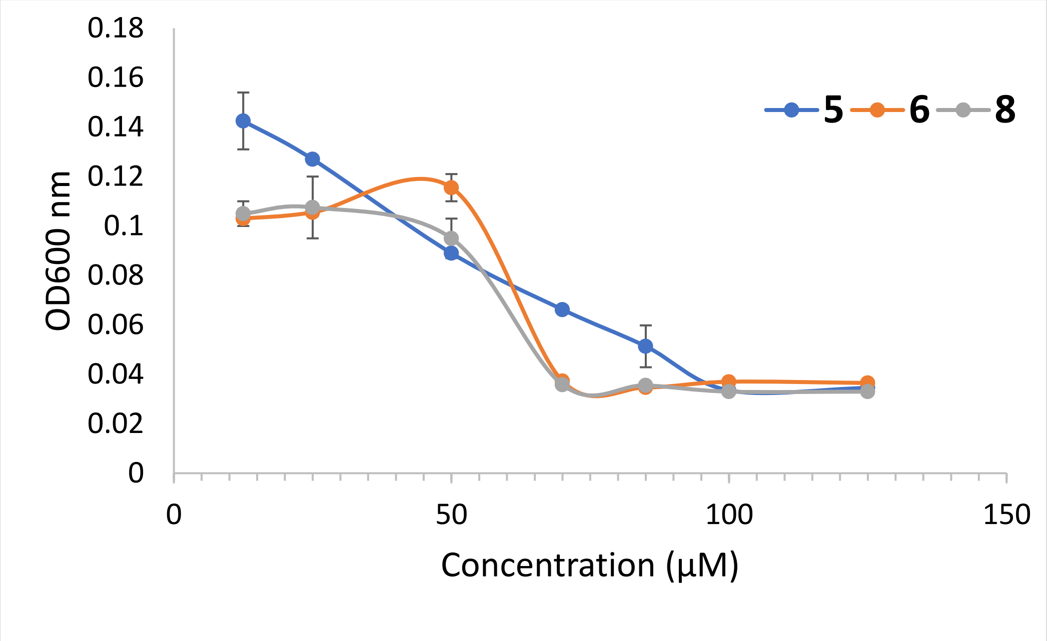

Figure S 109109. Concentration-effect curve for compounds **5**, **6**, and **8** against *Streptococcus agalactiae*.
